# Facility-level characteristics associated with family planning and child immunization services integration in urban areas of Nigeria: a longitudinal analysis

**DOI:** 10.1186/s12889-021-11436-x

**Published:** 2021-07-12

**Authors:** Kate L. Sheahan, Ilene S. Speizer, Jennifer Orgill-Meyer, Siân Curtis, Morris Weinberger, John Paul, Antonia V. Bennett

**Affiliations:** 1 Supported by the Durham Center of Innovation to Accelerate Discovery and Practice Transformation (ADAPT), (CIN 13-410) at the Durham VA Health Care System, Durham, USA; 2 Department of Maternal and Child Health, University of North Carolina at Chapel Hill, Chapel Hill, USA; 3 Department of Government and Public Health, Franklin and Marshall College, Lancaster, USA; 4 Department of Health Policy and Management, University of North Carolina at Chapel Hill, Chapel Hill, USA

**Keywords:** Family planning, Child immunization, Integration, Evaluation, Nigeria, Africa

## Abstract

**Background:**

Unmet need for postpartum contraception is high. Integration of family planning with routine child immunization services may help to satisfy unmet need. However, evidence about the determinants and effects of integration has been inconsistent, and more evidence is required to ascertain whether and how to invest in integration. In this study, facility-level family planning and immunization integration index scores are used to: (1) determine whether integration changes over time and (2) identify whether facility-level characteristics, including exposure to the Nigerian Urban Reproductive Health Initiative (NURHI), are associated with integration across facilities in six urban areas of Nigeria.

**Methods:**

This study utilizes health facility data collected at baseline (*n* = 400) and endline (*n* = 385) for the NURHI impact evaluation. Difference-in-differences models estimate the associations between facility-level characteristics, including exposure to NURHI, and Provider and Facility Integration Index scores. The two outcome measures, Provider and Facility Integration Index scores, reflect attributes that support integrated service delivery. These indexes, which range from 0 (low) to 10 (high), were constructed using principal component analysis. Scores were calculated for each facility. Independent variables are (1) time period, (2) whether the facility received the NURHI intervention, and (3) additional facility-level characteristics.

**Results:**

Within intervention facilities, mean Provider Integration Index scores were 6.46 at baseline and 6.79 at endline; mean Facility Integration Index scores were 7.16 (baseline) and 7.36 (endline). Within non-intervention facilities, mean Provider Integration Index scores were 5.01 at baseline and 6.25 at endline; mean Facility Integration Index scores were 5.83 (baseline) and 6.12 (endline). Provider Integration Index scores increased significantly (*p* = 0.00) among non-intervention facilities. Facility Integration Index scores did not increase significantly in either group. Results identify facility-level characteristics associated with higher levels of integration, including smaller family planning client load, family planning training among providers, and public facility ownership. Exposure to NURHI was not associated with integration index scores.

**Conclusion:**

Programs aiming to increase integration of family planning and immunization services should monitor and provide targeted support for the implementation of a well-defined integration strategy that considers the influence of facility characteristics and concurrent initiatives.

**Supplementary Information:**

The online version contains supplementary material available at 10.1186/s12889-021-11436-x.

## Background

Maternal and infant mortality has profoundly detrimental consequences [1]. Despite national policies resolving to reduce these deaths, Nigeria continues to bear among the highest maternal mortality ratios (MMR) and infant mortality rates (IMR) worldwide [2, 3]. Family planning has the potential to eliminate 25–40% of maternal deaths globally, in part by reducing the number of high-parity pregnancies [4]. Pregnancies spaced fewer than 18 months apart are associated with increased risk of neonatal, perinatal and infant death, low birth weight, small size for gestational age, pre-term delivery, maternal anemia, pre-mature membrane rupture, gestational diabetes, and maternal death [5, 6]. To safeguard the health of women and their babies, the World Health Organization (WHO) promotes interpregnancy intervals of at least 2 years [7]. Clinically, the postpartum period is often defined as the first 6 weeks following birth [8]. However, because of the preponderance of evidence supporting spacing pregnancies 2 years apart, as well as the changing needs and preferences of women throughout this timeframe many policies and programs refer to the postpartum period as up to 12 months to 2 years after childbirth [9–11].

Many women in the postpartum period want to delay their next pregnancy but are not using an effective method of contraception; these women have an unmet need for family planning [12]. In Nigeria, unmet need ranges from 78% in the first 0–5 months postpartum to 51% among women 12–23 months postpartum [13]. In 2012, the Federal Government of Nigeria aimed to increase the modern contraceptive prevalence rate (mCPR) from 10% in that year to 27% by 2020 [14]. By 2019, the mCPR among all women had increased to 14%, but still fell below the government’s goal [15]. Increasing contraceptive use, particularly among postpartum women, remains critical in Nigeria. Policies that focus on increasing access to contraception in the postpartum period encourage healthy birth spacing, and thus contribute to reduced MMR and IMR.

One approach to address high unmet need for family planning in the postpartum period is the integration of family planning services with routine immunization services. The Nigerian government recommends immunization at birth, 6 weeks, 10 weeks, 14 weeks, 9 months, 12 months, and 15 months, which aligns with WHO recommendations [16]. While immunization coverage in Nigeria is lower than in other sub-Saharan African countries, it has been improving in recent years [17]. Integration provides an opportunity to provide immunization while simultaneously addressing the family planning needs of mothers. While numerous integration approaches exist, the two most common are: (1) combining service provision efforts such that family planning and immunization services are provided on the same day at the same facility and (2) providing one of the two services at a facility and referring the woman for the other service at another time or facility [18, 19]. Although the Nigerian Ministry of Health promotes integration to increase access to family planning services, it does not advocate a specific model [20].

Despite its potential to improve service delivery and health outcomes, there is little research evaluating policies and programs that support integration [21, 22]. Integration of family planning and immunization services in sub-Saharan Africa is feasible and may increase contraceptive prevalence without detriment to immunization rates [23–25]; however, recent studies show no significant increase in family planning when family planning services are integrated with immunization visits [26–28]. Systematic reviews highlight the need for more robust evidence about the effects of integration on service delivery and health outcomes [21, 22]. Despite the lack of conclusive evidence, numerous international organizations, donors, and national governments promote policies supporting integration [29, 30].

### Nigerian urban reproductive health initiative

The Nigerian Urban Reproductive Health Initiative (NURHI) is a Bill & Melinda Gates Foundation-funded project launched in 2009 that sought to increase modern contraceptive use in urban areas, especially among the urban poor [31]. Phase I of NURHI (2009 to mid-2015) aimed to dismantle supply and demand side barriers to contraceptive use by: (a) providing facility-level systems strengthening and quality improvement support; (b) generating demand for family planning services and sustained contraceptive use; (c) testing private sector approaches to increase access to and use of family planning among the urban poor; and (d) improving the policy environment for family planning initiatives in urban areas. Within health facilities, NURHI supported systems strengthening to improve the quality and accessibility of family planning services through: (a) improved contraceptive supply chains and logistics; (b) training health providers in family planning counseling and provision; and (c) improving facility level management systems [32]. NURHI promoted integration of family planning into: (a) maternal, newborn, and child health services; (b) post-abortion services; and (c) HIV/AIDS services. The NURHI integration strategy specifically identified integrating family planning into child immunization as a top priority because of its potential to increase family planning uptake among postpartum women. At the facility level, NURHI incorporated the following family planning approaches into immunization services: (a) provision of information, education, and counseling materials on all methods; (b) group counseling; and (c) referral of prospective clients to the family planning clinic [33]. NURHI Phase II (2015–2020) continued its focus on increasing contraceptive prevalence through advocacy, demand generation and service delivery support. NURHI Phase II incorporated evidence from Phase I into its approach and continued to support integration of family planning services into maternal, newborn and child health services, including immunization.

### Measuring integration

Only a few studies have attempted to develop categorical or continuous measures of integration [34, 35]. Generally, studies classify a facility as ‘integrated’ if an intervention to improve integration has been implemented in the facility [25, 36, 37]. More nuanced integration measures may be able to more accurately reflect dynamic service delivery environments and the effect of integration on service delivery and health outcomes. In a previous study, we developed a Provider Integration Index (PII) and Facility Integration Index (FII) and measured the degree of facility-level family planning and immunization services integration attained across 400 facilities in Nigeria and found substantial heterogeneity in provider and facility capacity to offer integrated services [38]. Using these integration indexes as outcomes, this study leverages a longitudinal dataset to identify associations between facility-level characteristics, including exposure to NURHI, and family planning and routine child immunization services integration.

### Facility-level characteristics associated with integration

It is critical to identify facility-level characteristics associated with integration in order to design interventions that effectively support family planning and immunization services integration [26]. Some studies have used qualitative methods to document that contextual characteristics influence integrated care [39]. To our knowledge, this is the first study that utilizes quantitative measures to identify facility-level characteristics associated with family planning and routine immunization services integration. The objectives of this study are to: 1) determine whether facility-level integration changes over time and 2) identify whether facility-level characteristics, including exposure to NURHI, are associated with integration. The results of this study are relevant to policy makers, programmers, and donors seeking to better understand the evolution and facility-level characteristics associated with family planning and immunization services integration so as to develop health interventions that will have the greatest positive impact on critical health outcomes, such as MMR and IMR.

## Methods

### Setting

This study uses data from six cities in Nigeria: Abuja (Nigeria’s capital), Benin City, Ibadan, Ilorin, Kaduna and Zaria. These cities are located in both the northern and the southern regions of Nigeria, which differ culturally and socioeconomically. The country’s more affluent Christian population is concentrated in the south, while the poorer Muslim population predominates in the north.

### Data source and study sample

This study leverages data collected for the NURHI impact evaluation, which was conducted by the Measurement, Learning & Evaluation (MLE) project, led by the Carolina Population Center [31]. Baseline data were collected in 2011 (*n* = 400 facilities) and endline data were collected in 2014 (*n* = 385 facilities).

The sample includes two categories of health facilities: high-volume facilities (HVF) and preferred-provider facilities (PPF). These facilities may be primary or secondary and publicly or privately owned. All public facilities that offered reproductive health services in the study cities were included in the sample, most as HVF within the intervention group and the others as lower volume PPF within the non-intervention group. NURHI implemented the intervention in all of the HVF in the sample. These facilities generally had the highest patient volumes of all facilities in the study cluster - they provided antenatal services to over 1000 women annually and offered child immunization services. The non-intervention group consists of all PPF. The PPF were selected based on the results of a MLE survey, conducted in 2010/2011, of a representative sample of 16,144 women aged 15–49. In this survey, women specified the facility at which they received child health, maternal health, and family planning services. MLE then used that listing to identify the most commonly named facility in each study cluster. The study clusters are the enumeration areas created for the 2006 Population and Housing Census of the Federal Republic of Nigeria. A random sample of clusters was selected from each city based on probability proportional to the population. If the most commonly mentioned facility in the study cluster had already been included in the sample as an HVF, then the second most frequently named facility was included as the PPF. If that facility was already included, then no additional facility was added. Inclusion of the PPF makes certain that the non-intervention group contains facilities commonly utilized by women in the study cities. Overall, there were 112 HVF at baseline and 132 at endline. There were 228 PPF at baseline and 253 at endline. The baseline and endline samples reflect the programmatic reality of shifting intervention groups. At baseline, NURHI identified the facilities where they would work; by endline, they had added some facilities to their sample. Some of the added facilities were part of the non-intervention sample at baseline. To examine whether the shifts within the sample influenced the outcomes, we conducted a sensitivity analysis that restricted the sample to facilities that were present at both baseline and endline. We retained in our sample facilities that did not offer child immunization at baseline in order to assess integration across the range of facilities and circumstances represented by our sample. A second sensitivity analysis was conducted to identify the effects of excluding those facilities from the sample. Table 1 shows facility characteristics at baseline and endline.

**Table 1:** Facility Characteristics by in High-Volume and Preferred-Provider Facilities at Baseline and Endline

Facility Characteristics	Baseline (***n*** = 400)			Endline (***n*** = 385)		
	*HVF (n = 112)*	*PPF* *(n = 288)*	*p-value*	*HVF* *(n = 132)*	*PPF* *(n = 253)*	*p-value*
*Facility Ownership*						
Public Facility	0.79	0.26	0.00	0.79	0.28	0.00
Private Facility	0.21	0.74	0.00	0.21	0.72	0.00
Facility FP client load	73.48	40.71	0.00	143.97	56.31	0.00
Average years of provider experience	14.89	10.77	0.00	15.95	11.15	0.00
*Facility Level*						
Hospital	0.54	0.60	0.31	0.47	0.58	0.05
Primary Health Center	0.45	0.39	0.32	0.52	0.42	0.06
Primary Health Post	0.01	0.01	0.84	0.01	0.01	0.97
*Location*						
Abuja	0.10	0.13	0.40	0.10	0.10	0.71
Benin	0.15	0.19	0.40	0.20	0.19	0.80
Ibadan	0.27	0.11	0.00	0.23	0.11	0.00
Ilorin	0.19	0.18	0.81	0.17	0.19	0.52
Kaduna	0.18	0.25	0.13	0.17	0.24	0.09
Zaria	0.12	0.15	0.44	0.14	0.15	0.64
Providers at facility ever received in-service training on modern FP methods	0.63	0.31	0.00	0.58	0.40	0.00
Facility provides FP but not RI	0.04	0.24	0.00	0.07	0.25	0.00
Facility provides RI but not FP	0.00	0.02	0.10	0.01	0.01	0.69
Facility provides FP and RI services	0.96	0.72	0.00	0.92	0.74	0.00
Facility provides neither FP nor RI services	0.01	0.01	0.69	0.00	0.01	0.21

Notes: *HVF* High-volume facilities. *PPF* Preferred-provider facilities, *FP* Family planning. *RI* Routine childhood immunization. Proportions are reported, except for facility FP client load and average years of provider experience. Facility FP client load is defined as the number of clients who received family planning services in the past twelve months per health worker. Provision of FP services includes those facilities that offer referral only. Some numbers may not add to 1.0 due to rounding

### Survey instruments

This study uses instruments developed by MLE for the NURHI impact evaluation; the instruments are informed by validated tools from the Quick Investigation of Quality [40]. A health facility audit and provider survey was conducted in each facility (see Additional files 1, 2, 3 and 4). One administrator or manager within each facility completed a facility audit, which gathered information about health facility characteristics, family planning service provision, and the extent of family planning integration into maternal, newborn and child health services. Within each facility up to four providers, selected through simple random sampling, completed the provider survey. In smaller facilities with fewer than four providers, all eligible providers were invited to complete the survey. Eligible providers offered family planning and/or maternal, newborn and child health services at the facility. Providers were asked about their training related to provision of family planning as well as their common practices related to integrated care.

### Measures

The outcome measures are a) PII score and b) FII score. Both of these scores were calculated for each facility. Each index score ranges from zero (lowest level of integration) to ten (highest level of integration). The two integration indexes reflect facility-level attributes that support service integration. The PII measures provider skills and practices that support integrated service delivery while the FII measures facility operating norms that support integrated service delivery. The indexes align with guidance provided by Nigeria’s Federal Ministry of Health [41], specifically, the inclusion of concurrent service provision and referral systems within the facility as well as provider behavior during a health visit. To create the indexes, we employed principal component analysis (PCA) on the baseline and endline data using eight input variables that contain variation sufficient to differentiate degrees of integration. Table 2 provides an overview of the constructs included in each index. Additional information about the construction of the indexes is detailed in another paper and can be found in the Additional file 5 [38].

**Table 2:** Provider and Facility Integration Index Components

**Provider Integration Index Components**	
Proportion of providers at facility that offer both RI and FP services	
Proportion of providers at facility that routinely offers FP information during RI or CGM visits	
Average count of FP items that providers at facility tell client during CHS visits	
Proportion of providers at facility that do not request partner consent prior to woman’s receipt of FP services during CHS visit	
Facility provides both child immunization and family planning services	
**Facility Integration Index Components**	
Normal practice at this facility if client wants FP information during CHS visit	
Normal practice at this facility if client wants hormonal method of FP during CHS visit	
Score of operational days when both RI and FP services are offered	

Notes: *RI* Routine Immunization, *FP* Family Planning, *CGM* Child Growth Monitoring, *CHS* Child Health Service CHS visits include either RI or CGM visits, but not sick child visits. Less than 2% of women report that CGM was the primary reason for their visit

Independent variables reflect various facility-level characteristics that may be associated with the degree of family planning and immunization services integration within a health facility, including: a) whether the facility received the NURHI intervention, b) facility family planning client load (defined as the number of clients who received family planning services in the past 12 months per health worker), c) average years of experience of health workers in facility, and d) the proportion of health workers at the facility who have received any in-service training on modern family planning methods. We also include variables to reflect the type of facility (primary healthcare center or hospital), facility ownership (public or private), and the city (the reference city is Abuja).

### Analytic methods

The gold-standard method of identifying the effect of NURHI on integration scores would have been to conduct a randomized controlled trial (RCT). However, a number of constraints and operational realities, including NURHI’s intended focus on public facilities, precluded this. We thus employed quasi-experimental difference-in-differences analyses using multivariate regression models clustered at the facility level to identify associations between facility-level characteristics associated with PII and FII scores . The difference-in-differences approach measures the effect of NURHI and identifies other facility-level characteristics associated with integration index scores. Difference-in-differences also controls for baseline differences between the HVF and PPF, and also for temporal differences that may have occurred due to underlying changes over time [42, 43]. Because the intervention was not randomized, significant differences exist between the HVF (chosen specifically because they were the highest patient-volume facilities in the primary sampling unit and primarily public) and PPF (Table 1). We control for these differences. Use of this quasi-experimental method rests on the parallel trends assumption. While the most empirically sound way to assess parallel trends is to analyze pre-baseline trends, we did not have pre-baseline data available to do this. However, to the best of our knowledge, no concurrent programs or policies were implemented in these facilities that would have altered their respective trajectories. We ran five models to analyze the associations between the independent variables and the PII and the FII; each model includes robust standard errors clustered at the facility level. Models 1–4 each include additional covariates that are analyzed as facility-level characteristics associated with integration; these models show whether and how statistical significance changes with the addition of covariates. Model 5, the preferred model, is the fully specified estimation model to identify potential facility-level characteristics, including exposure to NURHI, associated with PII and FII scores:

1 $$ {\mathrm{Y}}_{\mathrm{it}}=\upalpha +{\upbeta}_1{\left(\mathrm{Year}2014\right)}_{\mathrm{t}}+{\upbeta}_2\kern0.5em {\left(\mathrm{NURHI}\right)}_{\mathrm{g}}+{\upbeta}_3\left(\mathrm{Year}20{14}_{\mathrm{t}}\ast {\mathrm{NURHI}}_{\mathrm{g}}\right)+{\upbeta}_4{\mathrm{X}}_{\mathrm{g}\mathrm{t}}+{\upvarepsilon}_{\mathrm{it}} $$

Y_it_ is the PII or Integration Index score. Year2014 is an indicator variable that is equal to one if the observation pertains to the endline period (post-intervention) and equal to zero if the observation refers to the baseline period (pre-intervention). NURHI is an indicator variable equal to one if the facility was exposed to the NURHI intervention at any time. β_3_ is the estimate of the impact of the NURHI intervention. X indicates a vector of variables, mentioned above, analyzed as potential facility-level characteristics associated with PII and FII scores.

## Results

Results of the sensitivity analysis conducted to analyze shifts within the sample show that of the 432 facilities in the original analysis, 353 appeared in both baseline and endline. Because some facilities did not have complete exposure to the intervention, we anticipated that the estimate of program impact could be biased downward in the full sample. However, the results did not change with the analysis using the restricted sample of facilities present in both the baseline and endline datasets. We thus present results from the full sample.

**Fig. 1:**
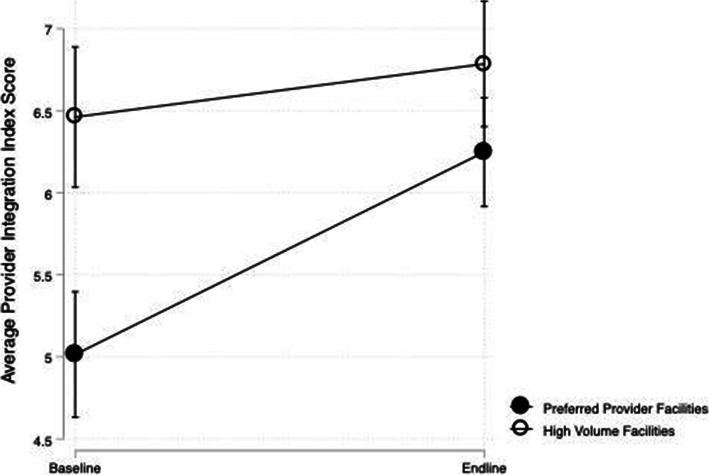
Average Provider Integration Index Scores from Baseline to Endline

The mean PII score of the PPF (5.01, SD = 3.28) and HVF (6.46, SD = 2.28) facilities at baseline differed significantly (*p* < 0.001). The mean FII score of the PPF (5.83, SD = 2.90) and HVF (7.16, SD = 1.92) facilities at baseline also differed significantly (*p* < 0.001). We found an upward trend in PII and FII scores among all facilities over time; however, this increase was statistically significant only for PII scores within the PPF. Table 3 presents mean PII and FII scores while Fig. 1 shows the trend in the average PII score among both HVF and PPF facilities over time.

**Table 3:** Mean Provider and Facility Integration Index Scores In High-Volume and Preferred-Provider Facilities at Baseline and Endline

	***High-Volume Facilities***			***Preferred-Provider Facilities***		
	*Baseline (n = 112)*	*Endline (n = 132)*	*p-value*	*Baseline (n = 288)*	*Endline (n = 253)*	*p-value*
**Mean Provider Integration Index Scores**						
All facilities	6.46	6.79	0.26	5.01	6.25	**0.00**
Public facilities	6.48	6.90	0.19	6.80	7.46	0.06
Private facilities	6.40	6.38	0.98	4.36	5.72	**0.00**
Primary health facilities	7.14	7.48	0.36	5.02	6.51	**0.00**
Hospitals	5.89	5.98	0.84	5.02	6.04	**0.00**
**Mean Facility Integration Index Scores**						
All facilities	7.16	7.36	0.40	5.83	6.12	0.24
Public facilities	7.31	7.44	0.59	6.67	6.78	0.71
Private facilities	6.57	7.04	0.49	5.52	5.86	0.27
Primary health facilities	7.04	7.11	0.84	5.50	6.08	0.13
Hospitals	7.26	7.65	0.238	6.05	6.14	0.77

### Change in provider and facility integration index scores over time

#### Provider integration index scores

**Fig. 2:**
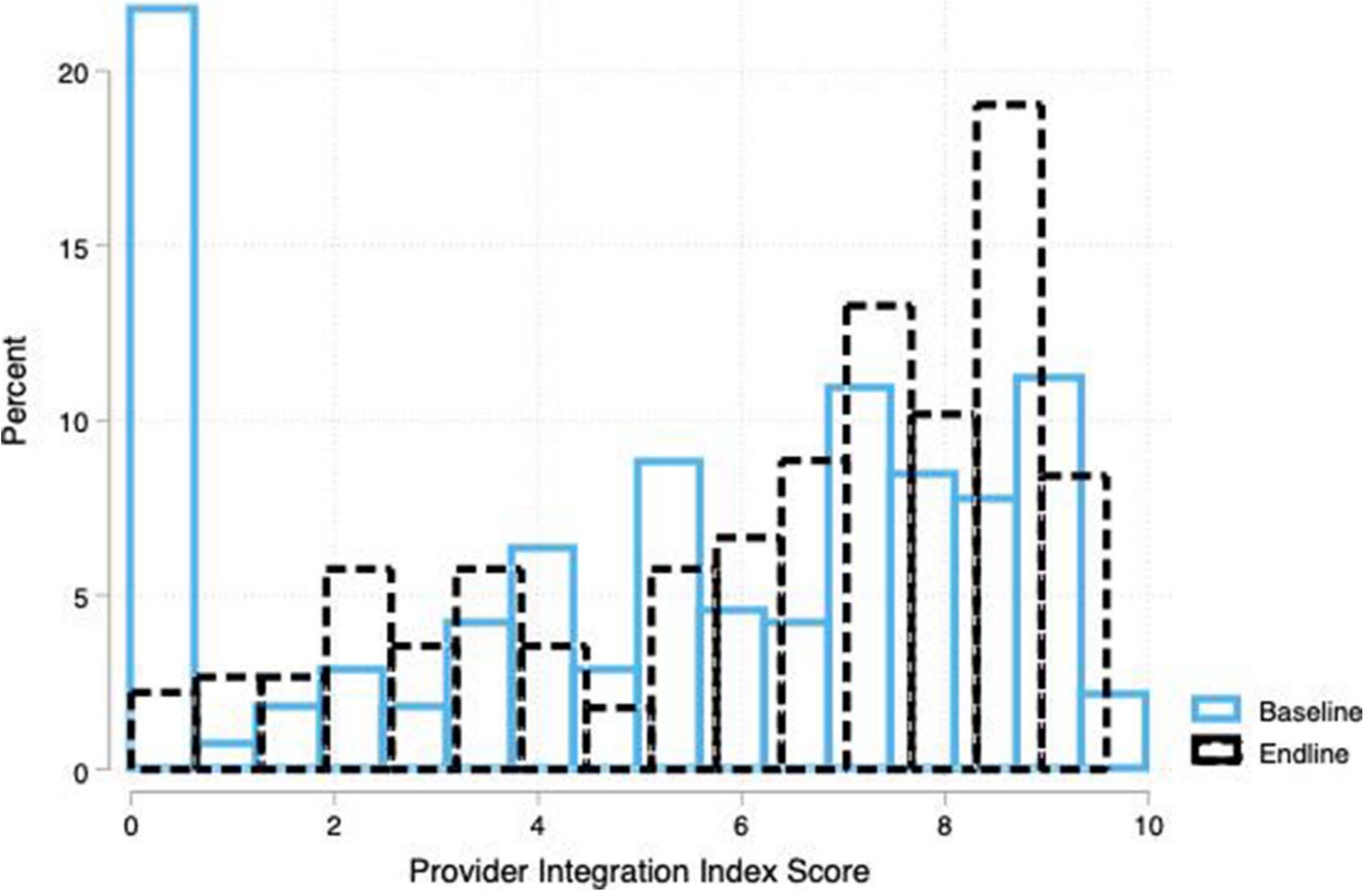
Distribution of Provider Integration Index Scores in Preferred-Provider Facilities at Baseline and Endline

**Fig. 3:**
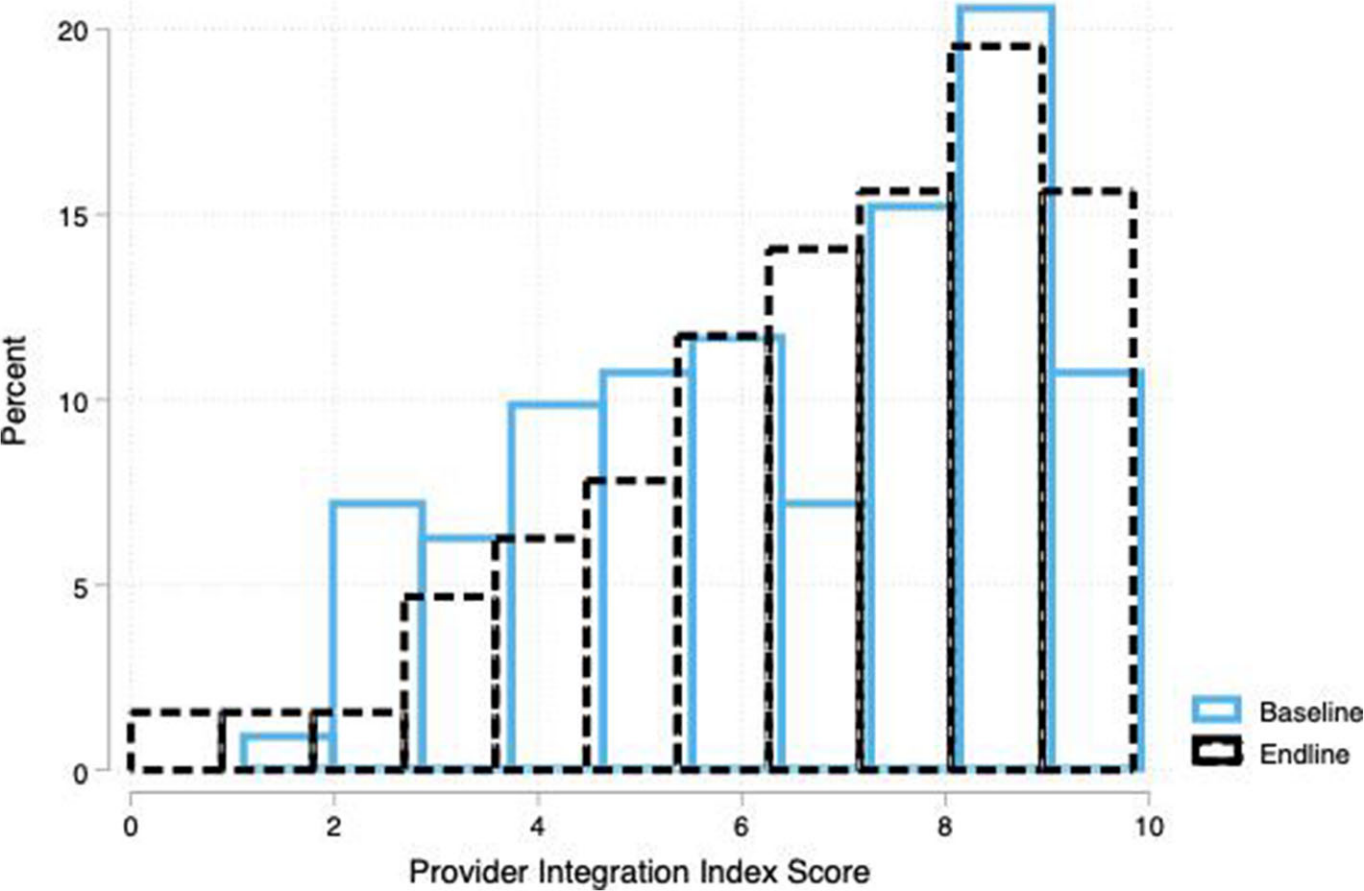
Distribution of Provider Integration Index Scores in High Volume Facilities at Baseline and Endline

Among PPF, the mean change in PII scores from baseline (5.01) to endline (6.25) was statistically significant (*p* < 0.001). The PII scores increased significantly among private facilities, primary care facilities, and hospitals (*p* < 0.05). Public facilities within PPF group did not show a statistically significant increase in PII scores; however, the raw mean score in public PPF facilities remained higher at endline than the raw mean score among private PPF facilities at endline. The average proportion of providers within each PPF that offered both family planning and immunization services increased from 52% at baseline to 61% at endline. At the same time, the proportion of providers within each PPF that routinely offered family planning information during child health services visits increased from 54 to 73%. Additionally, the average number of family planning items that providers at PPF discussed with clients during child health services visits increased from 1.5 to 2.1. Figure 2 shows that the proportion of PPF facilities with a PII score of zero decreased from baseline to endline – specifically, at baseline, 21% of PPF had a score of zero while at endline only 2% did. The decrease in the number of zero scores can also be attributed to improvements in provider capacity to offer integrated services. For example, among PPF facilities that score a zero at baseline, 18% of providers at baseline reported offering family planning information during child health visits while 41% of providers at endline reported the same. Results of the sensitivity analysis show that the increase among PII scores remains significant when we exclude those facilities that do not offer routine immunization services at baseline (*p* = 0.01). Among HVF facilities, the increase in PII scores was not statistically significant within any sub-group of facilities. Figure 3 shows the distribution of PII scores in HVF facilities.

#### Facility integration index scores

Among intervention facilities, the mean FII score was 7.16 at baseline and 7.36 at endline; among non-intervention facilities, the mean score was 5.83 at baseline and 6.12 at endline. These increases are not statistically significant. Figures pertaining to FII scores may be obtained from the first author.

### Facility-level characteristics associated with provider and facility integration index scores

#### Provider integration index scores

Table 4 presents associations between the facility characteristics and PII and FII scores using the fully-specified model. Results from models 1–4 for both indexes are presented in Tables 5 and 6 in the Additional file 5. Our results indicate that facility-level exposure to NURHI is not associated with significant changes in PII scores. Time had a significant positive effect on PII scores (β = 0.90, *p* < 0.001). Facility family planning client load was negatively associated with PII scores (β = − 0.01, *p* < 0.05), as was the average number of years of experience of health care providers in a facility (β = − 0.60, p < 0.001). However, the proportion of providers who attended in-service training on the provision of modern family planning methods was positively associated with PII scores (β = 1.15, p < 0.001). Public ownership of a facility was associated with higher PII scores relative to privately owned facilities (β = 2.04, *p* < 0.001). Facilities located in Benin (β = 1.41, *p* < 0.001), Ibadan (β = 1.60, *p* < 0.001), Ilorin (β = 1.77, *p* < 0.001), and Zaria (β = 0.98, *p* < 0.05) scored higher than those in the reference city, Abuja.

**Table 4:** Association Between NURHI Intervention and Other Facility Characteristics and Provider and Facility Integration Index Scores

Characteristic	Provider Integration Index Score	Facility Integration Index Score
Time	**0.90**	0.21
	(0.21)	(0.20)
NURHI facility	0.14	0.38
	(0.31)	(0.25)
NURHI intervention (time*NURHI facility)	−0.36	0.21
	(0.32)	(0.31)
Facility FP client load	**−0.0100**	−0.0059
	(0.0044)	(0.0059)
Average number of years of provider experience	**−0.60**	− 0.37
	(0.19)	(0.16)
Benin	**1.41**	−0.18
	(0.43)	(0.42)
Ibadan	**1.60**	−0.67
	(0.44)	(0.41)
Ilorin	**1.77**	−0.26
	(0.43)	(0.37)
Kaduna	0.74	−0.80
	(0.39)	(0.39)
Zaria	**0.98**	−1.23
	(0.43)	(0.44)
Public Facility	**2.04**	1.58
	(0.31)	(0.28)
Hospital	0.09	0.77
	(0.27)	(0.25)
Proportion providers received any in-service FP training	**1.15**	1.02
	(0.31)	(0.28)
Constant	**3.67**	5.58
	(0.43)	(0.45)
Observations	751	762

#### Facility integration index scores

FII scores did not change significantly over time. The average number of years of experience among health care providers in a facility was associated with a significant decrease in FII scores (β = − 0.37, *p* < 0.05). Facilities located in Kaduna (β = − 0.80, p < 0.05) and Zaria (β = − 1.23, *p* < 0.01) scored lower than those in Abuja. Several other facility characteristics were associated with higher relative FII scores: (1) the proportion of providers at a facility who had attended in-service trainings on the provision of modern family planning methods (β = 1.02, *p* < 0.001); (2) hospitals scored higher than primary healthcare facilities (β = 0.77, *p* < 0.01); and (3) public ownership of a health facility relative to private ownership (β = 1.58, *p* < 0.001).

## Discussion

Our results show that, aside from a significant increase in PII scores among PPF, integration index scores did not increase significantly over the NURHI project period. The significant increase in the average PII scores among PPF is attributable to increases in the proportion of providers that offered both family planning and immunization services within each facility as well as increases in the proportion of providers that routinely offered family planning information during child health services visits. The average number of family planning items that providers at PPF discussed with clients during child health services visits also increased. This indicates that not only were individual providers at these facilities increasingly able to offer integrated services, but they were discussing a wider range of family planning topics with clients during integrated visits.

NURHI met its primary objective of increasing contraceptive prevalence in the intervention areas [31]. However, our results indicate that exposure to the program was not associated with changes in PII or FII scores. Several plausible explanations exist for the lack of association between NURHI and integration index scores. First, the family planning client load within NURHI facilities increased significantly over the project period. This may have been due to improved family planning service provision at the facilities and demand generation activities in the project cities leading to increased demand for family planning within the communities. Therefore, while NURHI articulated a strategy to integrate family planning into immunization services, health workers in these facilities may have had to prioritize non-integrated family planning provision in order to provide services to the additional clients. Providers and facilities found it challenging to incorporate family planning information and services into immunization services. This would reinforce research by Vance et al. (2014) that questions the feasibility of effectively providing family planning information during immunization appointments [28]. Future studies could collect in-depth information from providers and clients to understand their perspectives on facilitators and barriers to family planning and immunization services integration. This would provide valuable information for the development and support of integration strategies. It is important to consider that facilities may not need to attain very high levels of integration in order to have an impact on service delivery and health outcomes. It is also important to consider that integration models that focus on ensuring effective referral mechanisms are more beneficial than those aiming to provide comprehensive family planning information and services during immunization visits. It is also possible that integration, as measured by the PII and FII, reaches a ‘ceiling’. Indeed, it is possible that the PII scores increased in the PPF because there was room for improvement, while the HVF may have been approaching a ceiling. Understanding the extent to which integration can be achieved within specific service delivery models, and whether and how the degree of integration affects outcomes is an important area for future research.

Finally, our results pinpoint several facility-level characteristics associated with integration index scores, including location, family planning client load, years of provider experience, provider training, and facility ownership (public or private). First, facility location was associated with both PII and FII scores. Facilities in Kaduna and Zaria had lower FII scores than facilities in Abuja; standard facility practices in these cities were less likely to link women attending for child health visits to family planning information or services on the same day. However, facilities located in Benin, Ibadan, Ilorin, and Zaria had higher PII scores than those in Abuja. Programs should consider demand-side preferences when developing integration strategies. It is crucial to ensure that specific integration approaches are acceptable to communities and providers so that immunization coverage and family planning prevalence does not fall, particularly in regions where immunization coverage and contraceptive prevalence is already low or has limited community acceptance. This is particularly relevant in northern Nigeria, where communities have boycotted immunizations because of widespread belief that immunizations were infused with anti-fertility drugs [44].

Second, the negative association between PII score and facility family planning client load suggests that providers may be less able to offer high-quality integrated care in busier settings. This finding reinforces studies highlighting that heavier workloads challenge integration efforts and result in poorer quality of care [45]. One suggestion may be to increase staffing to manage workloads; however, chronic provider shortages make this an unlikely option in many contexts. Family planning service quality impacts family planning use [46]. Poor quality integrated care could prove detrimental to family planning utilization and immunization coverage. It is therefore important to consider whether, in what contexts, and how integration should be promoted.

Third, provider experience is associated with lower PII and FII scores. Though it is commonly assumed that more experienced providers offer higher quality care, some research suggests an inverse relationship between years of clinical practice and quality of care [47]. One explanation for this is that provider “toolboxes” are developed during pre-service training and may not be regularly updated [48]. Further, providers with more years of experience may be less likely to adopt new approaches or incorporate new information or services into their practice [49]. Within the context of integration, individual providers with more years of experience may be less likely to expand their practice by providing family planning information and services during child health visits. This could also influence facility level norms, whereby facilities staffed by more experienced providers may be less likely to implement new systems that facilitate integration. Provision of in-service training may counterbalance the negative association between provider experience and integration index scores. Providers who receive in-service training on modern methods of family planning may be more apt to provide these services and discuss a wider range of family planning information during immunization visits [36]. In turn, increased provider capacity to offer family planning information and services may facilitate facility-level practices that promote integration.

Lastly, publicly owned facilities score higher on integration than those that are privately owned. The private sector provides more than one-third of family planning services in low and middle-income countries globally and is an important source of contraception for women in Nigeria [50]. Compared to the public sector, the private sector plays a limited role in the provision of immunization services in Nigeria [51]. Public facilities may demonstrate a greater capacity to provide integrated services because they are more accountable to government standards and guidelines, which emphasize service integration. They may also have greater access to the vaccine delivery infrastructure, such as cold chain equipment. The Nigeria Strategy for Immunization and Primary Health Care System Strengthening calls for increased engagement of the private sector in the provision of immunizations [52]. High-volume private facilities, such as the ones in this study, could be prioritized for inclusion in government supply chains. To ensure equitable access to both immunization and family planning services, it is important to understand and support the service delivery environment in both public and private facilities.

This research has several limitations, including possible social desirability and recall bias during interviews. Additionally, as with most quasi-experimental designs, we cannot eliminate the risk of bias. An RCT would have been the ideal method of identifying the impact of NURHI on integration index scores. However, operational constraints and program priorities rendered this unfeasible. NURHI had a stated focus on high volume, public facilities and implemented the intervention within all such facilities in the study cities. Therefore, it was not possible to create a comparison group with a balance of public and private facilities, and the HVF and PPF differ significantly in some characteristics. We have controlled for these significant differences, and the difference-in-differences model accommodates differences in intervention and non-intervention groups assuming the parallel trend assumption is met. In this case, we believe that the parallel trend holds as we could not identify any contextual factors that would alter the trajectories of the HVF and PPF during the intervention period. However, due to lack of pre-trend data, we cannot empirically assess the parallel trend assumption. If the assumption is violated, it is possible that these differences bias the estimates of NURHI’s impact on integration index scores. For this reason, we interpret our results conservatively by focusing on the association between exposure to NURHI and integration index scores rather than the impact of NURHI. Additionally, our sample was drawn from select cities; therefore, it is not representative of all facilities, particularly rural facilities, or all cities in Nigeria. Thus, our results are not generalizable to all facilities in Nigeria or other contexts. Finally, a better understanding of the fidelity with which NURHI’s integration strategy was implemented would enable a more accurate analysis of the association between particular approaches and the extent of integration attained; unfortunately, we lack this information. Strengths balance the limitations of this study. First, although facility randomization was not possible, the study does leverage an innovative sampling strategy that ensures that the PPF are those facilities that the women throughout the study cities do commonly attend for reproductive health services. Aside from the HVF, they are the most commonly utilized facilities in the study cities. Second, the large sample size and longitudinal design enable us to track the facility-level characteristics and evolution of family planning and immunization services integration in a variety of facilities commonly attended by urban women in Nigeria over time. This is important information in light of the Nigerian government’s goal to reduce MMR and IMR by increasing contraceptive prevalence, in part, by reaching more postpartum women through integration of family planning and child immunization services.

## Conclusion

Understanding the facility-level characteristics associated with family planning and child immunization services integration is an important step in optimizing its potential to increase postpartum contraception use. Programs seeking to increase integration of immunization and family planning services should provide monitoring and support that focuses specifically on helping health workers to provide high-quality integrated services. Further, health services implementers and policy makers should consider the influence of facility characteristics and concurrent initiatives when designing and implementing integrated service delivery.

More evidence is needed to better understand whether and how varying degrees of integration affect service delivery and health outcomes. Future research should test different integration strategies, for example, how to optimize each of the recommended immunization appointments and should also monitor intervention fidelity. Such research should investigate the effects of integration on contraceptive uptake and continuation, client knowledge, immunization coverage, service delivery efficiency and quality, cost, and provider workload. The facilitators and barriers to integration should be explored from both provider and client perspectives so that implemented approaches are sustainable, quality of care is maintained, and contraceptive and immunization coverage improves.

## Supplementary Information

**Additional file 1.** Baseline Health Facility Audit.

**Additional file 2.** Baseline Provider Survey.

**Additional file 3.** Endline Health Facility Audit.

**Additional file 4.** Endline Provider Survey.

**Additional file 5: Appendix A.** Additional Details on Construction of the Integration Indexes. **Appendix B**. Association Between NURHI Intervention and Other Facility Characteristics and Provider and Facility Integration Index Scores.

## Data Availability

Data from this study and all survey tools and documentation are available upon request through the MLE Dataverse website at: https://dataverse.unc.edu/dataverse/mle

## Abbreviations

AIDS: Acquired Immune Deficiency Syndrome
FII: Facility Integration Index
HVF: High-Volume Facility
HIV: Human Immunodeficiency Virus
IMR: Infant mortality rate
LGA: Local Government Area
MMR: Maternal mortality ratio
MLE: Measurement Learning & Evaluation Project
mCPR: Modern contraceptive prevalence rate
NURHI: Nigerian Urban Reproductive Health Initiative
PCA: Principal component analysis
PII: Provider Integration Index
PPF: Preferred-Provider Facility
WHO: World Health Organization
