# Supplementary material for: Facility-level characteristics associated with family planning and child immunization services integration in urban areas of Nigeria: a longitudinal analysis
Source: BMC Public Health. 2021 Jul 12;21:1379. doi: 10.1186/s12889-021-11436-x (PMC8274034; doi:10.1186/s12889-021-11436-x)
Supplement: Supplementary file 1 — Additional file 1. Baseline Health Facility Audit. [file 12889_2021_11436_MOESM1_ESM.pdf]

MLE MEASUREMENT, LEARNING & EVALUATION PROJECT FOR THE URBAN REPRODUCTIVE HEALTH INITIATIVE

Measurement, Learning & Evaluation (MLE) Project
Health Facility audit – Nigeria - 2011

CITY NAME & CODE ______________________________<br>(Abuja=1, Benin=2, Ibadan=3, Ilorin=4, Kaduna=5, Zaria=6)	[__]
LGA NAME & CODE______________________________	[__|__|__]
LOCALITY NAME & CODE ______________________________	[__|__|__|__]
FACILITY NAME AND CODE ______________________________	[__|__|__|__|__]
FACILITY PHYSICAL ADDRESS______________________________	

LOCATION OF FACILITY<br>GPS Reading<br>Altitude	[ ][ ][ ][ ]
Latitude	N [ ][ ] [ ][ ][ ][ ][ ]
Longitude	E [ ][ ][ ] [ ][ ][ ][ ][ ]

TYPE OF HEALTH FACILITY	PUBLIC SECTOR<br>GOVT. HOSPITAL.......................... 11<br>WOMEN AND CHILDREN HOSPITAL 12<br>CHILD WELFARE CLINIC............... 13<br>GOVT. HEALTH CENTRE................ 14<br>GOVT. HEALTH POST/DISPENSARY 15<br>MATERNITY HOME........................ 16<br>OTHER PUBLIC________________ 18<br>(SPECIFY)<br><br>PRIVATE SECTOR<br>PRIVATE HOSPITAL..................... 21<br>PRIVATE CLINIC.......................... 22<br>PRIVATE DOCTOR'S OFFICE.......... 23<br>NURSING/MATERNITY HOME........... 24<br>OTHER PRIVATE________________ 29<br>(SPECIFY)<br>FBO<br>MISSION HOSPITAL...................... 31<br>FAITH-BASED HOME/HEALTH CENTRE................................. 32<br><br>OTHER<br>OTHER NGO HOSPITAL................ 41<br>OTHER NGO CLINIC..................... 42<br>Other________________________96<br>(Specify)
HEALTH FACILITY VOLUME TYPE	HIGH VOLUME..1<br>OTHER..2

INTERVIEWER VISITS

**VISIT No.**	**1**	**2**	**3**	**FINAL VISIT**
DATE	DAY/ MONTH/ YEAR<br>[____/_____/_11_]	DAY/ MONTH/ YEAR<br>[____/_____/__11_]	DAY/ MONTH/ YEAR<br>[____/_____/__11_]	DAY [____|____]<br>MONTH [____|____]<br>YEAR [_2_|_0_|_1_|_1_]
INTERVIEWER'S NAME	________________	________________	________________	____________________
INTERVIEWER CODE	☐☐☐	☐☐☐	☐☐☐	☐☐☐
RESULT*	☐	☐	☐	☐
**NEXT VISIT:**<br>**DATE**<br>**TIME**	[___/____/11_]<br>☐☐ ☐☐<br>H H M M	[___/____/11_]<br>☐☐ ☐☐<br>H H M M	[___/____/11_]<br>☐☐ ☐☐<br>H H M M	TOTAL NO. OF VISITS ☐

***RESULT CODES:**

1. COMPLETED
2. FACILITY MOVED OR IS DESTROYED
3. RESPONDENT NOT AVAILABLE (NOT AT WORK, ON STRIKE, ETC)
4. RESPONDENT REFUSED
5. PARTLY COMPLETED
6. POSTPONED
7. OTHER ______________________________ (Specify)

LANGUAGE

	ENGLISH	HAUSA	YORUBA	IGBO	PIDGIN	OTHER (SPECIFY)	TRANSLATOR USED?
LANGUAGE OF INTERVIEW	1	2	3	4	5	6____________	YES 1 NO 2

POSITION OF MAIN PERSON INTERVIEWED - **CIRCLE ONE**

CLINIC MANAGER/FACILITY ADMINISTRATOR……………….…..01
PHYSICIAN……………………………………………………..02
NURSE……………………………………………………………03
MIDWIFE…………………………………………………………04
NURSE/ MIDWIFE…………………………………………..…05
CHEW……………………………………………………………06
OTHER _________ _____________________________96
(SPECIFY)

NAME____

MOBILE PHONE NUMBER______________________________

SEX OF MAIN PERSON INTERVIEWED

MALE…………………………………..1
FEMALE……………………………..…2

**SUPERVISOR**	**OFFICE EDITOR**	**KEYED BY**
NAME………………………………	NAME………………………………	NAME………………………………………
CODE: ☐☐	CODE: ☐☐	CODE: ☐☐
DATE [__/ ___/ 11_]<br>DD MM YY	DATE [__/ ___/ 11_]<br>DD MM YY	DATE [__/ __/ 11_]<br>DD MM YY

GENERAL FACILITY INFORMATION

Source	Questions	Coding	Skip/Notes
Q1.	RECORD THE TIME<br>(IN 24 HOUR FORMAT) Hour …………. [ ][ ]	Minutes ………… [ ][ ]	
Q2.	In what year did this facility open?<br><br>**PROBE, IF RESPONDANT SAYS DON'T KNOW: THIS IS VERY IMPORTANT**. Can you tell me how old this facility is? For example, would you say it is about 3 years old? 7 years old? (etc.)<br><br>**FILL IN EITHER YEAR OPENED OR YEARS OLD.**	Year opened . . . . . . [ ][ ][ ][ ]<br>**OR**<br>Years old . . . . [ ][ ]<br>Don't know 9998	
Q3.	On average, how many days per week is the facility open?	Days per week. . . . . [ ]	
Q4.	What time does the facility typically open?<br><br>WRITE ANSWER ON 24-HOUR CLOCK (IE. IF OPENS AT 7:00 AM, MARK 07:00	[ ][ ] : [ ][ ]<br>Open 24 hours a day………..99:91 →	**Q6a**
Q5.	What time does the facility typically close?<br><br>WRITE ANSWER ON 24-HOUR CLOCK (IE. IF CLOSES AT 7:00 PM, MARK 19:00	[ ][ ] : [ ][ ]	
Q6a.	Is this facility linked with PPFN or SFH or another organization that provides family planning methods and materials at a discounted rate or for free?	Yes……………………………….1<br>No……………………………….2 →<br>Don't know……………………….8 →	 **Q7**<br>**Q7**
	6b. What is the name of this organization?	6c.What year did this facility begin to associate with each organization named?	
	1.	Year …………….. [ ][ ][ ][ ]<br>Don't know……………….9998	
	2.	Year …………….. [ ][ ][ ][ ]<br>Don't know……………….9998	
	3.	Year …………….. [ ][ ][ ][ ]<br>Don't know……………….9998	
	4	Year …………….. [ ][ ][ ][ ]<br>Don't know……………….9998	
Q7.	How many **permanent** staff of each type (cadre) does this facility have?<br><br>1. Obstetrician/Gynecologists (OB/GYN)<br>2. General surgeons<br>3. Pediatricians<br>4. General physicians<br>5. Theatre nurse<br>6. Nurse/Midwives<br>7. Nurses<br>8. Midwives<br>9. Community health extension workers (CHEWs)<br>10. Community health officers (CHO)<br>11. VCT Counsellor<br><br>**FOR LARGE MEDICAL HOSPITALS AND COLLEGES, PLEASE PROBE TO ESTIMATE TO YOUR BEST ABILITY.**<br>***NOTE: PERMANENT STAFF DOES NOT INCLUDE DOCTORS IN RESIDENCY TRAINING INTERNS OR NYSC**	OB/GYNS [___|___|___]<br>GENERAL SURGEONS [___|___|___]<br>PEDIATRICIANS [___|___|___]<br>GENERAL PHYSICIANS [___|___|___]<br>THEATRE NURSES [___|___|___]<br>NURSE/MIDWIVES [___|___|___]<br>NURSES [___|___|___]<br>MIDWIVES [___|___|___]<br>CHEW [___|___|___]<br>CHO [___|___|___]<br>VCT PROVIDER [___|___|___]	

Now I would like to ask you some questions about the permanent staff who work in this facility. We would like to ask their names, positions and departments, so that we can randomly sample a few to interview using a separate questionnaire. These few will then represent the group. Remember that this is for research purposes only and we will keep all details strictly confidential.

STAFF

LIST NAMES OF ALL PERMANENT STAFF INVOLVED IN PROVIDING REPRODUCTIVE HEALTH SERVICES, INCLUDING FAMILY PLANNING, MATERNAL AND CHILD HEALTH AND STI/VCT/HIV SERVICES. CODE "YES" IN Q8c FOR THOSE PROVIDERS ON DUTY TODAY AND "NO" FOR THOSE NOT ON DUTY AT ANY TIME TODAY.
FOR EACH PERMANENT SERVICE PROVIDER WHO IS **NOT** ON DUTY TODAY, WRITE "99" (NOT ELIGIBLE) IN **Q8d**. FOR ALL PERMANENT SERVICE PROVIDERS WHO **ARE** ON DUTY TODAY, ASSIGN A NUMBER TO EACH OF THEM (SERIALIZE) IN Q8d STARTING WITH "01" TO THE LAST NUMBER. DO NOT CONSIDER THE "99" AS PART OF THE NUMBERING.

FOR FACILITIES WITH FOUR OR FEWER PROVIDERS ON DUTY TODAY, INTERVIEW ALL OF THEM. FOR FACILITIES WITH FIVE OR MORE PROVIDERS ON DUTY TODAY, WRITE ALL NUMBERS FROM Q8d (EXCEPT FOR "99") ON SMALL PIECES OF PAPER AND RANDOMLY SELECT FOUR PROVIDERS. ONCE YOU HAVE BALLOTED/SELECTED FOUR PROVIDERS FROM Q8d, CAREFULLY AND NEATLY CIRCLE THE NUMBERS IN Q8d FOR THOSE SELECTED.

Q8a. No. of staff	Q8b. NAME	Q8c. Is NAME scheduled to be on duty any time today?	Q8d. Serial number of sampled on-duty staff	Q8e. POSITION CODE	Q8f. Does NAME work full-time?	Q8g. SEX	Does NAME provide service(s)? *Please indicate by checking the box of the services that NAME provides.*			
							Q8h. FAMILY PLANNING	Q8i. MATERNAL HEALTH	Q8j. CHILD HEALTH	Q8k. VCT/STI/HIV SERVICES
(01)		YES . . . . .1<br>NO 2			YES. . .1<br>NO. . . .2	MALE . . . 1<br>FEMALE...2	YES . . .. .1<br>NO 2	YES . . . . . .1<br>NO 2	YES . . . . . .1<br>NO 2	YES . . . . . .1<br>NO 2
(02)		YES . . . . .1<br>NO 2			YES. . .1<br>NO. . . .2	MALE . . . 1<br>FEMALE...2	YES . . .. .1<br>NO 2	YES . . . . . .1<br>NO 2	YES . . . . . .1<br>NO 2	YES . . . . . .1<br>NO 2
(03)		YES . . . . .1<br>NO 2			YES. . .1<br>NO. . . .2	MALE . . . 1<br>FEMALE...2	YES . . .. .1<br>NO 2	YES . . . . . .1<br>NO 2	YES . . . . . .1<br>NO 2	YES . . . . . .1<br>NO 2
(04)		YES . . . . .1<br>NO 2			YES. . .1<br>NO. . . .2	MALE . . . 1<br>FEMALE...2	YES . . .. .1<br>NO 2	YES . . . . . .1<br>NO 2	YES . . . . . .1<br>NO 2	YES . . . . . .1<br>NO 2
(05)		YES . . . . .1<br>NO 2			YES. . .1<br>NO. . . .2	MALE . . . 1<br>FEMALE...2	YES . . .. .1<br>NO 2	YES . . . . . .1<br>NO 2	YES . . . . . .1<br>NO 2	YES . . . . . .1<br>NO 2
(06)		YES . . . . .1<br>NO 2			YES. . .1<br>NO. . . .2	MALE . . . 1<br>FEMALE...2	YES . . .. .1<br>NO 2	YES . . . . . .1<br>NO 2	YES . . . . . .1<br>NO 2	YES . . . . . .1<br>NO 2
(07)		YES . . . . .1<br>NO 2			YES. . .1<br>NO. . . .2	MALE . . . 1<br>FEMALE...2	YES . . .. .1<br>NO 2	YES . . . . . .1<br>NO 2	YES . . . . . .1<br>NO 2	YES . . . . . .1<br>NO 2
(08)		YES . . . . .1<br>NO 2			YES. . .1<br>NO. . . .2	MALE . . . 1<br>FEMALE...2	YES . . .. .1<br>NO 2	YES . . . . . .1<br>NO 2	YES . . . . . .1<br>NO 2	YES . . . . . .1<br>NO 2
(09)		YES . . . . .1<br>NO 2			YES. . .1<br>NO. . . .2	MALE . . . 1<br>FEMALE...2	YES . . .. .1<br>NO 2	YES . . . . . .1<br>NO 2	YES . . . . . .1<br>NO 2	YES . . . . . .1<br>NO 2
(10)		YES . . . . .1<br>NO 2			YES. . .1<br>NO. . . .2	MALE . . . 1<br>FEMALE...2	YES . . .. .1<br>NO 2	YES . . . . . .1<br>NO 2	YES . . . . . .1<br>NO 2	YES . . . . . .1<br>NO 2
(11)		YES . . . . .1<br>NO 2			YES. . .1<br>NO. . . .2	MALE . . . 1<br>FEMALE...2	YES . . .. .1<br>NO 2	YES . . . . . .1<br>NO 2	YES . . . . . .1<br>NO 2	YES . . . . . .1<br>NO 2
(12)		YES . . . . .1<br>NO 2			YES. . .1<br>NO. . . .2	MALE . . . 1<br>FEMALE...2	YES . . .. .1<br>NO 2	YES . . . . . .1<br>NO 2	YES . . . . . .1<br>NO 2	YES . . . . . .1<br>NO 2
(13)		YES . . . . .1<br>NO 2			YES. . .1<br>NO. . . .2	MALE . . . 1<br>FEMALE...2	YES . . .. .1<br>NO 2	YES . . . . . .1<br>NO 2	YES . . . . . .1<br>NO 2	YES . . . . . .1<br>NO 2
(14)		YES . . . . .1<br>NO 2			YES. . .1<br>NO. . . .2	MALE . . . 1<br>FEMALE...2	YES . . .. .1<br>NO 2	YES . . . . . .1<br>NO 2	YES . . . . . .1<br>NO 2	YES . . . . . .1<br>NO 2

CODE: Obstetrician/Gynecologists=01 General surgeons=02 Pediatricians=03 General physicians=04 Theatre nurse=05 Nurse/Midwives=06 Nurses=07 Midwives=08 CHEWs=09 Community health officers (CHO)=10 VCT Counselors=11 Other=96

**STAFF**										
Q8a. No. of staff	Q8b. NAME	Q8c. Is NAME scheduled to be on duty any time today?	Q8d. Serial number of sampled on-duty staff	Q8e. POSITION CODE	Q8f. Does NAME work full-time?	Q8g. SEX	Does NAME provide service(s)? *Please indicate by checking the box of the services that NAME provides.*			
							Q8h. FAMILY PLANNING	Q8i. MATERNAL HEALTH	Q8j. CHILD HEALTH	Q8k. VCT/STI/HIV SERVICES
(15)		YES . . . . .1<br>NO 2			YES. . .1<br>NO. . . .2	MALE . . . 1<br>FEMALE...2	YES . . .. .1<br>NO 2	YES . . . . . .1<br>NO 2	YES . . . . . .1<br>NO 2	YES . . . . . .1<br>NO 2
(16)		YES . . . . .1<br>NO 2			YES. . .1<br>NO. . . .2	MALE . . . 1<br>FEMALE...2	YES . . .. .1<br>NO 2	YES . . . . . .1<br>NO 2	YES . . . . . .1<br>NO 2	YES . . . . . .1<br>NO 2
(17)		YES . . . . .1<br>NO 2			YES. . .1<br>NO. . . .2	MALE . . . 1<br>FEMALE...2	YES . . .. .1<br>NO 2	YES . . . . . .1<br>NO 2	YES . . . . . .1<br>NO 2	YES . . . . . .1<br>NO 2
(18)		YES . . . . .1<br>NO 2			YES. . .1<br>NO. . . .2	MALE . . . 1<br>FEMALE...2	YES . . .. .1<br>NO 2	YES . . . . . .1<br>NO 2	YES . . . . . .1<br>NO 2	YES . . . . . .1<br>NO 2
(19)		YES . . . . .1<br>NO 2			YES. . .1<br>NO. . . .2	MALE . . . 1<br>FEMALE...2	YES . . .. .1<br>NO 2	YES . . . . . .1<br>NO 2	YES . . . . . .1<br>NO 2	YES . . . . . .1<br>NO 2
(20)		YES . . . . .1<br>NO 2			YES. . .1<br>NO. . . .2	MALE . . . 1<br>FEMALE...2	YES . . .. .1<br>NO 2	YES . . . . . .1<br>NO 2	YES . . . . . .1<br>NO 2	YES . . . . . .1<br>NO 2
(21)		YES . . . . .1<br>NO 2			YES. . .1<br>NO. . . .2	MALE . . . 1<br>FEMALE...2	YES . . .. .1<br>NO 2	YES . . . . . .1<br>NO 2	YES . . . . . .1<br>NO 2	YES . . . . . .1<br>NO 2
(22)		YES . . . . .1<br>NO 2			YES. . .1<br>NO. . . .2	MALE . . . 1<br>FEMALE...2	YES . . .. .1<br>NO 2	YES . . . . . .1<br>NO 2	YES . . . . . .1<br>NO 2	YES . . . . . .1<br>NO 2
(23)		YES . . . . .1<br>NO 2			YES. . .1<br>NO. . . .2	MALE . . . 1<br>FEMALE...2	YES . . .. .1<br>NO 2	YES . . . . . .1<br>NO 2	YES . . . . . .1<br>NO 2	YES . . . . . .1<br>NO 2
(24)		YES . . . . .1<br>NO 2			YES. . .1<br>NO. . . .2	MALE . . . 1<br>FEMALE...2	YES . . .. .1<br>NO 2	YES . . . . . .1<br>NO 2	YES . . . . . .1<br>NO 2	YES . . . . . .1<br>NO 2
(25)		YES . . . . .1<br>NO 2			YES. . .1<br>NO. . . .2	MALE . . . 1<br>FEMALE...2	YES . . .. .1<br>NO 2	YES . . . . . .1<br>NO 2	YES . . . . . .1<br>NO 2	YES . . . . . .1<br>NO 2
(26)		YES . . . . .1<br>NO 2			YES. . .1<br>NO. . . .2	MALE . . . 1<br>FEMALE...2	YES . . .. .1<br>NO 2	YES . . . . . .1<br>NO 2	YES . . . . . .1<br>NO 2	YES . . . . . .1<br>NO 2
(27)		YES . . . . .1<br>NO 2			YES. . .1<br>NO. . . .2	MALE . . . 1<br>FEMALE...2	YES . . .. .1<br>NO 2	YES . . . . . .1<br>NO 2	YES . . . . . .1<br>NO 2	YES . . . . . .1<br>NO 2
(28)		YES . . . . .1<br>NO 2			YES. . .1<br>NO. . . .2	MALE . . . 1<br>FEMALE...2	YES . . .. .1<br>NO 2	YES . . . . . .1<br>NO 2	YES . . . . . .1<br>NO 2	YES . . . . . .1<br>NO 2
(29)		YES . . . . .1<br>NO 2			YES. . .1<br>NO. . . .2	MALE . . . 1<br>FEMALE...2	YES . . .. .1<br>NO 2	YES . . . . . .1<br>NO 2	YES . . . . . .1<br>NO 2	YES . . . . . .1<br>NO 2
(30)		YES . . . . .1<br>NO 2			YES. . .1<br>NO. . . .2	MALE . . . 1<br>FEMALE...2	YES . . .. .1<br>NO 2	YES . . . . . .1<br>NO 2	YES . . . . . .1<br>NO 2	YES . . . . . .1<br>NO 2
(31)		YES . . . . .1<br>NO 2			YES. . .1<br>NO. . . .2	MALE . . . 1<br>FEMALE...2	YES . . .. .1<br>NO 2	YES . . . . . .1<br>NO 2	YES . . . . . .1<br>NO 2	YES . . . . . .1<br>NO 2
(32)		YES . . . . .1<br>NO 2			YES. . .1<br>NO. . . .2	MALE . . . 1<br>FEMALE...2	YES . . .. .1<br>NO 2	YES . . . . . .1<br>NO 2	YES . . . . . .1<br>NO 2	YES . . . . . .1<br>NO 2
(33)		YES . . . . .1<br>NO 2			YES. . .1<br>NO. . . .2	MALE . . . 1<br>FEMALE...2	YES . . .. .1<br>NO 2	YES . . . . . .1<br>NO 2	YES . . . . . .1<br>NO 2	YES . . . . . .1<br>NO 2

CODE: Obstetrician/Gynecologists=01 General surgeons=02 Pediatricians=03 General physicians=04 Theatre nurse=05 Nurse/Midwives=06 Nurses=07 Midwives=08 CHEWs=09 Community health officers (CHO)=10 VCT Counselors=11 Other=96

STAFF										
Q8a. No. of staff	Q8b. NAME	Q8c. Is NAME scheduled to be on duty any time today?	Q8d. Serial number of sampled on-duty staff	Q8e. POSITION CODE	Q8f. Does NAME work full-time?	Q8g. SEX	Does NAME provide service(s)? *Please indicate by checking the box of the services that NAME provides.*			
							Q8h. FAMILY PLANNING	Q8i. MATERNAL HEALTH	Q8j. CHILD HEALTH	Q8k. VCT/STI/HIV SERVICES
(34)		YES . . . . .1<br>NO 2			YES. . .1<br>NO. . . .2	MALE . . . 1<br>FEMALE...2	YES . . . . .1<br>NO 2	YES . . . . . .1<br>NO 2	YES . . . . . .1<br>NO 2	YES . . . . . .1<br>NO 2
(35)		YES . . . . .1<br>NO 2			YES. . .1<br>NO. . . .2	MALE . . . 1<br>FEMALE...2	YES . . . . .1<br>NO 2	YES . . . . . .1<br>NO 2	YES . . . . . .1<br>NO 2	YES . . . . . .1<br>NO 2
(36)		YES . . . . .1<br>NO 2			YES. . .1<br>NO. . . .2	MALE . . . 1<br>FEMALE...2	YES . . . . .1<br>NO 2	YES . . . . . .1<br>NO 2	YES . . . . . .1<br>NO 2	YES . . . . . .1<br>NO 2
(37)		YES . . . . .1<br>NO 2			YES. . .1<br>NO. . . .2	MALE . . . 1<br>FEMALE...2	YES . . . . .1<br>NO 2	YES . . . . . .1<br>NO 2	YES . . . . . .1<br>NO 2	YES . . . . . .1<br>NO 2
(38)		YES . . . . .1<br>NO 2			YES. . .1<br>NO. . . .2	MALE . . . 1<br>FEMALE...2	YES . . . . .1<br>NO 2	YES . . . . . .1<br>NO 2	YES . . . . . .1<br>NO 2	YES . . . . . .1<br>NO 2
(39)		YES . . . . .1<br>NO 2			YES. . .1<br>NO. . . .2	MALE . . . 1<br>FEMALE...2	YES . . . . .1<br>NO 2	YES . . . . . .1<br>NO 2	YES . . . . . .1<br>NO 2	YES . . . . . .1<br>NO 2
(40)		YES . . . . .1<br>NO 2			YES. . .1<br>NO. . . .2	MALE . . . 1<br>FEMALE...2	YES . . . . .1<br>NO 2	YES . . . . . .1<br>NO 2	YES . . . . . .1<br>NO 2	YES . . . . . .1<br>NO 2
(41)		YES . . . . .1<br>NO 2			YES. . .1<br>NO. . . .2	MALE . . . 1<br>FEMALE...2	YES . . . . .1<br>NO 2	YES . . . . . .1<br>NO 2	YES . . . . . .1<br>NO 2	YES . . . . . .1<br>NO 2
(42)		YES . . . . .1<br>NO 2			YES. . .1<br>NO. . . .2	MALE . . . 1<br>FEMALE...2	YES . . . . .1<br>NO 2	YES . . . . . .1<br>NO 2	YES . . . . . .1<br>NO 2	YES . . . . . .1<br>NO 2
(43)		YES . . . . .1<br>NO 2			YES. . .1<br>NO. . . .2	MALE . . . 1<br>FEMALE...2	YES . . . . .1<br>NO 2	YES . . . . . .1<br>NO 2	YES . . . . . .1<br>NO 2	YES . . . . . .1<br>NO 2
(44)		YES . . . . .1<br>NO 2			YES. . .1<br>NO. . . .2	MALE . . . 1<br>FEMALE...2	YES . . . . .1<br>NO 2	YES . . . . . .1<br>NO 2	YES . . . . . .1<br>NO 2	YES . . . . . .1<br>NO 2
(45)		YES . . . . .1<br>NO 2			YES. . .1<br>NO. . . .2	MALE . . . 1<br>FEMALE...2	YES . . . . .1<br>NO 2	YES . . . . . .1<br>NO 2	YES . . . . . .1<br>NO 2	YES . . . . . .1<br>NO 2
(46)		YES . . . . .1<br>NO 2			YES. . .1<br>NO. . . .2	MALE . . . 1<br>FEMALE...2	YES . . . . .1<br>NO 2	YES . . . . . .1<br>NO 2	YES . . . . . .1<br>NO 2	YES . . . . . .1<br>NO 2
(47)		YES . . . . .1<br>NO 2			YES. . .1<br>NO. . . .2	MALE . . . 1<br>FEMALE...2	YES . . . . .1<br>NO 2	YES . . . . . .1<br>NO 2	YES . . . . . .1<br>NO 2	YES . . . . . .1<br>NO 2
(48)		YES . . . . .1<br>NO 2			YES. . .1<br>NO. . . .2	MALE . . . 1<br>FEMALE...2	YES . . . . .1<br>NO 2	YES . . . . . .1<br>NO 2	YES . . . . . .1<br>NO 2	YES . . . . . .1<br>NO 2
(49)		YES . . . . .1<br>NO 2			YES. . .1<br>NO. . . .2	MALE . . . 1<br>FEMALE...2	YES . . . . .1<br>NO 2	YES . . . . . .1<br>NO 2	YES . . . . . .1<br>NO 2	YES . . . . . .1<br>NO 2
(50)		YES . . . . .1<br>NO 2			YES. . .1<br>NO. . . .2	MALE . . . 1<br>FEMALE...2	YES . . . . .1<br>NO 2	YES . . . . . .1<br>NO 2	YES . . . . . .1<br>NO 2	YES . . . . . .1<br>NO 2
(51)		YES . . . . .1<br>NO 2			YES. . .1<br>NO. . . .2	MALE . . . 1<br>FEMALE...2	YES . . . . .1<br>NO 2	YES . . . . . .1<br>NO 2	YES . . . . . .1<br>NO 2	YES . . . . . .1<br>NO 2
(52)		YES . . . . .1<br>NO 2			YES. . .1<br>NO. . . .2	MALE . . . 1<br>FEMALE...2	YES . . . . .1<br>NO 2	YES . . . . . .1<br>NO 2	YES . . . . . .1<br>NO 2	YES . . . . . .1<br>NO 2

CODE: Obstetrician/Gynecologists=01 Pediatricians=03 Theatre nurse=05 Nurses=07 CHEWs=09 VCT Counselors=11
General surgeons=02 General physicians=04 Nurse/Midwives=06 Midwives=08 Community health officers (CHO)=10 Other=96

STAFF										
Q8a. No. of staff	Q8b. NAME	Q8c. Is NAME scheduled to be on duty any time today?	Q8d. Serial number of sampled on-duty staff	Q8e. POSITION CODE	Q8f. Does NAME work full-time?	Q8g. SEX	Does NAME provide service(s)? *Please indicate by checking the box of the services that NAME provides.*			
							Q8h. FAMILY PLANNING	Q8i. MATERNAL HEALTH	Q8j. CHILD HEALTH	Q8k. VCT/STI/HIV SERVICES
(53)		YES . . . . .1<br>NO 2			YES. . .1<br>NO. . . .2	MALE . . . 1<br>FEMALE...2	YES . . . . .1<br>NO 2	YES . . . . . .1<br>NO 2	YES . . . . . .1<br>NO 2	YES . . . . . .1<br>NO 2
(54)		YES . . . . .1<br>NO 2			YES. . .1<br>NO. . . .2	MALE . . . 1<br>FEMALE...2	YES . . . . .1<br>NO 2	YES . . . . . .1<br>NO 2	YES . . . . . .1<br>NO 2	YES . . . . . .1<br>NO 2
(55)		YES . . . . .1<br>NO 2			YES. . .1<br>NO. . . .2	MALE . . . 1<br>FEMALE...2	YES . . . . .1<br>NO 2	YES . . . . . .1<br>NO 2	YES . . . . . .1<br>NO 2	YES . . . . . .1<br>NO 2
(56)		YES . . . . .1<br>NO 2			YES. . .1<br>NO. . . .2	MALE . . . 1<br>FEMALE...2	YES . . . . .1<br>NO 2	YES . . . . . .1<br>NO 2	YES . . . . . .1<br>NO 2	YES . . . . . .1<br>NO 2
(57)		YES . . . . .1<br>NO 2			YES. . .1<br>NO. . . .2	MALE . . . 1<br>FEMALE...2	YES . . . . .1<br>NO 2	YES . . . . . .1<br>NO 2	YES . . . . . .1<br>NO 2	YES . . . . . .1<br>NO 2
(58)		YES . . . . .1<br>NO 2			YES. . .1<br>NO. . . .2	MALE . . . 1<br>FEMALE...2	YES . . . . .1<br>NO 2	YES . . . . . .1<br>NO 2	YES . . . . . .1<br>NO 2	YES . . . . . .1<br>NO 2
(59)		YES . . . . .1<br>NO 2			YES. . .1<br>NO. . . .2	MALE . . . 1<br>FEMALE...2	YES . . . . .1<br>NO 2	YES . . . . . .1<br>NO 2	YES . . . . . .1<br>NO 2	YES . . . . . .1<br>NO 2
(60)		YES . . . . .1<br>NO 2			YES. . .1<br>NO. . . .2	MALE . . . 1<br>FEMALE...2	YES . . . . .1<br>NO 2	YES . . . . . .1<br>NO 2	YES . . . . . .1<br>NO 2	YES . . . . . .1<br>NO 2
(61)		YES . . . . .1<br>NO 2			YES. . .1<br>NO. . . .2	MALE . . . 1<br>FEMALE...2	YES . . . . .1<br>NO 2	YES . . . . . .1<br>NO 2	YES . . . . . .1<br>NO 2	YES . . . . . .1<br>NO 2
(62)		YES . . . . .1<br>NO 2			YES. . .1<br>NO. . . .2	MALE . . . 1<br>FEMALE...2	YES . . . . .1<br>NO 2	YES . . . . . .1<br>NO 2	YES . . . . . .1<br>NO 2	YES . . . . . .1<br>NO 2
(63)		YES . . . . .1<br>NO 2			YES. . .1<br>NO. . . .2	MALE . . . 1<br>FEMALE...2	YES . . . . .1<br>NO 2	YES . . . . . .1<br>NO 2	YES . . . . . .1<br>NO 2	YES . . . . . .1<br>NO 2
(64)		YES . . . . .1<br>NO 2			YES. . .1<br>NO. . . .2	MALE . . . 1<br>FEMALE...2	YES . . . . .1<br>NO 2	YES . . . . . .1<br>NO 2	YES . . . . . .1<br>NO 2	YES . . . . . .1<br>NO 2
(65)		YES . . . . .1<br>NO 2			YES. . .1<br>NO. . . .2	MALE . . . 1<br>FEMALE...2	YES . . . . .1<br>NO 2	YES . . . . . .1<br>NO 2	YES . . . . . .1<br>NO 2	YES . . . . . .1<br>NO 2
(66)		YES . . . . .1<br>NO 2			YES. . .1<br>NO. . . .2	MALE . . . 1<br>FEMALE...2	YES . . . . .1<br>NO 2	YES . . . . . .1<br>NO 2	YES . . . . . .1<br>NO 2	YES . . . . . .1<br>NO 2
(67)		YES . . . . .1<br>NO 2			YES. . .1<br>NO. . . .2	MALE . . . 1<br>FEMALE...2	YES . . . . .1<br>NO 2	YES . . . . . .1<br>NO 2	YES . . . . . .1<br>NO 2	YES . . . . . .1<br>NO 2
(68)		YES . . . . .1<br>NO 2			YES. . .1<br>NO. . . .2	MALE . . . 1<br>FEMALE...2	YES . . . . .1<br>NO 2	YES . . . . . .1<br>NO 2	YES . . . . . .1<br>NO 2	YES . . . . . .1<br>NO 2
(69)		YES . . . . .1<br>NO 2			YES. . .1<br>NO. . . .2	MALE . . . 1<br>FEMALE...2	YES . . . . .1<br>NO 2	YES . . . . . .1<br>NO 2	YES . . . . . .1<br>NO 2	YES . . . . . .1<br>NO 2
(70)		YES . . . . .1<br>NO 2			YES. . .1<br>NO. . . .2	MALE . . . 1<br>FEMALE...2	YES . . . . .1<br>NO 2	YES . . . . . .1<br>NO 2	YES . . . . . .1<br>NO 2	YES . . . . . .1<br>NO 2
(71)		YES . . . . .1<br>NO 2			YES. . .1<br>NO. . . .2	MALE . . . 1<br>FEMALE...2	YES . . . . .1<br>NO 2	YES . . . . . .1<br>NO 2	YES . . . . . .1<br>NO 2	YES . . . . . .1<br>NO 2

CODE: Obstetrician/Gynecologists=01 General surgeons=02 Pediatricians=03 General physicians=04 Theatre nurse=05 Nurse/Midwives=06 Nurses=07 Midwives=08 CHEWs=09 Community health officers (CHO)=10 VCT Counselors=11 Other=96

STAFF										
Q8a. No. of staff	Q8b. NAME	Q8c. Is NAME scheduled to be on duty any time today?	Q8d. Serial number of sampled on-duty staff	Q8e. POSITION CODE	Q8f. Does NAME work full-time?	Q8g. SEX	Does NAME provide service(s)? *Please indicate by checking the box of the services that NAME provides.*			
							Q8h. FAMILY PLANNING	Q8i. MATERNAL HEALTH	Q8j. CHILD HEALTH	Q8k. VCT/STI/HIV SERVICES
(72)		YES . . . . .1<br>NO 2			YES. . .1<br>NO. . . .2	MALE . . . 1<br>FEMALE...2	YES . . . . .1<br>NO 2	YES . . . . . .1<br>NO 2	YES . . . . . .1<br>NO 2	YES . . . . . .1<br>NO 2
(73)		YES . . . . .1<br>NO 2			YES. . .1<br>NO. . . .2	MALE . . . 1<br>FEMALE...2	YES . . . . .1<br>NO 2	YES . . . . . .1<br>NO 2	YES . . . . . .1<br>NO 2	YES . . . . . .1<br>NO 2
(74)		YES . . . . .1<br>NO 2			YES. . .1<br>NO. . . .2	MALE . . . 1<br>FEMALE...2	YES . . . . .1<br>NO 2	YES . . . . . .1<br>NO 2	YES . . . . . .1<br>NO 2	YES . . . . . .1<br>NO 2
(75)		YES . . . . .1<br>NO 2			YES. . .1<br>NO. . . .2	MALE . . . 1<br>FEMALE...2	YES . . . . .1<br>NO 2	YES . . . . . .1<br>NO 2	YES . . . . . .1<br>NO 2	YES . . . . . .1<br>NO 2
(76)		YES . . . . .1<br>NO 2			YES. . .1<br>NO. . . .2	MALE . . . 1<br>FEMALE...2	YES . . . . .1<br>NO 2	YES . . . . . .1<br>NO 2	YES . . . . . .1<br>NO 2	YES . . . . . .1<br>NO 2
(77)		YES . . . . .1<br>NO 2			YES. . .1<br>NO. . . .2	MALE . . . 1<br>FEMALE...2	YES . . . . .1<br>NO 2	YES . . . . . .1<br>NO 2	YES . . . . . .1<br>NO 2	YES . . . . . .1<br>NO 2
(78)		YES . . . . .1<br>NO 2			YES. . .1<br>NO. . . .2	MALE . . . 1<br>FEMALE...2	YES . . . . .1<br>NO 2	YES . . . . . .1<br>NO 2	YES . . . . . .1<br>NO 2	YES . . . . . .1<br>NO 2
(79)		YES . . . . .1<br>NO 2			YES. . .1<br>NO. . . .2	MALE . . . 1<br>FEMALE...2	YES . . . . .1<br>NO 2	YES . . . . . .1<br>NO 2	YES . . . . . .1<br>NO 2	YES . . . . . .1<br>NO 2
(80)		YES . . . . .1<br>NO 2			YES. . .1<br>NO. . . .2	MALE . . . 1<br>FEMALE...2	YES . . . . .1<br>NO 2	YES . . . . . .1<br>NO 2	YES . . . . . .1<br>NO 2	YES . . . . . .1<br>NO 2
(81)		YES . . . . .1<br>NO 2			YES. . .1<br>NO. . . .2	MALE . . . 1<br>FEMALE...2	YES . . . . .1<br>NO 2	YES . . . . . .1<br>NO 2	YES . . . . . .1<br>NO 2	YES . . . . . .1<br>NO 2
(82)		YES . . . . .1<br>NO 2			YES. . .1<br>NO. . . .2	MALE . . . 1<br>FEMALE...2	YES . . . . .1<br>NO 2	YES . . . . . .1<br>NO 2	YES . . . . . .1<br>NO 2	YES . . . . . .1<br>NO 2
(83)		YES . . . . .1<br>NO 2			YES. . .1<br>NO. . . .2	MALE . . . 1<br>FEMALE...2	YES . . . . .1<br>NO 2	YES . . . . . .1<br>NO 2	YES . . . . . .1<br>NO 2	YES . . . . . .1<br>NO 2
(84)		YES . . . . .1<br>NO 2			YES. . .1<br>NO. . . .2	MALE . . . 1<br>FEMALE...2	YES . . . . .1<br>NO 2	YES . . . . . .1<br>NO 2	YES . . . . . .1<br>NO 2	YES . . . . . .1<br>NO 2
(85)		YES . . . . .1<br>NO 2			YES. . .1<br>NO. . . .2	MALE . . . 1<br>FEMALE...2	YES . . . . .1<br>NO 2	YES . . . . . .1<br>NO 2	YES . . . . . .1<br>NO 2	YES . . . . . .1<br>NO 2
(86)		YES . . . . .1<br>NO 2			YES. . .1<br>NO. . . .2	MALE . . . 1<br>FEMALE...2	YES . . . . .1<br>NO 2	YES . . . . . .1<br>NO 2	YES . . . . . .1<br>NO 2	YES . . . . . .1<br>NO 2
(87)		YES . . . . .1<br>NO 2			YES. . .1<br>NO. . . .2	MALE . . . 1<br>FEMALE...2	YES . . . . .1<br>NO 2	YES . . . . . .1<br>NO 2	YES . . . . . .1<br>NO 2	YES . . . . . .1<br>NO 2
(88)		YES . . . . .1<br>NO 2			YES. . .1<br>NO. . . .2	MALE . . . 1<br>FEMALE...2	YES . . . . .1<br>NO 2	YES . . . . . .1<br>NO 2	YES . . . . . .1<br>NO 2	YES . . . . . .1<br>NO 2

CODE: Obstetrician/Gynecologists=01 General surgeons=02 Pediatricians=03 General physicians=04 Theatre nurse=05 Nurse/Midwives=06 Nurses=07 Midwives=08 CHEWs=09 Community health officers (CHO)=10 VCT Counselors=11 Other=96

CHECK THE BOX IF ANOTHER FORM IS USED: ☐ TOTAL NUMBER OF FORMS: ☐ FORM NUMBER: ☐

GENERAL MCH AND FP					
SERVICE	Q9a. Does this facility provide the following Maternal and Child Health SERVICES?	Q9b. How many days per week is SERVICE available?	Q9c. What year was SERVICE first offered at this facility?	Q9d. How many clients received this service here in the past 3 months? ASK TO SEE MEDICAL RECORD SYSTEM, IF POSSIBLE. OTHERWISE, ASK RESPONDENT TO RECALL.	Q9e.WHAT WAS THE SOURCE OF THIS INFORMATION?
(1) Maternity care/delivery services	Yes . . . . 1<br>No . . . . . 2→**(2)**	Days . . ☐	☐☐☐☐<br>**Don't know = 9998**	☐☐☐☐☐<br>**NOT AVAILABLE.....99993**	OBSERVED RECORD................1<br>PROVIDER ESTIMATE..............2
(2) Counseling on initiating breast-feeding (after delivery)	Yes . . . . 1<br>No 2				
(3) Emergency care for prolonged or obstructed labor (cesarean section, blood transfusion)	Yes . . . . 1<br>No . . . . . 2→**(4)**	Days . . ☐	☐☐☐☐<br>**Don't know = 9998**	☐☐☐☐☐<br>**NOT AVAILABLE.....99993**	OBSERVED RECORD................1<br>PROVIDER ESTIMATE..............2
(4) Consultation for infertility	Yes . . . . 1<br>No . . . . . 2→**(5)**	Days . . ☐	☐☐☐☐<br>**Don't know = 9998**	☐☐☐☐☐<br>**NOT AVAILABLE.....99993**	OBSERVED RECORD................1<br>PROVIDER ESTIMATE..............2
(5) Post-abortion care	Yes . . . . 1<br>No . . . . . 2→**(6)**	Days . . ☐	☐☐☐☐<br>**Don't know = 9998**	☐☐☐☐☐<br>**NOT AVAILABLE.....99993**	OBSERVED RECORD................1<br>PROVIDER ESTIMATE..............2
(6) Ante-natal care	Yes . . . . 1<br>No . . . . 2→**(12)**	Days . . ☐	☐☐☐☐<br>**Don't know = 9998**	☐☐☐☐☐<br>**NOT AVAILABLE.....99993**	OBSERVED RECORD................1<br>PROVIDER ESTIMATE..............2
(7) Complete Regimen of Tetanus Toxoid immunization during pregnancy (5 doses)	Yes . . . . 1<br>No 2				
(8) Syphilis screening during pregnancy	Yes . . . . 1<br>No 2				
(9) Iron supplementation during pregnancy	Yes . . . . 1<br>No 2				
(10) Intermittent preventive treatment for malaria (IPT)	Yes . . . . 1<br>No 2				
(11) Nutrition counseling during pregnancy	Yes . . . . 1<br>No 2				

SERVICE	Q9a. Does this facility provide the following Maternal and Child Health SERVICE?	Q9b. How many days per week is SERVICE available?	Q9c. What year was SERVICE first offered at this facility?	Q9d. How many clients received this service here in the past 3 months? ASK TO SEE MEDICAL RECORD SYSTEM, IF POSSIBLE. OTHERWISE, ASK RESPONDENT TO RECALL.	Q9e.WHAT WAS THE SOURCE OF THIS INFORMATION?
(12) Post natal care	Yes . . . . 1<br>No . . . . 2→**(14)**	Days . . .	**Don't know = 9998**	**NOT AVAILABLE.....99993**	OBSERVED RECORD................1<br>PROVIDER ESTIMATE..............2
(13) Vitamin A supplementation after pregnancy	Yes . . . . 1<br>No 2				
(14) Child immunization	Yes . . . . 1<br>No . . . . 2→**(15)**	Days . . .	**Don't know = 9998**	**NOT AVAILABLE.....99993**	OBSERVED RECORD................1<br>PROVIDER ESTIMATE..............2
(15) Child growth monitoring	Yes . . . . 1<br>No . . . . 2→**(16)**	Days . . .	**Don't know = 9998**	**NOT AVAILABLE.....99993**	OBSERVED RECORD................1<br>PROVIDER ESTIMATE..............2
(16) Child respiratory disease	Yes . . . . 1<br>No . . . . 2→**(17)**	Days . . .	**Don't know = 9998**	**NOT AVAILABLE.....99993**	OBSERVED RECORD................1<br>PROVIDER ESTIMATE..............2
(17) Oral rehydration therapy services	Yes . . . . 1<br>No . . . . 2→**(18)**	Days . . .	**Don't know = 9998**	**NOT AVAILABLE.....99993**	OBSERVED RECORD................1<br>PROVIDER ESTIMATE..............2
(18) Detection and treatment of sexually transmitted infections (STIs)	Yes . . . . 1<br>No . . . . 2→**(19)**	Days . . .	**Don't know = 9998**	**NOT AVAILABLE.....99993**	OBSERVED RECORD................1<br>PROVIDER ESTIMATE..............2
(19) Voluntary counseling and testing (VCT)	Yes . . . . 1<br>No . . . . 2→**(20)**	Days . . .	**Don't know = 9998**	**NOT AVAILABLE.....99993**	OBSERVED RECORD................1<br>PROVIDER ESTIMATE..............2
(20) PMTCT	Yes . . . . 1<br>No . . . . 2→**(21)**	Days . . .	**Don't know = 9998**	**NOT AVAILABLE.....99993**	OBSERVED RECORD................1<br>PROVIDER ESTIMATE..............2
(21) HIV/AIDS Management	Yes . . . . 1<br>No . . . . 2→**(22)**	Days . . .	**Don't know = 9998**	**NOT AVAILABLE.....99993**	OBSERVED RECORD................1<br>PROVIDER ESTIMATE..............2
(22) Family planning counseling & services	Yes . . . . 1<br>No . . 2→**(Q11)**	Days . . .	**Don't know = 9998**	**NOT AVAILABLE.....99993**	OBSERVED RECORD................1<br>PROVIDER ESTIMATE..............2

Q10.	Approximately, what percentage of the clients who received family planning counseling and services in the past 3 months were between the ages of 15 and 19 years old?	[ ][ ][ ]<br>NONE…………...000<br>DON'T KNOW….998	
Q11.	Does this facility ever refer clients to other health care facilities?	Yes . . . . 1<br>No . . . . 2 →	Q13
Q12.	For which services are these referrals?<br><br>[**MULTIPLE RESPONSE POSSIBLE**]	FAMILY PLANNING……………………………………A<br>IMMUNIZATION…………………………………………B<br>ANTENATAL CARE……………………………………..C<br>DELIVERY CARE…………………………………………D<br>EMERGENCY DELIVERY CARE (C-SECTION)………..E<br>POSTNATAL CARE……………………………………F<br>DISEASE PREVENTION………………………………..G<br>TREATMENT OF ADULT……………………………….H<br>TREATMENT FOR CHILD……………………………I<br>GROWTH MONITORING OF CHILD…………………..J<br>HEALTH CHECK-UP . . . . ……………… …K<br>VCT……………………………………………………L<br>HIV/AIDS MANAGEMENT………………………………M<br>PMTCT………………………………………………N<br>OTHER________________________X<br>(SPECIFY)	
Now I would like to ask you some questions about other health services.			
Q13.	CHECK Q9A. IF YES TO (14) CHILD IMMUNIZATION, (15) CHILD GROWTH MONITORING, OR (16) CHILD RESPIRATORY DISEASE [ ] ↓	IF NO TO ALL CHILD SERVICES (14-16) [ ] →	Q17
Q14.	What is the normal practice for this facility if a woman who has come for a ***child health visit*** is interested in ***receiving information*** on FP? Is she able to receive this information on the day of her visit, or is she asked to come back on a different day?<br><br>CIRCLE ONE.	Always receive on same day…………………………01<br>Sometimes receive on same day……………………02<br>Make appointment to come back a different day….03<br>No appointment made, always told to come back different day……………………………………………04<br>Given referral to another facility………………………05<br>Given no information or referral………………………06<br>Do not offer family planning services………………07 →<br>Other____________________________________96<br>(SPECIFY)	Q17
Q15.	If a woman who has come for a ***child health visit*** is interested in ***receiving a hormonal method of FP***, what is the normal practice for this facility?<br><br>CIRCLE ONE.	Always receive on same day…………………………01<br>Sometimes receive on same day……………………02<br>Make appointment to come back a different day….03<br>No appointment made, always told to come back different day……………………………………………04<br>Given referral to another facility………………………05<br>Given no information or referral………………………06<br>Other____________________________________96<br>(SPECIFY)	
Q16.	If a woman who has come for a ***child health visit*** is interested in ***getting sterilized***, what is the normal practice for this facility?<br><br>CIRCLE ONE.	Procedure can happen on same day…………………01<br>Sometimes the procedure can happen on same day……………………………………………………02<br>Make appointment to come back a different day……03<br>No appointment made, always told to come back different day……………………………………………04<br>Given referral to another facility………………………05<br>Given no information or referral………………………06<br>Other____________________________________96<br>(SPECIFY)	
Q17.	CHECK Q9A: IF YES TO (12) POST NATAL CARE [ ] ↓	IF NO TO (12) POST NATAL CARE [ ] →	Q21
Q18.	What is the normal practice for this facility if a woman who has come for a ***postnatal care visit*** is interested in ***receiving information*** on FP? Is she able to receive this information on the day of her visit, or is she asked to come back on a different day?<br><br>CIRCLE ONE.	Always receive on same day…………………………01<br>Sometimes receive on same day……………………02<br>Make appointment to come back a different day….03<br>No appointment made, always told to come back different day……………………………………………04<br>Given referral to another facility………………………05<br>Given no information or referral………………………06<br>Do not offer family planning services………………07 →<br>Other____________________________________96<br>(SPECIFY)	Q21

Q19.	If a woman who has come for a ***postnatal care visit*** is interested in ***receiving a hormonal method of FP***, what is the normal practice for this facility?<br><br>CIRCLE ONE.	Always receive on same day……………………….01<br>Sometimes receive on same day……………………02<br>Make appointment to come back a different day….03<br>No appointment made, always told to come back different day……………………………………….04<br>Given referral to another facility………………………05<br>Given no information or referral………………………06<br>Other______________________________96<br>(SPECIFY)	
Q20.	If a woman who has come for a ***postnatal care visit*** is interested in ***getting sterilized***, what is the normal practice for this facility?<br><br>CIRCLE ONE.	Procedure can happen on same day…………………01<br>Sometimes the procedure can happen on same day……………………………………………………02<br>Make appointment to come back a different day….03<br>No appointment made, always told to come back different day……………………………………….04<br>Given referral to another facility………………………05<br>Given no information or referral………………………06<br>Other______________________________96<br>(SPECIFY)	
Q21.	CHECK Q9A. IF YES TO (5) POST-ABORTION CARE ☐ ↓	IF NO TO (5) POST-ABORTION CARE ☐ →	**Q25**
Q22.	What is the normal practice for this facility if a woman who has come for ***post-abortion care*** is interested in ***receiving information*** on FP? Is she able to receive this information on the day of her visit, or is she asked to come back on a different day?<br><br>CIRCLE ONE.	Always receive on same day……………………….01<br>Sometimes receive on same day……………………02<br>Make appointment to come back a different day….03<br>No appointment made, always told to come back different day……………………………………….04<br>Given referral to another facility………………………05<br>Given no information or referral………………………06<br>Do not offer family planning services……………….07 →<br>Other (specify)________________________96	 <br><br><br><br><br><br>**Q25**
Q23.	If a woman who has come for ***post-abortion care*** is interested in ***receiving a hormonal method of FP***, what is the normal practice for this facility?<br><br>CIRCLE ONE.	Always receive on same day……………………….01<br>Sometimes receive on same day……………………02<br>Make appointment to come back a different day….03<br>No appointment made, always told to come back different day……………………………………….04<br>Given referral to another facility………………………05<br>Given no information or referral………………………06<br>Other______________________________96<br>(SPECIFY)	
Q24.	If a woman who has come for ***post-abortion care*** is interested in ***getting sterilized***, what is the normal practice for this facility?<br><br>CIRCLE ONE.	Procedure can happen on same day…………………01<br>Sometimes the procedure can happen on same day……………………………………………………02<br>Make appointment to come back a different day….03<br>No appointment made, always told to come back different day……………………………………….04<br>Given referral to another facility………………………05<br>Given no information or referral………………………06<br>Other______________________________96	
Q25.	CHECK Q9A: IF YES TO ANY (18) Detection and treatment of STIs, (19) VCT, (20) PMTCT, OR (21) HIV/AIDS management ☐ ↓	IF NO TO ALL (18-21) ☐ →	**Q29**
Q26.	What is the normal practice for this facility if a woman or man who has come for ***STI treatment, VCT, PMTCT, or HIV/AIDS care*** is interested in ***receiving information*** on FP, is she/he able to receive this information on the day of her/his visit, or is she/he asked to come back on a different day?<br><br>CIRCLE ONE.	Always receive on same day……………………….01<br>Sometimes receive on same day……………………02<br>Make appointment to come back a different day….03<br>No appointment made, always told to come back different day……………………………………….04<br>Given referral to another facility………………………05<br>Given no information or referral………………………06<br>Do not offer family planning services……………….07 →<br>Other______________________________96<br>(SPECIFY)	 <br><br><br><br><br><br>**Q29**

Q27.	If a woman who has come for ***STI treatment, VCT, PMTCT, or HIV/AIDS care*** is interested in ***receiving a hormonal method***, what is the normal practice for this facility?<br><br>CIRCLE ONE.	Always receive on same day……………………………01<br>Sometimes receive on same day……………………02<br>Make appointment to come back a different day….03<br>No appointment made, always told to come back different day…………………………………………04<br>Given referral to another facility………………………05<br>Given no information or referral………………………06<br>Other_________________________________96<br>(SPECIFY)	
Q28.	If a woman who has come for ***STI treatment, VCT, PMTCT, or HIV/AIDS care*** is interested in ***getting sterilized***, what is the normal practice for this facility?<br><br>CIRCLE ONE.	Procedure can happen on same day…………………01<br>Sometimes the procedure can happen on same day………………………………………………….……02<br>Make appointment to come back a different day….03<br>No appointment made, always told to come back different day…………………………………………04<br>Given referral to another facility………………………05<br>Given no information or referral………………………06<br>Other_________________________________96	
Q29.	[SEE Q9A (22) FAMILY PLANNING COUNSELING & SERVICES] → **IF FP <u>IS OFFERED</u>**,<br><br>YES, ☐ ↓	[SEE Q9A (22) FAMILY PLANNING COUNSELING & SERVICES] → **IF FP IS <u>NOT OFFERED</u>**,<br><br>NO, ☐ ↓<br><br>Would FP counseling and services be appropriate to include into the existing services offered?<br><br>Yes . . . . 1<br>No . . . . 2<br>Don't know 8	**ALL SKIP TO Q67**

ASK IF THE FOLLOWING CONTRACEPTIVES ARE PROVIDED IN THIS FACILITY. FOR EACH ITEM, CIRCLE THE APPROPRIATE CODE.					
METHOD	Q30a. Does this facility provide the following FP methods/ services?	Q30b. How many days per week is the method provided?	Q30c. What year was METHOD first offered at this facility?<br><br>**Don't know = 9998**	Q30d. Are there requirements for partner's consent to receive the following METHOD?	Q30e. How many staff do you have that can provide METHOD?
(01) Combined oral pill	YES.....1<br>NO.......2 → **(02)**	Days… ☐	☐☐☐☐	YES . . . . . .1<br>NO 2	
(02) Progestin only pill	YES.....1<br>NO.......2 → **(03)**	Days… ☐	☐☐☐☐	YES . . . . . .1<br>NO 2	
(03) Emergency contraceptive	YES.....1<br>NO.......2 → **(04)**	Days… ☐	☐☐☐☐	YES . . . . . .1<br>NO 2	
(04) Male condom	YES.....1<br>NO.......2 → **(05)**	Days… ☐	☐☐☐☐	YES . . . . . .1<br>NO 2	
(05) Female condom	YES.....1<br>NO.......2 → **(06)**	Days… ☐	☐☐☐☐	YES . . . . . .1<br>NO 2	
(06) Injectables	YES.....1<br>NO.......2 → **(07)**	Days… ☐	☐☐☐☐	YES . . . . . .1<br>NO 2	
(07) Implants (Jadelle/ Implanon)	YES.....1<br>NO.......2 → **(08)**	Days… ☐	☐☐☐☐	YES . . . . . .1<br>NO 2	**[___I___I___]**
(08) IUD	YES.....1<br>NO.......2 → **(09)**	Days… ☐	☐☐☐☐	YES . . . . . .1<br>NO 2	**[___I___I___]**
(09) Female sterilization/ tubal ligation	YES.....1<br>NO.......2 → **(10)**	Days… ☐	☐☐☐☐	YES . . . . . .1<br>NO 2	**[___I___I___]**
(10) Male sterilization	YES.....1<br>NO.......2 → **(11)**	Days… ☐	☐☐☐☐	YES . . . . . .1<br>NO 2	**[___I___I___]**
(11) Other (specify) ______________	YES.....1<br>NO.......2 → **(Q31a)**	Days… ☐	☐☐☐☐	YES . . . . . .1<br>NO 2	

Now I would like to ask you about your specific stocks of different family planning methods/products.
ONLY ASK ABOUT THOSE METHODS THAT ARE AVAILABLE FROM Q30a.

CONTRACEPTIVE	Q31a. Where does your stock of CONTRACEPTIVE come from? CHOOSE ALL.	Q31b. When was the last time that you received a routine supply of CONTRACEPTIVE either that you ordered, or that is part of your routine supply system? READ LIST.	Q31c. Does this facility determine the quantity of each CONTRACEPTIVE that it needs and order that, or is the quantity that you receive determined elsewhere? READ LIST.	Q31d. Do you always receive a standard fixed quantity of CONTRACEPTIVE or does the quantity you receive vary according to recent need or activity level? READ LIST.	Q31e. **CHECK Q31C. IFQ31C IS "NO", SKIP TO Q31G** When you order CONTRACEPTIVE, how much do you order? READ LIST.	Q31f. When do you decide to order CONTRACEPTIVE? READ LIST.	Q31g. On average, how long does it take to receive your supplies after you have placed an order? READ LIST.	Q31h. Is METHOD usually delivered or must you go get them?
(01) Combination oral contraceptives (estrogen and progestin)	Govt…………… A<br>Intl NGO ……… B<br>Local NGO…….C<br>Pharmacy wholesaler/ dealer/ distributor………D<br>Other________X<br>(Specify)<br>Don't know….….Z	< 4 wks ago…......1<br>Between 4-12 wks………………2<br>> 12 wks ago……3<br>No routine supply system…………..4<br>Don't know………8	Determines own need….1 →**(Q31e)**<br>Determined Elsewhere……..2<br>Both.……………...3<br>Don't know ………..8 →**(Q31g)**	Quantity based on activity level…..1<br>Standard fixed supply…………..2<br>Don't know……...8	Order to maintain stock……………..1<br>Order same amount…………2<br>Order based on consumption…..3<br>Other__________.6<br>(Specify)<br>Don't know…..…...8	Fall below predeter-mined level……….1<br>Fixed time – Every [__|__|__] days…….2<br>Order when needed..3<br>Other…………………6<br>Don't know………..…8	One week or less…....1<br>Between 2-4 weeks….2<br>Between 5-8 weeks….3<br>More than 8 weeks…..4<br>Other _____________6<br>(Specify)<br>Don't know . …………8	Delivered …….1<br>Pick them up…2<br>Both …………..3
(02) Progestin-only oral contraceptives	Govt…………… A<br>Intl NGO ……… B<br>Local NGO…….C<br>Pharmacy wholesaler/ dealer/ distributor………D<br>Other________X<br>(Specify)<br>Don't know….….Z	< 4 wks ago…......1<br>Between 4-12 wks………………2<br>> 12 wks ago……3<br>No routine supply system…………..4<br>Don't know………8	Determines own need….1 →**(Q31e)**<br>Determined Elsewhere……..2<br>Both.……………...3<br>Don't know ………..8 →**(Q31g)**	Quantity based on activity level…..1<br>Standard fixed supply…………..2<br>Don't know……...8	Order to maintain stock……………..1<br>Order same amount…………2<br>Order based on consumption…..3<br>Other__________.6<br>(Specify)<br>Don't know…..…...8	Fall below predeter-mined level……….1<br>Fixed time – Every [__|__|__] days…….2<br>Order when needed..3<br>Other…………………6<br>Don't know………..…8	One week or less…....1<br>Between 2-4 weeks….2<br>Between 5-8 weeks….3<br>More than 8 weeks…..4<br>Other _____________6<br>(Specify)<br>Don't know . …………8	Delivered …….1<br>Pick them up…2<br>Both …………..3
(03) Emergency contraceptives	Govt…………… A<br>Intl NGO ……… B<br>Local NGO…….C<br>Pharmacy wholesaler/ dealer/ distributor………D<br>Other________X<br>(Specify)<br>Don't know….….Z	< 4 wks ago…......1<br>Between 4-12 wks………………2<br>> 12 wks ago……3<br>No routine supply system…………..4<br>Don't know………8	Determines own need….1 →**(Q31e)**<br>Determined Elsewhere……..2<br>Both.……………...3<br>Don't know ………..8 →**(Q31g)**	Quantity based on activity level…..1<br>Standard fixed supply…………..2<br>Don't know……...8	Order to maintain stock……………..1<br>Order same amount…………2<br>Order based on consumption…..3<br>Other__________.6<br>(Specify)<br>Don't know…..…...8	Fall below predeter-mined level……….1<br>Fixed time – Every [__|__|__] days…….2<br>Order when needed..3<br>Other…………………6<br>Don't know………..…8	One week or less…....1<br>Between 2-4 weeks….2<br>Between 5-8 weeks….3<br>More than 8 weeks…..4<br>Other _____________6<br>(Specify)<br>Don't know . …………8	Delivered …….1<br>Pick them up…2<br>Both …………..3
(04) Male condoms	Govt…………… A<br>Intl NGO ……… B<br>Local NGO…….C<br>Pharmacy wholesaler/ dealer/ distributor………D<br>Other________X<br>(Specify)<br>Don't know….….Z	< 4 wks ago…......1<br>Between 4-12 wks………………2<br>> 12 wks ago……3<br>No routine supply system…………..4<br>Don't know………8	Determines own need….1 →**(Q31e)**<br>Determined Elsewhere……..2<br>Both.……………...3<br>Don't know ………..8 →**(Q31g)**	Quantity based on activity level…..1<br>Standard fixed supply…………..2<br>Don't know……...8	Order to maintain stock……………..1<br>Order same amount…………2<br>Order based on consumption…..3<br>Other__________.6<br>(Specify)<br>Don't know…..…...8	Fall below predeter-mined level……….1<br>Fixed time – Every [__|__|__] days…….2<br>Order when needed..3<br>Other…………………6<br>Don't know………..…8	One week or less…....1<br>Between 2-4 weeks….2<br>Between 5-8 weeks….3<br>More than 8 weeks…..4<br>Other _____________6<br>(Specify)<br>Don't know . …………8	Delivered …….1<br>Pick them up…2<br>Both …………..3

CONTRACEPTIVE	Q31a. Where does your stock of CONTRACEPTIVE come from? CHOOSE ALL.	Q31b. When was the last time that you received a routine supply of CONTRACEPTIVE either that you ordered, or that is part of your routine supply system? READ LIST.	Q31c. Does this facility determine the quantity of each CONTRACEPTIVE that it needs and order that, or is the quantity that you receive determined elsewhere? READ LIST.	Q31d. Do you always receive a standard fixed quantity of CONTRACEPTIVE or does the quantity you receive vary according to recent need or activity level? READ LIST.	Q31e. **CHECK Q31C. IFQ31C IS "NO", SKIP TO Q31G** When you order CONTRACEPTIVE, how much do you order?<br><br>READ LIST.	Q31f. When do you decide to order CONTRACEPTIVE?<br><br>READ LIST.	Q31g. On average, how long does it take to receive your supplies after you have placed an order?<br><br>READ LIST.	Q31h. Is METHOD usually delivered or must you go get them?
(05) Female condoms	Govt…………… A<br>Intl NGO ……… B<br>Local NGO…….C<br>Pharmacy wholesaler/ dealer/ distributor………D<br>Other________X<br>(Specify)<br>Don't know….….Z	< 4 wks ago….......1<br>Between 4-12 wks………………2<br>> 12 wks ago……3<br>No routine supply system…………..4<br>Don't know………8	Determines own need….1 →**(Q31e)**<br>Determined Elsewhere……..2<br>Both..….……….....3<br>Don't know ………..8 →**(Q31g)**	Quantity based on activity level…..1<br>Standard fixed supply…………..2<br>Don't know……...8	Order to maintain stock……………..1<br>Order same amount…………2<br>Order based on consumption…..3<br>Other__________.6<br>(Specify)<br>Don't know…..…....8	Fall below predeter-mined level……….1<br>Fixed time – Every [__|__|__] days…….2<br>Order when needed..3<br>Other…………………6<br>Don't know………….8	One week or less…....1<br>Between 2-4 weeks….2<br>Between 5-8 weeks….3<br>More than 8 weeks…..4<br>Other _____________6<br>(Specify)<br><br>Don't know . ………….8	Delivered …….1<br><br>Pick them up…2<br><br>Both …………..3
(06) Injectables (e.g., Depo Provera, Noristerat)	Govt…………… A<br>Intl NGO ……… B<br>Local NGO…….C<br>Pharmacy wholesaler/ dealer/ distributor………D<br>Other________X<br>(Specify)<br>Don't know….….Z	< 4 wks ago….......1<br>Between 4-12 wks………………2<br>> 12 wks ago……3<br>No routine supply system…………..4<br>Don't know………8	Determines own need….1 →**(Q31e)**<br>Determined Elsewhere……..2<br>Both..….……….....3<br>Don't know ………..8 →**(Q31g)**	Quantity based on activity level…..1<br>Standard fixed supply…………..2<br>Don't know……...8	Order to maintain stock……………..1<br>Order same amount…………2<br>Order based on consumption…..3<br>Other__________.6<br>(Specify)<br>Don't know…..…....8	Fall below predeter-mined level……….1<br>Fixed time – Every [__|__|__] days…….2<br>Order when needed..3<br>Other…………………6<br>Don't know………….8	One week or less…....1<br>Between 2-4 weeks….2<br>Between 5-8 weeks….3<br>More than 8 weeks…..4<br>Other _____________6<br>(Specify)<br><br>Don't know . ………….8	Delivered …….1<br><br>Pick them up…2<br><br>Both …………..3
(07) Implant (Norplant)	Govt…………… A<br>Intl NGO ……… B<br>Local NGO…….C<br>Pharmacy wholesaler/ dealer/ distributor………D<br>Other________X<br>(Specify)<br>Don't know….….Z	< 4 wks ago….......1<br>Between 4-12 wks………………2<br>> 12 wks ago……3<br>No routine supply system…………..4<br>Don't know………8	Determines own need….1 →**(Q31e)**<br>Determined Elsewhere……..2<br>Both..….……….....3<br>Don't know ………..8 →**(Q31g)**	Quantity based on activity level…..1<br>Standard fixed supply…………..2<br>Don't know……...8	Order to maintain stock……………..1<br>Order same amount…………2<br>Order based on consumption…..3<br>Other__________.6<br>(Specify)<br>Don't know…..…....8	Fall below predeter-mined level……….1<br>Fixed time – Every [__|__|__] days…….2<br>Order when needed..3<br>Other…………………6<br>Don't know………….8	One week or less…....1<br>Between 2-4 weeks….2<br>Between 5-8 weeks….3<br>More than 8 weeks…..4<br>Other _____________6<br>(Specify)<br><br>Don't know . ………….8	Delivered …….1<br><br>Pick them up…2<br><br>Both …………..3
(08) IUCD	Govt…………… A<br>Intl NGO ……… B<br>Local NGO…….C<br>Pharmacy wholesaler/ dealer/ distributor………D<br>Other________X<br>(Specify)<br>Don't know….….Z	< 4 wks ago….......1<br>Between 4-12 wks………………2<br>> 12 wks ago……3<br>No routine supply system…………..4<br>Don't know………8	Determines own need….1 →**(Q31e)**<br>Determined Elsewhere……..2<br>Both..….……….....3<br>Don't know ………..8 →**(Q31g)**	Quantity based on activity level…..1<br>Standard fixed supply…………..2<br>Don't know……...8	Order to maintain stock……………..1<br>Order same amount…………2<br>Order based on consumption…..3<br>Other__________.6<br>(Specify)<br>Don't know…..…....8	Fall below predeter-mined level……….1<br>Fixed time – Every [__|__|__] days…….2<br>Order when needed..3<br>Other…………………6<br>Don't know………….8	One week or less…....1<br>Between 2-4 weeks….2<br>Between 5-8 weeks….3<br>More than 8 weeks…..4<br>Other _____________6<br>(Specify)<br><br>Don't know . ………….8	Delivered …….1<br><br>Pick them up…2<br><br>Both …………..3

(09) Other (specify)<br>____________	Govt…………… A<br>Intl NGO ……… B<br>Local NGO…….C<br>Pharmacy wholesaler/ dealer/ distributor………D<br>Other________X<br>(Specify)<br>Don't know….….Z	< 4 wks ago…......1<br>Between 4-12 wks……………….2<br>> 12 wks ago……3<br>No routine supply system…………..4<br>Don't know………8	Determines own need….1 →(Q31e)<br>Determined Elsewhere……..2<br>Both..….………...3<br>Don't know ………..8 →(Q31g)	Quantity based on activity level…..1<br>Standard fixed supply…………..2<br>Don't know……...8	Order to maintain stock……………..1<br>Order same amount………….2<br>Order based on consumption…..3<br>Other__________.6<br>(Specify)<br>Don't know…..……..8	Fall below predeter-mined level……….1<br>Fixed time – Every [__|__|__] days….…2<br>Order when needed..3<br>Other…………………6<br>Don't know………….8	One week or less…....1<br>Between 2-4 weeks….2<br>Between 5-8 weeks….3<br>More than 8 weeks…..4<br>Other _____________6<br>(Specify)<br>Don't know . ………….8	Delivered …….1<br>Pick them up…2<br>Both …………..3

Now I would like to ask you some more questions specifically about stock-outs of family planning methods.
ONLY ASK ABOUT THOSE METHODS THAT ARE AVAILABLE FROM Q30a.

METHOD	Q32a. Is ________ METHOD currently available?	Q32b. Has this facility had a stockout of METHOD that lasted at least 24 hours in the last **one year**?	Q32c. If Yes, how many times has this facility had a stockout of METHOD in the past one year? (CHECK if **"Yes"** to Q32b)	Q32d. If Yes, how many **total** days of stockout of METHOD did this facility experience in the past one year?<br><br>(CHECK if **"Yes"** to Q32b)	Q32e. SOURCE OF INFORMATION FOR STOCKOUTS IN PAST ONE YEAR:	Q32f. Has this facility had a stockout of METHOD that lasted at least 24 hours in the last 30 days (one month)?	Q32g. If Yes, how many times has this facility had a stockout of METHOD in the past 30 days?<br><br>(CHECK if **"Yes"** to Q32f)	Q32h. If Yes, how many **total** days of stockout of METHOD did this facility experience in the last 30 days?<br><br>(CHECK if **"Yes"** to Q32f)	Q32i. SOURCE OF INFORMATION ON STOCKOUTS IN PAST 30 DAYS:
(10) Combined oral pill	YES .....1<br>NO 2	YES .....1<br>NO ....2<br>↓<br>**(02)**	Number…<br><br>DON'T KNOW..98	Days…<br><br>CONSTANT PROBLEM…995<br>DK…………998	OBSERVED RECORD…………1<br><br>PROVIDER ESTIMATE……….2	YES .....1<br>NO ....2<br>↓<br>**(02)**	Number…<br><br>DON'T KNOW…..98	Days…<br><br>DON'T KNOW..98	OBSERVED RECORD…………1<br><br>PROVIDER ESTIMATE……….2
(11) Progestin only pill	YES .....1<br>NO 2	YES .....1<br>NO ....2<br>↓<br>**(03)**	Number…<br><br>DON'T KNOW..98	Days…<br><br>CONSTANT PROBLEM…995<br>DK…………998	OBSERVED RECORD…………1<br><br>PROVIDER ESTIMATE……….2	YES .....1<br>NO ....2<br>↓<br>**(03)**	Number…<br><br>DON'T KNOW…..98	Days…<br><br>DON'T KNOW..98	OBSERVED RECORD…………1<br><br>PROVIDER ESTIMATE……….2
(12) Emer-gency contrace-ptive	YES .....1<br>NO 2	YES .....1<br>NO ....2<br>↓<br>**(04)**	Number…<br><br>DON'T KNOW..98	Days…<br><br>CONSTANT PROBLEM…995<br>DK…………998	OBSERVED RECORD…………1<br><br>PROVIDER ESTIMATE……….2	YES .....1<br>NO ....2<br>↓<br>**(04)**	Number…<br><br>DON'T KNOW…..98	Days…<br><br>DON'T KNOW..98	OBSERVED RECORD…………1<br><br>PROVIDER ESTIMATE……….2
(13) Male condom	YES .....1<br>NO 2	YES .....1<br>NO ....2<br>↓<br>**(05)**	Number…<br><br>DON'T KNOW..98	Days…<br><br>CONSTANT PROBLEM…995<br>DK…………998	OBSERVED RECORD…………1<br><br>PROVIDER ESTIMATE……….2	YES .....1<br>NO ....2<br>↓<br>**(05)**	Number…<br><br>DON'T KNOW…..98	Days…<br><br>DON'T KNOW..98	OBSERVED RECORD…………1<br><br>PROVIDER ESTIMATE……….2

ONLY ASK ABOUT THOSE METHODS THAT ARE AVAILABLE FROM Q30a.									
METHOD	Q32a. Is ________ METHOD currently available?	Q32b. Has this facility had a stockout of METHOD that lasted at least 24 hours in the last **one year**?	Q32c. If Yes, how many times has this facility had a stockout of METHOD in the past one year?<br><br>(CHECK if **"Yes"** to Q32b)	Q32d. If Yes, how many **total** days of stockout of METHOD did this facility experience in the past one year?<br><br>(CHECK if **"Yes"** to Q32b)	Q32e. SOURCE OF INFORMATION FOR STOCKOUTS IN PAST ONE YEAR:	Q32f. Has this facility had a stockout of METHOD that lasted at least 24 hours in the last 30 days (one month)?	Q32g. If Yes, how many times has this facility had a stockout of METHOD in the past 30 days?<br><br>(CHECK if **"Yes"** to Q32f)	Q32h. If Yes, how many **total** days of stockout of METHOD did this facility experience in the last 30 days?<br><br>(CHECK if **"Yes"** to Q32f)	Q32i. SOURCE OF INFORMATION ON STOCKOUTS IN PAST 30 DAYS:
(14) Female condom	YES …..1<br>NO ……2	YES …..1<br>NO …….2 → **(06)**	Number… [ ][ ]<br>DON'T KNOW..98	Days… [ ][ ][ ]<br>CONSTANT PROBLEM…995<br>DK…………..998	OBSERVED RECORD……….1<br>PROVIDER ESTIMATE..……2	YES …..1<br>NO …….2 → **(06)**	Number… [ ][ ]<br>DON'T KNOW…..98	Days… [ ][ ]<br>DON'T KNOW..98	OBSERVED RECORD………….1<br>PROVIDER ESTIMATE………..2
(15) Injectable (DMPA, Noristerat)	YES …..1<br>NO ……2	YES …..1<br>NO …….2 → **(07)**	Number… [ ][ ]<br>DON'T KNOW..98	Days… [ ][ ][ ]<br>CONSTANT PROBLEM…995<br>DK…………..998	OBSERVED RECORD……….1<br>PROVIDER ESTIMATE..……2	YES …..1<br>NO …….2 → **(07)**	Number… [ ][ ]<br>DON'T KNOW…..98	Days… [ ][ ]<br>DON'T KNOW..98	OBSERVED RECORD………….1<br>PROVIDER ESTIMATE………..2
(16) Implants (Jadelle/ Implanon)	YES …..1<br>NO ……2	YES …..1<br>NO …….2 → **(08)**	Number… [ ][ ]<br>DON'T KNOW..98	Days… [ ][ ][ ]<br>CONSTANT PROBLEM…995<br>DK…………..998	OBSERVED RECORD……….1<br>PROVIDER ESTIMATE..……2	YES …..1<br>NO …….2 → **(08)**	Number… [ ][ ]<br>DON'T KNOW…..98	Days… [ ][ ]<br>DON'T KNOW..98	OBSERVED RECORD………….1<br>PROVIDER ESTIMATE………..2
(17) IUD	YES …..1<br>NO ……2	YES …..1<br>NO …….2 → **(09)**	Number… [ ][ ]<br>DON'T KNOW..98	Days… [ ][ ][ ]<br>CONSTANT PROBLEM…995<br>DK…………..998	OBSERVED RECORD……….1<br>PROVIDER ESTIMATE..……2	YES …..1<br>NO …….2 → **(09)**	Number… [ ][ ]<br>DON'T KNOW…..98	Days… [ ][ ]<br>DON'T KNOW..98	OBSERVED RECORD………….1<br>PROVIDER ESTIMATE………..2

ONLY ASK ABOUT THOSE METHODS THAT ARE AVAILABLE FROM Q30a.									
METHOD	Q32a. Is ________ METHOD currently available?	Q32b. Has this facility had a stockout of METHOD that lasted at least 24 hours in the last **one year**?	Q32c. If Yes, how many times has this facility had a stockout of METHOD in the past one year?<br><br>(CHECK if **"Yes"** to Q32b)	Q32d. If Yes, how many **total** days of stockout of METHOD did this facility experience in the past one year?<br><br>(CHECK if **"Yes"** to Q32b)	Q32e. SOURCE OF INFORMATION FOR STOCKOUTS IN PAST ONE YEAR:	Q32f. Has this facility had a stockout of METHOD that lasted at least 24 hours in the last 30 days (one month)?	Q32g. If Yes, how many times has this facility had a stockout of METHOD in the past 30 days?<br><br>(CHECK if **"Yes"** to Q32f)	Q32h. If Yes, how many **total** days of stockout of METHOD did this facility experience in the last 30 days?<br><br>(CHECK if **"Yes"** to Q32f)	Q32i. SOURCE OF INFORMATION ON STOCKOUTS IN PAST 30 DAYS:
(18) Other (specify)<br><br>____________	YES …..1<br>NO ……2	YES …..1<br>NO …….2 → **(Q33a)**	Number…<br>[ ][ ]<br>DON'T KNOW..98	Days…<br>[ ][ ][ ]<br>CONSTANT PROBLEM…995<br>DK………….998	OBSERVED RECORD……….1<br><br>PROVIDER ESTIMATE..……2	YES …..1<br>NO …….2 → **(Q33a)**	Number…<br>[ ][ ]<br>DON'T KNOW…..98	Days…<br>[ ][ ]<br>DON'T KNOW..98	OBSERVED RECORD…………1<br><br>PROVIDER ESTIMATE..……….2

ONLY ASK ABOUT THOSE METHODS THAT ARE OFFERED AT THE FACILITY FROM Q30a.		
METHOD	Q33a. How many [NAMED METHOD] do you usually provide to a **new acceptor** on her first visit?	Q33b. How many [NAMED METHOD] do you usually provide to a woman coming for **resupply/continuing to use the same method**?
(01) Combined oral contraceptives (number of cycles)	[ ][ ]	[ ][ ]
(02) Progestin-only oral contraceptives (number of cycles)	[ ][ ]	[ ][ ]
(03) Male condoms (number of pieces)	[ ][ ]	[ ][ ]
(04) Female condoms (number of pieces)	[ ][ ]	[ ][ ]

Now I'm going to ask you some questions related to how much clients pay for contraceptive services and methods.<br>**ONLY ASK ABOUT THOSE METHODS THAT ARE OFFERED BY THE FACILITY FROM Q30a.**				
METHOD	Q34a. How much is the consultation fee (in Naira) for METHOD/PROCEDURE?<br><br>OR<br><br>Package Deal (both consult and method/procedure)	Q34b. Do fees for METHOD vary depending on the product available?	Q34c. How much is the METHOD/PROCEDURE?<br><br>RECORD THE RANGE (in Naira) IF PRICE DIFFERS BY BRAND **FROM LOWEST TO HIGHEST PRICE.**<br><br>RECORD THE PRICE IN THE FIRST FIELD IF THERE IS ONLY ONE PRODUCT OR IF THE PRICE DOES NOT DIFFER BY BRAND	CHECK – IF OPTION Q34A AND Q34C is "FREE", GO TO NEXT METHOD<br><br>Q34d. What percent of clients pay the charge for METHOD/ PROCEDURE?
(01) Combined oral pill	CONSULTATION….1 [___|___|___|___]<br>FREE……………..99995<br>DON'T KNOW…….99998<br><br>OR<br>PACKAGE DEAL…..2 [___|___|___|___|___] → **Q34d**	Yes . . . ……………..1<br>No/only one brand or product available…... 2<br>Don't know …………8	[___|___|___|___]<br>TO<br>[___|___|___|___]per cycle<br><br>PRESCRIPTION/REFERRAL ONLY……..9994<br>FREE………………………,……………9995<br>DON'T KNOW……………………………9998	[___|___|___]
(02) Progestin only pill	CONSULTATION….1 [___|___|___|___]<br>FREE……………..99995<br>DON'T KNOW…….99998<br><br>OR<br>PACKAGE DEAL…..2 [___|___|___|___|___] → **Q34d**	Yes . . . ……………..1<br>No/only one brand or product available…... 2<br>Don't know …………8	[___|___|___|___]<br>TO<br>[___|___|___|___]per cycle<br><br>PRESCRIPTION/REFERRAL ONLY……..9994<br>FREE………………………,……………9995<br>DON'T KNOW……………………………9998	[___|___|___]
(03) Emergency contraceptive	CONSULTATION….1 [___|___|___|___]<br>FREE……………..99995<br>DON'T KNOW…….99998<br><br>OR<br>PACKAGE DEAL…..2 [___|___|___|___|___] → **Q34d**	Yes . . . ……………..1<br>No/only one brand or product available…... 2<br>Don't know …………8	[___|___|___|___]<br>TO<br>[___|___|___|___]per package/cycle<br><br>PRESCRIPTION/REFERRAL ONLY……..9994<br>FREE………………………,……………9995<br>DON'T KNOW……………………………9998	[___|___|___]
(04) Male condom	CONSULTATION….1 [___|___|___|___]<br>FREE……………..99995<br>DON'T KNOW…….99998<br><br>OR<br>PACKAGE DEAL…..2 [___|___|___|___|___] → **Q34d**	Yes . . . ……………..1<br>No/only one brand or product available…... 2<br>Don't know …………8	[___|___|___|___]<br>TO<br>[___|___|___|___]per piece<br><br>PRESCRIPTION/REFERRAL ONLY……..9994<br>FREE………………………,……………9995<br>DON'T KNOW……………………………9998	[___|___|___]

Now I'm going to ask you some questions related to how much clients pay for contraceptive services and methods.<br>**ONLY ASK ABOUT THOSE METHODS THAT ARE OFFERED BY THE FACILITY FROM Q30a.**				
METHOD	Q34a. How much is the consultation fee (in Naira) for METHOD/PROCEDURE?<br><br>OR<br><br>Package Deal (both consult and method/procedure)	Q34b. Do fees for METHOD vary depending on the product available?	Q34c. How much is the METHOD/PROCEDURE?<br><br>RECORD THE RANGE (in Naira) IF PRICE DIFFERS BY BRAND **FROM LOWEST TO HIGHEST PRICE.**<br><br>RECORD THE PRICE IN THE FIRST FIELD IF THERE IS ONLY ONE PRODUCT OR IF THE PRICE DOES NOT DIFFER BY BRAND	CHECK – IF OPTION Q34A AND Q34C is "FREE", GO TO NEXT METHOD<br><br>Q34d. What percent of clients pay the charge for METHOD/ PROCEDURE?
(05) Female condom	CONSULTATION….1 [___|___|___|___]<br>FREE……………..99995<br>DON'T KNOW…….99998<br><br>OR<br>PACKAGE DEAL…..2 [___|___|___|___|___] → **Q34d**	Yes . . . ……………1<br>No/only one brand or product available…... 2<br>Don't know …………8	[___|___|___|___|___]<br>TO<br>[___|___|___|___|___]per piece<br><br>PRESCRIPTION/REFERRAL ONLY……..9994<br>FREE………………………,……………9995<br>DON'T KNOW…………………………..9998	[___|___|___]
(06) Injectables [Depo (DMPA), Noristorat]	CONSULTATION….1 [___|___|___|___]<br>FREE……………..99995<br>DON'T KNOW…….99998<br><br>OR<br>PACKAGE DEAL…..2 [___|___|___|___|___] → **Q34d**	Yes . . . ……………1<br>No/only one brand or product available…... 2<br>Don't know …………8	[___|___|___|___|___]<br>TO<br>[___|___|___|___|___]per injectable<br><br>PRESCRIPTION/REFERRAL ONLY……..9994<br>FREE………………………,……………9995<br>DON'T KNOW…………………………..9998	[___|___|___]
(07) Implants (Jadelle/Implanon)	CONSULTATION….1 [___|___|___|___]<br>FREE……………..99995<br>DON'T KNOW…….99998<br><br>OR<br>PACKAGE DEAL…..2 [___|___|___|___|___] → **Q34d**	Yes . . . ……………1<br>No/only one brand or product available…... 2<br>Don't know …………8	[___|___|___|___|___|___]<br>TO<br>[___|___|___|___|___|___]per implant<br><br>PRESCRIPTION/REFERRAL ONLY……99994<br>FREE………………………,…………99995<br>DON'T KNOW…………………………99998	[___|___|___]
(08) IUD	CONSULTATION….1 [___|___|___|___]<br>FREE……………..99995<br>DON'T KNOW…….99998<br><br>OR<br>PACKAGE DEAL…..2 [___|___|___|___|___] → **Q34d**	Yes . . . ……………1<br>No/only one brand or product available…... 2<br>Don't know …………8	[___|___|___|___|___|___]<br>TO<br>[___|___|___|___|___|___]per IUD<br><br>PRESCRIPTION/REFERRAL ONLY…..99994<br>FREE………………………,…………99995<br>DON'T KNOW…………………………99998	[___|___|___]

Now I'm going to ask you some questions related to how much clients pay for contraceptive services and methods.<br>**ONLY ASK ABOUT THOSE METHODS THAT ARE OFFERED BY THE FACILITY FROM Q30a.**				
METHOD	Q34a. How much is the consultation fee (in Naira) for METHOD/PROCEDURE?<br><br>OR<br><br>Package Deal (both consult and method/procedure)	Q34b. Do fees for METHOD vary depending on the product available?	Q34c. How much is the METHOD/PROCEDURE?<br><br>RECORD THE RANGE (in Naira) IF PRICE DIFFERS BY BRAND **<u>FROM LOWEST TO HIGHEST PRICE.</u>**<br><br>RECORD THE PRICE IN THE FIRST FIELD IF THERE IS ONLY ONE PRODUCT OR IF THE PRICE DOES NOT DIFFER BY BRAND	CHECK – IF OPTION Q34A AND Q34C is "FREE", GO TO NEXT METHOD<br><br>Q34d. What percent of clients pay the charge for METHOD/ PROCEDURE?
(09) Female sterilization/ tubal ligation	CONSULTATION….1 [___|___|___|___]<br>FREE……………..99995<br>DON'T KNOW…....99998<br><br>OR<br>PACKAGE DEAL…..2 [___|___|___|___|___] → **Q34d**		[___|___|___|___|___]<br>TO<br>[___|___|___|___|___] PER OPERATION<br><br>REFERRAL ONLY…………………….99994<br>FREE…………………….,…………..99995<br>DON'T KNOW………..……………..…99998	[___|___|___]
(10) Male sterilization	CONSULTATION….1 [___|___|___|___]<br>FREE……………..99995<br>DON'T KNOW…....99998<br><br>OR<br>PACKAGE DEAL…..2 [___|___|___|___|___] → **Q34d**		[___|___|___|___|___]<br>TO<br>[___|___|___|___|___] PER OPERATION<br><br>REFERRAL ONLY…………………...…99994<br>FREE……………………….,…………99995<br>DON'T KNOW………..………………99998	[___|___|___]
(11) Other (specify)<br>______________	CONSULTATION….1 [___|___|___|___]<br>FREE……………..99995<br>DON'T KNOW…….99998<br><br>OR<br>PACKAGE DEAL…..2 [___|___|___|___|___] → **Q34d**	Yes . . . ……………..1<br>No/only one brand or product available… . . 2<br>Don't know ……….8 → **Q34d**	[___|___|___|___]<br>TO<br>[___|___|___|___]per UNIT<br><br>PRESCRIPTION/REFERRAL ONLY….9994<br>FREE…………………,…….………9995<br>DON'T KNOW…….…………….……9998	[___|___|___]

SERVICE STATISTICS Now I want to ask about service statistics for the following contraceptive methods.. For each method I ask about, please tell me the number of new acceptors/users and the number of resupply/continuing users for both the last month and the last 12 months.

Q35a.How many clients received family planning services in the ***last 12 completed months?***	Q35b. Total new family planning acceptors/users in the ***last 12 completed months?***	Q35c. Total FP visits in the ***last 12 completed months?***	Q35d. INDICATE WHERE STATISTICS COME FROM:
[___|___|___|___|___|___] NOT AVAILABLE …999993	[___|___|___|___|___|___] NOT AVAILABLE ……999993 NOTE: New acceptors/users = new to clinic and those who switch methods on day of service.	[___|___|___|___|___|___] NOT AVAILABLE …999993	OBSERVED ………..1 ESTIMATED………..2 NOT AVAILABLE……3 OTHER:__________6 (SPECIFY)

Q36.	INDICATE **BEGINNING** MONTH AND YEAR FOR Q35a-Q35c ABOVE . . . . [__|__] . . [__|__|__|__] MONTH YEAR
Q37.	INDICATE **ENDING** MONTH AND YEAR FOR Q35a-Q35c ABOVE. . . . . [__|__] . . . [__|__|__|__] MONTH YEAR

METHOD <br>**ONLY ASK ABOUT THOSE METHODS THAT ARE OFFERED IN Q30a.**	Q38a. Number of new acceptors/users **last month**	Q38b. Number of resupply/continuing clients **last month**	Q38c. Number of new acceptors/users **last 12 months**	Q38d. Number of resupply/continuing clients **last 12 months**	Q38e. INDICATE WHERE STATISTICS COME FROM:
(01) Combined oral pill	[__|__|__|__] NOT AVAILABLE ……………..9993	[__|__|__|__] NOT AVAILABLE ……………..9993	[__|__|__|__|__] NOT AVAILABLE ……………..99993	[__|__|__|__|__] NOT AVAILABLE ……………..99993	OBSERVED ……………………..1 ESTIMATED ……………………..2 NOT AVAILABLE………………...3 OTHER:__________________6 (SPECIFY)
(02) Progestin only pill	[__|__|__|__] NOT AVAILABLE ……………..9993	[__|__|__|__] NOT AVAILABLE ……………..9993	[__|__|__|__|__] NOT AVAILABLE ……………..99993	[__|__|__|__|__] NOT AVAILABLE ……………..99993	OBSERVED ……………………..1 ESTIMATED ……………………..2 NOT AVAILABLE………………...3 OTHER:__________________6 (SPECIFY)
(03) Emergency contraceptive	[__|__|__|__] NOT AVAILABLE ……………..9993	[__|__|__|__] NOT AVAILABLE ……………..9993	[__|__|__|__|__] NOT AVAILABLE ……………..99993	[__|__|__|__|__] NOT AVAILABLE ……………..99993	OBSERVED ……………………..1 ESTIMATED ……………………..2 NOT AVAILABLE………………...3 OTHER:__________________6 (SPECIFY)
(04) Male condom	[__|__|__|__] NOT AVAILABLE ……………..9993	[__|__|__|__] NOT AVAILABLE ……………..9993	[__|__|__|__|__] NOT AVAILABLE ……………..99993	[__|__|__|__|__] NOT AVAILABLE ……………..99993	OBSERVED ……………………..1 ESTIMATED ……………………..2 NOT AVAILABLE………………...3 OTHER:__________________6 (SPECIFY)

METHOD **ONLY ASK ABOUT THOSE METHODS THAT ARE OFFERED IN Q30a.**	Q38a. Number of new acceptors/users **last month**	Q38b. Number of resupply/continuing clients **last month**	Q38c. Number of new acceptors/users **last 12 months**	Q38d. Number of resupply/continuing clients **last 12 months**	Q38e. INDICATE WHERE STATISTICS COME FROM:
(05) Female condom	[___|___|___|___|___]<br>NOT AVAILABLE ……………..9993	[___|___|___|___|___]<br>NOT AVAILABLE ……………..9993	[___|___|___|___|___|___]<br>NOT AVAILABLE ……………..99993	[___|___|___|___|___|___]<br>NOT AVAILABLE ……………..99993	OBSERVED ……………………..1<br>ESTIMATED ……………………..2<br>NOT AVAILABLE…………………..3<br>OTHER:________________6<br>(SPECIFY)
(06) Injectables (Depo/ Noristerat)	[___|___|___|___|___]<br>NOT AVAILABLE ……………..9993	[___|___|___|___|___]<br>NOT AVAILABLE ……………..9993	[___|___|___|___|___|___]<br>NOT AVAILABLE ……………..99993	[___|___|___|___|___|___]<br>NOT AVAILABLE ……………..99993	OBSERVED ……………………..1<br>ESTIMATED ……………………..2<br>NOT AVAILABLE…………………..3<br>OTHER:________________6<br>(SPECIFY)
(07) Implants (Jadelle/ Implanon)	[___|___|___|___|___]<br>NOT AVAILABLE ……………..9993	[___|___|___|___|___]<br>NOT AVAILABLE ……………..9993	[___|___|___|___|___|___]<br>NOT AVAILABLE ……………..99993	[___|___|___|___|___|___]<br>NOT AVAILABLE ……………..99993	OBSERVED ……………………..1<br>ESTIMATED ……………………..2<br>NOT AVAILABLE…………………..3<br>OTHER:________________6<br>(SPECIFY)
(08) IUD	[___|___|___|___|___]<br>NOT AVAILABLE ……………..9993	[___|___|___|___|___]<br>NOT AVAILABLE ……………..9993	[___|___|___|___|___|___]<br>NOT AVAILABLE ……………..99993	[___|___|___|___|___|___]<br>NOT AVAILABLE ……………..99993	OBSERVED ……………………..1<br>ESTIMATED ……………………..2<br>NOT AVAILABLE…………………..3<br>OTHER:________________6<br>(SPECIFY)
(09) Female sterilization	[___|___|___|___|___]<br>NOT AVAILABLE ……………..9993		[___|___|___|___|___|___]<br>NOT AVAILABLE ……………..99993		OBSERVED ……………………..1<br>ESTIMATED ……………………..2<br>NOT AVAILABLE…………………..3<br>OTHER:________________6<br>(SPECIFY)
(10) Male sterilization	[___|___|___|___|___]<br>NOT AVAILABLE ……………..9993		[___|___|___|___|___|___]<br>NOT AVAILABLE ……………..99993		OBSERVED ……………………..1<br>ESTIMATED ……………………..2<br>NOT AVAILABLE…………………..3<br>OTHER:________________6<br>(SPECIFY)
(11) Other (specify)<br>________________	[___|___|___|___|___]<br>NOT AVAILABLE ……………..9993	[___|___|___|___|___]<br>NOT AVAILABLE ……………..9993	[___|___|___|___|___|___]<br>NOT AVAILABLE ……………..99993	[___|___|___|___|___|___]<br>NOT AVAILABLE ……………..99993	OBSERVED ……………………..1<br>ESTIMATED ……………………..2<br>NOT AVAILABLE…………………..3<br>OTHER:________________6<br>(SPECIFY)

Q39.	INDICATE **MONTH** OF RECORDS FOR Q38a-Q38b ABOVE . . . . [__|__] . . .<br>(I.e., For February, record "02")
Q40a.	INDICATE **BEGINNING** MONTH AND YEAR FOR Q38c-Q38d ABOVE . . . . [__|__] . . [__|__|__|__]<br>MONTH YEAR
Q40b.	INDICATE **ENDING** MONTH AND YEAR FOR Q38c-Q38d ABOVE. . . . . [__|__] . . . [__|__|__|__]<br>MONTH YEAR

IEC MATERIALS AND OUTREACH ACTIVITIES

Q41.	Are the following family planning IEC materials displayed and/or available for use?	OBSERVED	REPORTED, NOT SEEN	NOT AVAILABLE	DON'T KNOW
	a) Posters	1	2	3	8
	b) Informational flip chart	1	2	3	8
	c) Brochures/pamphlets	1	2	3	8
	d) Information sheets	1	2	3	8
	e) Job aids	1	2	3	8
	f) Demonstration models	1	2	3	8
	g) Counseling cards	1	2	3	8
	h) Samples of various FP methods	1	2	3	8
	i) Other (specify) ________________	1	2	3	8

Q42.	Does this facility have a health outreach program for IEC (Information, Education and Communication)?	Yes . . . .1<br>No . . . . 2 →<br>Don't know……………..…8 →	 **Q46**<br>**Q46**
Q43.	Does this outreach program discuss family planning/birth spacing?	Yes . . . . 1<br>No . . . . 2<br>Don't know………………..8	
Q44.	How many communities are regularly visited through this outreach program?	NUMBER . . . .[___|___]	
Q45.	About how often are these communities visited through this outreach program?	WEEKLY…………………..……1<br>MONTHLY . . . . 2<br>QUARTERLY . . . . 3<br>ANNUALLY . . . . 4<br>OTHER __________________6<br>(Specify)	
Q46.	Does this facility give health talks for members of the community?	Yes . . . . 1<br>No . . . . 2 →<br>Don't know………….8 →	 **Q50**<br>**Q50**
Q47.	Has this facility ever given a health talk on family planning/birth spacing to the community?	Yes . . . .…1<br>No . . . . 2<br>Don't know………………..8	
Q48.	How often does this facility give health talks to the community?	EVERY DAY . . . . 1<br>WEEKLY . . . . 2<br>MONTHLY……………………..3<br>QUARTERLY…………………4<br>OTHER_________________6<br>(Specify)	

Q49.	How often do the topics of the health talks change?	EVERY DAY . . . . 1<br>WEEKLY . . . . 2<br>MONTHLY……………………..3<br>QUARTERLY…………………4<br>OTHER_________________6<br>(Specify)	
Q50.	Does this facility supervise CBDs (community-based distributors of contraceptives)?	YES . . . . 1<br>NO . . . . 2 →	 **Q52**
Q51.	What organization sponsors the CBDs?<br><br>**CIRCLE ALL THAT APPLY**	MOH . . . . .A<br>MARIE STOPES . . . . .B<br>PPFN…………………………….C<br>SFH………………………………..D<br>OTHER _________________X<br>(SPECIFY)	

QUALITY ASSURANCE/STANDARD OPERATING PROCEDURES			
Now I want to ask about common quality assurance activities and guidelines. For each activity or guideline mentioned, please tell me if this exists anywhere in the facility.<br><br>IF QUALITY ASSURANCE ACTIVITIES ARE REPORTED TO BE CARRIED OUT, ASK: Can I see some document or record that shows this has been carried out during the past year?<br><br>A REPORT OR MINUTES OF A MEETING WHICH MENTIONS THE QUALITY ASSURANCE ACTIVITY IS ACCEPTABLE.			
Q52.	Are there any written guidelines or service protocols in this facility for family planning services?	Yes, document observed…………1<br>Yes, document reported but not seen………………………………2<br>No……………………………………3 →<br>Don't know …………………………8 →	 <br><br>**Q54**<br>**Q54**
Q53.	Who is the author of these guidelines or service protocols you are using?<br>NAME OF GUIDELINES:______________________<br>______________________________<br>______________________________<br><br>**CHOOSE ALL THAT APPLY.**	Facility created guidelines……….A<br>WHO guidelines……………………B<br>FMOH guidelines…………………..C<br>Other ____________________X<br>(Specify)	
Q54	Are there any written guidelines or service protocols in this facility for the integration of family planning and HIV services?	Yes, document observed…………1<br>Yes, document reported but not seen………………………………2<br>No……………………………………3<br>Don't know …………………………8	
Q55.	Are you using any guideline(s) or tool(s) to screen patients for pregnancy?	Yes, document observed…………1<br>Yes, document reported but not seen………………………………2<br>No……………………………………3 →<br>Don't know …………………………8 →	 <br><br>**Q57**<br>**Q57**
Q56.	Do these guideline(s) recommend that you screen all patients for pregnancy before dispensing a new family planning method?	Yes ……………………………………1<br>No ……………………………………2<br>Other guidance provided<br>________________________6<br>(Specify)<br>Don't know…………………………8	
Q57.	Do any of the guidelines recommend that family planning counseling is offered to most clients in this facility as a routine or normal practice?	Yes ……………………………………1<br>No ……………………………………2<br>No guidelines……………………….3<br>Other guidance provided<br>________________________ 6<br>(Specify)<br>Don't know…………………………8	
Q58.	Are periodic audits or reports of medical records or service registers conducted/compiled at least quarterly?	Yes, document observed……………1<br>Yes, document reported but not seen………………………………2<br>No……………………………………3<br>Don't know …………………………8	
Q59.	Is there any type of quality assurance committee or staff meetings that assure quality control for family planning service delivery?	Yes, document observed……………1<br>Yes, document reported but not seen………………………………2<br>No……………………………………3<br>Don't know …………………………8	

**STORAGE:** Now I would like to see the place where contraceptive methods are stored. We are just trying to get an idea of how facilities keep their stock and store contraceptive methods. Remember that my findings will be just used for research purposes and will be kept strictly confidential.			
Q60.	OBSERVE WHETHER ALL THE CONTRACEPTIVE METHODS ARE PROTECTED FROM WATER OR DAMPNESS	YES . . . . 1<br>NO . . . . 2<br>CANNOT OBSERVE STORAGE AREA……………….………………..3 →	**Q66**
Q61.	OBSERVE WHETHER ALL THE CONTRACEPTIVE METHODS ARE OFF THE FLOOR	YES . . . . 1<br>NO 2	
Q62.	OBSERVE WHETHER THE CEILING ABOVE THE CONTRACEPTIVE METHODS IS INTACT AND NOT LEAKING	YES . . . . 1<br>NO 2	
Q63.	OBSERVE WHETHER ALL THE CONTRACEPTIVE METHODS ARE PROTECTED FROM THE SUN.	YES . . . . 1<br>NO 2	
Q64.	OBSERVE WHETHER THE ROOM IS CLEAN OF EVIDENCE OF RODENTS (BATS, RATS) OR PESTS (ROACHES, ETC).	YES . . . . 1<br>NO 2	
Q65.	OBSERVE WHETHER THE INJECTABLES ARE STORED UPRIGHT.	YES . . . . 1<br>NO . . . . 2<br>NOT APPLICABLE/DON'T PROVIDE INJECTABLES……….7	
Q66.	Does the pharmacy separate damaged and/or expired family planning methods from the usable products, and remove them from the inventory?<br><br>IF YES, ASK TO SEE EVIDENCE OF EACH OF THE INDICATED PRACTICES AND ALL THAT WERE OBSERVED. ALSO ASK FOR THE TALLY CARD TO CHECK FOR RECORDED BALANCE.	YES, DAMAGED/EXPIRED ITEM REMOVED FROM INVENTORY . . . ………………….. …... . . . . 1<br><br>REMOVED FROM SHELVES AND NO EXPIRED ITEMS PRESENT………………………………..….. ... . 2<br><br>EXPIRED ITEMS OBSERVED . . . . . . ……. .. 3<br><br>REPORTED YES BUT CANNOT OBSERVE….4<br><br>NO 5	

Now, I would like to ask you some questions about the physical infrastructure and equipment that you have at this facility.				
**PHYSICAL INFRASTRUCTURE AND EQUIPMENT**				
Are the following types of facilities/equipment available on a functioning basis at the service location?<br>INTERVIEWER NEEDS TO CHECK FUNCTIONING WHERE POSSIBLE.				
Q67.	DOES THIS FACILITY HAVE A SIGN POSTED WITH ITS HOURS OF OPERATION AND SERVICES?	Observed, both hours and services. .1<br>Observed, hours only…………………………... . . . . 2<br>Observed, services only………………………………………….3<br>Reported, both hours and services……………………………..4<br>Reported, hours only……………………………………………5<br>Reported, services only…………………………………………6<br>No sign……………………………………………………………7		
		**Not Available**	**Available but not functioning**	**Available and functioning**
Q68.	Electricity	1	2	3
Q69.	Back-up generator	1	2	3
Q70.	Piped water supply	1	2	3
Q71.	Toilet facilities/latrine	1	2	3
Q72.	Telephone/GSM (dedicated to the facility)	1	2	3
Q73.	Storage area for drugs and supplies	1	2	3
Q74.	Sharps container for needles	1	2	3
Q75.	Laboratory	1	2	3
Q76.	Private examination room (ie, a private room for pelvic exams and IUD insertion)	1	2	3
Q77.	Exam table for gynecological examination	1	2	3
Q78.	Examination light	1	2	3
Q79.	Delivery room with bed and lighting	1	2	3
Q80.	Operating theatre with basic/required equipment	1	2	3
Q81.	Weighing scale for adults	1	2	3
Q82.	Infant weighing scale	1	2	3
Q83.	Blood pressure apparatus	1	2	3
Q84.	Stethoscope	1	2	3
Q85.	Fetal stethoscope	1	2	3
Q86.	Sterilizer	1	2	3
Q87.	Microscope	1	2	3
Q88.	Oxygen apparatus	1	2	3
Q89.	Centrifuge	1	2	3
Q90.	Thermometer	1	2	3
Q91.	Scalpels	1	2	3
Q92.	Two pairs of scissors	1	2	3
Q93.	Long needle holder	1	2	3
Q94.	Forceps	1	2	3
Q95.	Sponge holding forceps	1	2	3
Q96.	Tenacula (Volsellum forcepts)	1	2	3
Q97.	Vaginal speculum (small size)	1	2	3
Q98.	Vaginal speculum (medium size)	1	2	3
Q99.	Vaginal speculum (large size)	1	2	3
Q100.	Minor surgery kit (e.g. artery forceps, hemostat)	1	2	3
Q101.	Vacuum extractor	1	2	3

		Not Available	Available but not functioning	Available and functioning
Q102.	Manual vacuum aspiration (MVA) kit	1	2	3
Q103.	Minilaparotomy kit	1	2	3
Q104.	Uterine hook	1	2	3
Q105.	Tubal hook	1	2	3
Q106.	Vasectomy kit	1	2	3
Q107.	Uterine sounds	1	2	3
Q108.	Canula and trochar for inserting implants	1	2	3

Now, I would like to ask you some questions about the physical infrastructure and equipment that you have at this facility.

CONSUMABLE SUPPLIES

Are the following types of supplies available on a regular basis at the service location?
INTERVIEWER NEEDS TO CHECK AVAILABILITY WHERE POSSIBLE.

		Not Available	Available sometimes but not on a regular basis	Available all of the time
Q109.	Sutures	1	2	3
Q110.	Antiseptic solution (such as iodine)	1	2	3
Q111.	Methylated spirit	1	2	3
Q112.	Sterile gauze pad or cotton wool	1	2	3
Q113.	Sterile disposable latex gloves	1	2	3
Q114.	Long gloves	1	2	3
Q115.	Disposable sterile syringes and needles	1	2	3
Q116.	Intravenous kit	1	2	3
Q117.	Scalpel blades	1	2	3
Q118.	Sealed implants pack (for performing FP implant insertions)	1	2	3
Q119.	Sedatives (such as Valium)	1	2	3
Q120.	Atropine (such as Buscopan)	1	2	3
Q121.	Opioid analgesic	1	2	3
Q122.	Local anesthetic (such as lignocaine)	1	2	3

Q123.	RECORD THE TIME<br>[24-HOUR TIME] Hour ………. [ ][ ]<br>Minutes …….. [ ][ ]

Thank you very much for taking the time to answer my questions. Once again, any information you have given will be kept confidential. Have a good day!

COMMENTS:
