# Supplementary material for: Facility-level characteristics associated with family planning and child immunization services integration in urban areas of Nigeria: a longitudinal analysis
Source: BMC Public Health. 2021 Jul 12;21:1379. doi: 10.1186/s12889-021-11436-x (PMC8274034; doi:10.1186/s12889-021-11436-x)
Supplement: Supplementary file 2 — Additional file 2. Baseline Provider Survey. [file 12889_2021_11436_MOESM2_ESM.pdf]

QUESTIONNAIRE IDENTIFICATION NO:[__|__|__|__|__|__|__|__]
5 digit facility code + 3 digit prov code

MLE MEASUREMENT, LEARNING & EVALUATION PROJECT FOR THE URBAN REPRODUCTIVE HEALTH INITIATIVE

Measurement, Learning & Evaluation (MLE) Project
Service Provider – Nigeria - 2011

IDENTIFICATION	
CITY NAME & CODE ____________________ (Abuja=1, Benin=2, Ibadan=3, Ilorin=4, Kaduna=5, Zaria=6)	[__]
LGA NAME & CODE____________________	[__|__|__]
LOCALITY NAME & CODE ____________________	[__|__|__|__]
FACILITY NAME AND CODE ____________________	[__|__|__|__|__]
PROVIDER NAME AND CODE (FROM THE FACILITY AUDIT LIST – Q8d)____________	[__|__|__]
RESPONDENT: NOT INTERVIEWED = 1 PREVIOUSLY INTERVIEWED IN THIS FACILITY = 2 (END)	[ ]
IF PREVIOUSLY INTERVIEWED, OTHER FACILITY NAME AND CODE____________	[ ][ ][ ][ ][ ]

INTERVIEWER VISITS

VISIT No.	1	2	3	FINAL VISIT
DATE	DAY/ MONTH/YEAR [___/____/_11_]	DAY/ MONTH/ YEAR [___/____/__11_]	DAY/ MONTH/ YEAR [___/____/__11_]	DAY [___|___] MONTH [___|___] YEAR [_2_I_0_I_1_I_1_]
INTERVIEWER'S NAME	________	________	________	________
INTERVIEWER CODE	[ ][ ][ ]	[ ][ ][ ]	[ ][ ][ ]	[ ][ ][ ]
RESULT*	[ ]	[ ]	[ ]	[ ]
**NEXT VISIT:** **DATE:**	[___/____/11_]	[___/____/11_]	[___/____/11_]	TOTAL NO. OF VISITS [ ]
**TIME:**	[ ][ ] [ ][ ] H H M M	[ ][ ] [ ][ ] H H M M	[ ][ ] [ ][ ] H H M M	

***RESULT CODES:**

1. COMPLETED
2. RESPONDENT NOT AVAILABLE
3. POSTPONED
4. REFUSED
5. PARTLY COMPLETED
6. OTHER ____________________ (Specify)

QUESTIONNAIRE IDENTIFICATION NO:[__|__|__|__|__|__|__|__]
5 digit facility code + 3 digit prov code

SUPERVISOR	OFFICE EDITOR	KEYED BY
NAME ________________________	NAME __________________________	NAME ________________________
CODE [____|____]	CODE [____|____]	CODE [____|____]
DATE [____/_____/_**11**_] DD MM YY	DATE [____/_____/_**11**_] DD MM YY	DATE [____/_____/_**11**_] DD MM YY

**BACKGROUND INFORMATION**			
**Source**	**Questions**	**Coding**	**Skip**
Q1.	RECORD THE TIME<br><br>(IN 24 HOUR FORMAT)	Hour ………… [|] Minutes …….. [|]	
Q2.	SEX OF PROVIDER INTERVIEWED	MALE………………………………….1<br>FEMALE……………………….……..2	
Q3.	How long have you been working here at this facility?	YEARS… [|]<br><br>LESS THAN ONE YEAR =00<br>DON'T KNOW = 98	
Q4.	What cadre of staff are you?	OBSTETRICIAN/GYNECOLOGIST……………..……….…...01<br>GENERAL SURGEON……………………………..…..…..…02<br>PEDIATRICIAN…………………………………………...…...…03<br>GENERAL PHYSICIAN………………………………………..04<br>THEATRE NURSE…………………………………….……05<br>NURSE/MIDWIFE………………………………………………06<br>NURSE……………………………………………………........07<br>MIDWIFE…………………………..………………….………08<br>COMMUNITY HEALTH EXTENSION WORKER (CHEW)…09<br>COMMUNITY HEALTH OFFICER (CHO)……………………10<br>VCT COUNSELOR…………………………………….……..11<br>OTHER ____96<br>(SPECIFY)	
Q5.	How old were you at your last birthday?	YEARS…….. [|]	
Q6.	What is your religion?	CHRISTIAN-CATHOLIC………………………………………01<br>CHRISTIAN-PROTESTANT/OTHER CHRISTIAN…………02<br>ISLAM………………………...……………………………03<br>TRADITIONAL……………………………………………………04<br>NO RELIGION …………………………………………...…05<br><br>OTHER____06<br>(SPECIFY)	
Q7.	In which department or unit do you work?	GENERAL OUTPATIENT DEPARTMENT (GOPD) ... 01<br>OBSTETRICS AND GYNECOLOGY …...……………… .02<br>SURGERY …….………………………………..………..03<br>PEDIATRICS ……….…………………………………04<br>FAMILY PLANNING DEPARTMENT ….………………05<br>INFANT AND CHILD CARE ……………………………..06<br>ANC……………………………………….……….....……07<br>HIV TESTING OR STI/HIV TREATMENT……………..08<br>Other ______________________________________96<br>(SPECIFY)	
Q8.	How many years have you been working as a health care provider?	NUMBER OF YEARS: [|]	
Q9.	How many years ago did you finish your **pre-service** training?	YEARS AGO….. [|]<br><br>LESS THAN ONE YEAR = 00<br>NO PRE-SERVICE TRAINING=97	

QUESTIONNAIRE IDENTIFICATION NO:[___|___|___|___|___|___|___|___]
5 digit facility code + 3 digit prov code

Q10.	Have you received any **in-service** training on family planning?	YES…………………………………1<br>NO…………………………………..2 →	Q12
Q11.	How long ago was the last **in-service** family planning training that you attended?	DAYS AGO……….1<br>WEEKS AGO……..2<br>MONTHS AGO…...3<br>YEARS AGO……...4<br>DON'T REMEMBER….998 [__|__]	

TRAINING ON FAMILY PLANNING

Now, I will ask you few questions related to training on FP.

Q12. CHECK Q09 AND Q10 ON PRE-SERVICE AND IN-SERVICE TRAINING:

HAS HAD BOTH PRE AND IN-SERVICE TRAINING ☐
(Q9=00 OR HIGHER AND Q10=1)
THEN ANSWER Q12a-Q12d

HAS HAD IN-SERVICE TRAINING ONLY ☐ → **Q12b**
(Q9=97 AND Q10=1)

HAS HAD PRE-SERVICE TRAINING ONLY ☐
(Q9=00 OR GREATER AND Q10=2)
THEN ANSWER 12a ONLY

HAS NOT HAD ANY PRE OR IN SERVICE TRAINING ☐ → **Q13**
(Q9=97 AND Q10=2)

TOPICS		Q12a. Did your **pre-service** training cover TOPIC?	Q12b. Have you ever attended an **in-service** training on TOPIC?	Q12c. What year was your most recent **in-service** training on TOPIC?	Q12d. Which organization or government ministry conducted this training?<br><br>LIST NAME OF ORGANIZATION.
(01)	Contraceptive technology update	YES . . . . .1<br>NO . . . . 2<br>DK…………..8	YES . . . . .1<br>NO . . . . .2 →**(02)**	[___|___|___|___]<br>**DK=9998**	____________<br>|___|___|
(02)	Exclusive breastfeeding counseling/LAM	YES . . . . .1<br>NO . . . . 2<br>DK…………..8	YES . . . . .1<br>NO . . . . .2 →**(03)**	[___|___|___|___]<br>**DK=9998**	____________<br>|___|___|
(03)	Natural family planning (rhythm method, cycle beads, etc.)	YES . . . . .1<br>NO . . . . 2<br>DK…………..8	YES . . . . .1<br>NO . . . . .2 →**(04)**	[___|___|___|___]<br>**DK=9998**	____________<br>|___|___|
(04)	Emergency Contraceptive	YES . . . . .1<br>NO . . . . 2<br>DK…………..8	YES . . . . .1<br>NO . . . . .2 →**(05)**	[___|___|___|___]<br>**DK=9998**	____________<br>|___|___|
(05)	Oral pills	YES . . . . .1<br>NO . . . . 2<br>DK…………..8	YES . . . . .1<br>NO . . . . .2 →**(06)**	[___|___|___|___]<br>**DK=9998**	____________<br>|___|___|
(06)	FP counseling skills	YES . . . . .1<br>NO . . . . 2<br>DK…………..8	YES . . . . .1<br>NO . . . . .2 →**(07)**	[___|___|___|___]<br>**DK=9998**	____________<br>|___|___|
(07)	Clinical skills on IUD	YES . . . . .1<br>NO . . . . 2<br>DK…………..8	YES . . . . .1<br>NO . . . . .2 →**(08)**	[___|___|___|___]<br>**DK=9998**	____________<br>|___|___|
(08)	Clinical skills on injectable contraceptive	YES . . . . .1<br>NO . . . . 2<br>DK…………..8	YES . . . . .1<br>NO . . . . .2 →**(09)**	[___|___|___|___]<br>**DK=9998**	____________<br>|___|___|

TOPICS		Q12a. Did your **pre-service** training cover TOPIC?	Q12b. Have you ever attended an **in-service** training on TOPIC?	Q12c. What year was your most recent **in-service** training on TOPIC?	Q12d. Which organization or government ministry conducted this training?<br><br>LIST NAME OF ORGANIZATION.
(09)	Clinical skills on implant	YES . . . . .1<br>NO . . . . 2<br>DK…………..8	YES . . . . .1<br>NO . . . . .2 →**(10)**	[___|___|___|___]<br>**DK=9998**	____________________<br>|___|___|
(10)	Clinical skills on Female Sterilization	YES . . . . .1<br>NO . . . . 2<br>DK…………..8	YES . . . . .1<br>NO . . . . .2 →**(11)**	[___|___|___|___]<br>**DK=9998**	____________________<br>|___|___|
(11)	Clinical skills on male sterilization	YES . . . . .1<br>NO . . . . 2<br>DK…………..8	YES . . . . .1<br>NO . . . . .2 →**(12)**	[___|___|___|___]<br>**DK=9998**	____________________<br>|___|___|
(12)	Management of incomplete abortion (Post-Abortion Care)	YES . . . . .1<br>NO . . . . 2<br>DK…………..8	YES . . . . .1<br>NO . . . . .2 →**(13)**	[___|___|___|___]<br>**DK=9998**	____________________<br>|___|___|
(13)	Manual vacuum aspiration (MVA)	YES . . . . .1<br>NO . . . . 2<br>DK…………..8	YES . . . . .1<br>NO . . . . .2 →**(14)**	[___|___|___|___]<br>**DK=9998**	____________________<br>|___|___|

Now I would like to ask you some questions about your knowledge and provision of various methods of family planning. If you have provided a particular method before, we are also interested in the availability and quality of the materials required to provide that method.						
METHOD	13a. Can you please tell me which of the following best describes your knowledge of [METHOD]:<br>1. You know METHOD sufficiently well to counsel and provide/assist in provision to a client;<br>2. You know METHOD sufficiently well to counsel, but not to provide;<br>3. You know little about METHOD and would not feel comfortable counseling or providing;<br>8. You know do not know METHOD at all	13b. Have you provided (assisted with) [METHOD] to clients at this facility?	13c. Have you experienced any stockouts in this facility that lasted more than 24 hours of [METHOD] in the last one year?	13d. If yes, how many total days of stockouts did this facility have in the last ONE YEAR of [METHOD] (all stockouts combined)?	13e. Have you experienced a lack of essential equipment needed to provide [METHOD] in the last ONE YEAR?	13f. If Yes, how many total days did you lack essential equipment needed to provide [METHOD] in the last ONE YEAR?
(01) Combined oral pill	PROVIDE & COUNSEL…………….1<br>COUNSEL, NOT PROVIDE…..2<br>KNOW LITTLE ABOUT……….3<br>DO NOT KNOW……………….8<br>(2, 3, 8) → (02)	YES……..1<br>NO………2 → (02)	YES………..1<br>NO…………2 → (02)<br>PRESCRIPTION ONLY……….3 → (02)	DAYS… [__][__][__]<br>CONSTANT PROBLEM…995<br>DON'T KNOW..998		
(02) Progestin-only pill	PROVIDE & COUNSEL…………….1<br>COUNSEL, NOT PROVIDE…..2<br>KNOW LITTLE ABOUT……….3<br>DO NOT KNOW……………….8<br>(2, 3, 8) → (03)	YES……..1<br>NO………2 → (03)	YES………..1<br>NO…………2 → (03)<br>PRESCRIPTION ONLY……….3 → (03)	DAYS… [__][__][__]<br>CONSTANT PROBLEM…995<br>DON'T KNOW..998		
(03) Injectables	PROVIDE & COUNSEL…………….1<br>COUNSEL, NOT PROVIDE…..2<br>KNOW LITTLE ABOUT……….3<br>DO NOT KNOW……………….8<br>(2, 3, 8) → (04)	YES……..1<br>NO………2 → (04)	YES………..1<br>NO…………2 → (04)<br>PRESCRIPTION ONLY……….3 → (04)	DAYS… [__][__][__]<br>CONSTANT PROBLEM…995<br>DON'T KNOW..998		

Now I would like to ask you some questions about your knowledge and provision of various methods of family planning. If you have provided a particular method before, we are also interested in the availability and quality of the materials required to provide that method.

METHOD	13a. Can you please tell me which of the following best describes your knowledge of [METHOD]:<br>1. You know METHOD sufficiently well to counsel and provide/assist in provision to a client;<br>2. You know METHOD sufficiently well to counsel, but not to provide;<br>3. You know little about METHOD and would not feel comfortable counseling or providing;<br>8. You know do not know METHOD at all	13b. Have you provided (assisted with) [METHOD] to clients at this facility?	13c. Have you experienced any stockouts in this facility that lasted more than 24 hours of [METHOD] in the last one year?	13d. If yes, how many total days of stockouts did this facility have in the last ONE YEAR of [METHOD] (all stockouts combined)?	13e. Have you experienced a lack of essential equipment needed to provide [METHOD] in the last ONE YEAR?	13f. If Yes, how many total days did you lack essential equipment needed to provide [METHOD] in the last ONE YEAR?
(04) Male condom	PROVIDE & COUNSEL……………1<br>COUNSEL, NOT PROVIDE…..2<br>KNOW LITTLE ABOUT……….3 } **(05)**<br>DO NOT KNOW………………8	YES……..1<br>NO………2→ **(05)**	YES………..1<br>NO…………2→ **(05)**<br>PRESCRIPTION ONLY……….3→ **(05)**	DAYS…<br>[ ][ ][ ]<br>CONSTANT PROBLEM…995<br>DON'T KNOW..998		
(05) Female condom	PROVIDE & COUNSEL……………1<br>COUNSEL, NOT PROVIDE…..2<br>KNOW LITTLE ABOUT……….3 } **(06)**<br>DO NOT KNOW………………8	YES……..1<br>NO………2→ **(06)**	YES………..1<br>NO…………2→ **(06)**<br>PRESCRIPTION ONLY……….3→ **(06)**	DAYS…<br>[ ][ ][ ]<br>CONSTANT PROBLEM…995<br>DON'T KNOW..998		
(06) Emergency contraception	PROVIDE & COUNSEL……………1<br>COUNSEL, NOT PROVIDE…..2<br>KNOW LITTLE ABOUT……….3 } **(07)**<br>DO NOT KNOW………………8	YES……..1<br>NO………2→ **(07)**	YES………..1<br>NO…………2→ **(07)**<br>PRESCRIPTION ONLY……….3→ **(07)**	DAYS…<br>[ ][ ][ ]<br>CONSTANT PROBLEM…995<br>DON'T KNOW..998		

QUESTIONNAIRE IDENTIFICATION NO:[__|__|__|__|__|__|__|__]
5 digit facility code + 3 digit prov code

Now I would like to ask you some questions about your knowledge and provision of various methods of family planning. If you have provided a particular method before, we are also interested in the availability and quality of the materials required to provide that method.

METHOD	13a. Can you please tell me which of the following best describes your knowledge of [METHOD]:<br>1. You know METHOD sufficiently well to counsel and provide/assist in provision to a client;<br>2. You know METHOD sufficiently well to counsel, but not to provide;<br>3. You know little about METHOD and would not feel comfortable counseling or providing;<br>8. You know do not know METHOD at all	13b. Have you provided (assisted with) [METHOD] to clients at this facility?	13c. Have you experienced any stockouts in this facility that lasted more than 24 hours of [METHOD] in the last one year?	13d. If yes, how many total days of stockouts did this facility have in the last ONE YEAR of [METHOD] (all stockouts combined)?	13e. Have you experienced a lack of essential equipment needed to provide [METHOD] in the last ONE YEAR?	13f. If Yes, how many total days did you lack essential equipment needed to provide [METHOD] in the last ONE YEAR?
(07) Spermicide	PROVIDE & COUNSEL…………….1<br>COUNSEL, NOT PROVIDE…..2<br>KNOW LITTLE ABOUT……….3 } **(08)**<br>DO NOT KNOW………………8	YES……..1<br>NO………2➔ **(08)**	YES………..1<br>NO…………2➔ **(08)**<br>PRESCRIPTION ONLY……….3➔ **(08)**	DAYS…<br>[ ][ ][ ]<br>CONSTANT PROBLEM…995<br>DON'T KNOW..998		
(08) Diaphragm	PROVIDE & COUNSEL…………….1<br>COUNSEL, NOT PROVIDE…..2<br>KNOW LITTLE ABOUT……….3 } **(09)**<br>DO NOT KNOW………………8	YES……..1<br>NO………2➔ **(09)**	YES………..1<br>NO…………2➔ **(09)**<br>PRESCRIPTION ONLY……….3➔ **(09)**	DAYS…<br>[ ][ ][ ]<br>CONSTANT PROBLEM…995<br>DON'T KNOW..998		
(09) IUD	PROVIDE & COUNSEL…………….1<br>COUNSEL, NOT PROVIDE…..2<br>KNOW LITTLE ABOUT……….3 } **(10)**<br>DO NOT KNOW………………8	YES……..1<br>NO………2➔ **(10)**	YES………..1<br>NO…………2➔ **(10)**<br>PRESCRIPTION ONLY……….3➔ **(10)**	DAYS…<br>[ ][ ][ ]<br>CONSTANT PROBLEM…995<br>DON'T KNOW..998	YES……..1<br>NO………2➔ **(10)**	DAYS…<br>[ ][ ][ ]<br>CONSTANT PROBLEM…995<br>DK…...998

Now I would like to ask you some questions about your knowledge and provision of various methods of family planning. If you have provided a particular method before, we are also interested in the availability and quality of the materials required to provide that method.

METHOD	13a. Can you please tell me which of the following best describes your knowledge of [METHOD]:<br>1. You know METHOD sufficiently well to counsel and provide/assist in provision to a client;<br>2. You know METHOD sufficiently well to counsel, but not to provide;<br>3. You know little about METHOD and would not feel comfortable counseling or providing;<br>8. You know do not know METHOD at all	13b. Have you provided (assisted with) [METHOD] to clients at this facility?	13c. Have you experienced any stockouts in this facility that lasted more than 24 hours of [METHOD] in the last one year?	13d. If yes, how many total days of stockouts did this facility have in the last ONE YEAR of [METHOD] (all stockouts combined)?	13e. Have you experienced a lack of essential equipment needed to provide [METHOD] in the last ONE YEAR?	13f. If Yes, how many total days did you lack essential equipment needed to provide [METHOD] in the last ONE YEAR?
(10) Implants	PROVIDE & COUNSEL……………1<br>COUNSEL, NOT PROVIDE…..2<br>KNOW LITTLE ABOUT……….3 → (11)<br>DO NOT KNOW………………8	YES……..1<br>NO………2→ (11)	YES………..1<br>NO…………2→ (11)<br>PRESCRIPTION ONLY……….3→ (11)	DAYS… [| |]<br>CONSTANT PROBLEM…995<br>DON'T KNOW..998	YES……..1<br>NO………2→ (11)	DAYS… [| |]<br>CONSTANT PROBLEM…995<br>DK…...998
(11) Female sterilization	PROVIDE & COUNSEL……………1<br>COUNSEL, NOT PROVIDE…..2<br>KNOW LITTLE ABOUT……….3 → (12)<br>DO NOT KNOW………………8	YES……..1<br>NO………2→ (12)			YES……..1<br>NO………2→ (12)	DAYS… [| |]<br>CONSTANT PROBLEM…995<br>DK…...998
(12) Male sterilization	PROVIDE & COUNSEL……………1<br>COUNSEL, NOT PROVIDE…..2<br>KNOW LITTLE ABOUT……….3 → (13)<br>DO NOT KNOW………………8	YES……..1<br>NO………2→ (13)			YES……..1<br>NO………2→ (13)	DAYS… [| |]<br>CONSTANT PROBLEM…995<br>DK…...998

	13a. Can you please tell me which of the following best describes your knowledge of [METHOD]:<br>1. Know the method sufficiently well to counsel and recommend to client<br>2. Know little about the method and would not feel comfortable counseling or recommending<br>8. Do not know method	13b. Have you ever recommended [METHOD] to clients at this facility?
(13) Natural methods (Rhythm, periodic abstinence, withdrawal, cycle beads)	COUNSEL & RECOMMEND……………..…1<br>KNOW LITTLE ABOUT……………..2<br>DO NOT KNOW……………………...8 → (14)	Yes……..1<br>No………2
(14) Exclusive breastfeeding method (LAM)	COUNSEL & RECOMMEND……………..…1<br>KNOW LITTLE ABOUT……………..2<br>DO NOT KNOW……………………..8 → Q14	Yes……..1<br>No………2

Q14. **CHECK Q13A:**

PROVIDES AND/OR COUNSELS ANY FP METHOD (ANY Q13A = 1 OR 2) ☐ ↓

DOES NOT PROVIDE AND DOES NOT COUNSEL ANY FP METHOD (ALL Q13A = 3 OR 4) ☐ → **Q20**

QUESTIONNAIRE IDENTIFICATION NO:[__|__|__|__|__|__|__|__]
5 digit facility code + 3 digit prov code

Now I would like to ask you specifically about the contraceptive methods that you provide.
(ASK ONLY ABOUT THE FAMILY PLANNING METHODS THE RESPONDENT IS PROVIDING – Q13b)

METHOD	Q15a. What is the minimum age that you would offer this [METHOD]?	Q15b. What is the maximum age that you would offer this [METHOD]?	Q15c. Is there a minimum number of children a person must have before you will offer [METHOD]?	Q15d. What is that minimum number of children?	Q15e. Do you require a partner's consent before you will provide [METHOD]?	Q15f. Would you offer METHOD to an unmarried person?
(1) Combined oral pills	NO MIN…...93<br>DK……….98	NO MAX…….93<br>DK……….98	YES . . .1<br>NO . . ...2 →**Q15e**<br>DK…….8→**Q15e**		YES . . . .1<br>NO 2	YES . . . .1<br>NO 2
(2) Progestin-only pill	NO MIN…...93<br>DK……….98	NO MAX…….93<br>DK……….98	YES . . .1<br>NO . . ...2 →**Q15e**<br>DK…….8→**Q15e**		YES . . . .1<br>NO 2	YES . . . .1<br>NO 2
(3) Male condom	NO MIN…...93<br>DK……….98	NO MAX……..93<br>DK……….98	YES . . .1<br>NO . . ...2 →**Q15e**<br>DK…….8→**Q15e**		YES . . . .1<br>NO 2	YES . . . .1<br>NO 2
(4) Female condom	NO MIN…...93<br>DK……….98	NO MAX…......93<br>DK……….98	YES . . .1<br>NO . . ...2 →**Q15e**<br>DK…….8→**Q15e**		YES . . . .1<br>NO 2	YES . . . .1<br>NO 2
(5) IUD	NO MIN…...93<br>DK……….98	NO MAX.........93<br>DK……….98	YES . . .1<br>NO . . ...2 →**Q15e**<br>DK…….8→**Q15e**		YES . . . .1<br>NO 2	YES . . . .1<br>NO 2
(6) Spermicide	NO MIN…...93<br>DK……….98	NO MAX…….93<br>DK……….98	YES . . .1<br>NO . . ...2 →**Q15e**<br>DK…….8→**Q15e**		YES . . . .1<br>NO 2	YES . . . .1<br>NO 2
(7) Diaphragm	NO MIN…...93<br>DK……….98	NO MAX…….93<br>DK……….98	YES . . .1<br>NO . . ...2 →**Q15e**<br>DK…….8→**Q15e**		YES . . . .1<br>NO 2	YES . . . .1<br>NO 2
(8) Injectables	NO MIN…...93<br>DK……….98	NO MAX…….93<br>DK……….98	YES . . .1<br>NO . . ...2 →**Q15e**<br>DK…….8→**Q15e**		YES . . . .1<br>NO 2	YES . . . .1<br>NO 2
(9) Implants	NO MIN…...93<br>DK……….98	NO MAX……..93<br>DK……….98	YES . . .1<br>NO . . ...2 →**Q15e**<br>DK…….8→**Q15e**		YES . . . .1<br>NO 2	YES . . . .1<br>NO 2
(10) Male sterilization	NO MIN…...93<br>DK……….98	NO MAX...93<br>DK……….98	YES . . .1<br>NO . . ...2 →**Q15e**<br>DK…….8→**Q15e**		YES . . . .1<br>NO 2	YES . . . .1<br>NO 2
(11) Female sterilization	NO MIN…….93<br>DK……….98	NO MAX...93<br>DK……….98	YES . . .1<br>NO . . ...2 →**Q15e**<br>DK…….8→**Q15e**		YES . . . .1<br>NO 2	YES . . . .1<br>NO 2
(12) Emergency contraceptive	NO MIN….93<br>DK……….98	NO MAX...93<br>DK……….98	YES . . .1<br>NO . . ...2 →**Q15e**<br>DK…….8→**Q15e**		YES . . . .1<br>NO 2	YES . . . .1<br>NO 2

Q16.	What do you do/tell the client when talking about FP to clients?<br><br>PROBE – Anything else?<br>**MULTIPLE RESPONSES POSSIBLE.**<br>**CIRCLE ALL MENTIONED.**	IDENTIFY REPRODUCTIVE GOALS OF CLIENT……A<br>PROVIDE INFORMATION ABOUT DIFFERENT FP METHODS……………………………………………B<br>DISCUSS THE CLIENT'S FP PREFERENCES……....C<br>HELP CLIENT SELECT A SUITABLE METHOD……..D<br>EXPLAIN THE WAY TO USE THE SELECTED METHOD……………………………………………E<br>EXPLAIN THE SIDE-EFFECTS……………………….F<br>EXPLAIN SPECIFIC MEDICAL REASONS TO RETURN……………………………………………G<br>REQUEST FOR PARTNER'S CONSENT…………….H<br><br>OTHERS________________________________ X<br>(SPECIFY)	
Q17.	**CHECK Q13B:**<br><br>PROVIDES HORMONAL METHODS (PILL OF ANY TYPE, IUD, INJECTABLE, OR IMPLANTS: Q13B(1)=1 OR Q13B(2)=1 OR Q13B(3)=1 OR Q13B(9)=1 OR Q13B(10)=1) ☐ ↓	DOES NOT PROVIDE HORMONAL METHODS (ALL OF THE FOLLOWING EQUAL "2" OR ARE SKIPPED: Q13B(1), Q13B(2), Q13B(3), Q13B(9), Q13B(10)) ☐ →	**Q19**
Q18.	What do you do for a new client who wants the pill or another hormonal method but is not having her menses?<br><br>**DO NOT READ OPTIONS**<br><br>PROBE WITH "Anything else?"<br><br>**MULTIPLE RESPONSES POSSIBLE.**<br>**CIRCLE ALL MENTIONED.**	QUESTION TO EXCLUDE PREGNANCY………….A<br>EXAMINE TO EXCLUDE PREGNANCY……………B<br>TEST TO EXCLUDE PREGNANCY…………………C<br>TELL HER TO COME BACK AT NEXT MENSES….D<br>TRY TO INDUCE MENSES………………………….E<br>SUPPLY CONDOMS UNTIL NEXT MENSES………F<br>SUPPLY HORMONAL METHOD IF REASONABLY CERTAIN SHE IS NOT PREGNANT…………….G<br>SUPPLY HORMONAL METHOD AND CONDOMS, ASK HER TO USE CONDOMS UNTIL NEXT MENSES……………………………………………H<br>JUST GIVE HORMONAL METHOD……………….. J<br>REQUEST FOR PARTNER'S CONSENT………….K<br>OTHER________________________________ X<br>(SPECIFY)	
Q19.	Which kind of personal and financial records do you complete each time you provide a client with family planning services?<br><br>**MULTIPLE RESPONSES POSSIBLE.**<br>**CIRCLE ALL MENTIONED.**	NO RECORD KEPT…………………………………..Y<br>A CLIENT RECORD CARD/FORM………………...A<br>AN ENTRY IN THE FP REGISTER…...................B<br>AN ENTRY IN THE FACILITY LOGBOOK/ REGISTER……………………………………….C<br>INFORMAL NOTES IN A NOTEBOOK……………..D<br>A PAYMENT RECEIPT IF A FEE IS INVOLVED…..E<br>OTHER ______________________________X<br>(SPECIFY)	

**INTEGRATION OF FAMILY PLANNING WITH OTHER SERVICES**			
Q20.	Which are the other services that you yourself provide to clients at this health facility? READ THE OPTIONS.<br><br>**MULTIPLE RESPONSES POSSIBLE.**<br>**CIRCLE ALL MENTIONED.**	ANTE-NATAL CARE………………………………A<br>DELIVERY SERVICES……………………………..B<br>POST-NATAL CARE……...................................C<br>POST-ABORTION CARE………………………….D<br>CHILD IMMUNIZATION……………………………E<br>CHILD GROWTH MONITORING………………….F<br>OTHER CURATIVE SERVICES FOR WOMEN………G<br>OTHER CURATIVE SERVICES FOR CHILDREN…..H<br>HIV/AIDS MANAGEMENT…………………………I<br>PMTCT……………………………………………..J<br>VCT………………………………………………..K<br>NONE OF THESE…………………………………..Y →	**Q62**
Q21.	**CHECK Q20:**<br><br>IF OPTION A (ANTENATAL CARE) IS CIRCLED ☐ ↓	IF OPTION A (ANTENATAL CARE) IS **NOT** CIRCLED ☐ →	**Q27**
Q22.	During Antenatal care, do you provide information about FP routinely?	YES...1<br>NO...2 →	Q25

Q23.	What do you do/tell the client when talking about FP during antenatal care?<br><br>PROBE: "ANYTHING ELSE?"<br>**MULTIPLE RESPONSES POSSIBLE.**<br>**CIRCLE ALL MENTIONED.**	HELP THE WOMAN SELECT A SUITABLE METHOD FOR POST-DELIVERY……………A<br>INFORM ABOUT THE IMPORTANCE OF USING FP BY 40 DAYS POSTPARTUM……………B<br>PROVIDE INFORMATION ON LAM……………C<br>EXPLAIN SIDE-EFFECTS……………D<br>ENCOURAGE WOMEN TO WAIT FOR SOME TIME BEFORE THE NEXT PREGNANCY……………E<br>REQUEST FOR PARTNER'S CONSENT……………F<br>OTHERS: ______________ X<br>(SPECIFY)	
Q24.	Do you tell women where they can obtain an FP method after delivery?	YES……………1<br>NO……………2	**All skip to Q27**
Q25.	Why are you not able to provide FP information routinely during antenatal care visits?<br><br>**MULTIPLE RESPONSES POSSIBLE.**<br>**CIRCLE ALL MENTIONED.**	ADEQUATE CONTRACEPTIVE METHODS FREQUENTLY UNAVAILABLE……………A<br>AVAILABLE CONTRACEPTIVES OFTEN PAST EXPIRATION DATE……………B<br>LACK OF STERILE EQUIPMENT SO NO POINT DISCUSSING……………C<br>LACK OF FUNCTIONAL EQUIPMENT SO NO POINT DISCUSSING……………D<br>NO INTEREST IN PROVIDING FP INFORMATION……………E<br>LACK KNOWLEDGE ABOUT FP……………F<br>DO NOT FEEL ADEQUATELY TRAINED TO PROVIDE FP INFORMATION……………G<br>NO INTEREST IN FP ON THE PART OF THE PATIENTS…H<br>OVERLOAD OF WORK/NO TIME TO DISCUSS……………I<br>NO NEED TO……………K<br>NOT A PROFITABLE SERVICE TO PROVIDE……………L<br><br>OTHERS______________X<br>(SPECIFY)	
Q26.	Would you be willing to include family planning information routinely in your antenatal care services/visits?	YES……………1<br>NO……………2	
Q27.	**CHECK Q20:**<br><br>IF OPTION B (DELIVERY CARE) IS CIRCLED ☐ ↓	IF OPTION B (DELIVERY CARE) IS **<u>NOT</u>** CIRCLED ☐ →	**Q33**
Q28.	During <u>delivery care</u> (anytime before they are discharged from your facility), do you provide information about FP routinely?	YES……………1<br>NO……………2 →	**Q31**
Q29.	What do you do/tell the client when talking about FP during delivery care?<br><br>PROBE: "ANYTHING ELSE?"<br>**MULTIPLE RESPONSES POSSIBLE.**<br>**CIRCLE ALL MENTIONED.**	HELP SELECT SUITABLE FP METHOD BY 40 DAYS POSTPARTUM……………A<br>PROVIDE INFORMATION ON LAM……………B<br>EXPLAIN SIDE-EFFECTS……………C<br>EXPLAIN SPECIFIC MEDICAL REASONS TO RETURN……………D<br>ENCOURAGE WOMEN TO WAIT SOME TIME BEFORE THE NEXT PREGNANCY……………E<br>REQUEST FOR PARTNER'S CONSENT……………F<br>OTHER______________ X<br>(SPECIFY)	
Q30.	Do you tell women where they can obtain an FP method during delivery care?	YES……………1<br>NO……………2	**All skip to Q33**

Q31.	Why are you not able to provide FP information routinely during delivery care?<br><br>PROBE: "ANYTHING ELSE?"<br>**MULTIPLE RESPONSES POSSIBLE. CIRCLE ALL MENTIONED.**	ADEQUATE CONTRACEPTIVE METHODS FREQUENTLY UNAVAILABLE……A<br>AVAILABLE CONTRACEPTIVES OFTEN PAST EXPIRATION DATE……B<br>LACK OF STERILE EQUIPMENT SO NO POINT DISCUSSING……C<br>LACK OF FUNCTIONAL EQUIPMENT SO NO POINT DISCUSSING……D<br>NO INTEREST IN PROVIDING FP INFORMATION……E<br>LACK KNOWLEDGE ABOUT FP……F<br>DO NOT FEEL ADEQUATELY TRAINED TO PROVIDE FP INFORMATION……G<br>NO INTEREST IN FP ON THE PART OF THE PATIENTS…H<br>OVERLOAD OF WORK/NO TIME TO DISCUSS……I<br>NO NEED TO……K<br>NOT A PROFITABLE SERVICE TO PROVIDE……L<br><br>OTHERS________X<br>(SPECIFY)	
Q32.	Would you be willing to include family planning information routinely in your delivery care services?	YES……1<br>NO……2	
Q33.	**CHECK Q20:**<br>IF OPTION C (POST-NATAL CARE) IS CIRCLED ☐ ↓	IF OPTION C (POST-NATAL CARE) IS **<u>NOT</u>** CIRCLED ☐ →	**Q39**
Q34.	During <u>post-natal care</u> visits, do you provide information about FP routinely?	YES……1<br>NO……2 →	**Q37**
Q35.	What do you do/tell the client when talking about FP during post-natal care visits?<br><br>PROBE: "ANYTHING ELSE?"<br>**MULTIPLE RESPONSES POSSIBLE. CIRCLE ALL MENTIONED.**	HELP SELECT SUITABLE FP METHOD BY 40 DAYS POSTPARTUM……A<br>PROVIDE INFORMATION ON LAM……B<br>EXPLAIN SIDE-EFFECTS……C<br>EXPLAIN SPECIFIC MEDICAL REASONS TO RETURN……D<br>ENCOURAGE WOMEN TO WAIT SOME TIME BEFORE THE NEXT PREGNANCY……E<br>REQUEST FOR PARTNER'S CONSENT……F<br>OTHER________X<br>(SPECIFY)	
Q36.	Do you tell women where they can obtain an FP method during post-natal care visits?	YES……1 }<br>NO……2 } →	**All skip to Q39**
Q37.	Why are you not able to provide FP information routinely during post-natal care visits?<br><br>PROBE: "ANYTHING ELSE?"<br>**MULTIPLE RESPONSES POSSIBLE. CIRCLE ALL MENTIONED.**	ADEQUATE CONTRACEPTIVE METHODS FREQUENTLY UNAVAILABLE……A<br>AVAILABLE CONTRACEPTIVES OFTEN PAST EXPIRATION DATE……B<br>LACK OF STERILE EQUIPMENT SO NO POINT DISCUSSING……C<br>LACK OF FUNCTIONAL EQUIPMENT SO NO POINT DISCUSSING……D<br>NO INTEREST IN PROVIDING FP INFORMATION……E<br>LACK KNOWLEDGE ABOUT FP……F<br>DO NOT FEEL ADEQUATELY TRAINED TO PROVIDE FP INFORMATION……G<br>NO INTEREST IN FP ON THE PART OF THE PATIENTS…H<br>OVERLOAD OF WORK/NO TIME TO DISCUSS……I<br>NO NEED TO……K<br>NOT A PROFITABLE SERVICE TO PROVIDE……L<br><br>OTHERS________X<br>(SPECIFY)	
Q38.	Would you be willing to include family planning information routinely in your delivery care services?	YES……1<br>NO……2	
Q39.	**CHECK Q20:**<br><br>IF OPTION D (POST-ABORTION CARE) IS CIRCLED ☐ ↓	IF OPTION D (POST-ABORTION CARE) IS **NOT** CIRCLED ☐ →	**Q45**

Q40.	During a post abortion care, do you provide information about FP routinely?	YES......1<br>NO......2 →	Q43
Q41.	What do/tell the client when talking about FP during post abortion care visits?<br><br>PROBE: "ANYTHING ELSE?"<br>**MULTIPLE RESPONSES POSSIBLE.**<br>**CIRCLE ALL MENTIONED.**	IDENTIFY REPRODUCTIVE GOALS OF WOMAN......A<br>PROVIDE INFORMATION ABOUT DIFFERENT FP METHODS......B<br>DISCUSS THE CLIENT'S FP PREFERENCES......C<br>HELP WOMEN SELECT A SUITABLE METHOD......D<br>EDUCATE WOMEN TO USE THE SELECTED METHOD......E<br>INFORM ABOUT HOW SOON AFTER ABORTION SHE MAY BECOME PREGNANT IF NOT USING CONTRACEPTION......F<br>EXPLAIN SIDE-EFFECTS......G<br>EXPLAIN SPECIFIC MEDICAL REASONS TO RETURN......H<br>REQUEST FOR PARTNER'S CONSENT......I<br>OTHERS: ______________ X<br>(SPECIFY)	
Q42.	Do you tell women where they can obtain an FP method during post abortion care visits?	YES......1<br>NO......2 } →	**All skip to Q45**
Q43.	Why are you not able to provide FP information routinely during post abortion care visits?<br><br>PROBE: "ANYTHING ELSE?"<br>**MULTIPLE RESPONSES POSSIBLE.**<br>**CIRCLE ALL MENTIONED.**	ADEQUATE CONTRACEPTIVE METHODS FREQUENTLY UNAVAILABLE......A<br>AVAILABLE CONTRACEPTIVES OFTEN PAST EXPIRATION DATE......B<br>LACK OF STERILE EQUIPMENT SO NO POINT DISCUSSING......C<br>LACK OF FUNCTIONAL EQUIPMENT SO NO POINT DISCUSSING......D<br>NO INTEREST IN PROVIDING FP INFORMATION......E<br>LACK KNOWLEDGE ABOUT FP......F<br>DO NOT FEEL ADEQUATELY TRAINED TO PROVIDE FP INFORMATION......G<br>NO INTEREST IN FP ON THE PART OF THE PATIENTS......H<br>OVERLOAD OF WORK/NO TIME TO DISCUSS......I<br>NO NEED TO......K<br>NOT A PROFITABLE SERVICE TO PROVIDE......L<br>OTHERS______________X<br>(SPECIFY)	
Q44.	Would you be willing to include family planning information routinely in your post abortion care services/visits?	YES......1<br>NO......2	
Q45.	**CHECK Q20:**<br><br>IF **EITHER** OPTION E (CHILD IMMUNIZATION) OR OPTION F (CHILD GROWTH MONITORING) IS CIRCLED ☐ ↓	IF **NEITHER** OPTION E (CHILD IMMUNIZATION) **NOR** OPTION F (CHILD GROWTH MONITORING) IS CIRCLED ☐ →	Q51
Q46.	During child immunization/child growth monitoring, do you provide information about FP routinely?	YES......1<br>NO......2 →	**Q49**
Q47.	What do you do/tell clients when talking about FP during child immunization or child growth monitoring visits?<br><br>PROBE: "ANYTHING ELSE?"<br>**MULTIPLE RESPONSES POSSIBLE.**<br>**CIRCLE ALL MENTIONED.**	IDENTIFY REPRODUCTIVE GOALS OF WOMAN......A<br>PROVIDE INFORMATION ABOUT DIFFERENT FP METHODS......B<br>DISCUSS THE CLIENT'S FP PREFERENCES......C<br>HELP WOMEN SELECT A SUITABLE METHOD......D<br>EDUCATE WOMEN TO USE THE SELECTED METHOD......E<br>EXPLAIN SIDE-EFFECTS......F<br>EXPLAIN SPECIFIC MEDICAL REASONS TO RETURN......G<br>REQUEST FOR PARTNER'S CONSENT......H<br>OTHERS: ______________ X<br>(SPECIFY)	
Q48.	Do you tell women where they can obtain an FP method?	Yes......1<br>No......2 } →	**All skip to Q51**

Q49.	Why are you not able to provide FP information routinely?<br><br>PROBE: "ANYTHING ELSE?"<br>**MULTIPLE RESPONSES POSSIBLE.**<br>**CIRCLE ALL MENTIONED.**	ADEQUATE CONTRACEPTIVE METHODS FREQUENTLY UNAVAILABLE……… ……..A<br>AVAILABLE CONTRACEPTIVES OFTEN PAST EXPIRATION DATE……………………… …...B<br>LACK OF STERILE EQUIPMENT SO NO POINT DISCUSSING……………………………………..C<br>LACK OF FUNCTIONAL EQUIPMENT SO NO POINT DISCUSSING………………………..D<br>NO INTEREST IN PROVIDING FP INFORMATION…………………………………….E<br>LACK KNOWLEDGE ABOUT FP……………………F<br>DO NOT FEEL ADEQUATELY TRAINED TO PROVIDE FP INFORMATION…………………...G<br>NO INTEREST IN FP ON THE PART OF THE PATIENTS…………………………………….......H<br>OVERLOAD OF WORK/NO TIME…………………....I<br>NO NEED TO…………………………………………K<br>NOT A PROFITABLE SERVICE TO PROVIDE…….L<br><br>OTHERS________________________________X<br>(SPECIFY)	
Q50.	Would you be willing to include family planning information routinely in your child immunization or child growth monitoring visits?	YES...1<br>NO..2	
Q51.	**CHECK Q20**:<br><br>IF **EITHER** OPTION G (CURATIVE SERVICES FOR WOMEN) OR H (CURATIVE SERVICES FOR CHILDREN) IS CIRCLED ☐ ↓	IF **NEITHER** OPTION G (CURATIVE SERVICES FOR WOMEN) **NOR** H (CURATIVE SERVICES FOR CHILDREN) IS CIRCLED ☐ →	**Q57**
Q52.	While providing curative services to women or children, do you provide information on FP routinely?	YES...1<br>NO..2 →	**Q55**
Q53.	What are the main activities you follow when talking about FP to clients?<br><br>PROBE: "ANYTHING ELSE?"<br>**MULTIPLE RESPONSES POSSIBLE.**<br>**CIRCLE ALL MENTIONED.**	IDENTIFY REPRODUCTIVE GOALS OF WOMAN...A<br>PROVIDE INFORMATION ABOUT DIFFERENT FP METHODS……………………………………B<br>DISCUSS THE CLIENT'S FP PREFERENCES….C<br>HELP WOMEN SELECT A SUITABLE METHOD…………………………………………..D<br>EDUCATE WOMEN TO USE THE SELECTED METHOD……………………………………….E<br>EXPLAIN SIDE-EFFECTS………………………….F<br>EXPLAIN SPECIFIC MEDICAL REASONS TO RETURN…………………………………….…G<br>OTHERS: ______________________________ X<br>(SPECIFY)	
Q54.	Do you tell women where they can obtain an FP method?	YES..1<br>NO...2 } →	**All skip to Q57**
Q55.	Why are you not able to provide FP information routinely?<br><br>PROBE: "ANYTHING ELSE?"<br>**MULTIPLE RESPONSES POSSIBLE.**<br>**CIRCLE ALL MENTIONED.**	ADEQUATE CONTRACEPTIVE METHODS FREQUENTLY UNAVAILABLE……… ……..A<br>AVAILABLE CONTRACEPTIVES OFTEN PAST EXPIRATION DATE……………………… …...B<br>LACK OF STERILE EQUIPMENT SO NO POINT DISCUSSING……………………………………..C<br>LACK OF FUNCTIONAL EQUIPMENT SO NO POINT DISCUSSING………………………..D<br>NO INTEREST IN PROVIDING FP INFORMATION…………………………………….E<br>LACK KNOWLEDGE ABOUT FP……………………F<br>DO NOT FEEL ADEQUATELY TRAINED TO PROVIDE FP INFORMATION…………………...G<br>NO INTEREST IN FP ON THE PART OF THE PATIENTS…………………………………….......H<br>OVERLOAD OF WORK/NO TIME…………………....I<br>NO NEED TO…………………………………………K<br>NOT A PROFITABLE SERVICE TO PROVIDE…….L<br><br>OTHERS________________________________X<br>(SPECIFY)	

Q56.	Would you be willing to include family planning information routinely in your curative care services/visits for women or children?	YES....1<br>NO....2	
Q57.	**CHECK Q20:**<br><br>IF **ANY** OF THE OPTIONS I (HIV/AIDS MANAGEMENT), OPTION J (PMTCT), OR K (VCT) ARE CIRCLED ☐ ↓	IF **NONE** OF THE OPTIONS I (HIV/AIDS MANAGEMENT), OPTION J (PMTCT), OR K (VCT) ARE CIRCLED ☐ →	**Q63**
Q58.	While providing HIV-related services (HIV/AIDS management, PMTCT, and/or VCT) to women and men, do you provide information on FP routinely?	YES....1<br>NO....2 →	**Q61**
Q59.	What are the main activities you follow when talking about FP to clients?<br><br>PROBE: "ANYTHING ELSE?"<br>**MULTIPLE RESPONSES POSSIBLE.**<br>**CIRCLE ALL MENTIONED.**	IDENTIFY REPRODUCTIVE GOALS OF WOMAN...A<br>PROVIDE INFORMATION ABOUT DIFFERENT FP METHODS....B<br>DISCUSS THE CLIENT'S FP PREFERENCES....C<br>HELP WOMEN SELECT A SUITABLE METHOD....D<br>EDUCATE WOMEN TO USE THE SELECTED METHOD....E<br>EXPLAIN SIDE-EFFECTS....F<br>EXPLAIN SPECIFIC MEDICAL REASONS TO RETURN....G<br>DISCUSS HIV/AIDS PREVENTION METHODS....H<br>DISCUSS METHODS NOT RECOMMENDED FOR HIV POSITIVE (LAM, IUD)....I<br>RECOMMEND ALWAYS USE CONDOM IN ADDITION TO OTHER FP METHODS....J<br>REQUEST FOR PARTNER'S CONSENT....K<br>OTHERS: ______________ X<br>(SPECIFY)	
Q60.	Do you tell women where they can obtain an FP method?	YES....1<br>NO....2 } →	**All skip to Q63**
Q61.	Why are you not able to provide FP information routinely?<br><br>PROBE: "ANYTHING ELSE?"<br>**MULTIPLE RESPONSES POSSIBLE.**<br>**CIRCLE ALL MENTIONED.**<br>.	ADEQUATE CONTRACEPTIVE METHODS FREQUENTLY UNAVAILABLE.........A<br>AVAILABLE CONTRACEPTIVES OFTEN PAST EXPIRATION DATE.......B<br>LACK OF STERILE EQUIPMENT SO NO POINT DISCUSSING....C<br>LACK OF FUNCTIONAL EQUIPMENT SO NO POINT DISCUSSING....D<br>NO INTEREST IN PROVIDING FP INFORMATION....E<br>LACK KNOWLEDGE ABOUT FP....F<br>DO NOT FEEL ADEQUATELY TRAINED TO PROVIDE FP INFORMATION....G<br>NO INTEREST IN FP ON THE PART OF THE PATIENTS....H<br>OVERLOAD OF WORK/NO TIME....I<br>NO NEED TO....K<br>NOT A PROFITABLE SERVICE TO PROVIDE....L<br><br>OTHERS______________X<br>(SPECIFY)	
Q62.	Would you be willing to include family planning information routinely in your HIV-related services/visits for women and men?	YES....1<br>NO....2	

Q63.	Is this facility linked with another organization that provides family planning methods and materials at a discounted rate or for free (for example PPFN or SFH)?	YES..........1<br>NO..........2 →<br>DON'T KNOW..........8 →	 **Q65**<br>**Q65**
Q64a.	What is the name of the organization?	Q64b. What year did this facility begin to associate with each organization named?	
	1.	YEAR .... [ ][ ][ ][ ]<br>DON'T KNOW 9998	
	2.	YEAR .... [ ][ ][ ][ ]<br>DON'T KNOW 9998	
	3.	YEAR .... [ ][ ][ ][ ]<br>DON'T KNOW 9998	
	4.	YEAR .... [ ][ ][ ][ ]<br>DON'T KNOW 9998	
Q65.	RECORD THE TIME<br>IN 24 HOUR FORMAT	HOUR .... [ ][ ] MINUTES .... [ ][ ]	
Thank you very much for taking the time to answer my questions. Once again, any information you have given will be kept completely confidential. Have a good day!			
**COMMENTS:**			
