# Supplementary material for: Facility-level characteristics associated with family planning and child immunization services integration in urban areas of Nigeria: a longitudinal analysis
Source: BMC Public Health. 2021 Jul 12;21:1379. doi: 10.1186/s12889-021-11436-x (PMC8274034; doi:10.1186/s12889-021-11436-x)
Supplement: Supplementary file 3 — Additional file 3. Endline Health Facility Audit. [file 12889_2021_11436_MOESM3_ESM.pdf]

Measurement, Learning & Evaluation (MLE) Project
Health Facility audit – Nigeria - 2014

CITY NAME & CODE ____________________<br>(Abuja=1, Benin=2, Ibadan=3, Ilorin=4, Kaduna=5, Zaria=6)	[__]
LGA NAME & CODE____________________	[__|__|__]
LOCALITY NAME & CODE ____________________	[__|__|__|__]
FACILITY NAME AND CODE ____________________	[__|__|__|__|__|__]
FACILITY PHYSICAL ADDRESS____________________	

LOCATION OF FACILITY<br>GPS Reading<br>Altitude ……………………………………	[ ][ ][ ][ ]
Latitude	N [ ][ ] [ ][ ][ ][ ][ ]
Longitude	E [ ][ ][ ] [ ][ ][ ][ ][ ]

TYPE OF HEALTH FACILITY	PUBLIC SECTOR<br>GOVT. HOSPITAL……………………….. 11<br>WOMEN AND CHILDREN HOSPITAL 12<br>CHILD WELFARE CLINIC………….. 13<br>GOVT. HEALTH CENTRE……………. 14<br>GOVT. HEALTH POST/DISPENSARY 15<br>MATERNITY HOME…………………… 16<br>OTHER PUBLIC________________ 18<br>(SPECIFY)<br><br>PRIVATE SECTOR<br>PRIVATE HOSPITAL………….……… 21<br>PRIVATE CLINIC……………………… 22<br>PRIVATE DOCTOR'S OFFICE………. 23<br>NURSING/MATERNITY HOME……….. 24<br>OTHER PRIVATE________________ 29<br>(SPECIFY)<br>FBO<br>MISSION HOSPITAL………………… 31<br>FAITH-BASED HOME/HEALTH CENTRE……………….…………….. 32<br><br>OTHER<br>OTHER NGO HOSPITAL……………. 41<br>OTHER NGO CLINIC………………… 42<br>Other______________________96<br>(Specify)
HEALTH FACILITY VOLUME TYPE	HIGH VOLUME…………………………………..1<br>OTHER……………………………………….…2
FACILITY WAS INTERVIEWED AT BASELINE	YES………………………………..……………1<br>NO ………………………………..……………2

INTERVIEWER VISITS				
VISIT No.	1	2	3	FINAL VISIT
DATE<br><br>INTERVIEWER'S NAME<br>INTERVIEWER CODE<br>RESULT*	DAY/ MONTH/ YEAR<br>[____/_____/_14_]<br>________________<br>[ ][ ][ ]<br>[ ]	DAY/ MONTH/ YEAR<br>[____/_____/__14_]<br>________________<br>[ ][ ][ ]<br>[ ]	DAY/ MONTH/ YEAR<br>[____/_____/__14_]<br>________________<br>[ ][ ][ ]<br>[ ]	DAY [____|____]<br>MONTH [____|____]<br>YEAR [_2_|_0_|_1_|_4_]<br>____________________<br>[ ][ ][ ]<br>[ ]
**NEXT VISIT:**<br>**DATE**<br>**TIME**	[____/_____/14_]<br>[ ][ ] [ ][ ]<br>H H M M	[____/_____/14_]<br>[ ][ ] [ ][ ]<br>H H M M	[____/_____/14_]<br>[ ][ ] [ ][ ]<br>H H M M	TOTAL NO. OF VISITS [ ]

***RESULT CODES:**

1. COMPLETED
2. FACILITY MOVED OR IS DESTROYED
3. RESPONDENT NOT AVAILABLE (NOT AT WORK, ON STRIKE, ETC)
4. RESPONDENT REFUSED
5. PARTLY COMPLETED
6. POSTPONED
7. OTHER ________________________________ (Specify)

LANGUAGE

	ENGLISH	HAUSA	YORUBA	IGBO	PIDGIN	OTHER (SPECIFY)	TRANSLATOR USED?
LANGUAGE OF INTERVIEW	1	2	3	4	5	6____________	YES 1 / NO 2

POSITION OF MAIN PERSON INTERVIEWED - **CIRCLE ONE**

CLINIC MANAGER/FACILITY ADMINISTRATOR……………….….01
PHYSICIAN……………………..………………………….….…..02
NURSE…………………………………………….……………...03
MIDWIFE…………………………………………………….……04
NURSE/ MIDWIFE………………………………………….…..…05
CHEW / CHO………………………………………………….…06
OTHER _________ _________________________________96
(SPECIFY)
NAME____

MOBILE PHONE NUMBER______________________________

SEX OF MAIN PERSON INTERVIEWED

MALE…………………………………..1
FEMALE…………………………….…2

SUPERVISOR	OFFICE EDITOR	KEYED BY
NAME………………………………	NAME……………………………….	NAME………………………………………….
CODE: [ ][ ]	CODE: [ ][ ]	CODE: [ ][ ]
DATE [__ / ___/ 14_]<br>DD MM YY	DATE [__ / ___/ 14_]<br>DD MM YY	DATE [__/ __/ 14_]<br>DD MM YY

GENERAL FACILITY INFORMATION			
**Source**	**Questions**	**Coding**	**Skip/Notes**
Q1.	RECORD THE TIME<br>(IN 24 HOUR FORMAT) Hour ………….. [ ][ ] Minutes ………… [ ][ ]		
Q2.	In what year did this facility open?<br><br>**PROBE, IF RESPONDENT SAYS DON'T KNOW: THIS IS VERY IMPORTANT**. Can you tell me how old this facility is? For example, would you say it is about 3 years old? 7 years old? (etc.)<br><br>**FILL IN EITHER YEAR OPENED <u>OR</u> YEARS OLD.**	Year opened . . . . . . [ ][ ][ ][ ]<br>**<u>OR</u>**<br>Years old . . . . [ ][ ]<br>Don't know 9998	
Q3.	On average, how many days per week is the facility open?	Days per week. . . . . [ ]	
Q4.	What time does the facility typically open?<br><br>WRITE ANSWER ON 24-HOUR CLOCK (IE. IF OPENS AT 7:00 AM, MARK 07:00	[ ][ ] : [ ][ ]<br><br>Open 24 hours a day…………….99:91 →	**Q6a**
Q5.	What time does the facility typically close?<br><br>WRITE ANSWER ON 24-HOUR CLOCK (IE. IF CLOSES AT 7:00 PM, MARK 19:00	[ ][ ] : [ ][ ]	
Q6a.	Is this facility linked with PPFN or SFH or another organization that provides family planning methods and materials at a discounted rate or for free?	Yes…………………………………..……..1<br>No………………………………………….2 →<br>Don't know……………………………….8 →	 **Q7**<br>**Q7**
	6b. What is the name of this organization?	6c.What year did this facility begin to associate with each organization named?	
	1.	Year …………….. [ ][ ][ ][ ]<br><br>Don't know……………….9998	
	2.	Year …………….. [ ][ ][ ][ ]<br><br>Don't know……………….9998	
	3.	Year …………….. [ ][ ][ ][ ]<br><br>Don't know……………….9998	
	4	Year …………….. [ ][ ][ ][ ]<br><br>Don't know……………….9998	
Q7	Was there renovation done in this facility in the last five years?	Yes……………………………………..……..……..1<br>No…………………………………………..……..…2 →<br>Don't know……………………………….……...8 →	 Q9<br>Q9
Q8	What kinds of renovation?<br><br>MULTIPLE RESPONSES POSSIBLE	NURHI 72 hours makeover<br>(PLAQUE OBSERVED)………………… A<br>NURHI 72 hours makeover<br>(PLAQUE NOT OBSERVED)……..……B<br>CRUFFY……………………..……..……… C<br>Other<br>Renovation types ________________X<br>(Specify)	

Q9.	How many **permanent** staff of each type (cadre) does this facility have?<br><br>1. Obstetrician/Gynecologists (OB/GYN)<br>2. General surgeons<br>3. Pediatricians<br>4. General physicians<br>5. Theatre nurse<br>6. Nurse/Midwives<br>7. Nurses<br>8. Midwives<br>9. Community health extension workers (CHEWs)<br>10. Community health officers (CHO)<br>11. VCT Counsellor<br><br>**FOR LARGE MEDICAL HOSPITALS AND COLLEGES, PLEASE PROBE TO ESTIMATE TO YOUR BEST ABILITY.**<br>***NOTE: PERMANENT STAFF DOES NOT INCLUDE DOCTORS IN RESIDENCY TRAINING INTERNS OR NYSC**	OB/GYNS [___|___|___]<br>GENERAL SURGEONS [___|___|___]<br>PEDIATRICIANS [___|___|___]<br>GENERAL PHYSICIANS [___|___|___]<br>THEATRE NURSES [___|___|___]<br>NURSE/MIDWIVES [___|___|___]<br>NURSES [___|___|___]<br>MIDWIVES [___|___|___]<br>CHEW [___|___|___]<br>CHO [___|___|___]<br>VCT PROVIDER [___|___|___]	

Now I would like to ask you some questions about the permanent staff who work in this facility. We would like to ask their names, positions and departments, so that we can randomly sample a few to interview using a separate questionnaire. These few will then represent the group. Remember that this is for research purposes only and we will keep all details strictly confidential.

STAFF

LIST NAMES OF ALL PERMANENT STAFF INVOLVED IN PROVIDING REPRODUCTIVE HEALTH SERVICES, INCLUDING FAMILY PLANNING, MATERNAL AND CHILD HEALTH AND STI/VCT/HIV SERVICES. CODE "YES" IN Q10c FOR THOSE PROVIDERS ON DUTY TODAY AND "NO" FOR THOSE NOT ON DUTY AT ANY TIME TODAY.
FOR EACH PERMANENT SERVICE PROVIDER WHO IS **NOT** ON DUTY TODAY, WRITE "999" (NOT ELIGIBLE) IN **Q10d**. FOR ALL PERMANENT SERVICE PROVIDERS WHO **ARE** ON DUTY TODAY, ASSIGN A NUMBER TO EACH OF THEM (SERIALIZE) IN Q10d STARTING WITH "01" TO THE LAST NUMBER. DO NOT CONSIDER THE "999" AS PART OF THE NUMBERING.

FOR FACILITIES WITH FOUR OR FEWER PROVIDERS ON DUTY TODAY, INTERVIEW ALL OF THEM. FOR FACILITIES WITH FIVE OR MORE PROVIDERS ON DUTY TODAY, WRITE ALL NUMBERS FROM Q10d (EXCEPT FOR "999") ON SMALL PIECES OF PAPER AND RANDOMLY SELECT FOUR PROVIDERS. ONCE YOU HAVE BALLOTED/SELECTED FOUR PROVIDERS FROM Q10d, CAREFULLY AND NEATLY CIRCLE THE NUMBERS IN Q10d FOR THOSE SELECTED.

Q10a. No. of staff	Q10b. NAME	Q10c. Is NAME scheduled to be on duty any time today?	Q10d. Serial number of sampled on-duty staff	Q10e. POSITION CODE	Q10f. Does NAME work full-time?	Q10g. SEX	Does NAME provide service(s)?			
							Q10h. FAMILY PLANNING	Q10i. MATERNAL HEALTH	Q10j. CHILD HEALTH	Q10k. VCT/STI/HIV SERVICES
(01)		YES . . . . .1<br>NO 2	☐☐☐	☐☐	YES. . .1<br>NO. . . .2	MALE . . . 1<br>FEMALE...2	YES . . .. .1<br>NO 2	YES . . . . . .1<br>NO 2	YES . . . . . .1<br>NO 2	YES . . . . . .1<br>NO 2
(02)		YES . . . . .1<br>NO 2	☐☐☐	☐☐	YES. . .1<br>NO. . . .2	MALE . . . 1<br>FEMALE...2	YES . . .. .1<br>NO 2	YES . . . . . .1<br>NO 2	YES . . . . . .1<br>NO 2	YES . . . . . .1<br>NO 2
(03)		YES . . . . .1<br>NO 2	☐☐☐	☐☐	YES. . .1<br>NO. . . .2	MALE . . . 1<br>FEMALE...2	YES . . .. .1<br>NO 2	YES . . . . . .1<br>NO 2	YES . . . . . .1<br>NO 2	YES . . . . . .1<br>NO 2
(04)		YES . . . . .1<br>NO 2	☐☐☐	☐☐	YES. . .1<br>NO. . . .2	MALE . . . 1<br>FEMALE...2	YES . . .. .1<br>NO 2	YES . . . . . .1<br>NO 2	YES . . . . . .1<br>NO 2	YES . . . . . .1<br>NO 2
(05)		YES . . . . .1<br>NO 2	☐☐☐	☐☐	YES. . .1<br>NO. . . .2	MALE . . . 1<br>FEMALE...2	YES . . .. .1<br>NO 2	YES . . . . . .1<br>NO 2	YES . . . . . .1<br>NO 2	YES . . . . . .1<br>NO 2
(06)		YES . . . . .1<br>NO 2	☐☐☐	☐☐	YES. . .1<br>NO. . . .2	MALE . . . 1<br>FEMALE...2	YES . . .. .1<br>NO 2	YES . . . . . .1<br>NO 2	YES . . . . . .1<br>NO 2	YES . . . . . .1<br>NO 2
(07)		YES . . . . .1<br>NO 2	☐☐☐	☐☐	YES. . .1<br>NO. . . .2	MALE . . . 1<br>FEMALE...2	YES . . .. .1<br>NO 2	YES . . . . . .1<br>NO 2	YES . . . . . .1<br>NO 2	YES . . . . . .1<br>NO 2
(08)		YES . . . . .1<br>NO 2	☐☐☐	☐☐	YES. . .1<br>NO. . . .2	MALE . . . 1<br>FEMALE...2	YES . . .. .1<br>NO 2	YES . . . . . .1<br>NO 2	YES . . . . . .1<br>NO 2	YES . . . . . .1<br>NO 2
(09)		YES . . . . .1<br>NO 2	☐☐☐	☐☐	YES. . .1<br>NO. . . .2	MALE . . . 1<br>FEMALE...2	YES . . .. .1<br>NO 2	YES . . . . . .1<br>NO 2	YES . . . . . .1<br>NO 2	YES . . . . . .1<br>NO 2
(10)		YES . . . . .1<br>NO 2	☐☐☐	☐☐	YES. . .1<br>NO. . . .2	MALE . . . 1<br>FEMALE...2	YES . . .. .1<br>NO 2	YES . . . . . .1<br>NO 2	YES . . . . . .1<br>NO 2	YES . . . . . .1<br>NO 2
(11)		YES . . . . .1<br>NO 2	☐☐☐	☐☐	YES. . .1<br>NO. . . .2	MALE . . . 1<br>FEMALE...2	YES . . .. .1<br>NO 2	YES . . . . . .1<br>NO 2	YES . . . . . .1<br>NO 2	YES . . . . . .1<br>NO 2
(12)		YES . . . . .1<br>NO 2	☐☐☐	☐☐	YES. . .1<br>NO. . . .2	MALE . . . 1<br>FEMALE...2	YES . . .. .1<br>NO 2	YES . . . . . .1<br>NO 2	YES . . . . . .1<br>NO 2	YES . . . . . .1<br>NO 2
(13)		YES . . . . .1<br>NO 2	☐☐☐	☐☐	YES. . .1<br>NO. . . .2	MALE . . . 1<br>FEMALE...2	YES . . .. .1<br>NO 2	YES . . . . . .1<br>NO 2	YES . . . . . .1<br>NO 2	YES . . . . . .1<br>NO 2
(14)		YES . . . . .1<br>NO 2	☐☐☐	☐☐	YES. . .1<br>NO. . . .2	MALE . . . 1<br>FEMALE...2	YES . . .. .1<br>NO 2	YES . . . . . .1<br>NO 2	YES . . . . . .1<br>NO 2	YES . . . . . .1<br>NO 2

CODE: Obstetrician/Gynecologists=01 General surgeons=02 Pediatricians=03 General physicians=04 Theatre nurse=05 Nurse/Midwives=06 Nurses=07 Midwives=08 CHEWs=09 Community health officers (CHO)=10 VCT Counselors=11 Other=96

STAFF

Q10a. No. of staff	Q10b. NAME	Q10c. Is NAME scheduled to be on duty any time today?	Q10d. Serial number of sampled on-duty staff	Q10e. POSITION CODE	Q10f. Does NAME work full-time?	Q10g. SEX	Does NAME provide service(s)? Q10h. FAMILY PLANNING	Q10i. MATERNAL HEALTH	Q10j. CHILD HEALTH	Q10k. VCT/STI/HIV SERVICES
(15)		YES . . . . .1<br>NO 2			YES. . .1<br>NO. . . .2	MALE . . . 1<br>FEMALE...2	YES . . .. .1<br>NO 2	YES . . . . . .1<br>NO 2	YES . . . . . .1<br>NO 2	YES . . . . . .1<br>NO 2
(16)		YES . . . . .1<br>NO 2			YES. . .1<br>NO. . . .2	MALE . . . 1<br>FEMALE...2	YES . . .. .1<br>NO 2	YES . . . . . .1<br>NO 2	YES . . . . . .1<br>NO 2	YES . . . . . .1<br>NO 2
(17)		YES . . . . .1<br>NO 2			YES. . .1<br>NO. . . .2	MALE . . . 1<br>FEMALE...2	YES . . .. .1<br>NO 2	YES . . . . . .1<br>NO 2	YES . . . . . .1<br>NO 2	YES . . . . . .1<br>NO 2
(18)		YES . . . . .1<br>NO 2			YES. . .1<br>NO. . . .2	MALE . . . 1<br>FEMALE...2	YES . . .. .1<br>NO 2	YES . . . . . .1<br>NO 2	YES . . . . . .1<br>NO 2	YES . . . . . .1<br>NO 2
(19)		YES . . . . .1<br>NO 2			YES. . .1<br>NO. . . .2	MALE . . . 1<br>FEMALE...2	YES . . .. .1<br>NO 2	YES . . . . . .1<br>NO 2	YES . . . . . .1<br>NO 2	YES . . . . . .1<br>NO 2
(20)		YES . . . . .1<br>NO 2			YES. . .1<br>NO. . . .2	MALE . . . 1<br>FEMALE...2	YES . . .. .1<br>NO 2	YES . . . . . .1<br>NO 2	YES . . . . . .1<br>NO 2	YES . . . . . .1<br>NO 2
(21)		YES . . . . .1<br>NO 2			YES. . .1<br>NO. . . .2	MALE . . . 1<br>FEMALE...2	YES . . .. .1<br>NO 2	YES . . . . . .1<br>NO 2	YES . . . . . .1<br>NO 2	YES . . . . . .1<br>NO 2
(22)		YES . . . . .1<br>NO 2			YES. . .1<br>NO. . . .2	MALE . . . 1<br>FEMALE...2	YES . . .. .1<br>NO 2	YES . . . . . .1<br>NO 2	YES . . . . . .1<br>NO 2	YES . . . . . .1<br>NO 2
(23)		YES . . . . .1<br>NO 2			YES. . .1<br>NO. . . .2	MALE . . . 1<br>FEMALE...2	YES . . .. .1<br>NO 2	YES . . . . . .1<br>NO 2	YES . . . . . .1<br>NO 2	YES . . . . . .1<br>NO 2
(24)		YES . . . . .1<br>NO 2			YES. . .1<br>NO. . . .2	MALE . . . 1<br>FEMALE...2	YES . . .. .1<br>NO 2	YES . . . . . .1<br>NO 2	YES . . . . . .1<br>NO 2	YES . . . . . .1<br>NO 2
(25)		YES . . . . .1<br>NO 2			YES. . .1<br>NO. . . .2	MALE . . . 1<br>FEMALE...2	YES . . .. .1<br>NO 2	YES . . . . . .1<br>NO 2	YES . . . . . .1<br>NO 2	YES . . . . . .1<br>NO 2
(26)		YES . . . . .1<br>NO 2			YES. . .1<br>NO. . . .2	MALE . . . 1<br>FEMALE...2	YES . . .. .1<br>NO 2	YES . . . . . .1<br>NO 2	YES . . . . . .1<br>NO 2	YES . . . . . .1<br>NO 2
(27)		YES . . . . .1<br>NO 2			YES. . .1<br>NO. . . .2	MALE . . . 1<br>FEMALE...2	YES . . .. .1<br>NO 2	YES . . . . . .1<br>NO 2	YES . . . . . .1<br>NO 2	YES . . . . . .1<br>NO 2
(28)		YES . . . . .1<br>NO 2			YES. . .1<br>NO. . . .2	MALE . . . 1<br>FEMALE...2	YES . . .. .1<br>NO 2	YES . . . . . .1<br>NO 2	YES . . . . . .1<br>NO 2	YES . . . . . .1<br>NO 2
(29)		YES . . . . .1<br>NO 2			YES. . .1<br>NO. . . .2	MALE . . . 1<br>FEMALE...2	YES . . .. .1<br>NO 2	YES . . . . . .1<br>NO 2	YES . . . . . .1<br>NO 2	YES . . . . . .1<br>NO 2
(30)		YES . . . . .1<br>NO 2			YES. . .1<br>NO. . . .2	MALE . . . 1<br>FEMALE...2	YES . . .. .1<br>NO 2	YES . . . . . .1<br>NO 2	YES . . . . . .1<br>NO 2	YES . . . . . .1<br>NO 2
(31)		YES . . . . .1<br>NO 2			YES. . .1<br>NO. . . .2	MALE . . . 1<br>FEMALE...2	YES . . .. .1<br>NO 2	YES . . . . . .1<br>NO 2	YES . . . . . .1<br>NO 2	YES . . . . . .1<br>NO 2
(32)		YES . . . . .1<br>NO 2			YES. . .1<br>NO. . . .2	MALE . . . 1<br>FEMALE...2	YES . . .. .1<br>NO 2	YES . . . . . .1<br>NO 2	YES . . . . . .1<br>NO 2	YES . . . . . .1<br>NO 2
(33)		YES . . . . .1<br>NO 2			YES. . .1<br>NO. . . .2	MALE . . . 1<br>FEMALE...2	YES . . .. .1<br>NO 2	YES . . . . . .1<br>NO 2	YES . . . . . .1<br>NO 2	YES . . . . . .1<br>NO 2

CODE: Obstetrician/Gynecologists=01, General surgeons=02, Pediatricians=03, General physicians=04, Theatre nurse=05, Nurse/Midwives=06, Nurses=07, Midwives=08, CHEWs=09, Community health officers (CHO)=10, VCT Counselors=11, Other=96

STAFF										
Q10a. No. of staff	Q10b. NAME	Q10c. Is NAME scheduled to be on duty any time today?	Q10d. Serial number of sampled on-duty staff	Q10e. POSITION CODE	Q10f. Does NAME work full-time?	Q10g. SEX	Does NAME provide service(s)?			
							Q10h. FAMILY PLANNING	Q10i. MATERNAL HEALTH	Q10j. CHILD HEALTH	Q10k. VCT/STI/HIV SERVICES
(34)		YES . . . . .1<br>NO 2			YES. . .1<br>NO. . . .2	MALE . . . 1<br>FEMALE...2	YES . . . . .1<br>NO 2	YES . . . . . .1<br>NO 2	YES . . . . . .1<br>NO 2	YES . . . . . .1<br>NO 2
(35)		YES . . . . .1<br>NO 2			YES. . .1<br>NO. . . .2	MALE . . . 1<br>FEMALE...2	YES . . . . .1<br>NO 2	YES . . . . . .1<br>NO 2	YES . . . . . .1<br>NO 2	YES . . . . . .1<br>NO 2
(36)		YES . . . . .1<br>NO 2			YES. . .1<br>NO. . . .2	MALE . . . 1<br>FEMALE...2	YES . . . . .1<br>NO 2	YES . . . . . .1<br>NO 2	YES . . . . . .1<br>NO 2	YES . . . . . .1<br>NO 2
(37)		YES . . . . .1<br>NO 2			YES. . .1<br>NO. . . .2	MALE . . . 1<br>FEMALE...2	YES . . . . .1<br>NO 2	YES . . . . . .1<br>NO 2	YES . . . . . .1<br>NO 2	YES . . . . . .1<br>NO 2
(38)		YES . . . . .1<br>NO 2			YES. . .1<br>NO. . . .2	MALE . . . 1<br>FEMALE...2	YES . . . . .1<br>NO 2	YES . . . . . .1<br>NO 2	YES . . . . . .1<br>NO 2	YES . . . . . .1<br>NO 2
(39)		YES . . . . .1<br>NO 2			YES. . .1<br>NO. . . .2	MALE . . . 1<br>FEMALE...2	YES . . . . .1<br>NO 2	YES . . . . . .1<br>NO 2	YES . . . . . .1<br>NO 2	YES . . . . . .1<br>NO 2
(40)		YES . . . . .1<br>NO 2			YES. . .1<br>NO. . . .2	MALE . . . 1<br>FEMALE...2	YES . . . . .1<br>NO 2	YES . . . . . .1<br>NO 2	YES . . . . . .1<br>NO 2	YES . . . . . .1<br>NO 2
(41)		YES . . . . .1<br>NO 2			YES. . .1<br>NO. . . .2	MALE . . . 1<br>FEMALE...2	YES . . . . .1<br>NO 2	YES . . . . . .1<br>NO 2	YES . . . . . .1<br>NO 2	YES . . . . . .1<br>NO 2
(42)		YES . . . . .1<br>NO 2			YES. . .1<br>NO. . . .2	MALE . . . 1<br>FEMALE...2	YES . . . . .1<br>NO 2	YES . . . . . .1<br>NO 2	YES . . . . . .1<br>NO 2	YES . . . . . .1<br>NO 2
(43)		YES . . . . .1<br>NO 2			YES. . .1<br>NO. . . .2	MALE . . . 1<br>FEMALE...2	YES . . . . .1<br>NO 2	YES . . . . . .1<br>NO 2	YES . . . . . .1<br>NO 2	YES . . . . . .1<br>NO 2
(44)		YES . . . . .1<br>NO 2			YES. . .1<br>NO. . . .2	MALE . . . 1<br>FEMALE...2	YES . . . . .1<br>NO 2	YES . . . . . .1<br>NO 2	YES . . . . . .1<br>NO 2	YES . . . . . .1<br>NO 2
(45)		YES . . . . .1<br>NO 2			YES. . .1<br>NO. . . .2	MALE . . . 1<br>FEMALE...2	YES . . . . .1<br>NO 2	YES . . . . . .1<br>NO 2	YES . . . . . .1<br>NO 2	YES . . . . . .1<br>NO 2
(46)		YES . . . . .1<br>NO 2			YES. . .1<br>NO. . . .2	MALE . . . 1<br>FEMALE...2	YES . . . . .1<br>NO 2	YES . . . . . .1<br>NO 2	YES . . . . . .1<br>NO 2	YES . . . . . .1<br>NO 2
(47)		YES . . . . .1<br>NO 2			YES. . .1<br>NO. . . .2	MALE . . . 1<br>FEMALE...2	YES . . . . .1<br>NO 2	YES . . . . . .1<br>NO 2	YES . . . . . .1<br>NO 2	YES . . . . . .1<br>NO 2
(48)		YES . . . . .1<br>NO 2			YES. . .1<br>NO. . . .2	MALE . . . 1<br>FEMALE...2	YES . . . . .1<br>NO 2	YES . . . . . .1<br>NO 2	YES . . . . . .1<br>NO 2	YES . . . . . .1<br>NO 2
(49)		YES . . . . .1<br>NO 2			YES. . .1<br>NO. . . .2	MALE . . . 1<br>FEMALE...2	YES . . . . .1<br>NO 2	YES . . . . . .1<br>NO 2	YES . . . . . .1<br>NO 2	YES . . . . . .1<br>NO 2
(50)		YES . . . . .1<br>NO 2			YES. . .1<br>NO. . . .2	MALE . . . 1<br>FEMALE...2	YES . . . . .1<br>NO 2	YES . . . . . .1<br>NO 2	YES . . . . . .1<br>NO 2	YES . . . . . .1<br>NO 2
(51)		YES . . . . .1<br>NO 2			YES. . .1<br>NO. . . .2	MALE . . . 1<br>FEMALE...2	YES . . . . .1<br>NO 2	YES . . . . . .1<br>NO 2	YES . . . . . .1<br>NO 2	YES . . . . . .1<br>NO 2
(52)		YES . . . . .1<br>NO 2			YES. . .1<br>NO. . . .2	MALE . . . 1<br>FEMALE...2	YES . . . . .1<br>NO 2	YES . . . . . .1<br>NO 2	YES . . . . . .1<br>NO 2	YES . . . . . .1<br>NO 2

CODE: Obstetrician/Gynecologists=01 General surgeons=02 Pediatricians=03 General physicians=04 Theatre nurse=05 Nurse/Midwives=06 Nurses=07 Midwives=08 CHEWs=09 Community health officers (CHO)=10 VCT Counselors=11 Other=96

STAFF										
Q10a. No. of staff	Q10b. NAME	Q10c. Is NAME scheduled to be on duty any time today?	Q10d. Serial number of sampled on-duty staff	Q10e. POSITION CODE	Q10f. Does NAME work full-time?	Q10g. SEX	Does NAME provide service(s)?			
							Q10h. FAMILY PLANNING	Q10i. MATERNAL HEALTH	Q10j. CHILD HEALTH	Q10k. VCT/STI/HIV SERVICES
(53)		YES . . . . .1<br>NO 2			YES. . .1<br>NO. . . .2	MALE . . . 1<br>FEMALE...2	YES . . . . .1<br>NO 2	YES . . . . . .1<br>NO 2	YES . . . . . .1<br>NO 2	YES . . . . . .1<br>NO 2
(54)		YES . . . . .1<br>NO 2			YES. . .1<br>NO. . . .2	MALE . . . 1<br>FEMALE...2	YES . . . . .1<br>NO 2	YES . . . . . .1<br>NO 2	YES . . . . . .1<br>NO 2	YES . . . . . .1<br>NO 2
(55)		YES . . . . .1<br>NO 2			YES. . .1<br>NO. . . .2	MALE . . . 1<br>FEMALE...2	YES . . . . .1<br>NO 2	YES . . . . . .1<br>NO 2	YES . . . . . .1<br>NO 2	YES . . . . . .1<br>NO 2
(56)		YES . . . . .1<br>NO 2			YES. . .1<br>NO. . . .2	MALE . . . 1<br>FEMALE...2	YES . . . . .1<br>NO 2	YES . . . . . .1<br>NO 2	YES . . . . . .1<br>NO 2	YES . . . . . .1<br>NO 2
(57)		YES . . . . .1<br>NO 2			YES. . .1<br>NO. . . .2	MALE . . . 1<br>FEMALE...2	YES . . . . .1<br>NO 2	YES . . . . . .1<br>NO 2	YES . . . . . .1<br>NO 2	YES . . . . . .1<br>NO 2
(58)		YES . . . . .1<br>NO 2			YES. . .1<br>NO. . . .2	MALE . . . 1<br>FEMALE...2	YES . . . . .1<br>NO 2	YES . . . . . .1<br>NO 2	YES . . . . . .1<br>NO 2	YES . . . . . .1<br>NO 2
(59)		YES . . . . .1<br>NO 2			YES. . .1<br>NO. . . .2	MALE . . . 1<br>FEMALE...2	YES . . . . .1<br>NO 2	YES . . . . . .1<br>NO 2	YES . . . . . .1<br>NO 2	YES . . . . . .1<br>NO 2
(60)		YES . . . . .1<br>NO 2			YES. . .1<br>NO. . . .2	MALE . . . 1<br>FEMALE...2	YES . . . . .1<br>NO 2	YES . . . . . .1<br>NO 2	YES . . . . . .1<br>NO 2	YES . . . . . .1<br>NO 2
(61)		YES . . . . .1<br>NO 2			YES. . .1<br>NO. . . .2	MALE . . . 1<br>FEMALE...2	YES . . . . .1<br>NO 2	YES . . . . . .1<br>NO 2	YES . . . . . .1<br>NO 2	YES . . . . . .1<br>NO 2
(62)		YES . . . . .1<br>NO 2			YES. . .1<br>NO. . . .2	MALE . . . 1<br>FEMALE...2	YES . . . . .1<br>NO 2	YES . . . . . .1<br>NO 2	YES . . . . . .1<br>NO 2	YES . . . . . .1<br>NO 2
(63)		YES . . . . .1<br>NO 2			YES. . .1<br>NO. . . .2	MALE . . . 1<br>FEMALE...2	YES . . . . .1<br>NO 2	YES . . . . . .1<br>NO 2	YES . . . . . .1<br>NO 2	YES . . . . . .1<br>NO 2
(64)		YES . . . . .1<br>NO 2			YES. . .1<br>NO. . . .2	MALE . . . 1<br>FEMALE...2	YES . . . . .1<br>NO 2	YES . . . . . .1<br>NO 2	YES . . . . . .1<br>NO 2	YES . . . . . .1<br>NO 2
(65)		YES . . . . .1<br>NO 2			YES. . .1<br>NO. . . .2	MALE . . . 1<br>FEMALE...2	YES . . . . .1<br>NO 2	YES . . . . . .1<br>NO 2	YES . . . . . .1<br>NO 2	YES . . . . . .1<br>NO 2
(66)		YES . . . . .1<br>NO 2			YES. . .1<br>NO. . . .2	MALE . . . 1<br>FEMALE...2	YES . . . . .1<br>NO 2	YES . . . . . .1<br>NO 2	YES . . . . . .1<br>NO 2	YES . . . . . .1<br>NO 2
(67)		YES . . . . .1<br>NO 2			YES. . .1<br>NO. . . .2	MALE . . . 1<br>FEMALE...2	YES . . . . .1<br>NO 2	YES . . . . . .1<br>NO 2	YES . . . . . .1<br>NO 2	YES . . . . . .1<br>NO 2
(68)		YES . . . . .1<br>NO 2			YES. . .1<br>NO. . . .2	MALE . . . 1<br>FEMALE...2	YES . . . . .1<br>NO 2	YES . . . . . .1<br>NO 2	YES . . . . . .1<br>NO 2	YES . . . . . .1<br>NO 2
(69)		YES . . . . .1<br>NO 2			YES. . .1<br>NO. . . .2	MALE . . . 1<br>FEMALE...2	YES . . . . .1<br>NO 2	YES . . . . . .1<br>NO 2	YES . . . . . .1<br>NO 2	YES . . . . . .1<br>NO 2
(70)		YES . . . . .1<br>NO 2			YES. . .1<br>NO. . . .2	MALE . . . 1<br>FEMALE...2	YES . . . . .1<br>NO 2	YES . . . . . .1<br>NO 2	YES . . . . . .1<br>NO 2	YES . . . . . .1<br>NO 2
(71)		YES . . . . .1<br>NO 2			YES. . .1<br>NO. . . .2	MALE . . . 1<br>FEMALE...2	YES . . . . .1<br>NO 2	YES . . . . . .1<br>NO 2	YES . . . . . .1<br>NO 2	YES . . . . . .1<br>NO 2

CODE: Obstetrician/Gynecologists=01, General surgeons=02, Pediatricians=03, General physicians=04, Theatre nurse=05, Nurse/Midwives=06, Nurses=07, Midwives=08, CHEWs=09, Community health officers (CHO)=10, VCT Counselors=11, Other=96

STAFF

Q10a. No. of staff	Q10b. NAME	Q10c. Is NAME scheduled to be on duty any time today?	Q10d. Serial number of sampled on-duty staff	Q10e. POSITION CODE	Q10f. Does NAME work full-time?	Q10g. SEX	Does NAME provide service(s)? Q10h. FAMILY PLANNING	Q10i. MATERNAL HEALTH	Q10j. CHILD HEALTH	Q10k. VCT/STI/HIV SERVICES
(72)		YES . . . . .1<br>NO 2			YES. . .1<br>NO. . . .2	MALE . . . 1<br>FEMALE...2	YES . . . . .1<br>NO 2	YES . . . . . .1<br>NO 2	YES . . . . . .1<br>NO 2	YES . . . . . .1<br>NO 2
(73)		YES . . . . .1<br>NO 2			YES. . .1<br>NO. . . .2	MALE . . . 1<br>FEMALE...2	YES . . . . .1<br>NO 2	YES . . . . . .1<br>NO 2	YES . . . . . .1<br>NO 2	YES . . . . . .1<br>NO 2
(74)		YES . . . . .1<br>NO 2			YES. . .1<br>NO. . . .2	MALE . . . 1<br>FEMALE...2	YES . . . . .1<br>NO 2	YES . . . . . .1<br>NO 2	YES . . . . . .1<br>NO 2	YES . . . . . .1<br>NO 2
(75)		YES . . . . .1<br>NO 2			YES. . .1<br>NO. . . .2	MALE . . . 1<br>FEMALE...2	YES . . . . .1<br>NO 2	YES . . . . . .1<br>NO 2	YES . . . . . .1<br>NO 2	YES . . . . . .1<br>NO 2
(76)		YES . . . . .1<br>NO 2			YES. . .1<br>NO. . . .2	MALE . . . 1<br>FEMALE...2	YES . . . . .1<br>NO 2	YES . . . . . .1<br>NO 2	YES . . . . . .1<br>NO 2	YES . . . . . .1<br>NO 2
(77)		YES . . . . .1<br>NO 2			YES. . .1<br>NO. . . .2	MALE . . . 1<br>FEMALE...2	YES . . . . .1<br>NO 2	YES . . . . . .1<br>NO 2	YES . . . . . .1<br>NO 2	YES . . . . . .1<br>NO 2
(78)		YES . . . . .1<br>NO 2			YES. . .1<br>NO. . . .2	MALE . . . 1<br>FEMALE...2	YES . . . . .1<br>NO 2	YES . . . . . .1<br>NO 2	YES . . . . . .1<br>NO 2	YES . . . . . .1<br>NO 2
(79)		YES . . . . .1<br>NO 2			YES. . .1<br>NO. . . .2	MALE . . . 1<br>FEMALE...2	YES . . . . .1<br>NO 2	YES . . . . . .1<br>NO 2	YES . . . . . .1<br>NO 2	YES . . . . . .1<br>NO 2
(80)		YES . . . . .1<br>NO 2			YES. . .1<br>NO. . . .2	MALE . . . 1<br>FEMALE...2	YES . . . . .1<br>NO 2	YES . . . . . .1<br>NO 2	YES . . . . . .1<br>NO 2	YES . . . . . .1<br>NO 2
(81)		YES . . . . .1<br>NO 2			YES. . .1<br>NO. . . .2	MALE . . . 1<br>FEMALE...2	YES . . . . .1<br>NO 2	YES . . . . . .1<br>NO 2	YES . . . . . .1<br>NO 2	YES . . . . . .1<br>NO 2
(82)		YES . . . . .1<br>NO 2			YES. . .1<br>NO. . . .2	MALE . . . 1<br>FEMALE...2	YES . . . . .1<br>NO 2	YES . . . . . .1<br>NO 2	YES . . . . . .1<br>NO 2	YES . . . . . .1<br>NO 2
(83)		YES . . . . .1<br>NO 2			YES. . .1<br>NO. . . .2	MALE . . . 1<br>FEMALE...2	YES . . . . .1<br>NO 2	YES . . . . . .1<br>NO 2	YES . . . . . .1<br>NO 2	YES . . . . . .1<br>NO 2
(84)		YES . . . . .1<br>NO 2			YES. . .1<br>NO. . . .2	MALE . . . 1<br>FEMALE...2	YES . . . . .1<br>NO 2	YES . . . . . .1<br>NO 2	YES . . . . . .1<br>NO 2	YES . . . . . .1<br>NO 2
(85)		YES . . . . .1<br>NO 2			YES. . .1<br>NO. . . .2	MALE . . . 1<br>FEMALE...2	YES . . . . .1<br>NO 2	YES . . . . . .1<br>NO 2	YES . . . . . .1<br>NO 2	YES . . . . . .1<br>NO 2
(86)		YES . . . . .1<br>NO 2			YES. . .1<br>NO. . . .2	MALE . . . 1<br>FEMALE...2	YES . . . . .1<br>NO 2	YES . . . . . .1<br>NO 2	YES . . . . . .1<br>NO 2	YES . . . . . .1<br>NO 2
(87)		YES . . . . .1<br>NO 2			YES. . .1<br>NO. . . .2	MALE . . . 1<br>FEMALE...2	YES . . . . .1<br>NO 2	YES . . . . . .1<br>NO 2	YES . . . . . .1<br>NO 2	YES . . . . . .1<br>NO 2
(88)		YES . . . . .1<br>NO 2			YES. . .1<br>NO. . . .2	MALE . . . 1<br>FEMALE...2	YES . . . . .1<br>NO 2	YES . . . . . .1<br>NO 2	YES . . . . . .1<br>NO 2	YES . . . . . .1<br>NO 2

CODE: Obstetrician/Gynecologists=01 General surgeons=02 Pediatricians=03 General physicians=04 Theatre nurse=05 Nurse/Midwives=06 Nurses=07 Midwives=08 CHEWs=09 Community health officers (CHO)=10 VCT Counselors=11 Other=96

CHECK THE BOX IF ANOTHER FORM IS USED: ☐ TOTAL NUMBER OF FORMS: ☐ FORM NUMBER: ☐

GENERAL MCH AND FP				
SERVICE	Q11a. Does this facility provide the following Maternal and Child Health SERVICES?	Q11b. How many days per week is SERVICE available?	Q11c. How many clients received this service here in the past 3 months? ASK TO SEE MEDICAL RECORD SYSTEM, IF POSSIBLE. OTHERWISE, ASK RESPONDENT TO RECALL.	Q11d.WHAT WAS THE SOURCE OF THIS INFORMATION?
(1) Maternity care/delivery services	Yes . . . . 1<br>No . . . . . 2→**(2)**	Days . . . ☐	☐☐☐☐☐<br>**NOT AVAILABLE.....99993**	OBSERVED RECORD…………….1<br>PROVIDER ESTIMATE…..………2
(2) Counseling on initiating breast-feeding (after delivery)	Yes . . . . 1<br>No 2			
(3) Emergency care for prolonged or obstructed labor (cesarean section, blood transfusion)	Yes . . . . 1<br>No . . . . . 2→**(4)**	Days . . . ☐	☐☐☐☐☐<br>**NOT AVAILABLE.....99993**	OBSERVED RECORD…………….1<br>PROVIDER ESTIMATE…..………2
(4) Consultation for infertility	Yes . . . . 1<br>No . . . . . 2→**(5)**	Days . . . ☐	☐☐☐☐☐<br>**NOT AVAILABLE.....99993**	OBSERVED RECORD…………….1<br>PROVIDER ESTIMATE…..………2
(5) Post-abortion care	Yes . . . . 1<br>No . . . . . 2→**(6)**	Days . . . ☐	☐☐☐☐☐<br>**NOT AVAILABLE.....99993**	OBSERVED RECORD…………….1<br>PROVIDER ESTIMATE…..………2
(6) Ante-natal care	Yes . . . . 1<br>No . . . . 2→**(12)**	Days . . . ☐	☐☐☐☐☐<br>**NOT AVAILABLE.....99993**	OBSERVED RECORD…………….1<br>PROVIDER ESTIMATE…..………2
(7) Complete Regimen of Tetanus Toxoid immunization during pregnancy (5 doses)	Yes . . . . 1<br>No 2			
(8) Syphilis screening during pregnancy	Yes . . . . 1<br>No 2			
(9) Iron supplementation during pregnancy	Yes . . . . 1<br>No 2			
(10) Intermittent preventive treatment for malaria (IPT)	Yes . . . . 1<br>No 2			
(11) Nutrition counseling during pregnancy	Yes . . . . 1<br>No 2			

SERVICE	Q11a. Does this facility provide the following Maternal and Child Health SERVICE?	Q11b. How many days per week is SERVICE available?	Q11c. How many clients received this service here in the past 3 months? ASK TO SEE MEDICAL RECORD SYSTEM, IF POSSIBLE. OTHERWISE, ASK RESPONDENT TO RECALL.	Q11d.WHAT WAS THE SOURCE OF THIS INFORMATION?
(12) Post natal care	Yes . . . . 1<br>No . . . . 2→**(14)**	Days . . . ☐	☐☐☐☐☐<br>**NOT AVAILABLE.....99993**	OBSERVED RECORD................1<br>PROVIDER ESTIMATE..............2
(13) Vitamin A supplementation after pregnancy	Yes . . . . 1<br>No 2			
(14) Child immunization	Yes . . . . 1<br>No . . . . 2→**(15)**	Days . . . ☐	☐☐☐☐☐<br>**NOT AVAILABLE.....99993**	OBSERVED RECORD................1<br>PROVIDER ESTIMATE..............2
(15) Child growth monitoring	Yes . . . . 1<br>No . . . . 2→**(16)**	Days . . . ☐	☐☐☐☐☐<br>**NOT AVAILABLE.....99993**	OBSERVED RECORD................1<br>PROVIDER ESTIMATE..............2
(16) Child respiratory disease	Yes . . . . 1<br>No . . . . 2→**(17)**	Days . . . ☐	☐☐☐☐☐<br>**NOT AVAILABLE.....99993**	OBSERVED RECORD................1<br>PROVIDER ESTIMATE..............2
(17) Oral rehydration therapy services	Yes . . . . 1<br>No . . . . 2→**(18)**	Days . . . ☐	☐☐☐☐☐<br>**NOT AVAILABLE.....99993**	OBSERVED RECORD................1<br>PROVIDER ESTIMATE..............2
(18) Detection and treatment of sexually transmitted infections (STIs)	Yes . . . . 1<br>No . . . . 2→**(19)**	Days . . . ☐	☐☐☐☐☐<br>**NOT AVAILABLE.....99993**	OBSERVED RECORD................1<br>PROVIDER ESTIMATE..............2
(19) Voluntary counseling and testing (VCT)	Yes . . . . 1<br>No . . . . 2→**(20)**	Days . . . ☐	☐☐☐☐☐<br>**NOT AVAILABLE.....99993**	OBSERVED RECORD................1<br>PROVIDER ESTIMATE..............2
(20) PMTCT	Yes . . . . 1<br>No . . . . 2→**(21)**	Days . . . ☐	☐☐☐☐☐<br>**NOT AVAILABLE.....99993**	OBSERVED RECORD................1<br>PROVIDER ESTIMATE..............2
(21) HIV/AIDS Management	Yes . . . . 1<br>No . . . . 2→**(22)**	Days . . . ☐	☐☐☐☐☐<br>**NOT AVAILABLE.....99993**	OBSERVED RECORD................1<br>PROVIDER ESTIMATE..............2
(22) Family planning counseling & services	Yes . . . . 1<br>No . . 2→**(Q12)**	Days . . . ☐	☐☐☐☐☐<br>**NOT AVAILABLE.....99993**	OBSERVED RECORD................1<br>PROVIDER ESTIMATE..............2

Q12.	Approximately, what percentage of the clients who received family planning counseling and services in the past 3 months were between the ages of 15 and 19 years old?	☐☐☐<br>NONE…………..000<br>DON'T KNOW….998	
Q13.	Does this facility ever refer clients to other health care facilities?	Yes . . . . 1<br>No . . . . 2 →	Q15
Q14.	For which services are these referrals?<br><br>[**MULTIPLE RESPONSE POSSIBLE**]	FAMILY PLANNING……………………………………A<br>IMMUNIZATION…………………………………….....B<br>ANTENATAL CARE……………………………………..C<br>DELIVERY CARE…………………………………….D<br>EMERGENCY DELIVERY CARE (C-SECTION)……….E<br>POSTNATAL CARE…………………………………F<br>DISEASE PREVENTION……………………………..G<br>TREATMENT OF ADULT…………………………....H<br>TREATMENT FOR CHILD…………………………I<br>GROWTH MONITORING OF CHILD…………………..J<br>HEALTH CHECK-UP . . . . ……………... …K<br>VCT……………………………………………………L<br>HIV/AIDS MANAGEMENT…………………………….M<br>PMTCT…………………………………………………N<br>OTHER__________________________X<br>(SPECIFY)	
Now I would like to ask you some questions about other health services.			
Q15.	CHECK Q11A. IF YES TO (14) CHILD IMMUNIZATION, (15) CHILD GROWTH MONITORING, OR (16) CHILD RESPIRATORY DISEASE ☐ ↓	IF NO TO ALL CHILD SERVICES (14-16) ☐ →	Q19
Q16.	What is the normal practice for this facility if a woman who has come for a ***child health visit*** is interested in ***receiving information*** on FP? Is she able to receive this information on the day of her visit, or is she asked to come back on a different day?<br><br>CIRCLE ONE.	Always receive on same day…………………………01<br>Sometimes receive on same day……………………02<br>Make appointment to come back a different day…..03<br>No appointment made, always told to come back different day……………………………………….04<br>Given referral to another facility………………………05<br>Given no information or referral………………………06<br>Do not offer family planning services………………..07 →<br>Other__________________________________96<br>(SPECIFY)	Q19
Q17.	If a woman who has come for a ***child health visit*** is interested in ***receiving a hormonal method of FP***, what is the normal practice for this facility?<br><br>CIRCLE ONE.	Always receive on same day…………………………01<br>Sometimes receive on same day……………………02<br>Make appointment to come back a different day…..03<br>No appointment made, always told to come back different day……………………………………….04<br>Given referral to another facility………………………05<br>Given no information or referral………………………06<br>Other__________________________________96<br>(SPECIFY)	
Q18.	If a woman who has come for a ***child health visit*** is interested in ***getting sterilized***, what is the normal practice for this facility?<br><br>CIRCLE ONE.	Procedure can happen on same day…………………01<br>Sometimes the procedure can happen on same day……………………………………………………02<br>Make appointment to come back a different day……03<br>No appointment made, always told to come back different day……………………………………….04<br>Given referral to another facility………………………05<br>Given no information or referral………………………06<br>Other__________________________________96<br>(SPECIFY)	
Q19.	CHECK Q11A: IF YES TO (1) MATERNITY CARE/ DELIVERY SERVICES ☐ ↓	IF NO TO (1) MATERNITY CARE/DELIVERY SERVICES ☐ →	Q23
Q20.	What is the normal practice for this facility if a woman who has come for ***maternity care/delivery service*** is interested in ***receiving information*** on FP? Is she able to receive this information on the day of her visit, or is she asked to come back on a different day?<br><br>CIRCLE ONE.	Always receive on same day…………………………01<br>Sometimes receive on same day……………………02<br>Make appointment to come back a different day…..03<br>No appointment made, always told to come back different day……………………………………….04<br>Given referral to another facility………………………05<br>Given no information or referral………………………06<br>Do not offer family planning services………………07 →<br>Other__________________________________96<br>(SPECIFY)	Q23

Q21.	If a woman who has come for ***maternity care/delivery service*** is interested in ***receiving a hormonal method of FP***, what is the normal practice for this facility?<br><br>CIRCLE ONE.	Always receive on same day……………………….01<br>Sometimes receive on same day…………………….02<br>Make appointment to come back a different day…..03<br>No appointment made, always told to come back different day……………………………………….04<br>Given referral to another facility……………………05<br>Given no information or referral……………………06<br>Other_________________________________96<br>(SPECIFY)	
Q22.	If a woman who has come for ***maternity care/delivery service*** is interested in ***getting sterilized***, what is the normal practice for this facility?<br><br>CIRCLE ONE.	Procedure can happen on same day…………………01<br>Sometimes the procedure can happen on same day……………………………………………….02<br>Make appointment to come back a different day……03<br>No appointment made, always told to come back different day……………………………………….04<br>Given referral to another facility……………………05<br>Given no information or referral……………………06<br>Other_________________________________96<br>(SPECIFY)	
Q23.	CHECK Q11A: IF YES TO (12) POST NATAL CARE ☐ ↓	IF NO TO (12) POST NATAL CARE ☐ →	**Q27**
Q24.	What is the normal practice for this facility if a woman who has come for a ***postnatal care visit*** is interested in ***receiving information*** on FP? Is she able to receive this information on the day of her visit, or is she asked to come back on a different day?<br><br>CIRCLE ONE.	Always receive on same day……………………….01<br>Sometimes receive on same day…………………….02<br>Make appointment to come back a different day…..03<br>No appointment made, always told to come back different day……………………………………….04<br>Given referral to another facility……………………05<br>Given no information or referral……………………06<br>Do not offer family planning services………………07 →<br>Other_________________________________96<br>(SPECIFY)	**Q27**
Q25.	If a woman who has come for a ***postnatal care visit*** is interested in ***receiving a hormonal method of FP***, what is the normal practice for this facility?<br><br>CIRCLE ONE.	Always receive on same day……………………….01<br>Sometimes receive on same day…………………….02<br>Make appointment to come back a different day…..03<br>No appointment made, always told to come back different day……………………………………….04<br>Given referral to another facility……………………05<br>Given no information or referral……………………06<br>Other_________________________________96<br>(SPECIFY)	
Q26.	If a woman who has come for a ***postnatal care visit*** is interested in ***getting sterilized***, what is the normal practice for this facility?<br><br>CIRCLE ONE.	Procedure can happen on same day…………………01<br>Sometimes the procedure can happen on same day……………………………………………….02<br>Make appointment to come back a different day…...03<br>No appointment made, always told to come back different day……………………………………….04<br>Given referral to another facility……………………05<br>Given no information or referral……………………06<br>Other_________________________________96<br>(SPECIFY)	
Q27.	CHECK Q11A. IF YES TO (5) POST-ABORTION CARE ☐ ↓	IF NO TO (5) POST-ABORTION CARE ☐ →	**Q31**
Q28.	What is the normal practice for this facility if a woman who has come for ***post-abortion care*** is interested in ***receiving information*** on FP? Is she able to receive this information on the day of her visit, or is she asked to come back on a different day?<br><br>CIRCLE ONE.	Always receive on same day……………………….01<br>Sometimes receive on same day…………………….02<br>Make appointment to come back a different day…..03<br>No appointment made, always told to come back different day……………………………………….04<br>Given referral to another facility……………………05<br>Given no information or referral……………………06<br>Do not offer family planning services………………07 →<br><br>Other (specify)___________________________96	**Q31**

Q29.	If a woman who has come for ***post-abortion care*** is interested in ***receiving a hormonal method of FP***, what is the normal practice for this facility?<br><br>CIRCLE ONE.	Always receive on same day…………………………01<br>Sometimes receive on same day……………………02<br>Make appointment to come back a different day….03<br>No appointment made, always told to come back different day…………………………………………..04<br>Given referral to another facility………………………05<br>Given no information or referral………………………06<br>Other_________________________________96<br>(SPECIFY)	
Q30.	If a woman who has come for ***post-abortion care*** is interested in ***getting sterilized***, what is the normal practice for this facility?<br><br>CIRCLE ONE.	Procedure can happen on same day…………………01<br>Sometimes the procedure can happen on same day………………………………………………….02<br>Make appointment to come back a different day….03<br>No appointment made, always told to come back different day…………………………………………..04<br>Given referral to another facility………………………05<br>Given no information or referral………………………06<br>Other_________________________________96	
Q31.	CHECK Q11A: IF YES TO ANY (18) Detection and treatment of STIs, (19) VCT, (20) PMTCT, OR (21) HIV/AIDS management ☐ ↓	IF NO TO ALL (18-21) ☐ →	**Q35**
Q32.	What is the normal practice for this facility if a woman or man who has come for ***STI treatment, VCT, PMTCT, or HIV/AIDS care*** is interested in ***receiving information*** on FP, is she/he able to receive this information on the day of her/his visit, or is she/he asked to come back on a different day?<br><br>CIRCLE ONE.	Always receive on same day…………………………01<br>Sometimes receive on same day……………………02<br>Make appointment to come back a different day….03<br>No appointment made, always told to come back different day…………………………………………..04<br>Given referral to another facility………………………05<br>Given no information or referral………………………06<br>Do not offer family planning services………………07 →<br>Other_________________________________96<br>(SPECIFY)	**Q35**
Q33.	If a woman who has come for ***STI treatment, VCT, PMTCT, or HIV/AIDS care*** is interested in ***receiving a hormonal method***, what is the normal practice for this facility?<br><br>CIRCLE ONE.	Always receive on same day…………………………01<br>Sometimes receive on same day……………………02<br>Make appointment to come back a different day….03<br>No appointment made, always told to come back different day…………………………………………..04<br>Given referral to another facility………………………05<br>Given no information or referral………………………06<br>Other_________________________________96<br>(SPECIFY)	
Q34.	If a woman who has come for ***STI treatment, VCT, PMTCT, or HIV/AIDS care*** is interested in ***getting sterilized***, what is the normal practice for this facility?<br><br>CIRCLE ONE.	Procedure can happen on same day…………………01<br>Sometimes the procedure can happen on same day………………………………………………….02<br>Make appointment to come back a different day….03<br>No appointment made, always told to come back different day…………………………………………..04<br>Given referral to another facility………………………05<br>Given no information or referral………………………06<br>Other_________________________________96	
Q35.	[SEE Q11A (22) FAMILY PLANNING COUNSELING & SERVICES] → **IF FP <u>IS OFFERED</u>**,<br><br>YES, ☐ ↓	[SEE Q11A (22) FAMILY PLANNING COUNSELING & SERVICES] → **IF FP IS <u>NOT OFFERED</u>**,<br><br>NO, ☐ ↓<br><br>Would FP counseling and services be appropriate to include into the existing services offered?<br><br>Yes . . . . 1<br>No . . . . 2<br>Don't know . . . . .8 } →	**ALL SKIP TO Q65**

ASK IF THE FOLLOWING CONTRACEPTIVES ARE PROVIDED IN THIS FACILITY. FOR EACH ITEM, CIRCLE THE APPROPRIATE CODE.					
METHOD	Q36a. Does this facility provide the following FP methods/ services?	Q36b. How many days per week is the method provided?	Q36c. What year was METHOD first offered at this facility?<br><br>**Don't know = 9998**	Q36d. Are there requirements for partner's consent to receive the following METHOD?	Q36e. How many staff do you have that can provide METHOD?
(01) Combined oral pill	YES…..1<br>NO…….2 → **(02)**	Days… [ ]	[ ][ ][ ][ ]	YES . . . . . .1<br>NO 2	
(02) Progestin only pill	YES…..1<br>NO…….2 → **(03)**	Days… [ ]	[ ][ ][ ][ ]	YES . . . . . .1<br>NO 2	
(03) Emergency contraceptive	YES…..1<br>NO…….2 → **(04)**	Days… [ ]	[ ][ ][ ][ ]	YES . . . . . .1<br>NO 2	
(04) Male condom	YES…..1<br>NO…….2 → **(05)**	Days… [ ]	[ ][ ][ ][ ]	YES . . . . . .1<br>NO 2	
(05) Female condom	YES…..1<br>NO…….2 → **(06)**	Days… [ ]	[ ][ ][ ][ ]	YES . . . . . .1<br>NO 2	
(06) Injectables	YES…..1<br>NO…….2 → **(07)**	Days… [ ]	[ ][ ][ ][ ]	YES . . . . . .1<br>NO 2	
(07) Implants (Jadelle/ Implanon)	YES…..1<br>NO…….2 → **(08)**	Days… [ ]	[ ][ ][ ][ ]	YES . . . . . .1<br>NO 2	**[___|___|___]**
(08) IUCD	YES…..1<br>NO…….2 → **(09)**	Days… [ ]	[ ][ ][ ][ ]	YES . . . . . .1<br>NO 2	**[___|___|___]**
(09) Female sterilization/ tubal ligation	YES…..1<br>NO…….2 → **(10)**	Days… [ ]	[ ][ ][ ][ ]	YES . . . . . .1<br>NO 2	**[___|___|___]**
(10) Male sterilization	YES…..1<br>NO…….2 → **(11)**	Days… [ ]	[ ][ ][ ][ ]	YES . . . . . .1<br>NO 2	**[___|___|___]**
(11) SDM/Cycle beads	YES…..1<br>NO…….2 → **(12)**	Days… [ ]	[ ][ ][ ][ ]	YES . . . . . .1<br>NO 2	
(12) LAM	YES…..1<br>NO…….2 → **(13)**	Days… [ ]	[ ][ ][ ][ ]	YES . . . . . .1<br>NO 2	
(13) Other (specify) ____________	YES…..1<br>NO…….2 → **(Q37a)**	Days… [ ]	[ ][ ][ ][ ]	YES . . . . . .1<br>NO 2	

**ONLY ASK ABOUT THOSE METHODS THAT ARE OFFERED AT THE FACILITY FROM Q36a.**		
METHOD	Q37a. How many [NAMED METHOD] do you usually provide to a **new acceptor** on her first visit?	Q37b. How many [NAMED METHOD] do you usually provide to a woman coming for **resupply/continuing to use the same method**?
(01) Combined oral contraceptives (number of cycles)		
(02) Progestin-only oral contraceptives (number of cycles)		
(03) Male condoms (number of pieces)		
(04) Female condoms (number of pieces)		

Now I'm going to ask you some questions related to how much clients pay for contraceptive services and methods.<br>**ONLY ASK ABOUT THOSE METHODS THAT ARE OFFERED BY THE FACILITY FROM Q36a.**				
METHOD	Q38a. How much is the consultation fee (in Naira) for METHOD/PROCEDURE?<br><br>OR<br><br>Package Deal (both consult and method/procedure)	Q38b. Do fees for METHOD vary depending on the product available?	Q38c. How much is the METHOD/PROCEDURE?<br><br>RECORD THE RANGE (in Naira) IF PRICE DIFFERS BY BRAND **FROM LOWEST TO HIGHEST PRICE.**<br><br>RECORD THE PRICE IN THE FIRST FIELD IF THERE IS ONLY ONE PRODUCT OR IF THE PRICE DOES NOT DIFFER BY BRAND	CHECK – IF OPTION Q38A AND Q38C is "FREE", GO TO NEXT METHOD<br><br>Q38d. What percent of clients pay the charge for METHOD/ PROCEDURE?
(01) Combined oral pill	CONSULTATION….1 [___|___|___|___|___]<br>FREE……………..999995<br>DON'T KNOW…….999998<br><br>OR<br>PACKAGE DEAL…..2 [___|___|___|___|___] → **Q38d**	Yes . . . ……………….1<br>No/only one brand or product available…... 2<br>Don't know …………8	[___|___|___|___]<br>TO<br>[___|___|___|___]per cycle<br><br>PRESCRIPTION/REFERRAL ONLY……..9994<br>FREE…………………………,……………9995<br>DON'T KNOW…………..………….……..9998	[___|___|___]
(02) Progestin only pill	CONSULTATION….1 [___|___|___|___|___]<br>FREE……………..999995<br>DON'T KNOW…….999998<br><br>OR<br>PACKAGE DEAL…..2 [___|___|___|___|___] → **Q38d**	Yes . . . ……………….1<br>No/only one brand or product available…... 2<br>Don't know …………8	[___|___|___|___]<br>TO<br>[___|___|___|___]per cycle<br><br>PRESCRIPTION/REFERRAL ONLY……..9994<br>FREE…………………………,……………9995<br>DON'T KNOW…………..…………………..9998	[___|___|___]
(03) Emergency contraceptive	CONSULTATION….1 [___|___|___|___|___]<br>FREE……………..999995<br>DON'T KNOW…….999998<br><br>OR<br>PACKAGE DEAL…..2 [___|___|___|___|___] → **Q38d**	Yes . . . ……………….1<br>No/only one brand or product available…... 2<br>Don't know …………8	[___|___|___|___]<br>TO<br>[___|___|___|___]per package/cycle<br><br>PRESCRIPTION/REFERRAL ONLY……..9994<br>FREE…………………………,……………9995<br>DON'T KNOW…………..………….……..9998	[___|___|___]

Now I'm going to ask you some questions related to how much clients pay for contraceptive services and methods.<br>**ONLY ASK ABOUT THOSE METHODS THAT ARE OFFERED BY THE FACILITY FROM Q36a.**				
METHOD	Q38a. How much is the consultation fee (in Naira) for METHOD/PROCEDURE?<br><br>OR<br><br>Package Deal (both consult and method/procedure)	Q38b. Do fees for METHOD vary depending on the product available?	Q38c. How much is the METHOD/PROCEDURE?<br><br>RECORD THE RANGE (in Naira) IF PRICE DIFFERS BY BRAND **FROM LOWEST TO HIGHEST PRICE.**<br><br>RECORD THE PRICE IN THE FIRST FIELD IF THERE IS ONLY ONE PRODUCT OR IF THE PRICE DOES NOT DIFFER BY BRAND	CHECK – IF OPTION Q38A AND Q38C is "FREE", GO TO NEXT METHOD<br><br>Q38d. What percent of clients pay the charge for METHOD/ PROCEDURE?
(04) Male condom	CONSULTATION….1 [___|___|___|___|___]<br>FREE……………..999995<br>DON'T KNOW…….999998<br><br>OR<br>PACKAGE DEAL…..2 [___|___|___|___|___] → **Q38d**	Yes . . . . ……………..1<br>No/only one brand or product available…... 2<br>Don't know …………8	[___|___|___|___]<br>TO<br>[___|___|___|___]per piece<br><br>PRESCRIPTION/REFERRAL ONLY……..9994<br>FREE……………………….,……………9995<br>DON'T KNOW…………...……..………..…9998	[___|___|___]
(05) Female condom	CONSULTATION….1 [___|___|___|___|___]<br>FREE……………..999995<br>DON'T KNOW…….999998<br><br>OR<br>PACKAGE DEAL…..2 [___|___|___|___|___] → **Q38d**	Yes . . . . ……………..1<br>No/only one brand or product available…... 2<br>Don't know …………8	[___|___|___|___]<br>TO<br>[___|___|___|___]per piece<br><br>PRESCRIPTION/REFERRAL ONLY……..9994<br>FREE……………………….,……………9995<br>DON'T KNOW…………...……..………..…9998	[___|___|___]
(06) Injectables [Depo (DMPA), Noristorat]	CONSULTATION….1 [___|___|___|___|___]<br>FREE……………..999995<br>DON'T KNOW…….999998<br><br>OR<br>PACKAGE DEAL…..2 [___|___|___|___|___] → **Q38d**	Yes . . . . ……………..1<br>No/only one brand or product available…... 2<br>Don't know …………8	[___|___|___|___]<br>TO<br>[___|___|___|___]per injectable<br><br>PRESCRIPTION/REFERRAL ONLY……..9994<br>FREE……………………….,……………9995<br>DON'T KNOW…………...……..………..…9998	[___|___|___]
(07) Implants (Jadelle/Implan on)	CONSULTATION….1 [___|___|___|___|___]<br>FREE……………..999995<br>DON'T KNOW…….999998<br><br>OR<br>PACKAGE DEAL…..2 [___|___|___|___|___] → **Q38d**	Yes . . . . ……………..1<br>No/only one brand or product available…... 2<br>Don't know …………8	[___|___|___|___|___]<br>TO<br>[___|___|___|___|___]per implant<br><br>PRESCRIPTION/REFERRAL ONLY…...99994<br>FREE……………………….,………..…99995<br>DON'T KNOW………...…………………99998	[___|___|___]

Now I'm going to ask you some questions related to how much clients pay for contraceptive services and methods.<br>**ONLY ASK ABOUT THOSE METHODS THAT ARE OFFERED BY THE FACILITY FROM Q36a.**				
METHOD	Q38a. How much is the consultation fee (in Naira) for METHOD/PROCEDURE?<br><br>OR<br><br>Package Deal (both consult and method/procedure)	Q38b. Do fees for METHOD vary depending on the product available?	Q38c. How much is the METHOD/PROCEDURE?<br><br>RECORD THE RANGE (in Naira) IF PRICE DIFFERS BY BRAND **FROM LOWEST TO HIGHEST PRICE.**<br><br>RECORD THE PRICE IN THE FIRST FIELD IF THERE IS ONLY ONE PRODUCT OR IF THE PRICE DOES NOT DIFFER BY BRAND	CHECK – IF OPTION Q38A AND Q38C is "FREE", GO TO NEXT METHOD<br><br>Q38d. What percent of clients pay the charge for METHOD/ PROCEDURE?
(08) IUCD	CONSULTATION….1 [___|___|___|___|___]<br>FREE……………..999995<br>DON'T KNOW….…999998<br><br>OR<br>PACKAGE DEAL…..2 [___|___|___|___|___] → **Q38d**	Yes . . . ……………..1<br>No/only one brand or product available…... 2<br>Don't know …………8	[___|___|___|___|___]<br>TO<br>[___|___|___|___|___]per IUD<br><br>PRESCRIPTION/REFERRAL ONLY…..99994<br>FREE………………………,…………99995<br>DON'T KNOW…………..……...………..99998	[___|___|___]
(09) Female sterilization/ tubal ligation	CONSULTATION….1 [___|___|___|___|___]<br>FREE……………..999995<br>DON'T KNOW….…999998<br><br>OR<br>PACKAGE DEAL…..2 [___|___|___|___|___] → **Q38d**		[___|___|___|___|___]<br>TO<br>[___|___|___|___|___] PER OPERATION<br><br>REFERRAL ONLY……………………...99994<br>FREE……………………,……………99995<br>DON'T KNOW…………..………………..99998	[___|___|___]
(10) Male sterilization	CONSULTATION….1 [___|___|___|___|___]<br>FREE……………..999995<br>DON'T KNOW….…999998<br><br>OR<br>PACKAGE DEAL…..2 [___|___|___|___|___] → **Q38d**		[___|___|___|___|___]<br>TO<br>[___|___|___|___|___] PER OPERATION<br><br>REFERRAL ONLY…………………..…..99994<br>FREE……………………,…………..99995<br>DON'T KNOW…………..……………….99998	[___|___|___]
(11) SDM/Cycle beads	CONSULTATION….1 [___|___|___|___|___]<br>FREE……………..999995<br>DON'T KNOW…….999998<br><br>OR<br>PACKAGE DEAL…..2 [___|___|___|___|___] → **Q38d**	Yes . . . ……………..1<br>No/only one brand or product available… . . 2<br>Don't know ……..…8	[___|___|___|___]<br>TO<br>[___|___|___|___]per UNIT<br><br>PRESCRIPTION/REFERRAL ONLY….9994<br>FREE……………………,……..………9995<br>DON'T KNOW……..…………………..……9998	[___|___|___]

Now I'm going to ask you some questions related to how much clients pay for contraceptive services and methods.<br>**ONLY ASK ABOUT THOSE METHODS THAT ARE OFFERED BY THE FACILITY FROM Q36a.**				
METHOD	Q38a. How much is the consultation fee (in Naira) for METHOD/PROCEDURE?<br><br>OR<br><br>Package Deal (both consult and method/procedure)	Q38b. Do fees for METHOD vary depending on the product available?	Q38c. How much is the METHOD/PROCEDURE?<br><br>RECORD THE RANGE (in Naira) IF PRICE DIFFERS BY BRAND **FROM LOWEST TO HIGHEST PRICE.**<br><br>RECORD THE PRICE IN THE FIRST FIELD IF THERE IS ONLY ONE PRODUCT OR IF THE PRICE DOES NOT DIFFER BY BRAND	CHECK – IF OPTION Q38A AND Q38C is "FREE", GO TO NEXT METHOD<br><br>Q38d. What percent of clients pay the charge for METHOD/ PROCEDURE?
(12) Other (specify)<br>_______________	CONSULTATION….1 [___|___|___|___|___]<br>FREE……………..999995<br>DON'T KNOW…….999998<br><br>OR<br>PACKAGE DEAL…..2 [___|___|___|___|___] → **Q38d**	Yes . . . ……………..1<br>No/only one brand or product available… . . 2<br>Don't know ……..…8	[___|___|___|___]<br>TO<br>[___|___|___|___]per UNIT<br><br>PRESCRIPTION/REFERRAL ONLY….9994<br>FREE…………………….,……..………9995<br>DON'T KNOW…………………….……9998	[___|___|___]

**SERVICE STATISTICS** Now I want to ask about service statistics for the following contraceptive methods. For each method I ask about, please tell me the number of new acceptors/users and the number of resupply/continuing users for both the last month and the last 12 months.		
**Q39a**.<br>How many clients received family planning services in the ***last 12 completed months?***<br><br>[___|___|___|___|___|___]<br><br>NOT AVAILABLE..999993 ⟶**Q40a**	**Q39b.**<br>RECORD THE NUMBER OF MONTHS OF DATA REPRESENTED IN Q39a<br><br>[ ][ ]	**Q39c**.<br>INDICATE FROM WHERE STATISTICS COME :<br><br>OBSERVED, COUNTED IN REGISTER……..…………….…1<br>OBSERVED, SUMMED FROM REGISTER MONTHLY TOTALS..2<br>OBSERVED, SUMMED FROM MONTHLY REPORTS…………..3<br>OBSERVED, FROM YEARLY REPORT…………………………..4<br>ESTIMATED………………………5<br>OTHER___________________6<br>(SPECIFY)
**Q40a**.<br>Total new family planning acceptors/users in the ***last 12 completed months?***<br><br>[___|___|___|___|___|___]<br><br>NOTE: New acceptors/users = new to clinic and those who switch methods on day of service.<br><br>NOT AVAILABLE...999993⟶**Q41a**	**Q40b.**<br>RECORD THE NUMBER OF MONTHS OF DATA REPRESENTED IN Q40a<br><br>[ ][ ]	**Q40c**.<br>INDICATE FROM WHERE STATISTICS COME :<br><br>OBSERVED, COUNTED IN REGISTER……..…………….…1<br>OBSERVED, SUMMED FROM REGISTER MONTHLY TOTALS..2<br>OBSERVED, SUMMED FROM MONTHLY REPORTS…………..3<br>OBSERVED, FROM YEARLY REPORT…………………………..4<br>ESTIMATED………………………5<br>OTHER___________________6<br>(SPECIFY)
**Q41a**.<br>Total FP visits in the ***last 12 completed months?***<br><br>[___|___|___|___|___|___]<br><br>NOT AVAILABLE...999993⟶**Q42a**	**Q41b.**<br>RECORD THE NUMBER OF MONTHS OF DATA REPRESENTED IN Q41a<br><br>[ ][ ]	**Q41c**.<br>INDICATE FROM WHERE STATISTICS COME :<br><br>OBSERVED, COUNTED IN REGISTER……..…………….…1<br>OBSERVED, SUMMED FROM REGISTER MONTHLY TOTALS..2<br>OBSERVED, SUMMED FROM MONTHLY REPORTS…………..3<br>OBSERVED, FROM YEARLY REPORT…………………………..4<br>ESTIMATED………………………5<br>OTHER___________________6<br>(SPECIFY)

***Q42.* METHOD**<br><br>**ONLY ASK ABOUT THOSE METHODS THAT ARE OFFERED IN Q36a.**	**Q42a.**<br>Number of new acceptors / users **last month**	**Q42b**.<br>Number of resupply / continuing clients **last month**	**Q42c**.<br>INDICATE FROM WHERE STATISTICS COME FOR Q42a AND Q42b<br><br>IF BOTH Q42a AND Q42b EQUAL 9993, SKIP TO **Q42d**	**Q42d**.<br>Number of new acceptors/users **last 12 months**	**Q42e.**<br>RECORD THE NUMBER OF MONTHS OF DATA REPRESENTED IN **Q42d**	**Q42f.**<br>INDICATE FROM WHERE STATISTICS COME FOR **Q42d**	**Q42g**.<br>Number of resupply / continuing clients **last 12 months**	**Q42h.**<br>RECORD THE NUMBER OF MONTHS OF DATA REPRESENT-ED IN **Q42g**	**Q42i.**<br>INDICATE FROM WHERE STATISTICS COME FOR **Q42g**
(01) Combined oral pill	[___|___|___|___| <br>NOT AVAILABLE...9993	[___|___|___|___| <br>NOT AVAILABLE…9993	OBSERVED, COUNTED IN REGISTER……………………1<br>OBSERVED, REGISTER MONTHLY TOTAL…………...2<br>OBSERVED, FROM MONTHLY REPORT…...……3<br>ESTIMATED…………………..4<br>OTHER:________________6<br>(SPECIFY)	[___|___|___|___|___| <br>NOT AVAILABLE …99993→ **Q42g**	[___|___|	OBSERVED, COUNTED IN REGISTER………………..1<br>OBSERVED, SUMMED FROM REGISTER MONTHLY TOTALS……..2<br>OBSERVED, SUMMED FROM MONTHLY REPORTS…………………3<br>OBSERVED, FROM YEARLY REPORT……….4<br>ESTIMATED……………..…5<br>OTHER:_____________6<br>(SPECIFY)	[___|___|___|___|___| <br>NOT AVAILABLE …99993→ **(02)**	[___|___|	OBSERVED, COUNTED IN REGISTER………1<br>OBSERVED, SUMMED FROM REGISTER MONTHLY TOTALS………....2<br>OBSERVED, SUMMED FROM MONTHLY REPORTS……….3<br>OBSERVED, FROM YEARLY REPORT…………4<br>ESTIMATED……..5<br>OTHER:________6<br>(SPECIFY)
(02) Progestin only pill	[___|___|___|___| <br>NOT AVAILABLE…9993	[___|___|___|___| <br>NOT AVAILABLE...9993	OBSERVED, COUNTED IN REGISTER……………………1<br>OBSERVED, REGISTER MONTHLY TOTAL…………...2<br>OBSERVED, FROM MONTHLY REPORT…...……3<br>ESTIMATED…………………..4<br>OTHER:________________6<br>(SPECIFY)	[___|___|___|___|___| <br>NOT AVAILABLE …99993 → **Q42g**	[___|___|	OBSERVED, COUNTED IN REGISTER………………..1<br>OBSERVED, SUMMED FROM REGISTER MONTHLY TOTALS……..2<br>OBSERVED, SUMMED FROM MONTHLY REPORTS…………………3<br>OBSERVED, FROM YEARLY REPORT……….4<br>ESTIMATED……………..…5<br>OTHER:_____________6<br>(SPECIFY)	[___|___|___|___|___| <br>NOT AVAILABLE …99993→ **(03)**	[___|___|	OBSERVED, COUNTED IN REGISTER………1<br>OBSERVED, SUMMED FROM REGISTER MONTHLY TOTALS………....2<br>OBSERVED, SUMMED FROM MONTHLY REPORTS……….3<br>OBSERVED, FROM YEARLY REPORT…………4<br>ESTIMATED……..5<br>OTHER:________6<br>(SPECIFY)

Q42. **METHOD**<br><br>**ONLY ASK ABOUT THOSE METHODS THAT ARE OFFERED IN Q36a.**	**Q42a.**<br>Number of new acceptors / users **last month**	**Q42b**.<br>Number of resupply / continuing clients **last month**	**Q42c**.<br>INDICATE FROM WHERE STATISTICS COME FOR Q42a AND Q42b<br><br>IF BOTH Q42a AND Q42b EQUAL 9993, SKIP TO **Q42d**	**Q42d**.<br>Number of new acceptors/users **last 12 months**	**Q42e.**<br>RECORD THE NUMBER OF MONTHS OF DATA REPRESENTED IN **Q42d**	**Q42f.**<br>INDICATE FROM WHERE STATISTICS COME FOR **Q42d**	**Q42g**.<br>Number of resupply / continuing clients **last 12 months**	**Q42h.**<br>RECORD THE NUMBER OF MONTHS OF DATA REPRESENT-ED IN **Q42g**	**Q42i.**<br>INDICATE FROM WHERE STATISTICS COME FOR **Q42g**
(03) Emergency contraceptive	[___|___|___|___| <br>NOT AVAILABLE...9993	[___|___|___|___| <br>NOT AVAILABLE...9993	OBSERVED, COUNTED IN REGISTER……………………1<br>OBSERVED, REGISTER MONTHLY TOTAL…………...2<br>OBSERVED, FROM MONTHLY REPORT…...……3<br>ESTIMATED…………………..…4<br>OTHER:________________6<br>(SPECIFY)	[___|___|___|___|___| <br>NOT AVAILABLE …99993 → **Q42g**	[___|___|	OBSERVED, COUNTED IN REGISTER………………..…1<br>OBSERVED, SUMMED FROM REGISTER MONTHLY TOTALS……...2<br>OBSERVED, SUMMED FROM MONTHLY REPORTS…………………3<br>OBSERVED, FROM YEARLY REPORT……….4<br>ESTIMATED……………..….5<br>OTHER:_____________6<br>(SPECIFY)	[___|___|___|___|___| <br>NOT AVAILABLE …99993 → **(04)**	[___|___|	OBSERVED, COUNTED IN REGISTER………1<br>OBSERVED, SUMMED FROM REGISTER MONTHLY TOTALS………....2<br>OBSERVED, SUMMED FROM MONTHLY REPORTS……….3<br>OBSERVED, FROM YEARLY REPORT…………4<br>ESTIMATED……..5<br>OTHER:________6<br>(SPECIFY)
(04) Male condom	[___|___|___|___| <br>NOT AVAILABLE...9993	[___|___|___|___| <br>NOT AVAILABLE...9993	OBSERVED, COUNTED IN REGISTER……………………1<br>OBSERVED, REGISTER MONTHLY TOTAL…………...2<br>OBSERVED, FROM MONTHLY REPORT…...……3<br>ESTIMATED…………………..…4<br>OTHER:________________6<br>(SPECIFY)	[___|___|___|___|___| <br>NOT AVAILABLE …99993 → **Q42g**	[___|___|	OBSERVED, COUNTED IN REGISTER………………..…1<br>OBSERVED, SUMMED FROM REGISTER MONTHLY TOTALS……...2<br>OBSERVED, SUMMED FROM MONTHLY REPORTS…………………3<br>OBSERVED, FROM YEARLY REPORT……….4<br>ESTIMATED……………..….5<br>OTHER:_____________6<br>(SPECIFY)	[___|___|___|___|___| <br>NOT AVAILABLE …99993 → **(05)**	[___|___|	OBSERVED, COUNTED IN REGISTER………1<br>OBSERVED, SUMMED FROM REGISTER MONTHLY TOTALS………....2<br>OBSERVED, SUMMED FROM MONTHLY REPORTS……….3<br>OBSERVED, FROM YEARLY REPORT…………4<br>ESTIMATED……..5<br>OTHER:________6<br>(SPECIFY)
(05) Female condom	[___|___|___|___| <br>NOT AVAILABLE...9993	[___|___|___|___| <br>NOT AVAILABLE...9993	OBSERVED, COUNTED IN REGISTER……………………1<br>OBSERVED, REGISTER MONTHLY TOTAL…………...2<br>OBSERVED, FROM MONTHLY REPORT…...……3<br>ESTIMATED…………………..…4<br>OTHER:________________6<br>(SPECIFY)	[___|___|___|___|___| <br>NOT AVAILABLE …99993 → **Q42g**	[___|___|	OBSERVED, COUNTED IN REGISTER………………..…1<br>OBSERVED, SUMMED FROM REGISTER MONTHLY TOTALS……...2<br>OBSERVED, SUMMED FROM MONTHLY REPORTS…………………3<br>OBSERVED, FROM YEARLY REPORT……….4<br>ESTIMATED……………..….5<br>OTHER:_____________6<br>(SPECIFY)	[___|___|___|___|___| <br>NOT AVAILABLE …99993 → **(06)**	[___|___|	OBSERVED, COUNTED IN REGISTER………1<br>OBSERVED, SUMMED FROM REGISTER MONTHLY TOTALS………....2<br>OBSERVED, SUMMED FROM MONTHLY REPORTS……….3<br>OBSERVED, FROM YEARLY REPORT…………4<br>ESTIMATED……..5<br>OTHER:________6<br>(SPECIFY)

Q42. **METHOD**<br><br>**ONLY ASK ABOUT THOSE METHODS THAT ARE OFFERED IN Q36a.**	**Q42a.**<br>Number of new acceptors / users **last month**	**Q42b**.<br>Number of resupply / continuing clients **last month**	**Q42c**.<br>INDICATE FROM WHERE STATISTICS COME FOR Q42a AND Q42b<br><br>IF BOTH Q42a AND Q42b EQUAL 9993, SKIP TO **Q42d**	**Q42d**.<br>Number of new acceptors/users **last 12 months**	**Q42e.**<br>RECORD THE NUMBER OF MONTHS OF DATA REPRESENTED IN **Q42d**	**Q42f.**<br>INDICATE FROM WHERE STATISTICS COME FOR **Q42d**	**Q42g**.<br>Number of resupply / continuing clients **last 12 months**	**Q42h.**<br>RECORD THE NUMBER OF MONTHS OF DATA REPRESENT-ED IN **Q42g**	**Q42i.**<br>INDICATE FROM WHERE STATISTICS COME FOR **Q42g**
(06) Injectables (Depo/ Noristerat)	[___|___|___|___]<br><br>NOT AVAILABLE...9993	[___|___|___|___]<br><br>NOT AVAILABLE...9993	OBSERVED, COUNTED IN REGISTER……………………1<br>OBSERVED, REGISTER MONTHLY TOTAL…………..2<br>OBSERVED, FROM MONTHLY REPORT…..……3<br>ESTIMATED……………….…..4<br>OTHER:________________6<br>(SPECIFY)	[___|___|___|___|___]<br><br>NOT AVAILABLE …99993 → **Q42g**	[___|___]	OBSERVED, COUNTED IN REGISTER……………..…1<br>OBSERVED, SUMMED FROM REGISTER MONTHLY TOTALS……..2<br>OBSERVED, SUMMED FROM MONTHLY REPORTS…………………3<br>OBSERVED, FROM YEARLY REPORT……….4<br>ESTIMATED…………..….5<br>OTHER:_____________6<br>(SPECIFY)	[___|___|___|___|___]<br><br>NOT AVAILABLE …99993 → **(07)**	[___|___]	OBSERVED, COUNTED IN REGISTER………1<br>OBSERVED, SUMMED FROM REGISTER MONTHLY TOTALS………....2<br>OBSERVED, SUMMED FROM MONTHLY REPORTS……….3<br>OBSERVED, FROM YEARLY REPORT…………4<br>ESTIMATED……..5<br>OTHER:________6<br>(SPECIFY)
(07) Implants (Jadelle/ Implanon)	[___|___|___|___]<br><br>NOT AVAILABLE...9993	[___|___|___|___]<br><br>NOT AVAILABLE...9993	OBSERVED, COUNTED IN REGISTER……………………1<br>OBSERVED, REGISTER MONTHLY TOTAL…………..2<br>OBSERVED, FROM MONTHLY REPORT…..……3<br>ESTIMATED……………….…..4<br>OTHER:________________6<br>(SPECIFY)	[___|___|___|___|___]<br><br>NOT AVAILABLE …99993 → **Q42g**	[___|___]	OBSERVED, COUNTED IN REGISTER……………..…1<br>OBSERVED, SUMMED FROM REGISTER MONTHLY TOTALS……..2<br>OBSERVED, SUMMED FROM MONTHLY REPORTS…………………3<br>OBSERVED, FROM YEARLY REPORT……….4<br>ESTIMATED…………..….5<br>OTHER:_____________6<br>(SPECIFY)	[___|___|___|___|___]<br><br>NOT AVAILABLE …99993 → **(08)**	[___|___]	OBSERVED, COUNTED IN REGISTER………1<br>OBSERVED, SUMMED FROM REGISTER MONTHLY TOTALS………....2<br>OBSERVED, SUMMED FROM MONTHLY REPORTS……….3<br>OBSERVED, FROM YEARLY REPORT…………4<br>ESTIMATED……..5<br>OTHER:________6<br>(SPECIFY)
(08) IUCD	[___|___|___|___]<br><br>NOT AVAILABLE...9993	[___|___|___|___]<br><br>NOT AVAILABLE...9993	OBSERVED, COUNTED IN REGISTER……………………1<br>OBSERVED, REGISTER MONTHLY TOTAL…………..2<br>OBSERVED, FROM MONTHLY REPORT…..……3<br>ESTIMATED……………….…..4<br>OTHER:________________6<br>(SPECIFY)	[___|___|___|___|___]<br><br>NOT AVAILABLE …99993 → **Q42g**	[___|___]	OBSERVED, COUNTED IN REGISTER……………..…1<br>OBSERVED, SUMMED FROM REGISTER MONTHLY TOTALS……..2<br>OBSERVED, SUMMED FROM MONTHLY REPORTS…………………3<br>OBSERVED, FROM YEARLY REPORT……….4<br>ESTIMATED…………..….5<br>OTHER:_____________6<br>(SPECIFY)	[___|___|___|___|___]<br><br>NOT AVAILABLE …99993 → **(09)**	[___|___]	OBSERVED, COUNTED IN REGISTER………1<br>OBSERVED, SUMMED FROM REGISTER MONTHLY TOTALS………....2<br>OBSERVED, SUMMED FROM MONTHLY REPORTS……….3<br>OBSERVED, FROM YEARLY REPORT…………4<br>ESTIMATED……..5<br>OTHER:________6<br>(SPECIFY)

Q42. **METHOD**<br><br>**ONLY ASK ABOUT THOSE METHODS THAT ARE OFFERED IN Q36a.**	**Q42a.**<br>Number of new acceptors / users **last month**	**Q42b**.<br>Number of resupply / continuing clients **last month**	**Q42c**.<br>INDICATE FROM WHERE STATISTICS COME FOR Q42a AND Q42b<br><br>IF BOTH Q42a AND Q42b EQUAL 9993, SKIP TO **Q42d**	**Q42d**.<br>Number of new acceptors/users **last 12 months**	**Q42e.**<br>RECORD THE NUMBER OF MONTHS OF DATA REPRESENTED IN **Q42d**	**Q42f.**<br>INDICATE FROM WHERE STATISTICS COME FOR **Q42d**	**Q42g**.<br>Number of resupply / continuing clients **last 12 months**	**Q42h.**<br>RECORD THE NUMBER OF MONTHS OF DATA REPRESENT-ED IN **Q42g**	**Q42i.**<br>INDICATE FROM WHERE STATISTICS COME FOR **Q42g**
(09) Female sterilization	[___|___|___|___| <br>NOT AVAILABLE……… 9993 ➔ **Q42d**		OBSERVED, COUNTED IN REGISTER……………………1<br>OBSERVED, REGISTER MONTHLY TOTAL…………...2<br>OBSERVED, FROM MONTHLY REPORT…...……3<br>ESTIMATED……………….….4<br>OTHER:________________6<br>(SPECIFY)	[___|___|___|___|___| <br>NOT AVAILABLE …99993 ➔ **(10)**	[___|___|	OBSERVED, COUNTED IN REGISTER……………...…1<br>OBSERVED, SUMMED FROM REGISTER MONTHLY TOTALS……...2<br>OBSERVED, SUMMED FROM MONTHLY REPORTS…………………3<br>OBSERVED, FROM YEARLY REPORT……….4<br>ESTIMATED…………...….5<br>OTHER:_____________6<br>(SPECIFY)			
(10) Male sterilization	[___|___|___|___| <br>NOT AVAILABLE……… 9993 ➔ **Q42d**		OBSERVED, COUNTED IN REGISTER……………………1<br>OBSERVED, REGISTER MONTHLY TOTAL…………...2<br>OBSERVED, FROM MONTHLY REPORT…...……3<br>ESTIMATED……………….….4<br>OTHER:________________6<br>(SPECIFY)	[___|___|___|___|___| <br>NOT AVAILABLE …99993 ➔ **(11)**	[___|___|	OBSERVED, COUNTED IN REGISTER……………...…1<br>OBSERVED, SUMMED FROM REGISTER MONTHLY TOTALS……...2<br>OBSERVED, SUMMED FROM MONTHLY REPORTS…………………3<br>OBSERVED, FROM YEARLY REPORT……….4<br>ESTIMATED…………...….5<br>OTHER:_____________6<br>(SPECIFY)			
(11) SDM/Cycle beads	[___|___|___|___| <br>NOT AVAILABLE……… 9993➔ **Q42d**		OBSERVED, COUNTED IN REGISTER……………………1<br>OBSERVED, REGISTER MONTHLY TOTAL…………...2<br>OBSERVED, FROM MONTHLY REPORT…...……3<br>ESTIMATED……………….….4<br>OTHER:________________6<br>(SPECIFY)	[___|___|___|___|___| <br>NOT AVAILABLE …99993 ➔ **(12)**	[___|___|	OBSERVED, COUNTED IN REGISTER……………...…1<br>OBSERVED, SUMMED FROM REGISTER MONTHLY TOTALS……...2<br>OBSERVED, SUMMED FROM MONTHLY REPORTS…………………3<br>OBSERVED, FROM YEARLY REPORT……….4<br>ESTIMATED…………...….5<br>OTHER:_____________6<br>(SPECIFY)			
(12) LAM	[___|___|___|___| <br>NOT AVAILABLE……… 9993➔ **Q42d**		OBSERVED, COUNTED IN REGISTER……………………1<br>OBSERVED, REGISTER MONTHLY TOTAL…………...2<br>OBSERVED, FROM MONTHLY REPORT…...……3<br>ESTIMATED……………….….4<br>OTHER:________________6<br>(SPECIFY)	[___|___|___|___|___| <br>NOT AVAILABLE …99993 ➔ **(13)**	[___|___|	OBSERVED, COUNTED IN REGISTER……………...…1<br>OBSERVED, SUMMED FROM REGISTER MONTHLY TOTALS……...2<br>OBSERVED, SUMMED FROM MONTHLY REPORTS…………………3<br>OBSERVED, FROM YEARLY REPORT……….4<br>ESTIMATED…………...….5<br>OTHER:_____________6<br>(SPECIFY)			

***Q42.* METHOD**<br><br>**ONLY ASK ABOUT THOSE METHODS THAT ARE OFFERED IN Q36a.**	**Q42a.**<br>Number of new acceptors / users **last month**	**Q42b**.<br>Number of resupply / continuing clients **last month**	**Q42c**.<br>INDICATE FROM WHERE STATISTICS COME FOR Q42a AND Q42b<br><br>IF BOTH Q42a AND Q42b EQUAL 9993, SKIP TO **Q42d**	**Q42d**.<br>Number of new acceptors/users **last 12 months**	**Q42e.**<br>RECORD THE NUMBER OF MONTHS OF DATA REPRESENTED IN **Q42d**	**Q42f.**<br>INDICATE FROM WHERE STATISTICS COME FOR **Q42d**	**Q42g**.<br>Number of resupply / continuing clients **last 12 months**	**Q42h.**<br>RECORD THE NUMBER OF MONTHS OF DATA REPRESENT-ED IN **Q42g**	**Q42i.**<br>INDICATE FROM WHERE STATISTICS COME FOR **Q42g**
(13) Other (Specify)<br><br>_____________	[___|___|___|___| <br>NOT AVAILABLE…9993	[___|___|___|___| <br>NOT AVAILABLE…9993	OBSERVED, COUNTED IN REGISTER……………………1<br>OBSERVED, REGISTER MONTHLY TOTAL…………...2<br>OBSERVED, FROM MONTHLY REPORT…...……3<br>ESTIMATED…………………..…4<br>OTHER:_________________6<br>(SPECIFY)	[___|___|___|___|___| <br>NOT AVAILABLE …99993 → **(Q43)**	[___|___|	OBSERVED, COUNTED IN REGISTER………………..…1<br>OBSERVED, SUMMED FROM REGISTER MONTHLY TOTALS……...2<br>OBSERVED, SUMMED FROM MONTHLY REPORTS…………………3<br>OBSERVED, FROM YEARLY REPORT……….4<br>ESTIMATED……………..….5<br>OTHER:______________6<br>(SPECIFY)	[___|___|___|___|___| <br>NOT AVAILABLE …99993 → **(Q43)**	[___|___|	OBSERVED, COUNTED IN REGISTER………1<br>OBSERVED, SUMMED FROM REGISTER MONTHLY TOTALS………....2<br>OBSERVED, SUMMED FROM MONTHLY REPORTS……….3<br>OBSERVED, FROM YEARLY REPORT…………4<br>ESTIMATED……..5<br>OTHER:________6<br>(SPECIFY)

IEC MATERIALS AND OUTREACH ACTIVITIES					
Q43.	Are the following family planning IEC materials displayed and/or available for use?	OBSERVED	REPORTED, NOT SEEN	NOT AVAILABLE	DON'T KNOW
	a) Posters	1	2	3	8
	b) Informational flip chart	1	2	3	8
	c) Brochures/pamphlets	1	2	3	8
	d) Information sheets	1	2	3	8
	e) Job aids	1	2	3	8
	f) Demonstration models	1	2	3	8
	g) Counseling cards	1	2	3	8
	h) Samples of various FP methods	1	2	3	8
	i) Other (specify) ________________	1	2	3	8

Q44.	Does this facility have a health outreach program for IEC (Information, Education and Communication)?	Yes . . . . ….1<br>No . . . . 2 →<br>Don't know……………..…8 →	 **Q48**<br>**Q48**
Q45.	Does this outreach program discuss family planning/birth spacing?	Yes . . . . ….. 1<br>No . . . . …. 2<br>Don't know……………….8	
Q46.	How many communities are regularly visited through this outreach program?	NUMBER . . . .[___|___]	
Q47.	About how often are these communities visited through this outreach program?	WEEKLY…………………..…..…1<br>MONTHLY . . . . 2<br>QUARTERLY . . . . 3<br>ANNUALLY . . . . 4<br>OTHER ________________6<br>(Specify)	
Q48.	Does this facility give health talks for members of the community?	Yes . . . . ….. 1<br>No . . . . …. 2 →<br>Don't know…………….8 →	 **Q52**<br>**Q52**
Q49.	Has this facility ever given a health talk on family planning/birth spacing to the community?	Yes . . . .…1<br>No . . . . ….. 2<br>Don't know……………….8	
Q50.	How often does this facility give health talks to the community?	EVERY DAY . . . . 1<br>WEEKLY . . . . . ….…. . . . . . 2<br>MONTHLY……………………..3<br>QUARTERLY………………….4<br>OTHER__________________6<br>(Specify)	
Q51.	How often do the topics of the health talks change?	EVERY DAY . . . . 1<br>WEEKLY . . . . . ….…. . . . . . 2<br>MONTHLY……………………..3<br>QUARTERLY………………….4<br>OTHER__________________6<br>(Specify)	
Q52.	Does this facility supervise CBDs (community-based distributors of contraceptives)?	YES . . . . 1<br>NO . . . . 2 →	 **Q54**
Q53.	What organization sponsors the CBDs?<br><br>**CIRCLE ALL THAT APPLY**	MOH . . . .A<br>MARIE STOPES . . . . .B<br>PPFN……………………………C<br>SFH……………………………..D<br>OTHER _________________X<br>(SPECIFY)	

QUALITY ASSURANCE/STANDARD OPERATING PROCEDURES			
Now I want to ask about common quality assurance activities and guidelines. For each activity or guideline mentioned, please tell me if this exists anywhere in the facility.<br><br>IF QUALITY ASSURANCE ACTIVITIES ARE REPORTED TO BE CARRIED OUT, ASK: Can I see some document or record that shows this has been carried out during the past year?<br><br>A REPORT OR MINUTES OF A MEETING WHICH MENTIONS THE QUALITY ASSURANCE ACTIVITY IS ACCEPTABLE.			
Q54.	Are there any written guidelines or service protocols in this facility for family planning services?	Yes, document observed…………..1<br>Yes, document reported but not seen…………………………………2<br>No……………………………………..3 →<br>Don't know …………………………….8 →	 <br><br>**Q56**<br>**Q56**
Q55.	Who is the author of these guidelines or service protocols you are using?<br>NAME OF GUIDELINES:___________________________<br>______________________________________<br>______________________________________<br><br>**CHOOSE ALL THAT APPLY**	Facility created guidelines………..A<br>WHO guidelines……………………B<br>FMOH guidelines…………………..C<br><br>Other ______________________X<br>(Specify)	
Q56.	Are there any written guidelines or service protocols in this facility for the integration of family planning and HIV services?	Yes, document observed………….1<br>Yes, document reported but not seen………………………………2<br>No……………………………………3<br>Don't know ………………………….8	
Q57.	Are you using any guideline(s) or tool(s) to screen patients for pregnancy?	Yes, document observed………….1<br>Yes, document reported but not seen………………………………2<br>No……………………………………3 →<br>Don't know …………………………..8 →	 <br><br>**Q59**<br>**Q59**
Q58.	Do these guideline(s) recommend that you screen all patients for pregnancy before dispensing a new family planning method?	Yes …………………………………..1<br>No …………………………………..2<br>Other guidance provided<br>___________________________6<br>(Specify)<br>Don't know…………………………8	
Q59.	Do any of the guidelines recommend that family planning counseling is offered to most clients in this facility as a routine or normal practice?	Yes …………………………………..1<br>No …………………………………..2<br>No guidelines……….....................3<br>Other guidance provided<br>___________________________ 6<br>(Specify)<br>Don't know…………………………8	
Q60.	Are periodic audits or reports of medical records or service registers conducted/compiled at least quarterly?	Yes, document observed…………..1<br>Yes, document reported but not seen………………………………2<br>No……………………………………3<br>Don't know …………………………..8	
Q61.	Does this facility receive integrated supportive supervision visit?	Yes . . . . …..…. . . . ……..1<br>No . . . . ………….... .2 →<br>Don't know…………………………8 →	 **Q63**<br>**Q63**
Q62.	How often does this facility receive integrated supportive supervision?	Once in month ………………. 1<br>Once in three months ………. 2<br>Once every six months……….3<br>Less fequently …………………4	
Q63.	Is there any type of quality assurance committee or staff meetings that assure quality control for family planning service delivery?	Yes, document observed…………..1<br>Yes, document reported but not seen………………………………2<br>No……………………………………3<br>Don't know …………………………..8	
Q64.	Are HMIS tools made available to relevant staff?	Yes, document observed…………..1<br>Yes, document reported but not seen………………………………2<br>No……………………………………3<br>Don't know …………………………..8	

Now, I would like to ask you some questions about the physical infrastructure and equipment that you have at this facility.				
**PHYSICAL INFRASTRUCTURE AND EQUIPMENT**				
Are the following types of facilities/equipment available on a functioning basis at the service location?<br>INTERVIEWER NEEDS TO CHECK FUNCTIONING WHERE POSSIBLE.				
Q65.	DOES THIS FACILITY HAVE A SIGN POSTED WITH ITS HOURS OF OPERATION AND SERVICES?	Observed, both hours and services. . . . . .1<br>Observed, hours only………………………... . . . . 2<br>Observed, services only…………………………………………3<br>Reported, both hours and services……………………………4<br>Reported, hours only……………………………………………5<br>Reported, services only…………………………………………6<br>No sign……………………………………………………………7		
		**Not Available**	**Available but not functioning**	**Available and functioning**
Q66	Dedicated Family planning service space	1	2	3
Q67.	Electricity	1	2	3
Q68.	Back-up generator	1	2	3
Q69.	Piped water supply	1	2	3
Q70.	Toilet facilities/latrine	1	2	3
Q71.	Telephone/GSM (dedicated to the facility)	1	2	3
Q72.	Storage area for drugs and supplies	1	2	3
Q73.	Sharps container for needles	1	2	3
Q74.	Laboratory	1	2	3
Q75.	Private examination room (ie, a private room for pelvic exams and IUD insertion)	1	2	3
Q76.	Exam table for gynecological examination	1	2	3
Q77.	Examination light	1	2	3
Q78.	Delivery room with bed and lighting	1	2	3
Q79.	Operating theatre with basic/required equipment	1	2	3
Q80.	Weighing scale for adults	1	2	3
Q81.	Infant weighing scale	1	2	3
Q82.	Blood pressure apparatus	1	2	3
Q83.	Stethoscope	1	2	3
Q84.	Fetal stethoscope	1	2	3
Q85.	Sterilizer	1	2	3
Q86.	Microscope	1	2	3
Q87.	Oxygen apparatus	1	2	3
Q88.	Centrifuge	1	2	3
Q89.	Thermometer	1	2	3
Q90.	Scalpels	1	2	3
Q91.	Two pairs of scissors	1	2	3
Q92.	Long needle holder	1	2	3
Q93.	Forceps	1	2	3
Q94.	Sponge holding forceps	1	2	3
Q95.	Tenacula (Volsellum forcepts)	1	2	3
Q96.	Vaginal speculum (small size)	1	2	3
Q97.	Vaginal speculum (medium size)	1	2	3
Q98.	Vaginal speculum (large size)	1	2	3

		Not Available	Available but not functioning	Available and functioning
Q99.	Minor surgery kit (e.g. artery forceps, hemostat)	1	2	3
Q100.	Vacuum extractor	1	2	3
Q101.	Manual vacuum aspiration (MVA) kit	1	2	3
Q102.	Minilaparotomy kit	1	2	3
Q103.	Uterine hook	1	2	3
Q104.	Tubal hook	1	2	3
Q105.	Vasectomy kit	1	2	3
Q106.	Uterine sounds	1	2	3
Q107.	Canula and trochar for inserting implants	1	2	3

Now, I would like to ask you some questions about the physical infrastructure and equipment that you have at this facility.

CONSUMABLE SUPPLIES

Are the following types of supplies available on a regular basis at the service location?
INTERVIEWER NEEDS TO CHECK AVAILABILITY WHERE POSSIBLE.

		Not Available	Available sometimes but not on a regular basis	Available all of the time
Q108.	Sutures	1	2	3
Q109.	Antiseptic solution (such as iodine)	1	2	3
Q110.	Methylated spirit	1	2	3
Q111.	Sterile gauze pad or cotton wool	1	2	3
Q112.	Sterile disposable latex gloves	1	2	3
Q113.	Long gloves	1	2	3
Q114.	Disposable sterile syringes and needles	1	2	3
Q115.	Intravenous kit	1	2	3
Q116.	Scalpel blades	1	2	3
Q117.	Sealed implants pack (for performing FP implant insertions)	1	2	3
Q118.	Sedatives (such as Valium)	1	2	3
Q119.	Atropine (such as Buscopan)	1	2	3
Q120.	Opioid analgesic	1	2	3
Q121.	Local anesthetic (such as lignocaine)	1	2	3

Q122	CHECK Q11A(22):<br><br>FACILITY PROVIDES FP SERVICES (Q11A(22)=YES) ☐ ↓	FACILITY DOES NOT PROVIDE FP SERVICES (Q11A(22)=NO) ☐ →	**Q132**
**STORAGE:** Now I would like to see the place where contraceptive methods are stored. We are just trying to get an idea of how facilities keep their stock and store contraceptive methods. Remember that my findings will be just used for research purposes and will be kept strictly confidential.			
Q123.	OBSERVE WHETHER ALL THE CONTRACEPTIVE METHODS ARE PROTECTED FROM WATER OR DAMPNESS	YES . . . . 1<br>NO . . . . 2<br>CANNOT OBSERVE STORAGE AREA………………..………………..3 →	**Q129**
Q124.	OBSERVE WHETHER ALL THE CONTRACEPTIVE METHODS ARE OFF THE FLOOR	YES . . . . 1<br>NO 2	
Q125.	OBSERVE WHETHER THE CEILING ABOVE THE CONTRACEPTIVE METHODS IS INTACT AND NOT LEAKING	YES . . . . 1<br>NO 2	
Q126.	OBSERVE WHETHER ALL THE CONTRACEPTIVE METHODS ARE PROTECTED FROM THE SUN.	YES . . . . 1<br>NO 2	
Q127.	OBSERVE WHETHER THE ROOM IS CLEAN OF EVIDENCE OF RODENTS (BATS, RATS) OR PESTS (ROACHES, ETC).	YES . . . . 1<br>NO 2	
Q128.	OBSERVE WHETHER THE INJECTABLES ARE STORED UPRIGHT.	YES . . . . 1<br>NO . . . . 2<br>NOT APPLICABLE/DON'T PROVIDE INJECTABLES……….7	
Q129.	Does the pharmacy separate damaged and/or expired family planning methods from the usable products, and remove them from the inventory?<br><br>IF YES, ASK TO SEE EVIDENCE OF EACH OF THE INDICATED PRACTICES AND ALL THAT WERE OBSERVED. ALSO ASK FOR THE TALLY CARD TO CHECK FOR RECORDED BALANCE.	YES, DAMAGED/EXPIRED ITEM REMOVED FROM INVENTORY . . . ………………….. …... . . . . 1<br><br>REMOVED FROM SHELVES AND NO EXPIRED ITEMS PRESENT………………………………..….. ... . 2<br><br>EXPIRED ITEMS OBSERVED . . . . . . ……. .. 3<br><br>REPORTED YES BUT CANNOT OBSERVE….4<br><br>NO 5	

Now I would like to ask you about your specific stocks of different family planning methods/products.
ONLY ASK ABOUT THOSE METHODS THAT ARE AVAILABLE FROM Q36a.

CONTRACEPTIVE	Q130a. Where does your stock of CONTRACEPTIVE come from? CHOOSE ALL.	Q130b. When was the last time that you received a routine supply of CONTRACEPTIVE either that you ordered, or that is part of your routine supply system? READ LIST.	Q130c. Does this facility determine the quantity of each CONTRACEPTIVE that it needs and order that, or is the quantity that you receive determined elsewhere? READ LIST.	Q130d. Do you always receive a standard fixed quantity of CONTRACEPTIVE or does the quantity you receive vary according to recent need or activity level? READ LIST.	Q130e. **CHECK Q130C. IFQ130C IS "2", SKIP TO Q130G**<br>When you order CONTRACEPTIVE, how much do you order?<br><br>READ LIST.	Q130f. When do you decide to order CONTRACEPTIVE?<br><br>READ LIST.	Q130g. On average, how long does it take to receive your supplies after you have placed an order?<br><br>READ LIST.	Q130h. Is METHOD usually delivered or must you go get them?
(01) Combined oral contraceptives (estrogen and progestin)	Govt…………… A<br>Intl NGO ……… B<br>Local NGO…….C<br>Pharmacy wholesaler/ dealer/ distributor………D<br>Other________X<br>(Specify)<br>Don't know….….Z	< 4 wks ago….…..1<br>Between 4-12 wks………………2<br>> 12 wks ago……3<br>No routine supply system…………..4<br>Don't know………8	Determines own need…1→**(Q130e)**<br>Determined Elsewhere……..2<br>Both.….………...3<br>Don't know ………8 →**(Q130g)**	Quantity based on activity level…..1<br>Standard fixed supply…………..2<br>Don't know……...8	Order to maintain stock……………..1<br>Order same amount…………2<br>Order based on consumption…..3<br>Other__________.6<br>(Specify)<br>Don't know….…….8	Fall below predeter-mined level……….1<br>Fixed time – Every [__|__|__] days…….2<br>Order when needed..3<br>Other…………………6<br>Don't know………….8	One week or less…....1<br>Between 2-4 weeks….2<br>Between 5-8 weeks….3<br>More than 8 weeks…..4<br>Other ____________6<br>(Specify)<br><br>Don't know . …………8	Delivered …….1<br><br>Pick them up…2<br><br>Both …………..3
(02) Progestin-only oral contraceptives	Govt…………… A<br>Intl NGO ……… B<br>Local NGO…….C<br>Pharmacy wholesaler/ dealer/ distributor………D<br>Other________X<br>(Specify)<br>Don't know….….Z	< 4 wks ago….…..1<br>Between 4-12 wks………………2<br>> 12 wks ago……3<br>No routine supply system…………..4<br>Don't know………8	Determines own Need…1→**(Q130e)**<br>Determined Elsewhere……..2<br>Both.….………...3<br>Don't know ………8 →**(Q130g)**	Quantity based on activity level…..1<br>Standard fixed supply…………..2<br>Don't know……...8	Order to maintain stock……………..1<br>Order same amount…………2<br>Order based on consumption…..3<br>Other__________.6<br>(Specify)<br>Don't know….…….8	Fall below predeter-mined level……….1<br>Fixed time – Every [__|__|__] days…….2<br>Order when needed..3<br>Other…………………6<br>Don't know………….8	One week or less…....1<br>Between 2-4 weeks….2<br>Between 5-8 weeks….3<br>More than 8 weeks…..4<br>Other ____________6<br>(Specify)<br><br>Don't know . …………8	Delivered …….1<br><br>Pick them up…2<br><br>Both …………..3
(03) Emergency contraceptives	Govt…………… A<br>Intl NGO ……… B<br>Local NGO…….C<br>Pharmacy wholesaler/ dealer/ distributor………D<br>Other________X<br>(Specify)<br>Don't know….….Z	< 4 wks ago….…..1<br>Between 4-12 wks………………2<br>> 12 wks ago……3<br>No routine supply system…………..4<br>Don't know………8	Determines own need…1→**(Q130e)**<br>Determined Elsewhere……..2<br>Both.….………...3<br>Don't know ………8 →**(Q130g)**	Quantity based on activity level…..1<br>Standard fixed supply…………..2<br>Don't know……...8	Order to maintain stock……………..1<br>Order same amount…………2<br>Order based on consumption…..3<br>Other__________.6<br>(Specify)<br>Don't know….…….8	Fall below predeter-mined level……….1<br>Fixed time – Every [__|__|__] days…….2<br>Order when needed..3<br>Other…………………6<br>Don't know………….8	One week or less…....1<br>Between 2-4 weeks….2<br>Between 5-8 weeks….3<br>More than 8 weeks…..4<br>Other ____________6<br>(Specify)<br><br>Don't know . …………8	Delivered …….1<br><br>Pick them up…2<br><br>Both …………..3
(04) Male condoms	Govt…………… A<br>Intl NGO ……… B<br>Local NGO…….C<br>Pharmacy wholesaler/ dealer/ distributor………D<br>Other________X<br>(Specify)<br>Don't know….….Z	< 4 wks ago….…..1<br>Between 4-12 wks………………2<br>> 12 wks ago……3<br>No routine supply system…………..4<br>Don't know………8	Determines own need…1→**(Q130e)**<br>Determined Elsewhere……..2<br>Both.….………...3<br>Don't know ………8 →**(Q130g)**	Quantity based on activity level…..1<br>Standard fixed supply…………..2<br>Don't know……...8	Order to maintain stock……………..1<br>Order same amount…………2<br>Order based on consumption…..3<br>Other__________.6<br>(Specify)<br>Don't know….…….8	Fall below predeter-mined level……….1<br>Fixed time – Every [__|__|__] days…….2<br>Order when needed..3<br>Other…………………6<br>Don't know………….8	One week or less…....1<br>Between 2-4 weeks….2<br>Between 5-8 weeks….3<br>More than 8 weeks…..4<br>Other ____________6<br>(Specify)<br><br>Don't know . …………8	Delivered …….1<br><br>Pick them up…2<br><br>Both …………..3

CONTRACEPTIVE	Q130a. Where does your stock of CONTRACEPTIVE come from? CHOOSE ALL.	Q130b. When was the last time that you received a routine supply of CONTRACEPTIVE either that you ordered, or that is part of your routine supply system? READ LIST.	Q130c. Does this facility determine the quantity of each CONTRACEPTIVE that it needs and order that, or is the quantity that you receive determined elsewhere? READ LIST.	Q130d. Do you always receive a standard fixed quantity of CONTRACEPTIVE or does the quantity you receive vary according to recent need or activity level? READ LIST.	Q130e. **CHECK Q130C. IFQ130C IS “2”, SKIP TO Q130G** When you order CONTRACEPTIVE, how much do you order?<br><br>READ LIST.	Q130f. When do you decide to order CONTRACEPTIVE?<br><br>READ LIST.	Q130g. On average, how long does it take to receive your supplies after you have placed an order?<br><br>READ LIST.	Q130h. Is METHOD usually delivered or must you go get them?
(05) Female condoms	Govt…………… A<br>Intl NGO ……… B<br>Local NGO…….C<br>Pharmacy wholesaler/ dealer/ distributor………D<br>Other________X<br>(Specify)<br>Don’t know….….Z	< 4 wks ago…......1<br>Between 4-12 wks……………….2<br>> 12 wks ago……3<br>No routine supply system…………..4<br>Don’t know………8	Determines own need…1→**(Q130e)**<br>Determined Elsewhere……..2<br>Both..….……….....3<br>Don’t know<br>………8 →**(Q130g)**	Quantity based on activity level…..1<br>Standard fixed supply…………..2<br>Don’t know……...8	Order to maintain stock……………..1<br>Order same amount…………2<br>Order based on consumption…..3<br>Other__________.6<br>(Specify)<br>Don’t know…..…...8	Fall below predeter-mined level……….1<br>Fixed time – Every [__|__|__] days…….2<br>Order when needed..3<br>Other…………………6<br>Don’t know………….8	One week or less…....1<br>Between 2-4 weeks….2<br>Between 5-8 weeks….3<br>More than 8 weeks…..4<br>Other ____________6<br>(Specify)<br><br>Don’t know . ………….8	Delivered …….1<br><br>Pick them up…2<br><br>Both …………..3
(06) Injectables (e.g., Depo Provera, Noristerat)	Govt…………… A<br>Intl NGO ……… B<br>Local NGO…….C<br>Pharmacy wholesaler/ dealer/ distributor………D<br>Other________X<br>(Specify)<br>Don’t know….….Z	< 4 wks ago…......1<br>Between 4-12 wks……………….2<br>> 12 wks ago……3<br>No routine supply system…………..4<br>Don’t know………8	Determines own need…1→**(Q130e)**<br>Determined Elsewhere……..2<br>Both..….……….....3<br>Don’t know<br>………8 →**(Q130g)**	Quantity based on activity level…..1<br>Standard fixed supply…………..2<br>Don’t know……...8	Order to maintain stock……………..1<br>Order same amount…………2<br>Order based on consumption…..3<br>Other__________.6<br>(Specify)<br>Don’t know…..…...8	Fall below predeter-mined level……….1<br>Fixed time – Every [__|__|__] days…….2<br>Order when needed..3<br>Other…………………6<br>Don’t know………….8	One week or less…....1<br>Between 2-4 weeks….2<br>Between 5-8 weeks….3<br>More than 8 weeks…..4<br>Other ____________6<br>(Specify)<br><br>Don’t know . ………….8	Delivered …….1<br><br>Pick them up…2<br><br>Both …………..3
(07) Implant (Norplant)	Govt…………… A<br>Intl NGO ……… B<br>Local NGO…….C<br>Pharmacy wholesaler/ dealer/ distributor………D<br>Other________X<br>(Specify)<br>Don’t know….….Z	< 4 wks ago…......1<br>Between 4-12 wks……………….2<br>> 12 wks ago……3<br>No routine supply system…………..4<br>Don’t know………8	Determines own need…1→**(Q130e)**<br>Determined Elsewhere……..2<br>Both..….……….....3<br>Don’t know<br>………8 →**(Q130g)**	Quantity based on activity level…..1<br>Standard fixed supply…………..2<br>Don’t know……...8	Order to maintain stock……………..1<br>Order same amount…………2<br>Order based on consumption…..3<br>Other__________.6<br>(Specify)<br>Don’t know…..…...8	Fall below predeter-mined level……….1<br>Fixed time – Every [__|__|__] days…….2<br>Order when needed..3<br>Other…………………6<br>Don’t know………….8	One week or less…....1<br>Between 2-4 weeks….2<br>Between 5-8 weeks….3<br>More than 8 weeks…..4<br>Other ____________6<br>(Specify)<br><br>Don’t know . ………….8	Delivered …….1<br><br>Pick them up…2<br><br>Both …………..3
(08) IUCD	Govt…………… A<br>Intl NGO ……… B<br>Local NGO…….C<br>Pharmacy wholesaler/ dealer/ distributor………D<br>Other________X<br>(Specify)<br>Don’t know….….Z	< 4 wks ago…......1<br>Between 4-12 wks……………….2<br>> 12 wks ago……3<br>No routine supply system…………..4<br>Don’t know………8	Determines own need…1→**(Q130e)**<br>Determined Elsewhere……..2<br>Both..….……….....3<br>Don’t know<br>………8 →**(Q130g)**	Quantity based on activity level…..1<br>Standard fixed supply…………..2<br>Don’t know……...8	Order to maintain stock……………..1<br>Order same amount…………2<br>Order based on consumption…..3<br>Other__________.6<br>(Specify)<br>Don’t know…..…...8	Fall below predeter-mined level……….1<br>Fixed time – Every [__|__|__] days…….2<br>Order when needed..3<br>Other…………………6<br>Don’t know………….8	One week or less…....1<br>Between 2-4 weeks….2<br>Between 5-8 weeks….3<br>More than 8 weeks…..4<br>Other ____________6<br>(Specify)<br><br>Don’t know . ………….8	Delivered …….1<br><br>Pick them up…2<br><br>Both …………..3

CONTRACEPTIVE	Q130a. Where does your stock of CONTRACEPTIVE come from? CHOOSE ALL.	Q130b. When was the last time that you received a routine supply of CONTRACEPTIVE either that you ordered, or that is part of your routine supply system? READ LIST.	Q130c. Does this facility determine the quantity of each CONTRACEPTIVE that it needs and order that, or is the quantity that you receive determined elsewhere? READ LIST.	Q130d. Do you always receive a standard fixed quantity of CONTRACEPTIVE or does the quantity you receive vary according to recent need or activity level? READ LIST.	Q130e. **CHECK Q130C. IFQ130C IS "2", SKIP TO Q130G** When you order CONTRACEPTIVE, how much do you order?<br><br>READ LIST.	Q130f. When do you decide to order CONTRACEPTIVE?<br><br>READ LIST.	Q130g. On average, how long does it take to receive your supplies after you have placed an order?<br><br>READ LIST.	Q130h. Is METHOD usually delivered or must you go get them?
(09) SDM/Cycle beads	Govt…………… A<br>Intl NGO ……… B<br>Local NGO…….C<br>Pharmacy wholesaler/ dealer/ distributor………D<br>Other________X<br>(Specify)<br>Don't know….….Z	< 4 wks ago…......1<br>Between 4-12 wks………………2<br>> 12 wks ago……3<br>No routine supply system…………..4<br>Don't know………8	Determines own need…1→**(Q130e)**<br>Determined Elsewhere……..2<br>Both..…..……....3<br>Don't know ….…..8 →**(Q130g)**	Quantity based on activity level…..1<br>Standard fixed supply…………..2<br>Don't know……...8	Order to maintain stock……………..1<br>Order same amount…………2<br>Order based on consumption…..3<br>Other__________.6<br>(Specify)<br>Don't know…..…...8	Fall below predeter-mined level……….1<br>Fixed time – Every [__|__|__] days….…2<br>Order when needed..3<br>Other…………………6<br>Don't know…………..8	One week or less…....1<br>Between 2-4 weeks….2<br>Between 5-8 weeks….3<br>More than 8 weeks…..4<br>Other ____________6<br>(Specify)<br><br>Don't know . …………8	Delivered …….1<br><br>Pick them up…2<br><br>Both …………..3
(10) Other (specify)<br><br>____________	Govt…………… A<br>Intl NGO ……… B<br>Local NGO…….C<br>Pharmacy wholesaler/ dealer/ distributor………D<br>Other________X<br>(Specify)<br>Don't know….….Z	< 4 wks ago…......1<br>Between 4-12 wks………………2<br>> 12 wks ago……3<br>No routine supply system…………..4<br>Don't know………8	Determines own need…1→**(Q130e)**<br>Determined Elsewhere……..2<br>Both..…..……....3<br>Don't know ….…..8 →**(Q130g)**	Quantity based on activity level…..1<br>Standard fixed supply…………..2<br>Don't know……...8	Order to maintain stock……………..1<br>Order same amount…………2<br>Order based on consumption…..3<br>Other__________.6<br>(Specify)<br>Don't know…..…...8	Fall below predeter-mined level……….1<br>Fixed time – Every [__|__|__] days….…2<br>Order when needed..3<br>Other…………………6<br>Don't know…………..8	One week or less…....1<br>Between 2-4 weeks….2<br>Between 5-8 weeks….3<br>More than 8 weeks…..4<br>Other ____________6<br>(Specify)<br><br>Don't know . …………8	Delivered …….1<br><br>Pick them up…2<br><br>Both …………..3

Now I would like to ask you some more questions specifically about stock-outs of family planning methods.
ONLY ASK ABOUT THOSE METHODS THAT ARE AVAILABLE FROM Q36a.

METHOD	Q131a. Is _________ METHOD currently available?	Q131b. Has this facility had a stockout of METHOD that lasted at least 24 hours in the last **one year**?	Q131c. If Yes, how many times has this facility had a stockout of METHOD in the past one year? (CHECK if **"Yes"** to Q131b)	Q131d. If Yes, how many **total** days of stockout of METHOD did this facility experience in the past one year? (CHECK if **"Yes"** to Q131b)	Q131e. SOURCE OF INFORMATION FOR STOCKOUTS IN PAST ONE YEAR:	Q131f. Has this facility had a stockout of METHOD that lasted at least 24 hours in the last 30 days (one month)?	Q131g. If Yes, how many times has this facility had a stockout of METHOD in the past 30 days? (CHECK if **"Yes"** to Q131f)	Q131h. If Yes, how many **total** days of stockout of METHOD did this facility experience in the last 30 days? (CHECK if **"Yes"** to Q131f)	Q131i. SOURCE OF INFORMATION ON STOCKOUTS IN PAST 30 DAYS:
(01) Combined oral pill	YES …..1<br>NO ……2	YES …..1<br>NO …….2 → **(02)**	Number… [ ][ ]<br>DON'T KNOW..98	Days… [ ][ ][ ]<br>CONSTANT PROBLEM…995<br>DK………….998	OBSERVED RECORD……….1<br>PROVIDER ESTIMATE..……2	YES …..1<br>NO …….2 → **(02)**	Number… [ ][ ]<br>DON'T KNOW…..98	Days… [ ][ ]<br>DON'T KNOW..98	OBSERVED RECORD………….1<br>PROVIDER ESTIMATE..………2
(02) Progestin only pill	YES …..1<br>NO ……2	YES …..1<br>NO …….2 → **(03)**	Number… [ ][ ]<br>DON'T KNOW..98	Days… [ ][ ][ ]<br>CONSTANT PROBLEM…995<br>DK………….998	OBSERVED RECORD……….1<br>PROVIDER ESTIMATE..……2	YES …..1<br>NO …….2 → **(03)**	Number… [ ][ ]<br>DON'T KNOW…..98	Days… [ ][ ]<br>DON'T KNOW..98	OBSERVED RECORD………….1<br>PROVIDER ESTIMATE..………2
(03) Emergency contraceptive	YES …..1<br>NO ……2	YES …..1<br>NO …….2 → **(04)**	Number… [ ][ ]<br>DON'T KNOW..98	Days… [ ][ ][ ]<br>CONSTANT PROBLEM…995<br>DK………….998	OBSERVED RECORD……….1<br>PROVIDER ESTIMATE..……2	YES …..1<br>NO …….2 → **(04)**	Number… [ ][ ]<br>DON'T KNOW…..98	Days… [ ][ ]<br>DON'T KNOW..98	OBSERVED RECORD………….1<br>PROVIDER ESTIMATE..………2
(04) Male condom	YES …..1<br>NO ……2	YES …..1<br>NO …….2 → **(05)**	Number… [ ][ ]<br>DON'T KNOW..98	Days… [ ][ ][ ]<br>CONSTANT PROBLEM…995<br>DK………….998	OBSERVED RECORD……….1<br>PROVIDER ESTIMATE..……2	YES …..1<br>NO …….2 → **(05)**	Number… [ ][ ]<br>DON'T KNOW…..98	Days… [ ][ ]<br>DON'T KNOW..98	OBSERVED RECORD………….1<br>PROVIDER ESTIMATE..………2

METHOD	Q131a. Is ________ METHOD currently available?	Q131b. Has this facility had a stockout of METHOD that lasted at least 24 hours in the last **one year**?	Q131c. If Yes, how many times has this facility had a stockout of METHOD in the past one year?<br><br>(CHECK if **"Yes"** to Q131b)	Q131d. If Yes, how many **total** days of stockout of METHOD did this facility experience in the past one year?<br><br>(CHECK if **"Yes"** to Q131b)	Q131e. SOURCE OF INFORMATION FOR STOCKOUTS IN PAST ONE YEAR:	Q131f. Has this facility had a stockout of METHOD that lasted at least 24 hours in the last 30 days (one month)?	Q131g. If Yes, how many times has this facility had a stockout of METHOD in the past 30 days?<br><br>(CHECK if **"Yes"** to Q131f)	Q131h. If Yes, how many **total** days of stockout of METHOD did this facility experience in the last 30 days?<br><br>(CHECK if **"Yes"** to Q131f)	Q131i. SOURCE OF INFORMATION ON STOCKOUTS IN PAST 30 DAYS:
(05) Female condom	YES .....1<br>NO 2	YES .....1<br>NO ....2<br>↓ **(06)**	Number… [ ][ ]<br>DON'T KNOW..98	Days… [ ][ ][ ]<br>CONSTANT PROBLEM…995<br>DK…………998	OBSERVED RECORD……….1<br>PROVIDER ESTIMATE…….2	YES .....1<br>NO ....2<br>↓ **(06)**	Number… [ ][ ]<br>DON'T KNOW…..98	Days… [ ][ ]<br>DON'T KNOW..98	OBSERVED RECORD………….1<br>PROVIDER ESTIMATE……….2
(06) Injectable (DMPA, Noristerat)	YES .....1<br>NO 2	YES .....1<br>NO ....2<br>↓ **(07)**	Number… [ ][ ]<br>DON'T KNOW..98	Days… [ ][ ][ ]<br>CONSTANT PROBLEM…995<br>DK…………998	OBSERVED RECORD………….1<br>PROVIDER ESTIMATE……….2	YES .....1<br>NO ....2<br>↓ **(07)**	Number… [ ][ ]<br>DON'T KNOW…..98	Days… [ ][ ]<br>DON'T KNOW..98	OBSERVED RECORD………….1<br>PROVIDER ESTIMATE……….2
(07) Implants (Jadelle/ Implanon)	YES .....1<br>NO 2	YES .....1<br>NO ....2<br>↓ **(08)**	Number… [ ][ ]<br>DON'T KNOW..98	Days… [ ][ ][ ]<br>CONSTANT PROBLEM…995<br>DK…………998	OBSERVED RECORD………….1<br>PROVIDER ESTIMATE……….2	YES .....1<br>NO ....2<br>↓ **(08)**	Number… [ ][ ]<br>DON'T KNOW…..98	Days… [ ][ ]<br>DON'T KNOW..98	OBSERVED RECORD………….1<br>PROVIDER ESTIMATE……….2
(08) IUCD	YES .....1<br>NO 2	YES .....1<br>NO ....2<br>↓ **(09)**	Number… [ ][ ]<br>DON'T KNOW..98	Days… [ ][ ][ ]<br>CONSTANT PROBLEM…995<br>DK…………998	OBSERVED RECORD………….1<br>PROVIDER ESTIMATE……….2	YES .....1<br>NO ....2<br>↓ **(09)**	Number… [ ][ ]<br>DON'T KNOW…..98	Days… [ ][ ]<br>DON'T KNOW..98	OBSERVED RECORD………….1<br>PROVIDER ESTIMATE……….2

(09) SDM/Cycle beads	YES …..1<br>NO ……2	YES …..1<br>NO …….2<br>(10)	Number…<br>DON'T KNOW..98	Days…<br>CONSTANT PROBLEM…995<br>DK…………998	OBSERVED RECORD…………1<br>PROVIDER ESTIMATE..………2	YES …..1<br>NO …….2<br>(10)	Number…<br>DON'T KNOW…..98	Days…<br>DON'T KNOW..98	OBSERVED RECORD…………1<br>PROVIDER ESTIMATE..………2
(10) Other (specify)<br>__________	YES …..1<br>NO ……2	YES …..1<br>NO …….2<br>(Q132)	Number…<br>DON'T KNOW..98	Days…<br>CONSTANT PROBLEM…995<br>DK…………998	OBSERVED RECORD…………1<br>PROVIDER ESTIMATE..………2	YES …..1<br>NO …….2<br>(Q132)	Number…<br>DON'T KNOW…..98	Days…<br>DON'T KNOW..98	OBSERVED RECORD…………1<br>PROVIDER ESTIMATE..………2

Q132.	RECORD THE TIME<br>[24-HOUR TIME] Hour …………<br>Minutes ……..
Thank you very much for taking the time to answer my questions. Once again, any information you have given will be kept confidential. Have a good day!	
COMMENTS:	
