# Supplementary material for: Facility-level characteristics associated with family planning and child immunization services integration in urban areas of Nigeria: a longitudinal analysis
Source: BMC Public Health. 2021 Jul 12;21:1379. doi: 10.1186/s12889-021-11436-x (PMC8274034; doi:10.1186/s12889-021-11436-x)
Supplement: Supplementary file 4 — Additional file 4. Endline Provider Survey. [file 12889_2021_11436_MOESM4_ESM.pdf]

QUESTIONNAIRE IDENTIFICATION NO:[__|__|__|__|__|__|__|__]
5 digit facility code + 3 digit prov code

MLE MEASUREMENT, LEARNING & EVALUATION PROJECT FOR THE URBAN REPRODUCTIVE HEALTH INITIATIVE

# Measurement, Learning & Evaluation (MLE) Project
# Service Provider – Nigeria - 2014

**IDENTIFICATION**

| | |
|---|---|
| CITY NAME & CODE ____________________<br>(Abuja=1, Benin=2, Ibadan=3, Ilorin=4, Kaduna=5, Zaria=6) | [__] |
| LGA NAME & CODE____________________ | [__|__|__] |
| LOCALITY NAME & CODE ____________________ | [__|__|__|__] |
| FACILITY NAME AND CODE ____________________ | [__|__|__|__|__] |
| PROVIDER NAME AND CODE (FROM THE FACILITY AUDIT LIST – Q10d)____________________ | [__|__|__] |

RESPONDENT: NOT INTERVIEWED = 1 PREVIOUSLY INTERVIEWED AT ANOTHER FACILITY = 2 (END) [ ]

**IF PREVIOUSLY INTERVIEWED**, OTHER FACILITY NAME AND CODE____________________ [ | | | | ]

**INTERVIEWER VISITS**

| **VISIT No.** | **1** | **2** | **3** | **FINAL VISIT** |
|---|---|---|---|---|
| DATE | DAY/ MONTH/YEAR<br>[____/_____/_**14**_] | DAY/ MONTH/ YEAR<br>[____/_____/__**14**_] | DAY/ MONTH/ YEAR<br>[____/_____/__**14**_] | DAY [____|____]<br>MONTH [____|____]<br>YEAR<br>[_**2**_|_**0**_|_**1**_|_**4**_] |
| INTERVIEWER'S NAME | ______________ | ______________ | ______________ | ______________ |
| INTERVIEWER CODE | [ | | ] | [ | | ] | [ | | ] | [ | | ] |
| RESULT* | [ ] | [ ] | [ ] | [ ] |
| **NEXT VISIT:**<br>**DATE:** | [___/_____/**14**_] | [___/_____/**14**_] | [___/_____/**14**_] | TOTAL NO. OF VISITS |
| **TIME:** | [ | ] [ | ]<br>H H M M | [ | ] [ | ]<br>H H M M | [ | ] [ | ]<br>H H M M | [ ] |

***RESULT CODES:**

1. COMPLETED
2. RESPONDENT NOT AVAILABLE
3. POSTPONED
4. REFUSED
5. PARTLY COMPLETED
6. OTHER ____________________
(Specify)

QUESTIONNAIRE IDENTIFICATION NO:[__|__|__|__|__|__|__|__]
5 digit facility code + 3 digit prov code

| SUPERVISOR | OFFICE EDITOR | KEYED BY |
|---|---|---|
| NAME ________________ | NAME ________________ | NAME ________________ |
| CODE [____\|____] | CODE [____\|____] | CODE [____\|____] |
| DATE [____/_____/_**14**_] DD MM YY | DATE [____/_____/_**14**_] DD MM YY | DATE [____/_____/_**14**_] DD MM YY |

| **BACKGROUND INFORMATION** | | | |
|---|---|---|---|
| **Source** | **Questions** | **Coding** | **Skip** |
| Q1. | RECORD THE TIME<br><br>(IN 24 HOUR FORMAT) | Hour ………… [ \| ] Minutes …….. [ \| ] | |
| Q2. | SEX OF PROVIDER INTERVIEWED | MALE……………………………….…1<br>FEMALE……………………….……..2 | |
| Q3. | How long have you been working here at this facility? | YEARS… [ \| ]<br><br>LESS THAN ONE YEAR =00<br>DON'T KNOW = 98 | |
| Q4. | What cadre of staff are you? | OBSTETRICIAN/GYNECOLOGIST…………..…………..01<br>GENERAL SURGEON…………………………….…..……02<br>PEDIATRICIAN…………………………………………..03<br>GENERAL PHYSICIAN……………………………………..04<br>THEATRE NURSE………………………………..……05<br>NURSE/MIDWIFE…………………………………..…06<br>NURSE……………………………………………….......07<br>MIDWIFE……………………………..……………….……08<br>COMMUNITY HEALTH EXTENSION WORKER (CHEW)…09<br>COMMUNITY HEALTH OFFICER (CHO)……………………10<br>VCT COUNSELOR………………………………………...11<br>OTHER ________________________96<br>(SPECIFY) | |
| Q5. | How old were you at your last birthday? | YEARS…….. [ \| ] | |
| Q6. | What is your religion? | CHRISTIAN-CATHOLIC………………………………………1<br>CHRISTIAN-PROTESTANT/OTHER CHRISTIAN…………2<br>ISLAM…………………………..…………………………3<br>TRADITIONAL……………………………………………….4<br>NO RELIGION ……………………………………...…5<br><br>OTHER________________________6<br>(SPECIFY) | |
| Q7. | In which department or unit do you work? | GENERAL OUTPATIENT DEPARTMENT (GOPD) ... 01<br>OBSTETRICS AND GYNECOLOGY …..……………. .02<br>SURGERY ……..……………………………..………..03<br>PEDIATRICS ……….………………………………….04<br>FAMILY PLANNING DEPARTMENT …..………………05<br>INFANT AND CHILD CARE ………………………….….06<br>ANC………………………………..……………….…07<br>HIV TESTING OR STI/HIV TREATMENT……………..08<br>OTHER ________________________96<br>(SPECIFY) | |
| Q8. | How many years have you been working as a health care provider? | NUMBER OF YEARS: [ \| ] | |
| Q9. | How many years ago did you finish your **<u>pre-service</u>** training? | YEARS AGO….. [ \| ]<br><br>LESS THAN ONE YEAR = 00<br>NO PRE-SERVICE TRAINING=97 | |

| | | | |
|---|---|---|---|
| Q10. | Have you received any **in-service** training on family planning? | YES……………………………….…1<br>NO…………………………………..2 | <br>→ **Q14** |
| Q11. | Who provided this training?<br><br>**MULTIPLE RESPONSES POSSIBLE.**<br><br>**CIRCLE ALL MENTIONED.** | NURHI/FPPN ………………………………. ..A<br>UNFPA …………………………………………B<br>WHO ……………………………………………C<br>SFH ……………………………………………..D<br>JPIEGO …………………………………………E<br>MARIE STOPES ………………………………F<br>PATH ……………………………………………G<br>PPFN ……………………………………………H<br>STATE MOH ……………………………………I<br>TSHIP ……………………………………………J<br>OTHER ___________________________ X<br>(SPECIFY) | |
| Q12. | What types of in-service trainings did you receive?<br><br>PROBE – Anything else?<br><br>**MULTIPLE RESPONSES POSSIBLE.**<br><br>**CIRCLE ALL MENTIONED.** | FP INTERPERSONAL COMMUNICATION AND COUNSELLING……………………… ………..A<br>INITIAL FP TRAININGS ……………………………… .. B<br>REFRESHER FP TRAINING ……………………………C<br>LONG-ACTING AND PERMANENT FP METHODS... .D<br>TRAINING OF TRAINERS OF FP PROVIDERS ……. E<br>CONTRACEPTIVE LOGISTICS AND MANAGEMENT SYSTEM (CLMS) TRAINING …………F<br>SUPERVISORY SKILLS TRAINING…………………… G<br><br>OTHER __________________________________ X<br>(SPECIFY) | |
| Q13. | How long ago was the last **in-service** family planning training that you attended? | DAYS AGO…………………1<br>WEEKS AGO………………..2<br>MONTHS AGO…...............3<br>YEARS AGO….....................4<br>DON'T REMEMBER………998<br>[__|__] | |
| Q14. | Are you a member of the NURHI supported Family Planning Provider Network (FPPN)? | YES………………………………………………………1<br>NO………………………………………………………2 | <br>→ **Q22** |
| Q15. | Have you attended any of the FPPN meetings? | YES…………………………………………………...1<br>NO……………………………………………………2<br>DON'T KNOW…………………………………………8 | |
| Q16. | Do you discuss family planning <u>with other FPPN members</u>? | YES…………………………………………………1<br>NO……………………………………………….. .2<br>DON'T KNOW ANY OTHER MEMBERS……..……..8 | <br>→ **Q19**<br>→ **Q19** |
| Q17. | What cadre of <u>providers in the FPPN</u> do you discuss family planning related issues with?<br><br>**MULTIPLE RESPONSES POSSIBLE.**<br><br>**CIRCLE ALL MENTIONED.** | DOCTORS ……………………………………………. A<br>NURSE/MIDWIFE………………………………………B<br>PHARMACISTS ………………………………………C<br>PMV ………………………………………………….D<br>CHEW ………………………………………………E<br>OTHER _________________________________X<br>(SPECIFY) | |
| Q18. | When you discuss family planning related topics <u>with other providers in the FPPN</u>, what do you talk about?<br><br>**MULTIPLE RESPONSES POSSIBLE.**<br><br>**CIRCLE ALL MENTIONED.** | CONTENT OF THE TRAININGS ……………………. A<br>INTERACTIONS WITH NURHI STAFF……… ……....B<br>REFERRAL …………………………………………C<br>RECORD KEEPING ……………………………………D<br>FP COMMODITY AVAILABILITY …………………….. E<br>SUPPORTIVE SUPERVISION…………………………F<br>CONSUMABLES ………………………………………G<br>FAMILY PLANNING PROVIDER ATTITUDES… ……H<br>FAMILY PLANNING SERVICE PROVISION…………I<br>OTHER ________________________ __________X<br>(SPECIFY) | |

| | | | |
|---|---|---|---|
| Q19. | Do you discuss family planning with other providers who are NOT FPPN members? | YES……………………………………………………1<br>NO……………………………………………………2<br>DON'T KNOW NON-FPPN PROVIDERS ………..8 | <br>→ **Q28**<br>→ **Q28** |
| Q20. | What cadre of providers outside the FPPN do you discuss family planning related issues with?<br><br>**MULTIPLE RESPONSES POSSIBLE.**<br><br>**CIRCLE ALL MENTIONED.** | DOCTORS ………………………………………… A<br>NURSE/MIDWIFE………………………………B<br>PHARMACISTS ……………………………………C<br>PMV …………………………………………………D<br>CHEW …………………………………………………E<br>OTHER ______________________________ X<br>(SPECIFY) | |
| Q21. | When you discuss family planning related topics with other providers who are not in the FPPN, what do you talk about?<br><br>**MULTIPLE RESPONSES POSSIBLE.**<br><br>**CIRCLE ALL MENTIONED.** | CONTENT OF THE TRAININGS ………………… A<br>INTERACTIONS WITH NURHI STAFF……… …..B<br>REFERRAL ……………………………………………C<br>RECORD KEEPING …………………………………D<br>FP COMMODITY AVAILABILITY …………………. E<br>SUPPORTIVE SUPERVISION………………………F<br>CONSUMABLES ……………………………………..G<br>FAMILY PLANNING PROVIDER ATTITUDES… …H<br>FAMILY PLANNING SERVICE PROVISION……….I<br>OTHER ________________________ ________X<br>(SPECIFY) | **All skip to Q28** |
| Q22. | Have you interacted with providers who are members of the FPPN? | YES……………………………………………………1<br>NO……………………………………………………...2<br>DON'T KNOW/DON'T KNOW FPPN MEMBERS ….8 | <br>→ **Q25**<br>→ **Q25** |
| Q23. | What cadre of providers in the FPPN do you discuss family planning related issues with?<br><br>**MULTIPLE RESPONSES POSSIBLE.**<br><br>**CIRCLE ALL MENTIONED.** | DOCTORS ………………………………………… A<br>NURSE/MIDWIFE………………………………..B<br>PHARMACISTS ……………………………………C<br>PMV …………………………………………………D<br>CHEW ………………………………………………...E<br>OTHER ________________________________ X<br>(SPECIFY)<br><br>DO NOT DISCUSS FP…………………………………Y | <br><br><br><br><br><br><br><br>→ **Q25** |
| Q24. | What types of family planning topics have you discussed with FPPN network members?<br><br>**MULTIPLE RESPONSES POSSIBLE.**<br><br>**CIRCLE ALL MENTIONED** | CONTENT OF THE TRAININGS ………………… A<br>INTERACTIONS WITH NURHI STAFF……… …..B<br>REFERRAL ……………………………………………C<br>RECORD KEEPING …………………………………D<br>FP COMMODITY AVAILABILITY …………………. E<br>SUPPORTIVE SUPERVISION………………………F<br>CONSUMABLES ……………………………………G<br>FAMILY PLANNING PROVIDER ATTITUDES…...H<br>FAMILY PLANNING SERVICE PROVISION……...I<br>OTHER ________________________ ________X<br>(SPECIFY) | |
| Q25. | Have you interacted with providers who are not members of the FPPN? | YES……………………………………………………1<br>NO…………………………………………………… 2<br>DON'T KNOW…………………………………………8 | <br>→ **Q28**<br>→ **Q28** |
| Q26. | What cadre of providers not in the FPPN do you discuss family planning related issues with?<br><br>**MULTIPLE RESPONSES POSSIBLE.**<br><br>**CIRCLE ALL MENTIONED.** | DOCTORS ……………………………… …………..A<br>NURSE/MIDWIFE…………………………………B<br>PHARMACISTS …………………………………C<br>PMV ………………………………………………D<br>CHEW ………………………………………………...E<br>OTHER _______________________________ X<br>(SPECIFY)<br><br>DO NOT DISCUSS FP…………………………………Y | <br><br><br><br><br><br><br><br>→ **Q28** |

| | | | |
|---|---|---|---|
| Q27. | What types of family planning topics have you discussed with providers who are not members of the FPPN?<br><br>**MULTIPLE RESPONSES POSSIBLE.**<br><br>**CIRCLE ALL MENTIONED** | CONTENT OF THE TRAININGS ………………… A<br>INTERACTIONS WITH NURHI STAFF……… …..B<br>REFERRAL …………………………………………..C<br>RECORD KEEPING ……………………………….D<br>FP COMMODITY AVAILABILITY …………………. E<br>SUPPORTIVE SUPERVISION………………………F<br>CONSUMABLES ……………………………………..G<br>FAMILY PLANNING PROVIDER ATTITUDES… …H<br>FAMILY PLANNING SERVICE PROVISION……….I<br>OTHER _____________________ _________X<br>(SPECIFY) | |

**TRAINING ON FAMILY PLANNING**

Now, I will ask you few questions related to training on FP.

**Q28. CHECK Q09 AND Q10 ON PRE-SERVICE AND IN-SERVICE TRAINING:**

**HAS HAD BOTH PRE AND IN-SERVICE TRAINING** ☐
(Q9=00 OR HIGHER AND Q10=1)
**THEN ANSWER Q28a-Q28d**

**HAS HAD IN-SERVICE TRAINING ONLY** ☐ → **Q28b**
(Q9=97 AND Q10=1)

**HAS HAD PRE-SERVICE TRAINING ONLY** ☐
(Q9=00 OR GREATER AND Q10=2)
**THEN ANSWER 28a ONLY**

**HAS NOT HAD ANY PRE OR IN SERVICE TRAINING** ☐ → **Q29a**
(Q9=97 AND Q10=2)

| TOPICS | | Q28a. Did your **pre-service** training cover TOPIC? | Q28b. Have you ever attended an **in-service** training on TOPIC? | Q28c. What year was your most recent **in-service** training on TOPIC? | Q28d. Which organization or government ministry conducted this training? |
|---|---|---|---|---|---|
| *(01)* | Contraceptive technology update | YES . . . . . . . .1<br>NO . . . . . . . .. 2<br>DK…………..8 | YES . . . . . . . . .1<br>NO . . . . . . . . . .2 →**(02)** | [___\|___\|___\|___]<br>**DK=9998** | ____________<br>\|___\|___\| |
| *(02)* | Exclusive breastfeeding counseling/LAM | YES . . . . . . . .1<br>NO . . . . . . . .. 2<br>DK…………..8 | YES . . . . . . . . .1<br>NO . . . . . . . . . .2 →**(03)** | [___\|___\|___\|___]<br>**DK=9998** | ____________<br>\|___\|___\| |
| *(03)* | Natural family planning (rhythm method, etc.) | YES . . . . . . . .1<br>NO . . . . . . . .. 2<br>DK…………..8 | YES . . . . . . . . .1<br>NO . . . . . . . . . .2 →**(04)** | [___\|___\|___\|___]<br>**DK=9998** | ____________<br>\|___\|___\| |
| *(04)* | SDM/Cycle beads | YES . . . . . . ..1<br>NO . . . . . . . ..2<br>DK…………..8 | YES . . . ….. .1<br>NO . . . . ……2 →**(05)** | [___\|___\|___\|___]<br>**DK=9998** | ____________<br>\|___\|___\| |
| *(05)* | Emergency Contraceptive | YES . . . . . . . .1<br>NO . . . . . . . .. 2<br>DK…………..8 | YES . . . . . . . . .1<br>NO . . . . . . . . . .2 →**(06)** | [___\|___\|___\|___]<br>**DK=9998** | ____________<br>\|___\|___\| |
| *(06)* | Oral pills | YES . . . . . . . .1<br>NO . . . . . . . .. 2<br>DK…………..8 | YES . . . . . . . . .1<br>NO . . . . . . . . . .2 →**(07)** | [___\|___\|___\|___]<br>**DK=9998** | ____________<br>\|___\|___\| |
| *(07)* | FP counseling skills | YES . . . . . . . .1<br>NO . . . . . . . .. 2<br>DK…………..8 | YES . . . . . . . . .1<br>NO . . . . . . . . . .2 →**(08)** | [___\|___\|___\|___]<br>**DK=9998** | ____________<br>\|___\|___\| |

| TOPICS | | Q28a. Did your **pre-service** training cover TOPIC? | Q28b. Have you ever attended an **in-service** training on TOPIC? | Q28c. What year was your most recent **in-service** training on TOPIC? | Q28d. Which organization or government ministry conducted this training? |
|---|---|---|---|---|---|
| *(08)* | Clinical skills on IUD | YES . . . . . . . .1<br>NO . . . . . . . .. 2<br>DK…………..8 | YES . . . . . . . . .1<br>NO . . . . . . . . . .2 →**(09)** | [___\|___\|___\|___]<br>**DK=9998** | ______________<br>\|___\|___\| |
| *(09)* | Clinical skills on injectable contraceptive | YES . . . . . . . .1<br>NO . . . . . . . .. 2<br>DK…………..8 | YES . . . . . . . . .1<br>NO . . . . . . . . . .2 →**(10)** | [___\|___\|___\|___]<br>**DK=9998** | ______________<br>\|___\|___\| |
| *(10)* | Clinical skills on implant | YES . . . . . . . .1<br>NO . . . . . . . .. 2<br>DK…………..8 | YES . . . . . . . . .1<br>NO . . . . . . . . . .2 →**(11)** | [___\|___\|___\|___]<br>**DK=9998** | ______________<br>\|___\|___\| |
| *(11)* | Clinical skills on Female Sterilization | YES . . . . . . . .1<br>NO . . . . . . . .. 2<br>DK…………..8 | YES . . . . . . . . .1<br>NO . . . . . . . . . .2 →**(12)** | [___\|___\|___\|___]<br>**DK=9998** | ______________<br>\|___\|___\| |
| *(12)* | Clinical skills on male sterilization | YES . . . . . . . .1<br>NO . . . . . . . .. 2<br>DK…………..8 | YES . . . . . . . . .1<br>NO . . . . . . . . . .2 →**(13)** | [___\|___\|___\|___]<br>**DK=9998** | ______________<br>\|___\|___\| |
| *(13)* | Management of incomplete abortion (Post-Abortion Care) | YES . . . . . . . .1<br>NO . . . . . . . .. 2<br>DK…………..8 | YES . . . . . . . . .1<br>NO . . . . . . . . . .2 →**(14)** | [___\|___\|___\|___]<br>**DK=9998** | ______________<br>\|___\|___\| |
| *(14)* | Manual vacuum aspiration (MVA) | YES . . . . . . . .1<br>NO . . . . . . . .. 2<br>DK…………..8 | YES. . . ... . .1<br>NO . . . . . . . .2→**(Q29a)** | [___\|___\|___\|___]<br>**DK=9998** | ______________<br>\|___\|___\| |

QUESTIONNAIRE IDENTIFICATION NO:[__|__|__|__|__|__|__|__]
5 digit facility code + 3 digit prov code

**Now I would like to ask you some questions about your knowledge and provision of various methods of family planning. If you have provided a particular method before, we are also interested in the availability and quality of the materials required to provide that method.**

| METHOD | 29a. Can you please tell me which of the following best describes your knowledge of [METHOD]:<br>1. You know METHOD sufficiently well to counsel and provide/assist in provision to a client;<br>2. You know METHOD sufficiently well to counsel, but not to provide;<br>3. You know little about METHOD and would not feel comfortable counseling or providing;<br>8. You do not know METHOD at all | 29b. Have you provided (assisted with) [METHOD] to clients at this facility? | 29c. Have you experienced any stockouts in this facility that lasted more than 24 hours of [METHOD] in the last one year? | 29d. If yes, how many total days of stockouts did this facility have in the last ONE YEAR of [METHOD] (all stockouts combined)? | 29e. Have you experienced a lack of essential equipment needed to provide [METHOD] in the last ONE YEAR? | 29f. If Yes, how many total days did you lack essential equipment needed to provide [METHOD] in the last ONE YEAR? |
|---|---|---|---|---|---|---|
| (01) Combined oral pill | PROVIDE & COUNSEL……………1<br>COUNSEL, NOT PROVIDE…..2<br>KNOW LITTLE ABOUT……….3<br>DO NOT KNOW………………8<br>(2, 3, 8) → **(02)** | YES……..1<br>NO………2 → **(02)** | YES………..1<br>NO…………2 → **(02)**<br>PRESCRIPTION ONLY……….3 → **(02)** | DAYS…<br>[ ][ ][ ]<br>CONSTANT PROBLEM…995<br>DON'T KNOW..998 | | |
| (02) Progestin-only pill | PROVIDE & COUNSEL……………1<br>COUNSEL, NOT PROVIDE…..2<br>KNOW LITTLE ABOUT……….3<br>DO NOT KNOW………………8<br>(2, 3, 8) → **(03)** | YES……..1<br>NO………2 → **(03)** | YES………..1<br>NO…………2 → **(03)**<br>PRESCRIPTION ONLY……….3 → **(03)** | DAYS…<br>[ ][ ][ ]<br>CONSTANT PROBLEM…995<br>DON'T KNOW..998 | | |
| (03) Injectables | PROVIDE & COUNSEL……………1<br>COUNSEL, NOT PROVIDE…..2<br>KNOW LITTLE ABOUT……….3<br>DO NOT KNOW………………8<br>(2, 3, 8) → **(04)** | YES……..1<br>NO………2 → **(04)** | YES………..1<br>NO…………2 → **(04)**<br>PRESCRIPTION ONLY……….3 → **(04)** | DAYS…<br>[ ][ ][ ]<br>CONSTANT PROBLEM…995<br>DON'T KNOW..998 | | |

**Now I would like to ask you some questions about your knowledge and provision of various methods of family planning. If you have provided a particular method before, we are also interested in the availability and quality of the materials required to provide that method.**

| METHOD | 29a. Can you please tell me which of the following best describes your knowledge of [METHOD]:<br>1. You know METHOD sufficiently well to counsel and provide/assist in provision to a client;<br>2. You know METHOD sufficiently well to counsel, but not to provide;<br>3. You know little about METHOD and would not feel comfortable counseling or providing;<br>8. You do not know METHOD at all | 29b. Have you provided (assisted with) [METHOD] to clients at this facility? | 29c. Have you experienced any stockouts in this facility that lasted more than 24 hours of [METHOD] in the last one year? | 29d. If yes, how many total days of stockouts did this facility have in the last ONE YEAR of [METHOD] (all stockouts combined)? | 29e. Have you experienced a lack of essential equipment needed to provide [METHOD] in the last ONE YEAR? | 29f. If Yes, how many total days did you lack essential equipment needed to provide [METHOD] in the last ONE YEAR? |
|---|---|---|---|---|---|---|
| (04) Male condom | PROVIDE & COUNSEL……………1<br>COUNSEL, NOT PROVIDE…..2<br>KNOW LITTLE ABOUT……….3<br>DO NOT KNOW………………8<br>(2, 3, 8) → **(05)** | YES……..1<br>NO………2 → **(05)** | YES………..1<br>NO…………2 → **(05)**<br>PRESCRIPTION ONLY……….3 → **(05)** | DAYS…<br>[ ][ ][ ]<br>CONSTANT PROBLEM…995<br>DON'T KNOW..998 | | |
| (05) Female condom | PROVIDE & COUNSEL……………1<br>COUNSEL, NOT PROVIDE…..2<br>KNOW LITTLE ABOUT……….3<br>DO NOT KNOW………………8<br>(2, 3, 8) → **(06)** | YES……..1<br>NO………2 → **(06)** | YES………..1<br>NO…………2 → **(06)**<br>PRESCRIPTION ONLY……….3 → **(06)** | DAYS…<br>[ ][ ][ ]<br>CONSTANT PROBLEM…995<br>DON'T KNOW..998 | | |
| (06) Emergency contraception | PROVIDE & COUNSEL……………1<br>COUNSEL, NOT PROVIDE…..2<br>KNOW LITTLE ABOUT……….3<br>DO NOT KNOW………………8<br>(2, 3, 8) → **(07)** | YES……..1<br>NO………2 → **(07)** | YES………..1<br>NO…………2 → **(07)**<br>PRESCRIPTION ONLY……….3 → **(07)** | DAYS…<br>[ ][ ][ ]<br>CONSTANT PROBLEM…995<br>DON'T KNOW..998 | | |

**Now I would like to ask you some questions about your knowledge and provision of various methods of family planning. If you have provided a particular method before, we are also interested in the availability and quality of the materials required to provide that method.**

| METHOD | 29a. Can you please tell me which of the following best describes your knowledge of [METHOD]:<br>1. You know METHOD sufficiently well to counsel and provide/assist in provision to a client;<br>2. You know METHOD sufficiently well to counsel, but not to provide;<br>3. You know little about METHOD and would not feel comfortable counseling or providing;<br>8. You do not know METHOD at all | 29b. Have you provided (assisted with) [METHOD] to clients at this facility? | 29c. Have you experienced any stockouts in this facility that lasted more than 24 hours of [METHOD] in the last one year? | 29d. If yes, how many total days of stockouts did this facility have in the last ONE YEAR of [METHOD] (all stockouts combined)? | 29e. Have you experienced a lack of essential equipment needed to provide [METHOD] in the last ONE YEAR? | 29f. If Yes, how many total days did you lack essential equipment needed to provide [METHOD] in the last ONE YEAR? |
|---|---|---|---|---|---|---|
| (07) Spermicide | PROVIDE & COUNSEL…………….1<br>COUNSEL, NOT PROVIDE…..2<br>KNOW LITTLE ABOUT……….3 } **(08)**<br>DO NOT KNOW……………….8 | YES……..1<br>NO………2 → **(08)** | YES………...1<br>NO…….……2 → **(08)**<br>PRESCRIPTION ONLY………..3 → **(08)** | DAYS…<br>[ ][ ][ ]<br>CONSTANT PROBLEM…995<br>DON'T KNOW..998 | | |
| (08) Diaphragm | PROVIDE & COUNSEL…………….1<br>COUNSEL, NOT PROVIDE…..2<br>KNOW LITTLE ABOUT……….3 } **(09)**<br>DO NOT KNOW……………….8 | YES……..1<br>NO………2 → **(09)** | YES………..1<br>NO…………2 → **(09)**<br>PRESCRIPTION ONLY……….3 → **(09)** | DAYS…<br>[ ][ ][ ]<br>CONSTANT PROBLEM…995<br>DON'T KNOW..998 | | |
| (09) SDM/Cycle beads | PROVIDE & COUNSEL…………….1<br>COUNSEL, NOT PROVIDE…..2<br>KNOW LITTLE ABOUT……….3 } **(10)**<br>DO NOT KNOW……………….8 | YES……..1<br>NO………2 → **(10)** | YES………..1<br>NO…….……2 → **(10)**<br>PRESCRIPTION ONLY……….3 → **(10)** | DAYS…<br>[ ][ ][ ]<br>CONSTANT PROBLEM…995<br>DON'T KNOW..998 | | |

**Now I would like to ask you some questions about your knowledge and provision of various methods of family planning. If you have provided a particular method before, we are also interested in the availability and quality of the materials required to provide that method.**

| METHOD | 29a. Can you please tell me which of the following best describes your knowledge of [METHOD]:<br>1. You know METHOD sufficiently well to counsel and provide/assist in provision to a client;<br>2. You know METHOD sufficiently well to counsel, but not to provide;<br>3. You know little about METHOD and would not feel comfortable counseling or providing;<br>8. You do not know METHOD at all | 29b. Have you provided (assisted with) [METHOD] to clients at this facility? | 29c. Have you experienced any stockouts in this facility that lasted more than 24 hours of [METHOD] in the last one year? | 29d. If yes, how many total days of stockouts did this facility have in the last ONE YEAR of [METHOD] (all stockouts combined)? | 29e. Have you experienced a lack of essential equipment needed to provide [METHOD] in the last ONE YEAR? | 29f. If Yes, how many total days did you lack essential equipment needed to provide [METHOD] in the last ONE YEAR? |
|---|---|---|---|---|---|---|
| (10) IUD | PROVIDE & COUNSEL…………….1<br>COUNSEL, NOT PROVIDE…..2<br>KNOW LITTLE ABOUT……….3<br>DO NOT KNOW……………….8<br>(2, 3, 8) → **(11)** | YES……..1<br>NO………2→ **(11)** | YES………...1<br>NO….……..2 → **(29e)**<br>PRESCRIPTION ONLY………..3 →**(11)** | DAYS… [ ][ ][ ]<br>CONSTANT PROBLEM…995<br>DON'T KNOW..998 | YES……..1<br>NO………2→ **(11)** | DAYS… [ ][ ][ ]<br>CONSTANT PROBLEM…995<br>DK…...998 |
| (11) Implants | PROVIDE & COUNSEL…………….1<br>COUNSEL, NOT PROVIDE…..2<br>KNOW LITTLE ABOUT……….3<br>DO NOT KNOW……………….8<br>(2, 3, 8) → **(12)** | YES……..1<br>NO………2→ **(12)** | YES………...1<br>NO….……..2 → **(29e)**<br>PRESCRIPTION ONLY………..3 →**(12)** | DAYS… [ ][ ][ ]<br>CONSTANT PROBLEM…995<br>DON'T KNOW..998 | YES……..1<br>NO………2→ **(12)** | DAYS… [ ][ ][ ]<br>CONSTANT PROBLEM…995<br>DK…...998 |
| (12) Female sterilization | PROVIDE & COUNSEL…………….1<br>COUNSEL, NOT PROVIDE…..2<br>KNOW LITTLE ABOUT……….3<br>DO NOT KNOW……………….8<br>(2, 3, 8) → **(13)** | YES……..1<br>NO………2→ **(13)** | | | YES……..1<br>NO………2→ **(13)** | DAYS… [ ][ ][ ]<br>CONSTANT PROBLEM…995<br>DK…...998 |
| (13) Male sterilization | PROVIDE & COUNSEL…………….1<br>COUNSEL, NOT PROVIDE…..2<br>KNOW LITTLE ABOUT……….3<br>DO NOT KNOW……………….8<br>(2, 3, 8) → **(14)** | YES……..1<br>NO………2→ **(14)** | | | YES……..1<br>NO………2→ **(14)** | DAYS… [ ][ ][ ]<br>CONSTANT PROBLEM…995<br>DK…...998 |

| | 29a. Can you please tell me which of the following best describes your knowledge of [METHOD]:<br>1. Know the method sufficiently well to counsel and recommend to client<br>2. Know little about the method and would not feel comfortable counseling or recommending<br>8. Do not know method | 29b. Have you ever recommended [METHOD] to clients at this facility? |
|---|---|---|
| (14) Natural methods (Rhythm, periodic abstinence, withdrawal) | COUNSEL & RECOMMEND……………..…1<br>KNOW LITTLE ABOUT……………..2 } → (15)<br>DO NOT KNOW……………………..8 } | Yes……..1<br>No………2 |
| (15) Exclusive breastfeeding method (LAM) | COUNSEL & RECOMMEND………………1<br>KNOW LITTLE ABOUT……………..2 } → Q30<br>DO NOT KNOW…………………….8 } | Yes……..1<br>No………2 |

Q30. **CHECK Q29A:**

PROVIDES AND/OR COUNSELS ANY FP METHOD (ANY Q29A(1-13) = 1 OR 2; Q29A(14-15)=1) ☐ ↓

DOES NOT PROVIDE AND DOES NOT COUNSEL ANY FP METHOD (ALL Q29A(1-13) = 3 OR 8 AND Q29A (14-15)= 2 OR 8) ☐ → **Q36**

**Now I would like to ask you specifically about the contraceptive methods that you provide.**
**(ASK ONLY ABOUT THE FAMILY PLANNING METHODS THE RESPONDENT IS PROVIDING – Q29b)**

| METHOD | Q31a. What is the minimum age that you would offer this [METHOD]? | Q31b. What is the maximum age that you would offer this [METHOD]? | Q31c. Is there a minimum number of children a person must have before you will offer [METHOD]? | Q31d. What is that minimum number of children? | Q31e. Do you require a partner's consent before you will provide [METHOD]? | Q31f. Would you offer METHOD to an unmarried person? |
|---|---|---|---|---|---|---|
| (1) Combined oral pills | [__\|__]<br>NO MIN…...93<br>DK……….98 | [__\|__]<br>NO MAX…….93<br>DK……….98 | YES . . .1<br>NO . . …2 → **Q31e**<br>DK…….8 → **Q31e** | [__\|__] | YES . . . .1<br>NO . . . . .2 | YES . . . .1<br>NO . . . . .2 |
| (2) Progestin-only pill | [__\|__]<br>NO MIN…...93<br>DK……….98 | [__\|__]<br>NO MAX…….93<br>DK……….98 | YES . . .1<br>NO . . …2 → **Q31e**<br>DK…….8 → **Q31e** | [__\|__] | YES . . . .1<br>NO . . . . .2 | YES . . . .1<br>NO . . . . .2 |
| (3) Injectables | [__\|__]<br>NO MIN…...93<br>DK……….98 | [__\|__]<br>NO MAX……..93<br>DK……….98 | YES . . .1<br>NO . . …2 → **Q31e**<br>DK…….8 → **Q31e** | [__\|__] | YES . . . .1<br>NO . . . . .2 | YES . . . .1<br>NO . . . . .2 |
| (4) Male condom | [__\|__]<br>NO MIN…...93<br>DK……….98 | [__\|__]<br>NO MAX……..93<br>DK……….98 | YES . . .1<br>NO . . …2 → **Q31e**<br>DK…….8 → **Q31e** | [__\|__] | YES . . . .1<br>NO . . . . .2 | YES . . . .1<br>NO . . . . .2 |
| (5) Female condom | [__\|__]<br>NO MIN…...93<br>DK……….98 | [__\|__]<br>NO MAX……..93<br>DK……….98 | YES . . .1<br>NO . . …2 → **Q31e**<br>DK…….8 → **Q31e** | [__\|__] | YES . . . .1<br>NO . . . . .2 | YES . . . .1<br>NO . . . . .2 |
| (6) Emergency contraceptive | [__\|__]<br>NO MIN…...93<br>DK……….98 | [__\|__]<br>NO MAX………93<br>DK……….98 | YES . . .1<br>NO . . …2 → **Q31e**<br>DK…….8 → **Q31e** | [__\|__] | YES . . . .1<br>NO . . . . .2 | YES . . . .1<br>NO . . . . .2 |
| (7) Spermicide | [__\|__]<br>NO MIN…...93<br>DK……….98 | [__\|__]<br>NO MAX…….93<br>DK……….98 | YES . . .1<br>NO . . …2 → **Q31e**<br>DK…….8 → **Q31e** | [__\|__] | YES . . . .1<br>NO . . . . .2 | YES . . . .1<br>NO . . . . .2 |

| METHOD | Q31a. What is the minimum age that you would offer this [METHOD]? | Q31b. What is the maximum age that you would offer this [METHOD]? | Q31c. Is there a minimum number of children a person must have before you will offer [METHOD]? | Q31d. What is that minimum number of children? | Q31e. Do you require a partner's consent before you will provide [METHOD]? | Q31f. Would you offer METHOD to an unmarried person? |
|---|---|---|---|---|---|---|
| (8) Diaphragm | [ ][ ]<br>NO MIN…...93<br>DK……….98 | [ ][ ]<br>NO MAX…….93<br>DK……….98 | YES . . .1<br>NO . . …2 →**Q30e**<br>DK…….8→**Q30e** | [ ][ ] | YES . . . .1<br>NO . . . . .2 | YES . . . .1<br>NO . . . . .2 |
| (9) SDM/Cycle beads | [ ][ ]<br>NO MIN…...93<br>DK……….98 | [ ][ ]<br>NO MAX…….93<br>DK……….98 | YES . . .1<br>NO . . …2 →**Q31e**<br>DK…….8→**Q31e** | [ ][ ] | YES . . . .1<br>NO . . . . .2 | YES . . . .1<br>NO . . . . .2 |
| (10) IUD | [ ][ ]<br>NO MIN…...93<br>DK……….98 | [ ][ ]<br>NO MAX……..93<br>DK……….98 | YES . . .1<br>NO . . …2 →**Q31e**<br>DK…….8→**Q31e** | [ ][ ] | YES . . . .1<br>NO . . . . .2 | YES . . . .1<br>NO . . . . .2 |
| (11) Implants | [ ][ ]<br>NO MIN…...93<br>DK……….98 | [ ][ ]<br>NO MAX...93<br>DK……….98 | YES . . .1<br>NO . . …2 →**Q31e**<br>DK…….8→**Q31e** | [ ][ ] | YES . . . .1<br>NO . . . . .2 | YES . . . .1<br>NO . . . . .2 |
| (12) Female sterilization | [ ][ ]<br>NO MIN…….93<br>DK……….98 | [ ][ ]<br>NO MAX...93<br>DK……….98 | YES . . .1<br>NO . . …2 →**Q31e**<br>DK…….8→**Q31e** | [ ][ ] | YES . . . .1<br>NO . . . . .2 | YES . . . .1<br>NO . . . . .2 |
| (13) Male sterilization | [ ][ ]<br>NO MIN….93<br>DK……….98 | [ ][ ]<br>NO MAX...93<br>DK……….98 | YES . . .1<br>NO . . …2 →**Q31e**<br>DK…….8→**Q31e** | [ ][ ] | YES . . . .1<br>NO . . . . .2 | YES . . . .1<br>NO . . . . .2 |

| | | | |
|---|---|---|---|
| Q32. | What do you do/tell the client when talking about FP to clients?<br><br>PROBE – Anything else?<br>**MULTIPLE RESPONSES POSSIBLE.**<br>**CIRCLE ALL MENTIONED.** | IDENTIFY REPRODUCTIVE GOALS OF CLIENT……A<br>PROVIDE INFORMATION ABOUT DIFFERENT FP METHODS……………………………….....……….B<br>DISCUSS THE CLIENT'S FP PREFERENCES……....C<br>HELP CLIENT SELECT A SUITABLE METHOD…..….D<br>EXPLAIN THE WAY TO USE THE SELECTED METHOD…………………...……………………..…E<br>EXPLAIN THE SIDE-EFFECTS……..……………….F<br>EXPLAIN SPECIFIC MEDICAL REASONS TO RETURN……………………………...…………..G<br>REQUEST FOR PARTNER'S CONSENT…………..…H<br><br>OTHERS_________________________________ X<br>(SPECIFY) | |
| Q33. | **CHECK Q29B:**<br><br>PROVIDES HORMONAL METHODS (PILL OF ANY TYPE, INJECTABLE, IUD, OR IMPLANTS: Q29B(1)=1 OR Q29B(2)=1 OR Q29B(3)=1 OR Q29B(10)=1 OR Q29B(11)=1) [ ] ↓ | DOES NOT PROVIDE HORMONAL METHODS (ALL OF THE FOLLOWING EQUAL "2" OR ARE SKIPPED: Q29B(1), Q29B(2), Q29B(3), Q29B(10), Q29B(11)) [ ] → | **Q35** |

| | | | |
|---|---|---|---|
| Q34. | What do you do for a new client who wants the pill or another hormonal method but is not having her menses?<br><br>**DO NOT READ OPTIONS**<br><br>PROBE WITH "Anything else?"<br><br>**MULTIPLE RESPONSES POSSIBLE.**<br>**CIRCLE ALL MENTIONED.** | QUESTION TO EXCLUDE PREGNANCY………….A<br>EXAMINE TO EXCLUDE PREGNANCY……………B<br>TEST TO EXCLUDE PREGNANCY…………………C<br>TELL HER TO COME BACK AT NEXT MENSES….D<br>TRY TO INDUCE MENSES…………………………..E<br>SUPPLY CONDOMS UNTIL NEXT MENSES………F<br>SUPPLY HORMONAL METHOD IF REASONABLY CERTAIN SHE IS NOT PREGNANT……………..G<br>SUPPLY HORMONAL METHOD AND CONDOMS, ASK HER TO USE CONDOMS UNTIL NEXT MENSES……………………………………………..H<br>JUST GIVE HORMONAL METHOD………………. J<br>REQUEST FOR PARTNER'S CONSENT…………K<br>OTHER______________________________ X<br>(SPECIFY) | |
| Q35. | Which kind of personal and financial records do you complete each time you provide a client with family planning services?<br><br>**MULTIPLE RESPONSES POSSIBLE.**<br>**CIRCLE ALL MENTIONED.** | A CLIENT RECORD CARD/FORM…………………..A<br>AN ENTRY IN THE FP REGISTER…......................B<br>AN ENTRY IN THE FACILITY LOGBOOK/ REGISTER…………………………………………C<br>INFORMAL NOTES IN A NOTEBOOK……………...D<br>A PAYMENT RECEIPT IF A FEE IS INVOLVED…..E<br>OTHER ______________________________X<br>(SPECIFY)<br>NO RECORD KEPT…………………………………...Y | |

| **INTEGRATION OF FAMILY PLANNING WITH OTHER SERVICES** | | | |
|---|---|---|---|
| Q36. | Which are the other services that you yourself provide to clients at this health facility? READ THE OPTIONS.<br><br>**MULTIPLE RESPONSES POSSIBLE.**<br>**CIRCLE ALL MENTIONED.** | ANTE-NATAL CARE……………………………………A<br>DELIVERY SERVICES…………………………………..B<br>POST-NATAL CARE……….......................................C<br>POST-ABORTION CARE……………………………….D<br>CHILD IMMUNIZATION…………………………………E<br>CHILD GROWTH MONITORING……………………….F<br>OTHER CURATIVE SERVICES FOR WOMEN………G<br>OTHER CURATIVE SERVICES FOR CHILDREN…..H<br>HIV/AIDS MANAGEMENT………………………………I<br>PMTCT……………………………………………………..J<br>VCT…………………………………………………………K<br>NONE OF THESE………………………………………..Y → | **Q89** |
| Q37. | **CHECK Q36:**<br><br>IF OPTION A (ANTENATAL CARE) IS CIRCLED ☐ ↓ | IF OPTION A (ANTENATAL CARE) IS **NOT** CIRCLED ☐ → | **Q46** |
| Q38. | During Antenatal care, do you provide information about FP routinely? | YES.............................................................1<br>NO...............................................................2 → | **Q44** |

| | | | |
|---|---|---|---|
| Q39. | What do you do/tell the client when talking about FP during antenatal care?<br><br>PROBE: "ANYTHING ELSE?"<br>**MULTIPLE RESPONSES POSSIBLE.**<br>**CIRCLE ALL MENTIONED.** | HELP THE WOMAN SELECT A SUITABLE METHOD FOR POST-DELIVERY……………………………………A<br>INFORM ABOUT THE IMPORTANCE OF USING FP BY 40 DAYS POSTPARTUM……………………………B<br>PROVIDE INFORMATION ON LAM…………………………C<br>EXPLAIN SIDE-EFFECTS……………………………………D<br>ENCOURAGE WOMEN TO WAIT FOR SOME TIME BEFORE THE NEXT PREGNANCY………………………E<br>REQUEST FOR PARTNER'S CONSENT…………………...F<br>OTHERS: ____________________________________ X<br>(SPECIFY) | |
| Q40. | Do you tell women where they can obtain an FP method after delivery? | YES.............................................................1<br>NO...............................................................2 | |
| Q41. | Do you use counseling job aids to provide FP services during antenatal care? | YES.............................................................1<br>NO...............................................................2 → | **Q46** |

| | | | |
|---|---|---|---|
| Q42. | Which job aids do you use?<br><br>PROBE: "ANYTHING ELSE?"<br>**MULTIPLE RESPONSES POSSIBLE.**<br>**CIRCLE ALL MENTIONED.** | NATIONAL STANDARD OF PRACTICE (SOP)<br>FOR FP SERVICES ……………………………………….A<br>FMOH FP SERVICES PERFORMANCE<br>STANDARDS………………………………………………B<br>WHO MEDICAL ELIGIBILITY CRITERIA (MERC)……….C<br>NURHI FP COUNSELING FLIP CHARTS…………………D<br>GATHER CHART…………………………………………..E<br>FP METHOD CHART (WALL TYPE) ………………………F<br>NURHI SMS FP COMMODITY TRACKING JOB AID ……G<br>OJT MANUALS (THREE COURSES)……………………….H<br>OTHER ________________________________X<br>(SPECIFY) | |
| Q43. | Who provided these job aids?<br><br>**MULTIPLE RESPONSES POSSIBLE.**<br>**CIRCLE ALL MENTIONED.** | NURHI ……………………………………….. A<br>MOBILE DL SIM …………………………..B<br>TSHIP………………………………………C<br>UNICEF……………………………………..D<br>SMOH………………………………………E<br>SFH…………………………………………F<br>OTHER ____________________X<br>(SPECIFY)<br>DON'T KNOW …………………………….Z | **ALL SKIP TO Q46** |
| Q44. | Why are you not able to provide FP information routinely during antenatal care visits?<br><br>**MULTIPLE RESPONSES POSSIBLE.**<br>**CIRCLE ALL MENTIONED.** | ADEQUATE CONTRACEPTIVE METHODS FREQUENTLY<br>UNAVAILABLE……………………………………………..A<br>AVAILABLE CONTRACEPTIVES OFTEN PAST<br>EXPIRATION DATE………………………………….….B<br>LACK OF STERILE EQUIPMENT SO NO POINT<br>DISCUSSING………………………………………….C<br>LACK OF FUNCTIONAL EQUIPMENT SO NO POINT<br>DISCUSSING………………………………………….D<br>NO INTEREST IN PROVIDING FP INFORMATION………..E<br>LACK KNOWLEDGE ABOUT FP………………………….F<br>DO NOT FEEL ADEQUATELY TRAINED TO PROVIDE<br>FP INFORMATION……………………………………….G<br>NO INTEREST IN FP ON THE PART OF THE PATIENTS…H<br>OVERLOAD OF WORK/NO TIME TO DISCUSS…………….I<br>NO NEED TO…………………………………………………K<br>NOT A PROFITABLE SERVICE TO PROVIDE……………..L<br><br>OTHERS______________________________X<br>(SPECIFY) | |
| Q45. | Would you be willing to include family planning information routinely in your antenatal care services/visits? | YES.........................................................1<br>NO..........................................................2 | |
| Q46. | **CHECK Q36:**<br><br>IF OPTION B (DELIVERY CARE) IS CIRCLED ☐ ↓ | IF OPTION B (DELIVERY CARE) IS **NOT** CIRCLED ☐ → | **Q53** |
| Q47. | During delivery care (anytime before they are discharged from your facility), do you provide information about FP routinely? | YES.........................................................1<br>NO..........................................................2 → | **Q51** |
| Q48. | What do you do/tell the client when talking about FP during delivery care?<br><br>PROBE: "ANYTHING ELSE?"<br>**MULTIPLE RESPONSES POSSIBLE.**<br>**CIRCLE ALL MENTIONED.** | HELP SELECT SUITABLE FP METHOD BY<br>40 DAYS POSTPARTUM…………………………….A<br>PROVIDE INFORMATION ON LAM……………………….B<br>EXPLAIN SIDE-EFFECTS…………………………………C<br>EXPLAIN SPECIFIC MEDICAL REASONS TO<br>RETURN…………………………………………………D<br>ENCOURAGE WOMEN TO WAIT SOME TIME<br>BEFORE THE NEXT PREGNANCY……..…………..E<br>REQUEST FOR PARTNER'S CONSENT………………..F<br>OTHER______________________________ X<br>(SPECIFY) | |
| Q49. | Do you tell women where they can obtain an FP method during delivery care? | YES.........................................................1<br>NO..........................................................2 | |
| Q50. | Do you counsel women on LAM during delivery care? | YES.........................................................1<br>NO.......................................................... 2 → | **All skip to Q53** |

| | | | |
|---|---|---|---|
| Q51. | Why are you not able to provide FP information routinely during delivery care?<br><br>PROBE: "ANYTHING ELSE?"<br>**MULTIPLE RESPONSES POSSIBLE.**<br>**CIRCLE ALL MENTIONED.** | ADEQUATE CONTRACEPTIVE METHODS FREQUENTLY UNAVAILABLE……A<br>AVAILABLE CONTRACEPTIVES OFTEN PAST EXPIRATION DATE……B<br>LACK OF STERILE EQUIPMENT SO NO POINT DISCUSSING……C<br>LACK OF FUNCTIONAL EQUIPMENT SO NO POINT DISCUSSING……D<br>NO INTEREST IN PROVIDING FP INFORMATION……E<br>LACK KNOWLEDGE ABOUT FP……F<br>DO NOT FEEL ADEQUATELY TRAINED TO PROVIDE FP INFORMATION……G<br>NO INTEREST IN FP ON THE PART OF THE PATIENTS…H<br>OVERLOAD OF WORK/NO TIME TO DISCUSS……I<br>NO NEED TO……K<br>NOT A PROFITABLE SERVICE TO PROVIDE……L<br><br>OTHERS______________X<br>(SPECIFY) | |
| Q52. | Would you be willing to include family planning information routinely in your delivery care services? | YES……1<br>NO……2 | |
| Q53. | **CHECK Q36:**<br>IF OPTION C (POST-NATAL CARE) IS CIRCLED ☐ ↓ | IF OPTION C (POST-NATAL CARE) IS **NOT** CIRCLED ☐ → | **Q62** |
| Q54. | During post-natal care visits, do you provide information about FP routinely? | YES……1<br>NO……2 → | **Q60** |
| Q55. | What do you do/tell the client when talking about FP during post-natal care visits?<br><br>PROBE: "ANYTHING ELSE?"<br>**MULTIPLE RESPONSES POSSIBLE.**<br>**CIRCLE ALL MENTIONED.** | HELP SELECT SUITABLE FP METHOD BY 40 DAYS POSTPARTUM……A<br>PROVIDE INFORMATION ON LAM……B<br>EXPLAIN SIDE-EFFECTS……C<br>EXPLAIN SPECIFIC MEDICAL REASONS TO RETURN……D<br>ENCOURAGE WOMEN TO WAIT SOME TIME BEFORE THE NEXT PREGNANCY……E<br>REQUEST FOR PARTNER'S CONSENT……F<br>OTHER______________ X<br>(SPECIFY) | |
| Q56. | Do you tell women where they can obtain an FP method during post-natal care visits? | YES……1<br>NO……2 | |
| Q57. | Do you use counseling job aids to provide FP services during postnatal care visits? | YES……1<br>NO……2 → | **Q62** |
| Q58. | Which job aids do you use?<br><br>PROBE: "ANYTHING ELSE?"<br>**MULTIPLE RESPONSES POSSIBLE.**<br>**CIRCLE ALL MENTIONED.** | NATIONAL STANDARD OF PRACTICE (SOP) FOR FP SERVICES ……A<br>FMOH FP SERVICES PERFORMANCE STANDARDS……B<br>WHO MEDICAL ELIGIBILITY CRITERIA (MERC)……C<br>NURHI FP COUNSELING FLIP CHARTS……D<br>GATHER CHART……E<br>FP METHOD CHART (WALL TYPE) ……F<br>NURHI SMS FP COMMODITY TRACKING JOB AID ……G<br>OJT MANUALS (THREE COURSES)……H<br>OTHER ______________X<br>(SPECIFY) | |
| Q59. | Who provided these job aids? | NURHI …… A<br>MOBILE DL SIM ……B<br>TSHIP……C<br>UNICEF……D<br>SMOH……E<br>SFH……F<br>OTHER ______________X<br>(SPECIFY)<br>} → (A–X)<br>DON'T KNOW ……Z | **ALL SKIP TO Q62** |

| | | | |
|---|---|---|---|
| Q60. | Why are you not able to provide FP information routinely during post-natal care visits?<br><br>PROBE: "ANYTHING ELSE?"<br>**MULTIPLE RESPONSES POSSIBLE.**<br>**CIRCLE ALL MENTIONED.** | ADEQUATE CONTRACEPTIVE METHODS FREQUENTLY UNAVAILABLE……A<br>AVAILABLE CONTRACEPTIVES OFTEN PAST EXPIRATION DATE……B<br>LACK OF STERILE EQUIPMENT SO NO POINT DISCUSSING……C<br>LACK OF FUNCTIONAL EQUIPMENT SO NO POINT DISCUSSING……D<br>NO INTEREST IN PROVIDING FP INFORMATION……E<br>LACK KNOWLEDGE ABOUT FP……F<br>DO NOT FEEL ADEQUATELY TRAINED TO PROVIDE FP INFORMATION……G<br>NO INTEREST IN FP ON THE PART OF THE PATIENTS…H<br>OVERLOAD OF WORK/NO TIME TO DISCUSS……I<br>NO NEED TO……K<br>NOT A PROFITABLE SERVICE TO PROVIDE……L<br><br>OTHERS______X<br>(SPECIFY) | |
| Q61. | Would you be willing to include family planning information routinely in your postnatal care services? | YES……1<br>NO……2 | |
| Q62. | **CHECK Q36:**<br><br>IF OPTION D (POST-ABORTION CARE) IS CIRCLED ☐ ↓ | IF OPTION D (POST-ABORTION CARE) IS **NOT** CIRCLED ☐ → | **Q71** |
| Q63. | During a post abortion care, do you provide information about FP routinely? | YES……1<br>NO……2 → | **Q69** |
| Q64. | What do/tell the client when talking about FP during post abortion care visits?<br><br>PROBE: "ANYTHING ELSE?"<br>**MULTIPLE RESPONSES POSSIBLE.**<br>**CIRCLE ALL MENTIONED.** | IDENTIFY REPRODUCTIVE GOALS OF WOMAN……A<br>PROVIDE INFORMATION ABOUT DIFFERENT FP METHODS……B<br>DISCUSS THE CLIENT'S FP PREFERENCES……C<br>HELP WOMEN SELECT A SUITABLE METHOD……D<br>EDUCATE WOMEN TO USE THE SELECTED METHOD……E<br>INFORM ABOUT HOW SOON AFTER ABORTION SHE MAY BECOME PREGNANT IF NOT USING CONTRACEPTION……F<br>EXPLAIN SIDE-EFFECTS……G<br>EXPLAIN SPECIFIC MEDICAL REASONS TO RETURN……H<br>REQUEST FOR PARTNER'S CONSENT……I<br>OTHERS: ______ X<br>(SPECIFY) | |
| Q65. | Do you tell women where they can obtain an FP method during post abortion care visits? | YES……1<br>NO……2 | |
| Q66. | Do you use counseling job aids to provide FP services during post abortion care visits? | YES……1<br>NO……2 → | **Q71** |
| Q67. | Which job aids do you use?<br><br>PROBE: "ANYTHING ELSE?"<br>**MULTIPLE RESPONSES POSSIBLE.**<br>**CIRCLE ALL MENTIONED.** | NATIONAL STANDARD OF PRACTICE (SOP) FOR FP SERVICES……A<br>FMOH FP SERVICES PERFORMANCE STANDARDS……B<br>WHO MEDICAL ELIGIBILITY CRITERIA (MERC)…C<br>NURHI FP COUNSELING FLIP CHARTS……D<br>GATHER CHART……E<br>FP METHOD CHART (WALL TYPE) ……F<br>NURHI SMS FP COMMODITY TRACKING JOB AID ……G<br>OJT MANUALS (THREE COURSES)……H<br>OTHER ______X<br>(SPECIFY) | |

| | | | |
|---|---|---|---|
| Q68. | Who provided these job aids? | NURHI …………………………………….. A<br>MOBILE DL SIM ……………………………..B<br>TSHIP…………………………………………C<br>UNICEF………………………………………..D<br>SMOH…………………………………………E<br>SFH……………………………………………F<br>OTHER ______________________X<br>(SPECIFY)<br>DON'T KNOW ………………………………Z | **ALL SKIP TO Q71** |
| Q69. | Why are you not able to provide FP information routinely during post abortion care visits?<br><br>PROBE: "ANYTHING ELSE?"<br>**MULTIPLE RESPONSES POSSIBLE.**<br>**CIRCLE ALL MENTIONED.** | ADEQUATE CONTRACEPTIVE METHODS FREQUENTLY UNAVAILABLE………………………A<br>AVAILABLE CONTRACEPTIVES OFTEN PAST EXPIRATION DATE……………………..………B<br>LACK OF STERILE EQUIPMENT SO NO POINT DISCUSSING…………………………………....C<br>LACK OF FUNCTIONAL EQUIPMENT SO NO POINT DISCUSSING……………………....D<br>NO INTEREST IN PROVIDING FP INFORMATION…………………………………..E<br>LACK KNOWLEDGE ABOUT FP…………………...F<br>DO NOT FEEL ADEQUATELY TRAINED TO PROVIDE FP INFORMATION………………………..G<br>NO INTEREST IN FP ON THE PART OF THE PATIENTS…………………………………………H<br>OVERLOAD OF WORK/NO TIME TO DISCUSS…...I<br>NO NEED TO……………………………………………K<br>NOT A PROFITABLE SERVICE TO PROVIDE.…….L<br>OTHERS______________________________X<br>(SPECIFY) | |
| Q70. | Would you be willing to include family planning information routinely in your post abortion care services/visits? | YES.................................................................1<br>NO..................................................................2 | |
| Q71. | **CHECK Q36:**<br><br>IF **EITHER** OPTION E (CHILD IMMUNIZATION) OR OPTION F (CHILD GROWTH MONITORING) IS CIRCLED ☐ ↓ | IF **NEITHER** OPTION E (CHILD IMMUNIZATION) **NOR** OPTION F (CHILD GROWTH MONITORING) IS CIRCLED ☐ → | Q77 |
| Q72. | During <u>child immunization/child growth monitoring</u>, do you provide information about FP routinely? | YES.................................................................1<br>NO……………………………………………….2 → | Q75 |
| Q73. | What do you do/tell clients when talking about FP during child immunization or child growth monitoring visits?<br><br>PROBE: "ANYTHING ELSE?"<br>**MULTIPLE RESPONSES POSSIBLE.**<br>**CIRCLE ALL MENTIONED.** | IDENTIFY REPRODUCTIVE GOALS OF WOMAN..............................................................A<br>PROVIDE INFORMATION ABOUT DIFFERENT FP METHODS……………………………………….B<br>DISCUSS THE CLIENT'S FP PREFERENCES……..C<br>HELP WOMEN SELECT A SUITABLE METHOD…..D<br>EDUCATE WOMEN TO USE THE SELECTED METHOD……………………….…………...….E<br>EXPLAIN SIDE-EFFECTS…………………….….…F<br>EXPLAIN SPECIFIC MEDICAL REASONS TO RETURN……………….………………………...…G<br>REQUEST FOR PARTNER'S CONSENT……………H<br>OTHERS: ____________________________ X<br>(SPECIFY) | |
| Q74. | Do you tell women where they can obtain an FP method? | Yes.................................................................1<br>No..................................................................2 | **All skip to Q77** |

| | | | |
|---|---|---|---|
| Q75. | Why are you not able to provide FP information routinely?<br><br>PROBE: "ANYTHING ELSE?"<br>**MULTIPLE RESPONSES POSSIBLE.**<br>**CIRCLE ALL MENTIONED.** | ADEQUATE CONTRACEPTIVE METHODS FREQUENTLY UNAVAILABLE……… ……..A<br>AVAILABLE CONTRACEPTIVES OFTEN PAST EXPIRATION DATE……………………… …..B<br>LACK OF STERILE EQUIPMENT SO NO POINT DISCUSSING……………………………………..C<br>LACK OF FUNCTIONAL EQUIPMENT SO NO POINT DISCUSSING………………………..D<br>NO INTEREST IN PROVIDING FP INFORMATION…………………………………….E<br>LACK KNOWLEDGE ABOUT FP…………………….F<br>DO NOT FEEL ADEQUATELY TRAINED TO PROVIDE FP INFORMATION…………………...G<br>NO INTEREST IN FP ON THE PART OF THE PATIENTS…………………………………….......H<br>OVERLOAD OF WORK/NO TIME……………….......I<br>NO NEED TO……………………………………….K<br>NOT A PROFITABLE SERVICE TO PROVIDE…….L<br><br>OTHERS________________________________X<br>(SPECIFY) | |
| Q76. | Would you be willing to include family planning information routinely in your child immunization or child growth monitoring visits? | YES.............................................................1<br>NO..............................................................2 | |
| Q77. | **CHECK Q36**:<br><br>IF **EITHER** OPTION G (CURATIVE SERVICES FOR WOMEN) OR H (CURATIVE SERVICES FOR CHILDREN) IS CIRCLED ☐ ↓ | IF **NEITHER** OPTION G (CURATIVE SERVICES FOR WOMEN) **NOR** H (CURATIVE SERVICES FOR CHILDREN) IS CIRCLED ☐ → | **Q83** |
| Q78. | While providing curative services to women or children, do you provide information on FP routinely? | YES.............................................................1<br>NO..............................................................2 → | **Q81** |
| Q79. | What are the main activities you follow when talking about FP to clients?<br><br>PROBE: "ANYTHING ELSE?"<br>**MULTIPLE RESPONSES POSSIBLE.**<br>**CIRCLE ALL MENTIONED.** | IDENTIFY REPRODUCTIVE GOALS OF WOMAN..........................................................A<br>PROVIDE INFORMATION ABOUT DIFFERENT FP METHODS……………………………………..B<br>DISCUSS THE CLIENT'S FP PREFERENCES….C<br>HELP WOMEN SELECT A SUITABLE METHOD…………………………………………...D<br>EDUCATE WOMEN TO USE THE SELECTED METHOD…………………………………………E<br>EXPLAIN SIDE-EFFECTS…………………………F<br>EXPLAIN SPECIFIC MEDICAL REASONS TO RETURN…………………………………………G<br>OTHERS: ____________________________ X<br>(SPECIFY) | |
| Q80. | Do you tell women where they can obtain an FP method? | YES.............................................................1<br>NO..............................................................2 } → | **All skip to Q83** |
| Q81. | Why are you not able to provide FP information routinely?<br><br>PROBE: "ANYTHING ELSE?"<br>**MULTIPLE RESPONSES POSSIBLE.**<br>**CIRCLE ALL MENTIONED.** | ADEQUATE CONTRACEPTIVE METHODS FREQUENTLY UNAVAILABLE……… ……..A<br>AVAILABLE CONTRACEPTIVES OFTEN PAST EXPIRATION DATE……………………… …..B<br>LACK OF STERILE EQUIPMENT SO NO POINT DISCUSSING……………………………………..C<br>LACK OF FUNCTIONAL EQUIPMENT SO NO POINT DISCUSSING………………………..D<br>NO INTEREST IN PROVIDING FP INFORMATION…………………………………….E<br>LACK KNOWLEDGE ABOUT FP…………………….F<br>DO NOT FEEL ADEQUATELY TRAINED TO PROVIDE FP INFORMATION…………………...G<br>NO INTEREST IN FP ON THE PART OF THE PATIENTS…………………………………….......H<br>OVERLOAD OF WORK/NO TIME……………….......I<br>NO NEED TO……………………………………….K<br>NOT A PROFITABLE SERVICE TO PROVIDE…….L<br><br>OTHERS________________________________X<br>(SPECIFY) | |

| | | | |
|---|---|---|---|
| Q82. | Would you be willing to include family planning information routinely in your curative care services/visits for women or children? | YES....1<br>NO....2 | |
| Q83. | **CHECK Q36:**<br><br>IF **ANY** OF THE OPTIONS I (HIV/AIDS MANAGEMENT), OPTION J (PMTCT), OR K (VCT) ARE CIRCLED ☐ ↓ | IF **NONE** OF THE OPTIONS I (HIV/AIDS MANAGEMENT), OPTION J (PMTCT), OR K (VCT) ARE CIRCLED ☐ → | **Q89** |
| Q84. | While providing HIV-related services (HIV/AIDS management, PMTCT, and/or VCT) to women and men, do you provide information on FP routinely? | YES....1<br>NO....2 → | **Q87** |

| | | | |
|---|---|---|---|
| Q85. | What are the main activities you follow when talking about FP to clients?<br><br>PROBE: "ANYTHING ELSE?"<br>**MULTIPLE RESPONSES POSSIBLE.**<br>**CIRCLE ALL MENTIONED.** | IDENTIFY REPRODUCTIVE GOALS OF WOMAN...A<br>PROVIDE INFORMATION ABOUT DIFFERENT FP METHODS....B<br>DISCUSS THE CLIENT'S FP PREFERENCES....C<br>HELP WOMEN SELECT A SUITABLE METHOD....D<br>EDUCATE WOMEN TO USE THE SELECTED METHOD....E<br>EXPLAIN SIDE-EFFECTS....F<br>EXPLAIN SPECIFIC MEDICAL REASONS TO RETURN....G<br>DISCUSS HIV/AIDS PREVENTION METHODS....H<br>DISCUSS METHODS NOT RECOMMENDED FOR HIV POSITIVE (LAM, IUD)....I<br>RECOMMEND ALWAYS USE CONDOM IN ADDITION TO OTHER FP METHODS....J<br>REQUEST FOR PARTNER'S CONSENT....K<br>OTHERS: ______________ X<br>(SPECIFY) | |
| Q86. | Do you tell women where they can obtain an FP method? | YES....1<br>NO....2 } → | **All skip to Q89** |
| Q87. | Why are you not able to provide FP information routinely?<br><br>PROBE: "ANYTHING ELSE?"<br>**MULTIPLE RESPONSES POSSIBLE.**<br>**CIRCLE ALL MENTIONED.**<br>. | ADEQUATE CONTRACEPTIVE METHODS FREQUENTLY UNAVAILABLE....A<br>AVAILABLE CONTRACEPTIVES OFTEN PAST EXPIRATION DATE....B<br>LACK OF STERILE EQUIPMENT SO NO POINT DISCUSSING....C<br>LACK OF FUNCTIONAL EQUIPMENT SO NO POINT DISCUSSING....D<br>NO INTEREST IN PROVIDING FP INFORMATION....E<br>LACK KNOWLEDGE ABOUT FP....F<br>DO NOT FEEL ADEQUATELY TRAINED TO PROVIDE FP INFORMATION....G<br>NO INTEREST IN FP ON THE PART OF THE PATIENTS....H<br>OVERLOAD OF WORK/NO TIME....I<br>NO NEED TO....K<br>NOT A PROFITABLE SERVICE TO PROVIDE....L<br><br>OTHERS______________X<br>(SPECIFY) | |
| Q88. | Would you be willing to include family planning information routinely in your HIV-related services/visits for women and men? | YES....1<br>NO....2 | |

| **Integrated Supportive Supervision (ISS)** | | | |
|---|---|---|---|
| Q89. | Have you received an integrated supportive supervision visit in the last three months? | YES....1<br>NO....2 → | **Q93** |
| Q90. | Which organization/group visited you? | NURHI....A<br>STATE GOVERNMENT....B<br>FEDERAL GOVERNMENT....C<br>OTHER ______________ X<br>(SPECIFY)<br>DON'T KNOW.... Z | |

QUESTIONNAIRE IDENTIFICATION NO:[__|__|__|__|__|__|__|__]
5 digit facility code + 3 digit prov code

| | | | |
|---|---|---|---|
| Q91. | What type of feedback did you receive? | RECEIVED VERBAL/WRITTEN FEEDBACK..........A<br>RECEIVED HANDS ON/PRACTICAL TRAINING...B<br>RECEIVED DEMONSTRATION.............................C<br>OTHER_________________________________X<br>(SPECIFY)<br><br>RECEIVED NO FEEDBACK...................................Y | **A-X SKIP TO Q93** |
| Q92. | Would you like to receive feedback? | YES..................................................................1<br>NO.....................................................................2 | |
| Q93. | Is this facility linked with another organization that provides family planning methods and materials at a discounted rate or for free (for example PPFN or SFH)? | YES...............................................................1<br>NO................................................................2 →<br>DON'T KNOW...............................................8 → | <br>**Q95**<br>**Q95** |

| | | | |
|---|---|---|---|
| Q94a. | What is the name of the organization? | Q94b. What year did this facility begin to associate with each organization named? | |
| | 1. | YEAR …………….. . . . [ ][ ][ ][ ]<br>DON'T KNOW . . . . . . . . . . . . . . . . . 9998 | |
| | 2. | YEAR …………….. . . . [ ][ ][ ][ ]<br>DON'T KNOW . . . . . . . . . . . . . . . . . 9998 | |
| | 3. | YEAR …………….. . . . [ ][ ][ ][ ]<br>DON'T KNOW . . . . . . . . . . . . . . . . . 9998 | |
| | 4. | YEAR …………….. . . . [ ][ ][ ][ ]<br>DON'T KNOW . . . . . . . . . . . . . . . . . 9998 | |
| Q95. | RECORD THE TIME<br><br>IN 24 HOUR FORMAT | HOUR ………… [ ][ ]    MINUTES …….. [ ][ ] | |

| |
|---|
| Thank you very much for taking the time to answer my questions. Once again, any information you have given will be kept completely confidential. Have a good day! |
| **COMMENTS:** |
