# Supplementary material for: Facility-level characteristics associated with family planning and child immunization services integration in urban areas of Nigeria: a longitudinal analysis
Source: BMC Public Health. 2021 Jul 12;21:1379. doi: 10.1186/s12889-021-11436-x (PMC8274034; doi:10.1186/s12889-021-11436-x)
Supplement: Supplementary file 5 — Additional file 5: Appendix A. Additional Details on Construction of the Integration Indexes. Appendix B. Association Between NURHI Intervention and Other Facility Characteristics and Provider and Facility Integration Index Scores. [file 12889_2021_11436_MOESM5_ESM.docx]

# Appendix A: Additional Details on Construction of the Integration Indexes

The integration indexes were conceptualized as a secondary analysis; we therefore selected eight variables from the existing data to use as index constructs. These variables reflect the attributes identified in a literature review as well as guidance from the Ministry of Health (Table 2). Several variables refer to child health services visits, which include either immunization or growth monitoring visits, but not sick child visits. We retained variables that refer to child growth monitoring or child health services because analysis of client exit interview data shows that 98% of women state that immunization is the primary purpose of their child health services visit. We assess the quality of integrated family planning service provision by including a measure of the extent to which providers discuss key family planning topics with a client during a child health service visit. We include social norm-based service barriers by quantifying the extent to which providers at a facility require spousal consent prior to providing contraception during an integrated visit.

To construct the indexes, the eight variables were entered into a principal components analysis (PCA). Based on the resulting eigenvalues and scree plot, we kept two components that capture the majority of variation in the data. We then examined the factor loading scores to determine which dimensions of integration were represented by each component. We constructed the PII and FII using weights calculated with the factor loading scores from each variable within the two retained components. We generated a PII score and FII score for each facility by multiplying the variables included in each component by their associated weights and summing the values. Each facility was classified as having “low integration” (index score 0 - 3.29), “medium integration” (3.30 - 6.59) or “high integration” (6.60 -10.00) for both the PII and FII scores. We gauged the internal coherence of the indexes by comparing facility characteristics and index scores across low, medium, and high integration groups. Statistically significant differences in facility characteristics and overall index scores across all groups for both indexes indicate strong internal coherence. We investigated index robustness by observing whether classifications of facilities with high index scores shift when different sub-sets of variables are entered into the PCA. The PII is highly robust to the inclusion of different sub-sets of variables in the model, while the FII shows more sensitivity. Additional information about the indexes has been published previously and is available from the first author ([38](#_ENREF_38)).

**Appendix B: Association Between NURHI Intervention and Other Facility Characteristics and Provider and Facility Integration Index Scores**

Table 5: Association Between NURHI Intervention and Other Facility Characteristics and Provider Integration Index Score

| Characteristic | Model 1 | Model 2 | Model 3 | Model 4 | Model 5 |
| --- | --- | --- | --- | --- | --- |
| Time | **1.01** | **1.03** | **1.03** | **1.00** | **0.90** |
|  | (0.20) | (0.21) | (0.21) | (0.21) | (0.21) |
| NURHI facility | **1.41** | **1.44** | **1.32** | 0.35 | 0.14 |
|  | (0.29) | (0.29) | (0.30) | (0.30) | (0.31) |
| NURHI intervention (time*NURHI facility) | **-0.68** | **-0.62** | **-0.64** | -0.51 | -0.36 |
|  | (0.32) | (0.32) | (0.32) | (0.32) | (0.32) |
| Facility FP client load |  | **-0.0101** | **-0.0120** | **-0.0105** | **-0.0100** |
|  |  | (0.0049) | (0.0049) | (0.0043) | (0.0044) |
| Provider experience |  |  | 0.24 | **-0.42** | **-0.60** |
|  |  |  | (0.17) | (0.18) | (0.19) |
| Benin |  |  |  | **1.58** | **1.41** |
|  |  |  |  | (0.43) | (0.43) |
| Ibadan |  |  |  | **1.97** | **1.60** |
|  |  |  |  | (0.43) | (0.44) |
| Ilorin |  |  |  | **1.88** | **1.77** |
|  |  |  |  | (0.43) | (0.43) |
| Kaduna |  |  |  | 0.71 | 0.74 |
|  |  |  |  | (0.39) | (0.39) |
| Zaria |  |  |  | **1.05** | **0.98** |
|  |  |  |  | (0.43) | (0.43) |
| Public Facility |  |  |  | **2.11** | **2.04** |
|  |  |  |  | (0.31) | (0.31) |
| Hospital |  |  |  | 0.15 | 0.09 |
|  |  |  |  | (0.27) | (0.27) |
| Proportion providers received any in-service FP training |  |  |  |  | **1.15** |
|  |  |  |  |  | (0.31) |
| Constant | **5.02** | **5.06** | **4.93** | **3.68** | **3.67** |
|  | (0.19) | (0.19) | (0.28) | (0.43) | (0.43) |
| Observations | 751 | 751 | 751 | 751 | 751 |
| Notes: Beta coefficients in bold indicate p<0.05. Robust standard errors in parentheses. | | | | | |
| FP: Family Planning. ‘Facility FP client load' unit is 10 additional FP patients per provider per year, i.e., an additional 10 FP patients per year per provider is associated with a 0.01 decrease in Provider Integration Index score. | | | | | |
| Unit for provider experience is 10 additional years, i.e., an additional 10 years of average experience among providers within a facility is associated with a 0.60 decrease in Provider Integration Index score. | | | | | |

Table 6: Association Between NURHI Intervention and Other Facility Characteristics and Facility Integration Index Score

| Characteristic | Model 1 | Model 2 | Model 3 | Model 4 | Model 5 |
| --- | --- | --- | --- | --- | --- |
| Time | 0.30 | 0.31 | 0.28 | 0.31 | 0.21 |
|  | (0.20) | (0.20) | (0.20) | (0.20) | (0.20) |
| NURHI Facility | **1.31** | **1.33** | **1.24** | **0.57** | 0.38 |
|  | (0.26) | (0.26) | (0.26) | (0.25) | (0.25) |
| NURHI intervention | -0.04 | -0.01 | 0.02 | 0.06 | 0.21 |
| (time*NURHI facility) | (0.31) | (0.31) | (0.31) | (0.31) | (0.31) |
| Facility FP client load |  | -0.0056 | -0.0057 | -0.0063 | -0.0059 |
|  |  | (0.0058) | (0.0061) | (0.0057) | (0.0059) |
| Average years experience of providers |  |  | 0.10 | -0.21 | **-0.37** |
|  |  |  | (0.15) | (0.16) | (0.16) |
| Benin |  |  | -0.53 | -0.03 | -0.18 |
|  |  |  | (0.42) | (0.42) | (0.42) |
| Ibadan |  |  | -0.53 | -0.36 | -0.67 |
|  |  |  | (0.42) | (0.41) | (0.41) |
| Ilorin |  |  | -0.20 | -0.17 | -0.26 |
|  |  |  | (0.39) | (0.37) | (0.37) |
| Kaduna |  |  | **-0.86** | **-0.82** | **-0.80** |
|  |  |  | (0.41) | (0.39) | (0.39) |
| Zaria |  |  | **-1.18** | **-1.19** | **-1.23** |
|  |  |  | (0.46) | (0.44) | (0.44) |
| Public Facility |  |  |  | **1.65** | **1.58** |
|  |  |  |  | (0.29) | (0.28) |
| Hospital |  |  |  | **0.82** | **0.77** |
|  |  |  |  | (0.26) | (0.25) |
| Proportion providers received any in-service FP training |  |  |  |  | **1.02** |
|  |  |  |  |  | (0.28) |
| Constant | **5.80** | **5.81** | **6.32** | **5.58** | **5.58** |
|  | (0.18) | (0.18) | (0.39) | (0.45) | (0.45) |
| Observations | 765 | 765 | 762 | 762 | 762 |
| Notes: Beta coefficients in bold indicate p<0.05. Robust standard errors in parentheses. | | | | | |
| FP: Family Planning. Facility FP client load' unit is 10 additional FP patients per provider per year, i.e., an additional 10 FP patients per year per provider is associated with a 0.01 decrease in Provider Integration Index score. | | | | | |
| Unit for provider experience is 10 additional years, i.e., an additional 10 years of average experience among providers within a facility is associated with a 0.60 decrease in Provider Integration Index score. | | | | | |
